# Mathematical Modeling of MPNs Offers Understanding and Decision Support for Personalized Treatment

**DOI:** 10.3390/cancers12082119

**Published:** 2020-07-30

**Authors:** Johnny T. Ottesen, Rasmus K. Pedersen, Marc J. B. Dam, Trine A. Knudsen, Vibe Skov, Lasse Kjær, Morten Andersen

**Affiliations:** 1 IMFUFA, Department of Science and Environment, Roskilde University, 4000 Roskilde, Denmark; rakrpe@ruc.dk (R.K.P.); mjbd@ruc.dk (M.J.B.D.); moan@ruc.dk (M.A.); 2 Department of Haematology, Zealand University Hospital, Roskilde, 2022 Roskilde, Denmark; trak@regionsjaelland.dk (T.A.K.); vihs@regionsjaelland.dk (V.S.); laskj@regionsjaelland.dk (L.K.)

**Keywords:** blood cancer, myeloproliferative neoplasms, *JAK2*V617F dynamics, mathematical modeling, personalized treatment

## Abstract

(1) Background: myeloproliferative neoplasms (MPNs) are slowly developing hematological cancers characterized by few driver mutations, with *JAK2*V617F being the most prevalent. (2) Methods: using mechanism-based mathematical modeling (MM) of hematopoietic stem cells, mutated hematopoietic stem cells, differentiated blood cells, and immune response along with longitudinal data from the randomized Danish DALIAH trial, we investigate the effect of the treatment of MPNs with interferon-α2 on disease progression. (3) Results: At the population level, the *JAK2*V617F allele burden is halved every 25 months. At the individual level, MM describes and predicts the *JAK2*V617F kinetics and leukocyte- and thrombocyte counts over time. The model estimates the patient-specific treatment duration, relapse time, and threshold dose for achieving a good response to treatment. (4) Conclusions: MM in concert with clinical data is an important supplement to understand and predict the disease progression and impact of interventions at the individual level.

## 1. Introduction

Hematopoietic stem cells (HSCs) are harbored in the bone marrow, where they self-renew and proliferate into progenitor cells, which further differentiate into next-generation progenitor cells, ultimately becoming mature white blood cells (WBCs), red blood cells (RBCs), or platelets (PBCs) [1]. Such healthy hematopoiesis is tightly regulated and produces daily as many cells as there are stars in the Milky Way i.e., around 10^11^ cells per day [2]. Disturbances of this feedback system—e.g., malignant mutations escaping repair mechanisms in the HSCs—may be lethal. Competitive clones may cause myeloid neoplasms, including acute myeloid leukemia (AML) or Philadelphia negative myeloproliferative neoplasms (MPNs). The MPNs may further be divided into different subtypes: essential thrombocythemia (ET), polycythemia vera (PV), and primary myelofibrosis (PMF) [3]. In the biological continuum from early cancer stages (ET and PV) to the advanced myelofibrosis stage, these different subtypes may transform into each other, but also MPNs into AML [4,5,6]. The diagnoses are based on different diagnostic criteria, such as elevation in different cell counts (RBC, WBC, or PBC) or mutations in *JAK2, CALR*, or *MPL*. MPNs have an increasing but low incidence, with the annual incidence rates per 100,000 citizens being 1.6 for ET, 0.84 for PV, and 0.47 for PMF, respectively [7,8].

During blood cancer development, a healthy HSC population evolves into a malignant population due to accumulated mutations and natural selection. The malignant stem cells adapt to the microenvironment in the bone marrow. If the malignant stem cells are sufficiently fit, they may outcompete the healthy stem cells and acquire further malignant mutations—e.g., becoming therapeutic-resistant and metastasizing [9]. The risk of the acquisition of additional mutations is proportional to the number of malignant cells. Thus, to avoid additional mutations, which are often more aggressive, early therapeutic intervention is preferable as long as the intervention has limited side effects and is non-mutagenic. Hence, in MPNs treatment with interferon-α2 (IFN) may by preferred over hydroxyurea (HU) [10].

Blood cancers are driven by mutations in stem cells, but studying the dynamics of both healthy and malignant stem cells in humans is not practically feasible at a high frequency. However, circulating peripheral blood cell counts are easily accessible, but investigating the bone marrow stem cell populations from peripheral blood cell counts is challenging in the clinical setting. In this regard, the mathematical discipline of solving inverse problems offers a useful tool. Hence, mathematical modelling plays a crucial role in better understanding the dynamics of normal hematopoiesis and the development of blood cancers.

Generally, cancers have a strong immune component [11]. Therefore, one needs to consider relevant parts of the immune system to model cancer evolution and treatment effect. Hydroxyurea is the most-used treatment for patients with MPNs worldwide, but patients with low-risk disease are often observed without any cytoreductive treatment (the “watch and wait” strategy). In Denmark and a few other places, off-label pegylated interferon-α2, mostly interferon-α-2b (PegIntron) and interferon-α-2a (Pegasys), have been used for the treatment of patients with MPNs with success for years, as demonstrated in the Danish DALIAH trial (see Material and Method). A statistical data-driven analysis of this study has most recently been reported [12].

Mathematical modelling plays an important role in the study of cancer [13,14,15,16], but only a few studies have addressed MPNs [17,18]. In the present paper, we apply a patient-specific mechanism-based mathematical model, the Cancitis model [19,20,21], to study the dynamics of Philadelphia chromosome-negative, *JAK2*V617F-positive MPN patients from the DALIAH trial. Furthermore, we investigate if IFN treatment responses are consistent with the Cancitis model and if the model can be used to predict the individual treatment responses. The Cancitis model takes into account the dynamics of the hematopoietic stem cell pool and the composition of the peripheral blood, including the competition between wild-type and mutated clones and the immune-mediated feedback where immune cells stimulate the expansion of the stem cell pool. In [19] and [20], it is documented that the Cancitis model describes cytokines (IL-1β, IL-1RA, IL-2R, IL-6, IL-8, IL-10, and IL-12), C-reactive protein (CRP), and lactate dehydrogenase (LDH) independently measured but associated with the *JAK2*V617F-positive MPNs. Data from a few untreated patients with MPNs have been used to calibrate the Cancitis model to natural disease progression. Then, longitudinal data (up to 5 years) from the randomized DALIAH trial, testing the treatment efficacy of IFN in patients with MPNs, were used to calibrate the Cancitis model to the individual interventions. Scenarios where the Cancitis model is calibrated to the first three data points (*JAK2*V617F allele burden) and predicts the outcome of the following trajectory are presented and show a good agreement in the majority of the included 63 patients.

In the mathematical model, we include the effect of IFN on the death rate of malignant stem cells [22,23]. Dormant stem cells may be activated by IFN [24], and stem cell quiescence-enforcing mechanisms may be suppressed by IFN [25]. A standard pharmacokinetics equation is included for the uptake of IFN to take into account dose–response relationships. The focus of the present paper is the effect of IFN by its capability to enhance the malignant stem cell death rate.

In the present paper, we show that the Cancitis model may be used in combination with a few initial measurements of the *JAK2*V617F allele burden to estimate both treatment duration and outcome for the benefit of the patients. In combination with unique data from a randomized trial, the mechanistic Cancitis model performs well. To our knowledge, such comprehensive data material of MPN patients with frequent serial measurements of cell counts and *JAK2*V617F allele burden over several years is rare. Earlier, the focus has typically been on population responses rather than individual trajectories that may show significant variations [26,27,28,29]. In addition, to our knowledge such data has not previously been explained by mechanism-based mathematical modelling capturing the data with high accuracy during treatment. This is the approach of the present paper. The clinical data shows that IFN therapy may be an efficient treatment for MPN patients; however, the Cancitis model identifies and quantifies the underlying mechanisms. Moreover, for the first time the therapy of MPN patients using IFN is described by a pharmacokinetic-pharmacodynamics (PK-PD) model. Finally, we demonstrate that, based on a few patient-specific measurements over time, the model can predict the subsequent personalized treatment response accurately.

## 2. Results

In this section, the data and model predictions are compared. The model predictions are obtained using the validated mechanism-based Cancitis model outlined in Section 4. The materials and methods have been previously described in greater detail [19,20,30].

**Figure 1:**
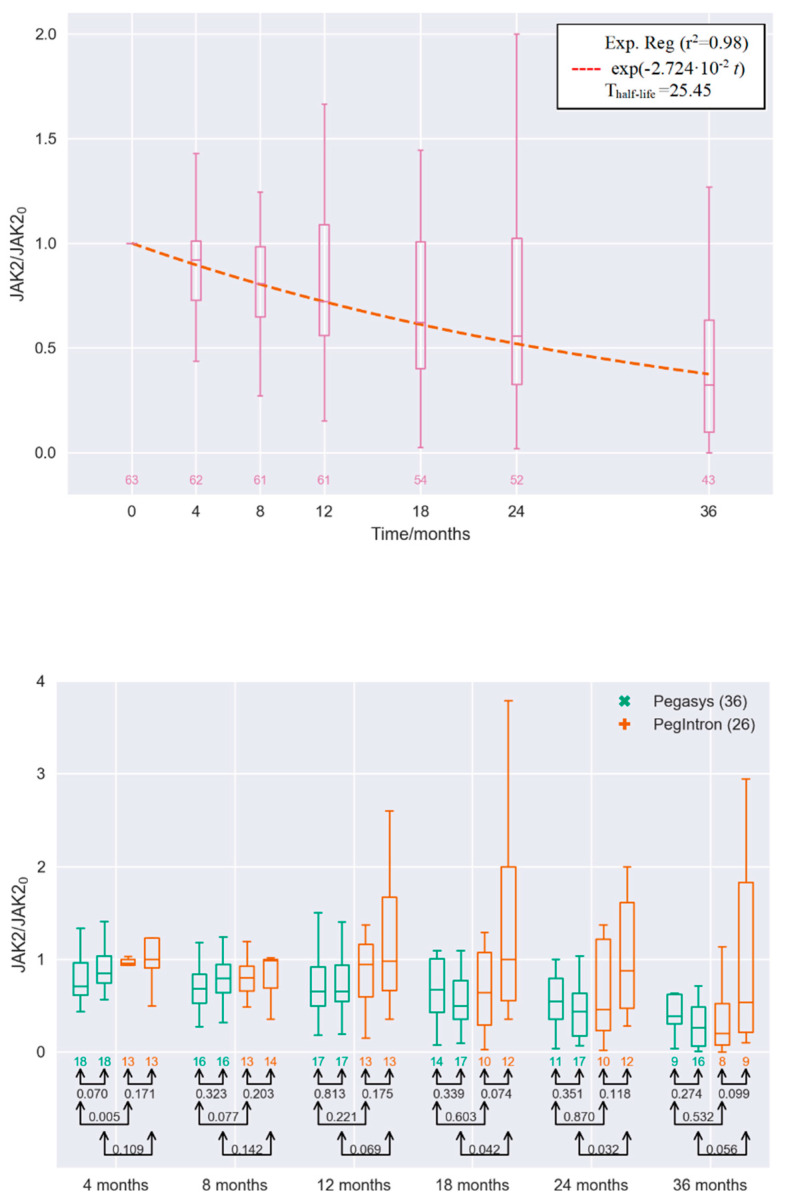
Top panel: *JAK2*V617F allele burden measurements for all the interferon-α2 (IFN)-treated myeloproliferative neoplasms (MPN) patients in the DALIAH trial normalized with their respective baseline value denoted *JAK2*_0_ at the secondary axis (see Section 4: Materials and Methods). Median values fitted with exponential decay reveal that the *JAK2*V617F allele burden is halved every 25 months on a population level. Bottom panel: Boxplot showing the medians and percentiles of the *JAK2*V617F allele burden measurements for the IFN-treated MPN patients in the DALIAH trial normalized with their respective baseline values (see Section 4: Materials and Methods). Boxplot values are depicted for each visit in the clinic—i.e., at months 4, 8, 12, 18, 24, and 36, where all have the normalized value 1 at baseline. For each visit, the leftmost is for the cohort receiving Pegasys and of an age below 60, whereas the next (the second from left) is for the cohort receiving Pegasys and of an age 60 or above (both groups in green). The next two are for the cohorts receiving PegIntron of an age below 60 (the third from the left) and the cohort receiving PegIntron of age 60 or above (the rightmost) (both groups in red). Only patients with more than three observations are included. Below each boxplot, the number of patients is shown, whereas connected pairs of arrows and the numbers in black represent the *p*-value in a Welch’s unequal variances *t*-test for the equality of means.

First, we investigated the separate effect of varying the parameters in the model, such as death rate, differentiation rate, self-renewal rate, etc. The far most sensitive parameter was the stem cell death rates. The death rate of the malignant stem cells was the single parameter which described the JAK2V617F allele burden data well. Figure 1 shows the medians and percentiles of the *JAK2*V617F response over time for IFN-treated patients from the DALIAH trial normalized with their respective baseline value. Thus, the effect of treatment will conservatively be compared to the baseline level. Due to drop-out from the study, the number of patients eligible for the analysis is decreasing over time. The longitudinal median values closely follow an exponential decay pattern, with a half-life of 25 months. Stratification by age and treatment dependence is also shown. Boxplots for each study visit in the protocol, scheduled at months 4, 8, 12, 18, 24, and 36, are shown in the figure. Similar investigation for patients staying in the trial until month 36 yields a half-life of 24 months. Likewise, the boxplots for each study visit for patients with ET, PV, and PMF receiving Pegasys or PegIntron and with an age below 60 or above 60 are shown. These figures show a statistically significant reduction in the *JAK2*V617F allele burden over time during treatment with IFN, despite great inter-variance, especially for patients treated with interferon-α-2b (PegIntron), except for those above 60 year of age receiving PegIntron. Untreated disease development cause exponential increase, which is dampened but not significantly reversed for those above 60 of age receiving PegIntron. The reduction in the *JAK2*V617F allele burden in response to interferon-α-2a (Pegasys) seems more pronounced than that of PegIntron (for the *p*-values per visit, see the figure).

**Figure 2:**
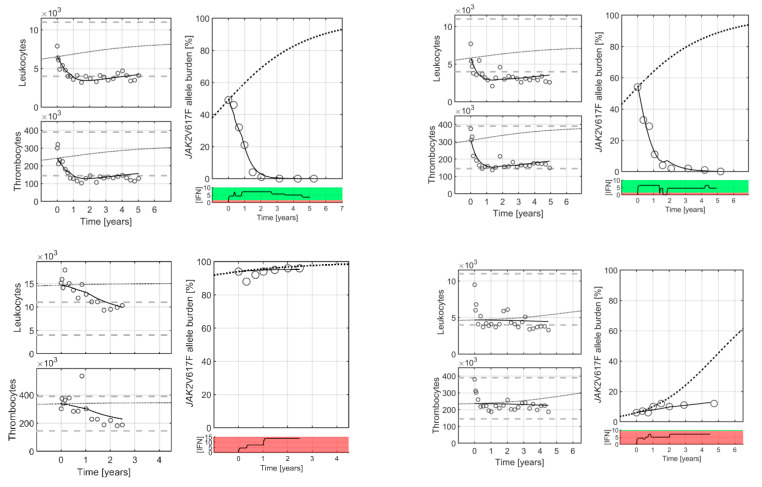
Four typical patients: two good responders (**top row**) and two poor responders (**bottom row**) treated with IFN. Good and poor responders refer to a decrease or increase in allele burden, respectively, regardless of the changes in cell counts. Circles are clinical measurement, whereas full curves are the Cancitis model trajectories. The stipulated curves are sigmoidal increasing “population” master curves based on the data from patients off treatment (see [12]). Death rate of the malignant cells is used to calibrate the Cancitis model to patient-specific data for the *JAK2*V617F allele burdens. Measurements of leukocyte and thrombocyte counts are used for validation. Grey horizontal lines indicate the upper and lower limit for the normal cell count ranges, respectively. Below the *JAK2*V617F allele burden, figure panels of the patient-specific daily average IFN dose over time are shown. From the Cancitis model, it follows that malignant cells are suppressed and decreasing when the dose is in the green region, and when dose is in the red region the suppression is insufficient. The regions are separated by a patient-specific threshold value. For the *JAK2*V617F, leukocyte, and thrombocyte data as well as the model calibration for all patients, see Supplementary Material C.

**Figure 3:**
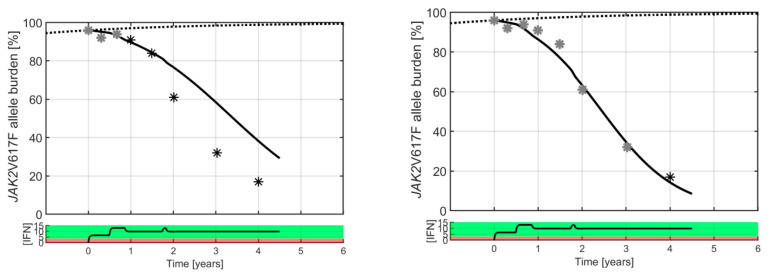
A typical good responder is shown with a high baseline value (50% *JAK2*V617F allele burden). Black and grey stars are clinical measurements, whereas the full curves are model predictions. The dotted curve is a sigmoidal increasing “population” master curve corresponding to unperturbed disease growth. Grey data points are used to calibrate the Cancitis model for personalized prediction, and the black data points are the clinical measurements used for validation. Generally, three data points for calibrating the model provide good predictions, but increasing the number of data points increases the accuracy of the predictions. A curve generated by a pharmacokinetic model displays the daily average IFN dose in µg in black in the bottom panel (see Supplementary Material A). The red dose–response region indicates the dosing value of poor individual response, and the green dose–response region indicates the dosing value of good individual response, hence the separation between the two represents the effect threshold for the particular patient shown. A catalogue of all model predictions based on the various numbers of visits to the clinic for each patient can be found in Supplementary Material B.

At the individual level, the Cancitis model is calibrated to clinical data, as illustrated in Figure 2. The Cancitis model describes individual patient trajectories—i.e., *JAK2*V617F as well as leukocyte and platelet counts over time—using the malignant stem cell death rate as a fitting parameter. The model can account for good responders (top row), defined as decreasing *JAK2*V617F response during treatment as well as poor responders (bottom row), where an increase in *JAK2*V617F response occurs during treatment. The response is patient-specific and dose-dependent, thus whether the actual dose is above or below an individual threshold value plays a crucial role. All the patient data and model calibrations are shown in the Supplementary Material. When calibrated to the *JAK2*V617F data, the model may be used as an individual predictive tool. Including the three first data points during treatment (months 0, 3, and 6) for calibration gives predictions for the remaining five-year period, as illustrated in Figure 3. Generally, the predictions are robust, since including additional data points for calibrating the model leads to only a minor increase in accuracy (see Supplementary Material).

**Figure 4:**
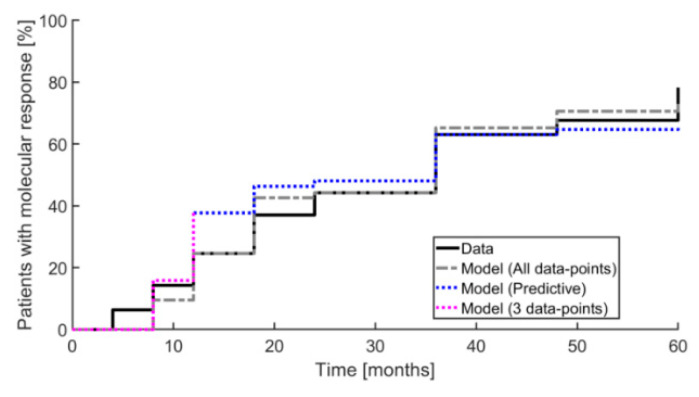
An inverse Kaplan–Meier plot is shown illustrating the fraction of patients obtaining a partial molecular response (50% reduction relative to baseline) during the 60 months of measurement. The full black curve shows data and the grey dashed curve shows model prediction using all data points for calibration. The dotted blue curve shows the model prediction, where for each step only the preceding data points were used. The dashed purple curve shows the predictions of the first three data points using these for calibration.

**Figure 5:**
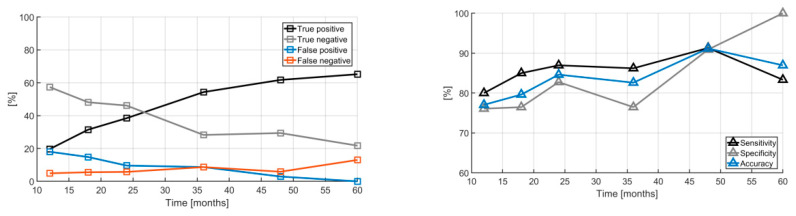
The “confusion matrix” of model trajectories showing whether the Cancitis model correctly classifies the patient as having a partial molecular response (left). The true positives increase as more data are obtained while the true negatives decrease. The sensitivity, specificity, and accuracy of the Cancitis model are illustrated in the right panel.

The Cancitis model also predicts the overall outcome of the effect of IFN treatment, as illustrated by the inverse Kaplan–Meier plot in Figure 4. This shows a 50% reduction relative to baseline for patients obtaining a partial molecular response (PMR) over the 60 months of measurements. The data are compared to the predictions based on the one-parameter (the death rate of the malignant cells) calibration of the Cancitis model. The calibration and predictions by the model may be further qualified using a quantitative “confusion matrix” consisting of the following entries: true positive (TP), true negative (TN), false positive (FP), and false negative (FN). These entities are defined as: (1) the fraction of model predictions of partial molecular response relative to data showing partial molecular response at the time of measurements, (2) the fraction of model predictions of partial molecular response relative to data not showing a partial molecular response at the time of measurements, (3) the fraction of model predictions of no partial molecular response relative to data showing no partial molecular response, and (4) the fraction of model predictions of no partial molecular response relative to data showing no partial molecular response at the time of measurements, respectively. From the results illustrated in Figure 5 we obtain the sensitivity, specificity, and accuracy as TP/(TP+FN), TN/(TN+FP), and (TP+TN)/(TP+TN+FP+FN), respectively.

The true positive PMR cases increase with time as more data are collected (from 20% to 63%) as the true negative cases decrease (from 59% to 21%). Likewise, the model-derived sensitivity, specificity, and accuracy increase over time as more data are included, see Figure 5 (right).

**Figure 6:**
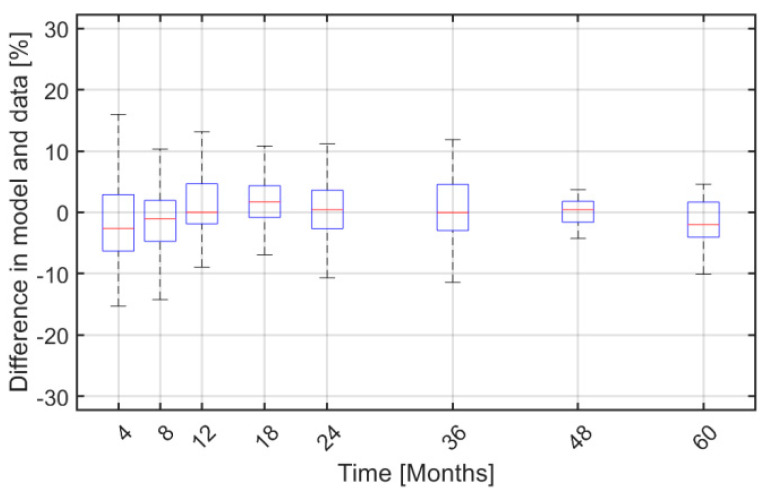
Boxplots of the absolute difference between the personalized *JAK2*V617F allele burden data and model predictions over time—i.e., at month 4, 8, 12, 18, 24, 36, 48, and 60 after baseline.

Finally, Figure 6 shows boxplots for the difference between *JAK2*V617F allele burden and model predictions as more data are included over time. Notice that the difference in medians between the measured and model predictions is negligible and the difference in the percentiles deviates less than 5%, while the medians of the model deviate less than 2.5% from the trial data.

**Figure 7:**
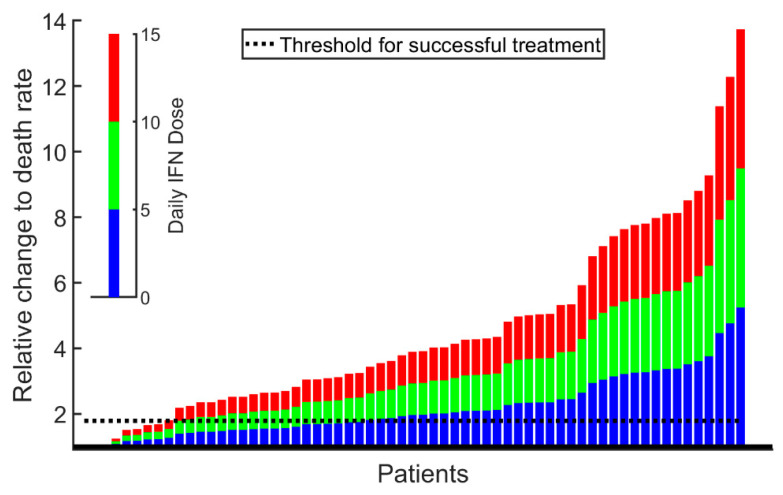
From the Cancitis model, it can be computed whether the specific patient dose–responses suffice to force the patient towards a healthy or a diseased state (i.e., the cancer is about to be eradicated or the cancer escapes with ensuing disease progression). Each patient prediction in the study is represented by one column. The blue part of the columns depicts if the patient received an average daily dose of 5 µg IFN, the green depicts if the patient received 10 µg IFN per day on average, and the red depicts if the patient received 15 µg IFN per day on average. If the top of the columns are above the black dashed line, corresponding to 1.8-fold increase in the malignant stem cell death rate, the patient is said to respond well to the treatment—i.e., the allele burden steadily declines and so do the leukocyte and thrombocyte counts. For an average daily dose of 15 µg, the model predicts that there are 3 non-responders (not visible) at the left most part of the figure, followed by 6 poor responders. The remaining 54 are good responders. A similar interpretation can be made for an average daily dose of, e.g., 5 or 10 mg IFN. A stratification of the figure into patients receiving Pegasys and PegIntron, respectively, resembles the merged figure but with lower number of patients.

The mechanism-based model is used for estimating the patient-specific threshold value for dosing, separating a good responder from a poor responder as shown in Figure 7. Adjusting multiple model treatment parameters revealed that it is sufficient solely to adjust the death rate of malignant stem cells.

**Figure 8:**
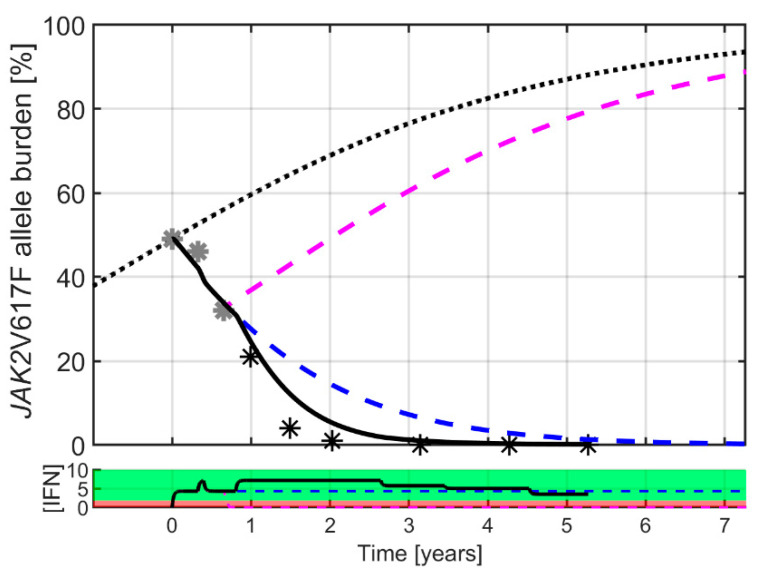
Different hypothetical modelling treatment scenarios: treatment discontinuation and prolonged treatment, respectively. Pausing the treatment after month 8 predicts a relapse of the cancer, and the pre-treatment *JAK2*V617F value is reached after 1.6 years (pink dashed curve). In contrast, prolonged treatment suppresses the cancer in the model (black solid curve) even with a lower dose (blue dashed curve). The model average daily IFN dose in µg is indicated in the lower panel, with line colors corresponding to those in the upper panel. Patient-specific model prediction for all patients exposed to hypothetical treatment discontinuation and prolongation, respectively, are depicted in Supplementary Material D.

Using the mathematical model to investigate different hypothetical treatment scenarios, such as treatment disruption and prolonged treatment, as illustrated in the two scenarios in Figure 8, provides additional results. Interrupting the treatment after month 8 shows a relapse of the cancer, and the pre-treatment *JAK2*V617F value is reached after 1.6 years. In contrast, prolonged treatment in the model even with a continued lower dose eliminates the cancer over time.

In the model, the number of malignant stem cell vanishes at year 12 for the patient shown in Figure 8. However, with a lower limit of detection of 0.01% the limit for detecting malignant cells is reached at year 7, while with a lower limit of detection of 0.1% the limit is reached around year 4. Thus, the model predicts the eradication of the cancer at year 12, while the number of malignant stem cells becomes undetectable between year 4 or 7 depending on the accuracy of measurement. In the Supplementary Material, all the model fits to patients are shown along with the predicted model outcome for the *JAK2*V617F allele burden if treatment is discontinued at any point of visit to the clinic. A prolonged treatment period implies a prolonged relapse time.

## 3. Discussion

The mechanism-based Cancitis model reproduces and predicts the *JAK2*V617F allele burden data better than the empirical model presented in [12], and in addition also predicts the leukocyte and platelet counts well. Furthermore, the Cancitis model suggests which mechanisms are responsible for treatment effect and quantifies these mechanisms. The use of mechanism-based mathematical modelling parametrized by longitudinal data has provided important insights into the disease mechanisms and effects of the treatment of chronic myeloid leukemia [31,32].

We emphasize that clinical practice which relies on comparing the recent data—e.g., the *JAK2*V617F allele burden and leukocyte and platelets counts with previous data—may be sensitive to many unknown factors, causing random fluctuations in data and thus making the comparison uncertain. In our approach, based on a time-series data analysis, the effects of noise are minimized for statistical reasons. Thus, the model gives good estimates for how long time a patient should expect to receive IFN treatment in order to decrease the *JAK2*V617F allele burden to a specific level, and the accuracy of those estimates increases when going from a prediction based on three observations to one based on four, five, or six. The clinicians could update their estimate at each visit to the clinic, thus improving the treatment strategy as well as informing the patients about the updated estimate. For the patient in Figure 8, the model predicts that 12 years of treatment is needed to eradicate the cancer. However, the lower limit of detection is reached between year 4 and 7 depending on the measuring accuracy. Discontinuing the treatment before year 12 would sooner or later result in a relapse. The model predicts the time of relapse to increase with an increasing treatment period. In health economy, such a prediction may also be of interest, since cost and gains could be anticipated [12]. Furthermore, the mechanism-based model can be used for estimating the patient-specific and dose-independent threshold value for dosing, separating a good responder from a poor responder—see Figure 7. In addition, having a mechanism-based model offers the possibility of mechanistic insights into the major effects of IFN—e.g., whether the increased proliferation or death rate of mainly malignant stem cells is sufficient to explain observations as suggested in [33,34] and [22,35]. Adjusting multiple treatment parameters—e.g., both the malignant stem cell death rate and differentiation rate—gives similar results to solely using the death rate.

The study shows that IFN normalizes blood cell levels and has a marked effect on the competition of wild-type and *JAK2*V617F mutated cells. This may suppress the malignant cells to go below the detection limit.

The mathematical modeling approach allows for the investigation of different hypothetical treatment scenarios—e.g., treatment disruption or prolonged treatment. These two scenarios are illustrated in Figure 8. Interrupting the treatment after month 8 shows a relapse of the cancer, and the pre-treatment *JAK2*V617F value is reached after 1.6 years. In contrast, prolonged treatment in the model even with a lower dose may eliminate the cancer over time. From Figure 1, Pegasys performs better than PegIntron. This may be due to differences in the pharmacokinetic and pharmacodynamic properties of the different formulations. Likewise, good responders generally appear to calibrate better to data than poor responders (see Supplementary Material for all fits and the corresponding sum-of-square errors normalized by the number of data points as a measure for the goodness of fits). Taking the median of the sum-of-square errors for *JAK2*V617F for the group of good responders versus that of the poor responders, we get 16.5 and 18.2, respectively, and the means become 29.7 and 65.2 with standard deviations of 38 and 56, respectively, due to a single outlier (P089). Thus, despite potential differences in the quality of the model fits, there are no statistically significant differences between the two groups. The observed difference in efficacy between PegIntron and Pegasys in favor of PegIntron is of interest. Several mechanisms might be operative. Differences in pharmacokinetics, drug dose, and toxicity profiles may account for the observed differences in the ability of the drugs to decrease the *JAK2*V617F allele burden. PegIntron is more likely to hydrolyze due to an unstable urethane bond between the pegylated portion and the recombinant IFN molecule compared to Pegasys, which has a more stable amide bond [36]. Therefore, Pegasys has a longer half-life and prolonged plasma concentration compared to PegIntron [37]. Whether this is associated with increased efficacy and less toxicity is not well described. In chronic myeloid leukemia (CML), comparative trials of both forms of interferons have not showed any difference in efficacy or safety profiles between the drugs [38]. In the DALIAH trial, treatment-related toxicity was higher among patients treated with PegIntron compared with Pegasys after 36 months of treatment. Hence, the patients randomized to PegIntron received lower doses compared to Pegasys, which may have resulted in a lower molecular efficacy.

The unique longitudinal data provided by the randomized DALIAH data are necessary for calibrating reliable mathematical models in relation to blood cancers. In turn, mathematical models (1) provide the means to test whether the IFN treatment mainly affects the malignant stem cell death rate; (2) offer “a microscope” for obtaining continuous measurements over time for the stem cell population, which is currently experimentally inaccessible from mature blood cell counts; (3) offer an efficient methodology for connecting data points over time and predicting the further time course; (4) deliver estimates of patient-specific threshold values for dosing and timing; (5) serve as an additional expert decision tool in the clinic for predicting the treatment outcome; and (6) provide estimates for the duration of the treatment period and dose to obtain a desired molecular residual disease and the relapse time that may follow.

Our results are more comprehensive than the previously presented results in [17,18], mainly due to the high-quality data over five years, which allow for more and better analyses. Thus, the complex relation between data and mathematical modeling calls for a close collaboration between clinicians and mathematicians in order to increase the scientific insights into the *JAK2*V617F kinetics and response to treatment.

It is a limiting factor of our study that the effect of IFN in the Cancitis model is implemented through the increased death rate of malignant stem cells. However, including stem cell differentiation rate also as being affected by the IFN treatment provided similar results, except for small humps in the *JAK2*V617F allele burden and to a minor degree in the leukocyte and platelet counts whenever the treatment dose is abruptly changed. In the present case, no other single parameter is able of calibrating the model to the data, thus the malignant stem cell death rate is the single dominating factor. IFN may affect HSC by the loss of quiescence, leading to exhaustion in the long run and hence the loss of self-renewal potential. Neglecting the IFN effect on quiescence in the model may lead to an overestimate of the actual estimated values of the malignant stem cell death rate.

Furthermore, in the Cancitis model only single malignant clones (here the *JAK2*V617F) are considered, ignoring the possibility of additional mutations, resistance, and comorbidities, which may be important especially in the long time horizon. The flexible framework of mathematical modelling allows for extensions of the Cancitis model to include additional mutations and drug resistance, which will be addressed in future work. Such extensions of the model are in principle straight forward, but the challenging part is that these extensions introduce additional parameters which have to be estimated, and therefore additional measurements are needed to support that. It is another limitation of the study that only side effects related to the *JAK2*V617F allele burden or leukocyte and thrombocyte counts are considered—i.e., we only discuss normal and elevated levels of these in our modeling attempt and do not include indirect or derived consequences, nor do we include other possible side effects.

## 4. Materials and Methods

### 4.1. Clinical Study Design

Data were obtained from the DALIAH trial (#EudraCT 2011-001919-31), as described in [12] and encompassing 113 patients being treated with IFN. DALIAH is an ongoing Danish multicenter prospective randomized open label phase III clinical trial comparing IFN (Pegasys and PegIntron) with hydroxyurea. The treatment dose was modified during the study based on the drug efficacy and toxicity according to pre-defined dosing guidelines. The interruption of IFN due to adverse events was allowed for a maximum of 6 months.

### 4.2. Mathematical Study Design

#### 4.2.1. The Cancitis Model

The Cancitis model has previously been published in [19], and an extended version refining the adaptive immune component related to the cytotoxic T cells is published in [20]. The model uses a mechanistic approach, where hematopoietic stem cells (HSC) and mature cells (HMC) as well as malignant stem cells (LSC) and their mature offspring (LMC) are included explicitly, while the progenitor cells are included implicitly. Moreover, cell debris is accounted for, since this is recycled and cleared from the environment by the upregulated immune system. The description of the immune system is lumped into one variable representing the immune activity; however, the specific cytokines representative of the MPNs correlate with this inflammatory activity. The malignant cells may activate helper CD4+ T cells and cytotoxic CD8+ T cells fighting the cancer. The change in variables over time is described by a system of non-linear ordinary differential equations, which is based on conservation laws. The equations are summarized in the Supplementary Material. The model is calibrated to known cell counts from the literature, the typical time constant for developing MPNs, and is validated against independent cytokine data from the literature for non-treated subjects; for further details, see [19,20]. From the mature blood cell counts predicted by the model, the leukocyte and thrombocyte counts are calculated, assuming that they constitute a certain but patient-specific fraction of the mature cells over the time span considered in the study. Throughout the paper, the term “mechanism-based model” is used whenever the problem addressed is described and explained at a lower taxonomic level, as is the standard in the mathematical modeling society.

In the context of IFN treatment, certain PD parameters need adjustment, as described in the next section. Here, we briefly address how IFN PK is added to the model. IFN is generally administrated weekly by self-injection subcutaneously over a period of years. Thus, we ignore the variation in IFN concentration in blood on a weekly scale and only include changes in doses according to the patient journals. Inspired by [39], we adopt the simplest possible PK model, which is a single compartment absorption and release compartment. It takes approximately 8 weeks to reach the maximum level with a constant dose [39]:

(1) $$\frac{dc}{dt}=\frac{c_{dose}-c}{\tau },$$

where $c_{dose}$ is the week-average dose given (as known from data as a function of time) and c is the concentration in the blood at the effect site (at time t), while τ is the uptake and elimination time constant, which are approximately equal for IFN [39]. The PD effect is $1+\rho_{i}c$ times the default untreated parameter value of the LSC death rate, where $\rho_{i}$ is a scalar defining the PD effect of the individual response for patient i. Blood cell counts are, in the model, represented as fractions of the total blood cell count. These fractions are taken as constant for each patient during the study, but are allowed to vary inter-individually. Thus, leukocytes and thrombocytes are calibrated using one parameter for each based on the model prediction of the total blood cell count and the corresponding data. For further details, see the Supplementary Material.

#### 4.2.2. Mathematical Analysis Design

The IFN therapy affects the death rate of stem cells but also the differentiation rate. In addition, IFN may activate dormant stem cells and put them in cycle [24] and may relax the stem cell quiescence-enforcing mechanisms [25]. Despite some indications, IFN is generally not associated with an impact on stem cell self-renewal [24]. However, IFN preferentially affects malignant cells [22,23]. It is a general principle to keep models as simple as possible as long as important elements are included in the model. In the present context, a minimum of the IFN effects should be considered. Hence, we start by including the most important mechanisms; if this does the job, then including further mechanisms would work. If not, one should include the second most important mechanism, etc. Using such a design, we solely need to include effects of IFN on the death rate of the malignant stem cells, and the remaining effects are assumed to be minor for the present study. In the model, the disease state is forced toward a parameter-dependent healthy steady state, a co-existing steady state, a full blow cancerous steady state, or extinction, hereby confirming the three Es of immune-editing [11]. Thus, changing the model parameters may change the system dynamics, such that the system is forced toward another steady state—e.g., increasing the death rate of the malignant stem cells in the model may reverse the disease progression from toward severe cancer to toward a healthy state, and the cancer has been eradicated in the model. Such behavior is compared to the individual patient data and does not only verify the Cancitis model but also gives threshold values for the parameters, switching the dynamics from going toward cancer development into going toward a healthy state. We are particularly focusing on the patient-specific malignant stem cell death rate, which may be translated into the IFN dose. Thus, the patient-specific threshold values for the effectiveness of the IFN doses are obtained, dividing the treatment into patient-specific good and poor dose–response regimes.

The Cancitis model described in Section 4.2.1 is first calibrated such that the growth of the *JAK2*V617F allele burden of the model agreed with the growth found in [12] for the initial exponential disease development from 0 to 50%. Patients having less than four longitudinal observations are excluded, reducing the number of patients available for analysis from 113 to 63. During treatment with IFN, the model is further calibrated to data, as described in Section 4.1. The death rate of malignant stem cells was determined with MATLAB R2018b by minimizing the sum of squared error using the *fminsearch* function. All the data points during treatment are used to calibrate the treatment effects, and secondly we solely use the first three data points (at time 0, 4, and 8 month after diagnosis) for the calibration of the treatment effects; based on this, we estimate the development to follow and compare this development with the actual data for *JAK2*V617F. Information about whether PMR appears in the patient-specific data are compared to the model predictions. A partial molecular response is defined according to the 2013 ELN and IWG-MRT criteria [40,41], but neglecting the baseline level requirement of being above 20%. Even if PMR is not observed, the cancer development may be weakened—i.e., cancer progression may be slowed down compared to the otherwise untreated patient. Furthermore, we investigate the outcome of the disruption of treatment and prolonged treatment in the model. By analyzing the model, one may determine the threshold separating good responders and poor responders, which hopefully may be translated to clinical practice.

## 5. Conclusions

The Cancitis model has been validated further on a cohort of *JAK2*V617F-positive MPN patients from the randomized DALIAH trial, and the model captures MPN patients treated with IFN very well. We demonstrate that the Cancitis model provides novel scientific insights into the underlying hematopoietic dynamics with and without treatment. The Cancitis model captures the pronounced effect of IFN treatment on the *JAK2*V617F allele burden, as well as on elevated leukocyte and thrombocyte counts. In relation to this, we note the true positive—i.e., the model’s ability to predict the PMR - increase over time. A (inverse) survival plot illustrates the PMR to IFN, which strongly supports the superiority of the Cancitis model to predict the treatment outcome on the population level.

The Cancitis model has proven to be potentially helpful in the clinical setting, as it may predict how long treatment has to be continued for good responders. It is important to have as short IFN treatment periods as possible while suppressing the disease or sufficiently limiting the disease burden due to side effects related to IFN therapy. This will not only reduce the discomfort for the patients but also will be more cost-effective. The model may be used to plan how long time a good responder needs treatment to obtain a complete molecular response. It is at least equally important to obtain information as early as possible on whether patients respond favorably or poorly to the IFN therapy both for economical and medical reasons, including the quality of life for patients.

## Supplementary Materials

The following are available online at https://www.mdpi.com/2072-6694/12/8/2119/s1: Supplementary Material A: Mathematical methodology. Supplementary Material B: Fits to *JAK**2*V617F data. Supplementary Material C: Fits to leukocyte and thrombocyte data. Supplementary Material D: Predictive plots of JAK2V617F. Further Supplementary Material is available online at http://dirac.ruc.dk/cancitis, where a stand-alone GUI is available for examine alternative treatment plans for the patients in the study.
