# Supplementary material for: Mathematical Modeling of MPNs Offers Understanding and Decision Support for Personalized Treatment
Source: Cancers (Basel). 2020 Jul 30;12(8):2119. doi: 10.3390/cancers12082119 (PMC7466162; doi:10.3390/cancers12082119)

Supplementary material A: Mathematical methodology

Model formulation

The *Cancitis model* consists of the differential equations:

$$\dot{x_0} = (r_x\phi_x(x_0, y_0)s - d_{x_0} - a_x)\, x_0 \tag{1a}$$
$$\dot{x_1} = a_x A_x x_0 - d_{x_1} x_1 \tag{1b}$$
$$\dot{y_0} = \left(r_y\phi_y(x_0, y_0)s - \hat{d}_{y_0} - \tilde{d}_{y_0} y_0 - a_y\right) y_0 \tag{1c}$$
$$\dot{y_1} = a_y A_y y_0 - d_{y_1} y_1 \tag{1d}$$
$$\dot{a} = d_{x_0}x_0 + d_{x_1}x_1 + \left(\hat{d}_{y_0} + \tilde{d}_{y_0}y_0\right) y_0 + d_{y_1}y_1 - e_a a s \tag{1e}$$
$$\dot{s} = r_s a - e_s s + I \tag{1f}$$

with

$$\phi_x(x_0, y_0) = \frac{1}{1 + c_{xx}x_0 + c_{xy}y_0} \tag{2}$$
$$\phi_y(x_0, y_0) = \frac{1}{1 + c_{yx}x_0 + c_{yy}y_0} \tag{3}$$

where x_0 denotes the healthy stem- and progenitor-cells, y_0 denotes the leukemic stem- and progenitor-cells, x_1 the healthy mature cells, y_1 the leukemic mature cells, a the debris of dead cells and s is an abstract notion of the immune system load. The default parameters values used are shown in table 1.

r_x	$8.7 \cdot 10^{-4}$	r_y	$15 \cdot 10^{-4}$	a_x	$1.1 \cdot 10^{-5}$	a_y	$0.52 \cdot 10^{-5}$
d_{x_0}	$2 \cdot 10^{-3}$	d_{y_0}	$2 \cdot 10^{-3}$	$\tilde{d}_{y_0}$	$2 \cdot 10^{-9}$	$\hat{d}_{y_0}$	$2 \cdot 10^{-3}$
A_x	$4.7 \cdot 10^{9}$	A_y	$4.7 \cdot 10^{9}$	d_{x_1}	$1.29 \cdot 10^{-2}$	d_{y_1}	$1.29 \cdot 10^{-2}$
c_{xx}	$5.6 \cdot 10^{-5}$	c_{xy}	$5.4 \cdot 10^{-5}$	c_{yx}	$5.2 \cdot 10^{-5}$	c_{yy}	$5.0 \cdot 10^{-5}$
e_a	$2 \cdot 10^{5}$	e_s	2	r_s	$3 \cdot 10^{-4}$	I	7

Table 1: Default values for the parameters of the Cancitis model.

A reduced model is proposed and analysed by Ottesen et al. (2019). The model reduction is based on quasi-steady-state approximations of $\dot{x_1}$, $\dot{y_1}$, $\dot{a}$ and $\dot{s}$. The quasi-steady-state approximations imply the following algebraic expression:

$$x_1 = \frac{a_x A_x}{d_{x_1}} x_0 \tag{4a}$$
$$y_1 = \frac{a_y A_y}{d_{y_1}} y_0 \tag{4b}$$
$$a = \frac{1}{2}\sqrt{\left(\frac{I}{r_s}\right)^2 + 4\frac{e_s\kappa}{e_a r_s}} - \frac{I}{2r_s} \tag{4c}$$
$$s = \frac{r_s}{e_s}a + \frac{I}{e_S} \tag{4d}$$

where $\kappa = d_{x_0}x_0 + d_{x_1}x_1 + \left(\hat{d}_{y_0} + \tilde{d}_{y_0}\right) y_0 + d_{y_1}y_1$. The two-dimensional system is then simply:

$$\dot{x_0} = (r_x\phi_x(x_0, y_0)s - d_{x_0} - a_x)\, x_0 \tag{5a}$$
$$\dot{y_0} = \left(r_y\phi_y(x_0, y_0)s - \hat{d}_{y_0} - \tilde{d}_{y_0} y_0 - a_y\right) y_0 \tag{5b}$$

Cell-counts are interpreted from the model as:

$$\text{Leukocytes} = K_{leukocytes}(x_1 + y_1) \tag{6a}$$
$$\text{Thrombocytes} = K_{thrombocytes}(x_1 + y_1) \tag{6b}$$

Dose-dependent perturbation of model parameters

Some differences in dosing and timing of IFN treatment occurred in the DALIAH trial. Dosing information was approximated as the average daily dose, but to have gradual changes in IFN-perturbed parameters, we made use of a simple pharmakokinetic model of IFN. Absorption and clearance of IFN was modelled to occur with the same rate, τ, independently of the current dose. The IFN blood-concentration can hence be described by:

$$\dot{B} = \frac{1}{\tau}\left(D(t) - B(t)\right) \tag{7}$$

where $D(t)$ is the dose at time t and $B(t)$ is the blood concentration at the given time. Note that $D(t)$ is a stepsize constant function describing the average daily dose. Considering an interval $t \in [t_0, t_1]$ where $D(t)$ is constant, $D(t) = D_0$, the differential equation (7) has solution:

$$B(t) = D_0 - \left(D_0 - B(t_0)\right) e^{-t/\tau} \tag{8}$$

for $t \in [t_0, t_1]$. This allowed for a very simple estimate of the blood concentration level with smooth transitions from one level of treatment to another.

The parameter $\hat{d}_{y_0}$ was modelled to increase with the current blood-concentration of IFN. This was done by defining a function $\overline{\hat{d}_{y_0}}(\rho, t)$ such that:

$$\overline{\hat{d}_{y_0}}(\rho, t) = \left(1 + \rho B(t)\right) \hat{d}_{y_0} \tag{9}$$

where $B(t)$ is the time-dependent blood-level concentration of IFN and ρ is a parameter describing the strength of the patient-specific response to IFN treatment. Hence, a large ρ corresponds to a significant increase to $\hat{d}_{y_0}$ during treatment, while a low ρ signifies a low response. To ensure positive parameters, only positive values of ρ were considered.

Fitting procedure

The *JAK2*V617F allele burden was interpreted in the model as $C(t) = \frac{y_1(t)}{x_1(t)+y_1(t)}$, i.e. as the contribution of leukemic mature cells to the sum of mature cells. Hence, solutions of equations (5) together with calculation of x_1 and y_1 from equations (4) can be used to determine a model estimate of the *JAK2*V617F allele burden.

The model was simulated numerically in `MATLAB` using the function `ode15s`. One healthy stem cell, x_0 was removed and a leukemic stem cell y_0 was added at time 0. Due to the default parameters used, the system approaches a leukemic steady-state asymptotically. The *JAK2*V617F allele burden is initially close to 0 and approaches 1 as time increases.

The development of MPN before diagnosis was assumed to be equal for all patients and hence that the untreated disease development to be the same for the population. The initial baseline measurement of the *JAK2*V617F allele burden at diagnosis was used to determine where on the common disease development curve the patient were before treatment initiation. Hence data and model were shifted in time such that baseline *JAK2*V617F coincides with the model. Simulations of treatment-response used the model variables at the corresponding time as initial conditions.

The model was simulated for each patient over the time-period where data for the given patient was available. By separately modelling the blood-concentration of IFN as described above, and simulating a dose-dependent perturbation of $\hat{d}_{y_0}$ as shown in equation (9), the model could be simulated for different values of ρ, yielding different responses and developments of the *JAK2*V617F for the simulated time-period.

Model error was determined by the difference between the measurements of the *JAK2*V617F allele burden and the model estimate at the same time-points. Note that the baseline our methodology forces the model to be in agreement with baseline measurement. The value of ρ found to (locally) minimize the sum of squared errors was found using the `MATLAB` function `fminsearch`.

Cell-count measurements

The sum of mature cells in the model was assumed to correlate with both the cell-counts of thrombocytes and of leukocytes, see equations (6). As fluctuations between cell-lines were expected, and indeed observed in data, a complete correlation with both cell-counts cannot be expected for all measurements.

By assuming a linear scaling, we were able to determine two scaling-parameters, $K_{leukocytes}$ and $K_{thrombocytes}$, that minimized the sum of squares difference between cell-count data and the sum of $x_1(t)$ and $y_1(tx)$ the *JAK2*V617F-error minimizing fits described above. These scaling parameters then describe the proportion of mature cells in the model which can be attributed to the given cell-line; thrombocytes or leukocytes.

As some significant differences were observed between thrombocytes and leukocytes (e.g. one patient had increasing thrombocyte-counts but decreasing leukocytes counts over the study period), such linear scaling of

cell counts provides a rough estimate of the general level of mature blood-cells, and cannot be expected to be in perfect agreement with data. However, for a subset of patients, these ad-hoc blood-data fits were found to agree well with both *JAK2*V617F data and blood-cell data simultaneously even though the model was only optimised for agreement with *JAK2*V617F data, see supplementary material C.

In summary, a single parameter, $\hat{d}_{y_0}$ was modelled to increase linearly for increasing IFN-dose. An error-minimizing fitting procedure of the *JAK2*V617F allele burden identified a patient-specific response-parameter describing the strength of the perturbation to $\hat{d}_{y_0}$. A subsequent fit of the resulting model estimate of mature blood-cells counts to the thrombocyte and leukocyte counts found excellent agreement between the response to IFN in data and in the model for most patients. Hence a mechanistic interpretation as IFN resulting in an dose-dependent increase of malignant stem-cell clearing ($\hat{d}_{y_0}$) appears as (part of) a plausible explanation for the treatment-responses observed in patient data.

References

Ottesen, J. T., Pedersen, R. K., Sajid, Z., Gudmand-Hoeyer, J., Bangsgaard, K. O., Skov, V., Kjær, L., Knudsen, T. A., Pallisgaard, N., Hasselbalch, H. C., and Andersen, M. (2019). Bridging blood cancers and inflammation: The reduced Cancitis model. *Journal of Theoretical Biology*, 465:90–108.

Supplementary material B: Fits to *JAK2*V617F data

Below we show fits of the Cancitis model to all available data for the *JAK2*V617F allele burden. In the fits, the PD-effect of the IFN was modelled to increase the LSC death rate, dependent on the current blood concentration of IFN, modelled by a simple PK model as described in the main text.

The top panel shows patient-data and the model disease burden. The dotted black curves shows disease development, assumed to be the same for all patients. Baseline *JAK2*V617F is shifted in time to coincide with this curve. Grey $*$ signify data-points used for the fitting procedure, while black $*$ signify later data-points not used for the procedure, shown only for comparison and evaluation of the model fit. The full black line shows the best fit to the given data, defined as the fit that minimizes the sum of squared distance between the used data-points and the model at the given time-points, see the supplementary describing the mathematical methods. For time after the last data-points used for fitting, the actual future dosing information was used.

The bottom panel shows the PK-modelling of the blood-concentration of IFN as a black curve. The background color signifies the long-term state that the system will move toward: For doses where the background is green, the model implies that long-term the healthy state will be approached, while the red background shows doses that will approach full-blown disease. The unit of the dose is the average daily dose of IFN in μg. No distinction was made between *Peginterferon-α-2a (Pegasys)* and *Peginterferonα-2b (PegIntron)*

At the top of all figures, a goodness-of-fit-metric of the best fit is shown, defined as the sum of squared errors, divided by the number of points used in the fit. A low number signifies a good fit. Only the error of points used (the grey points) were included in the error estimate.

Patient P002

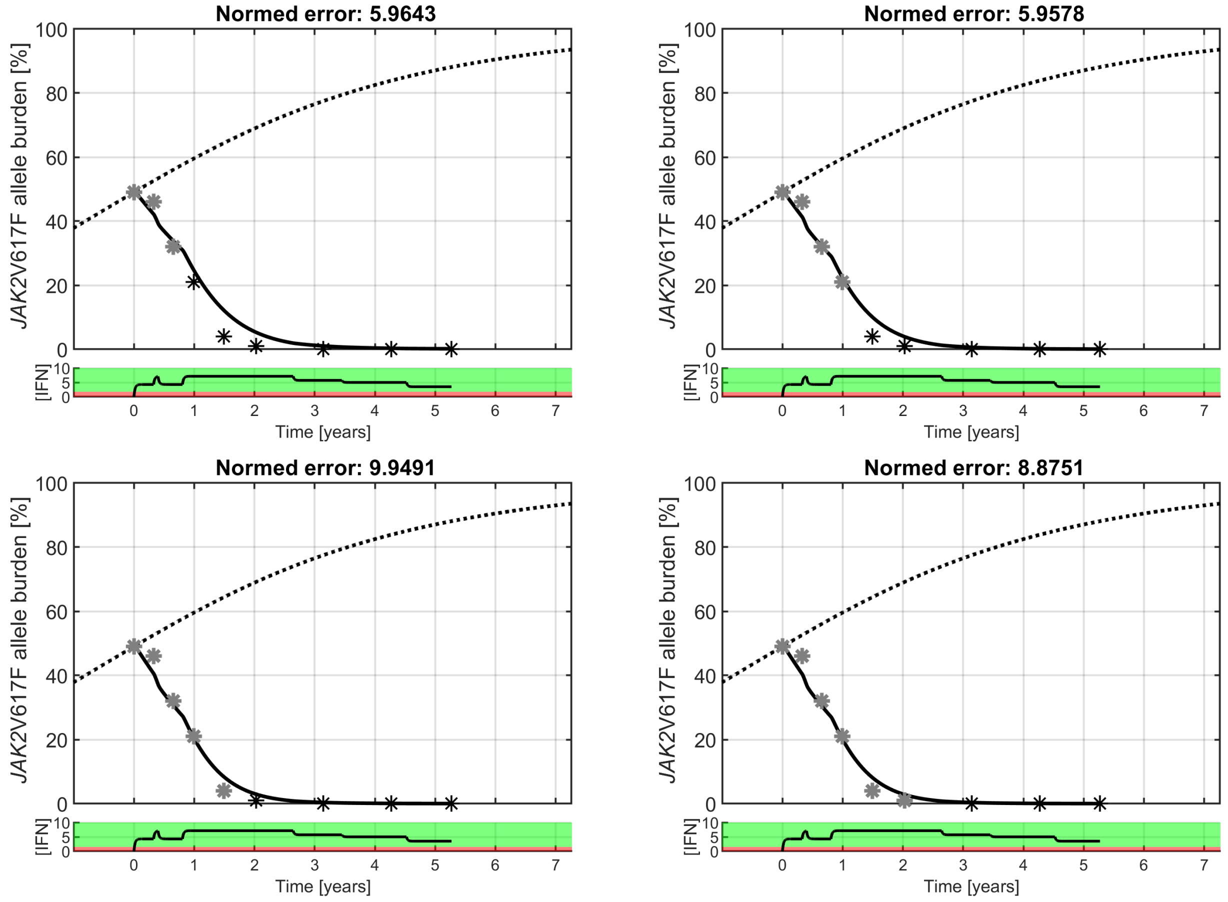

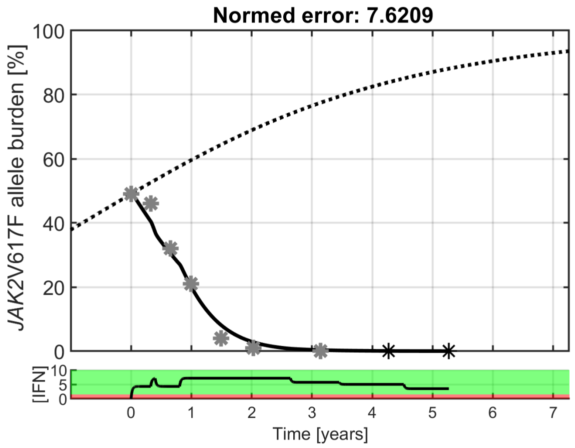

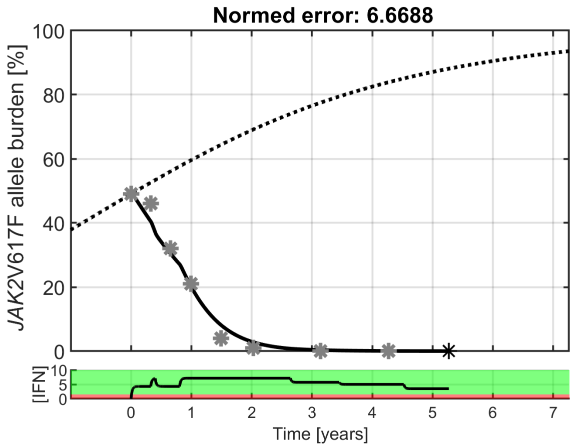

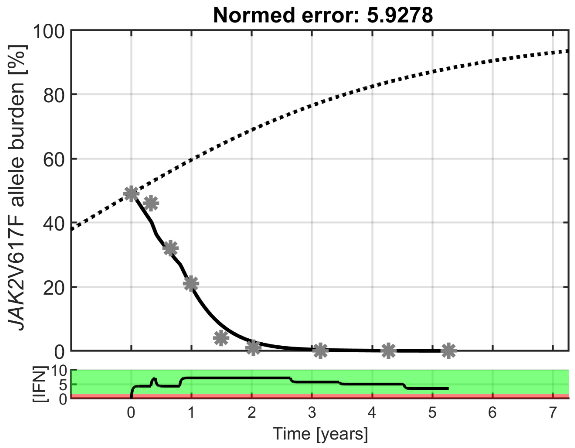

Patient P003

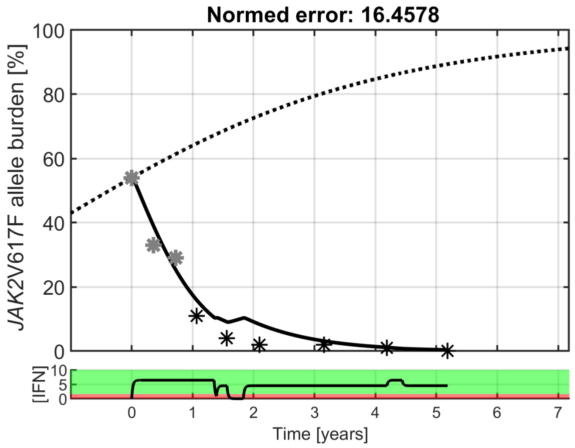

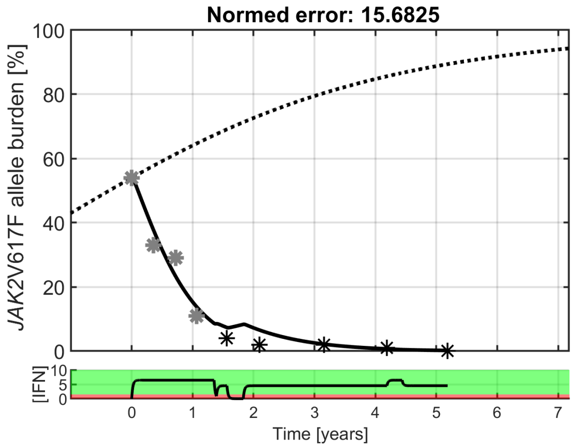

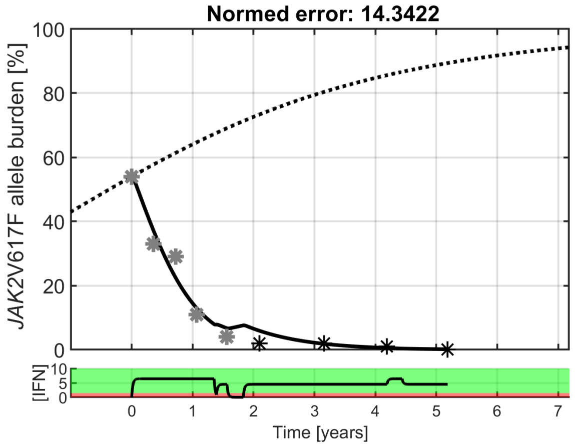

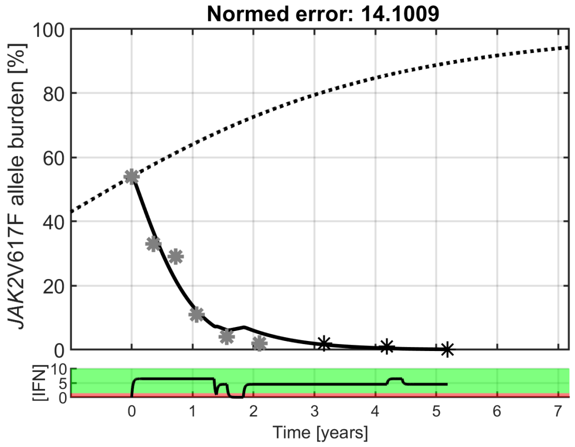

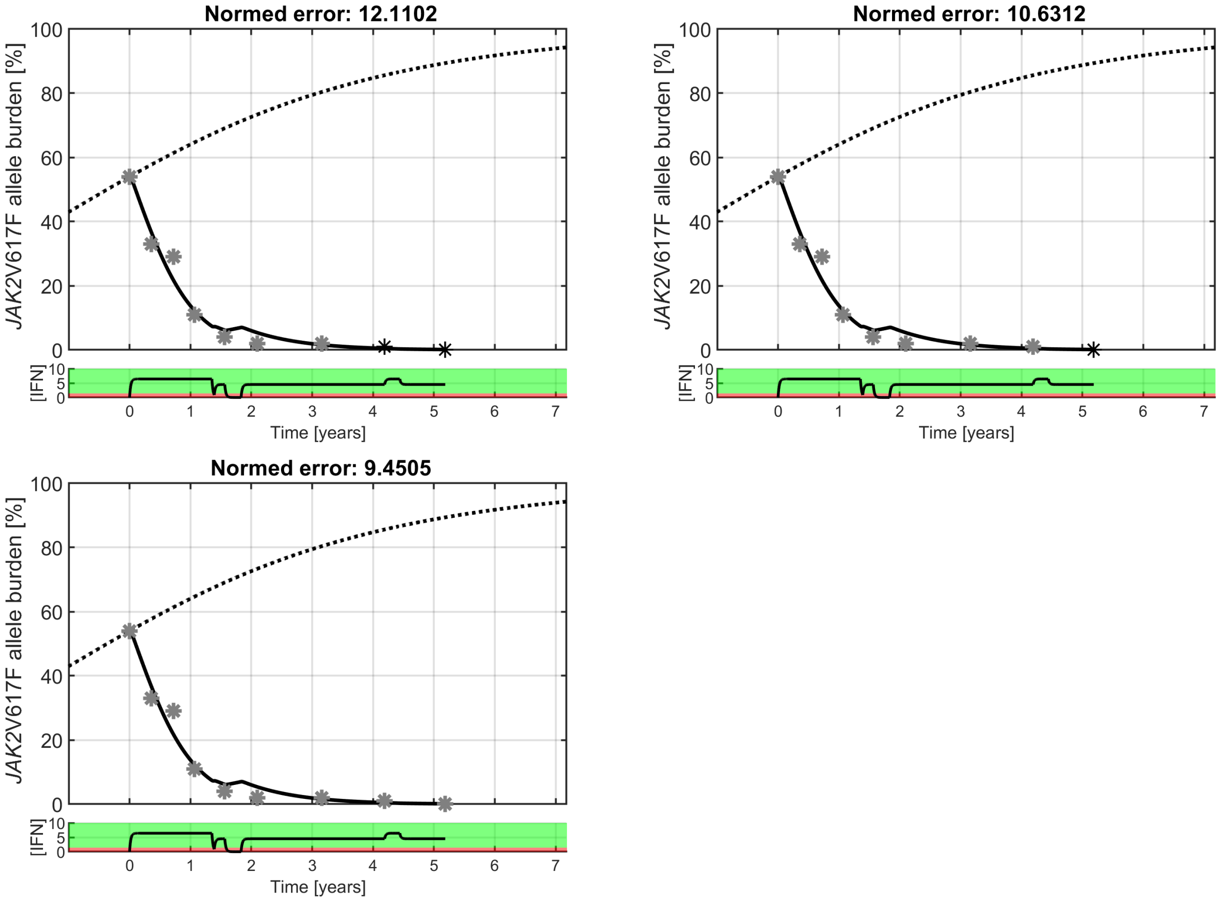

Patient P004

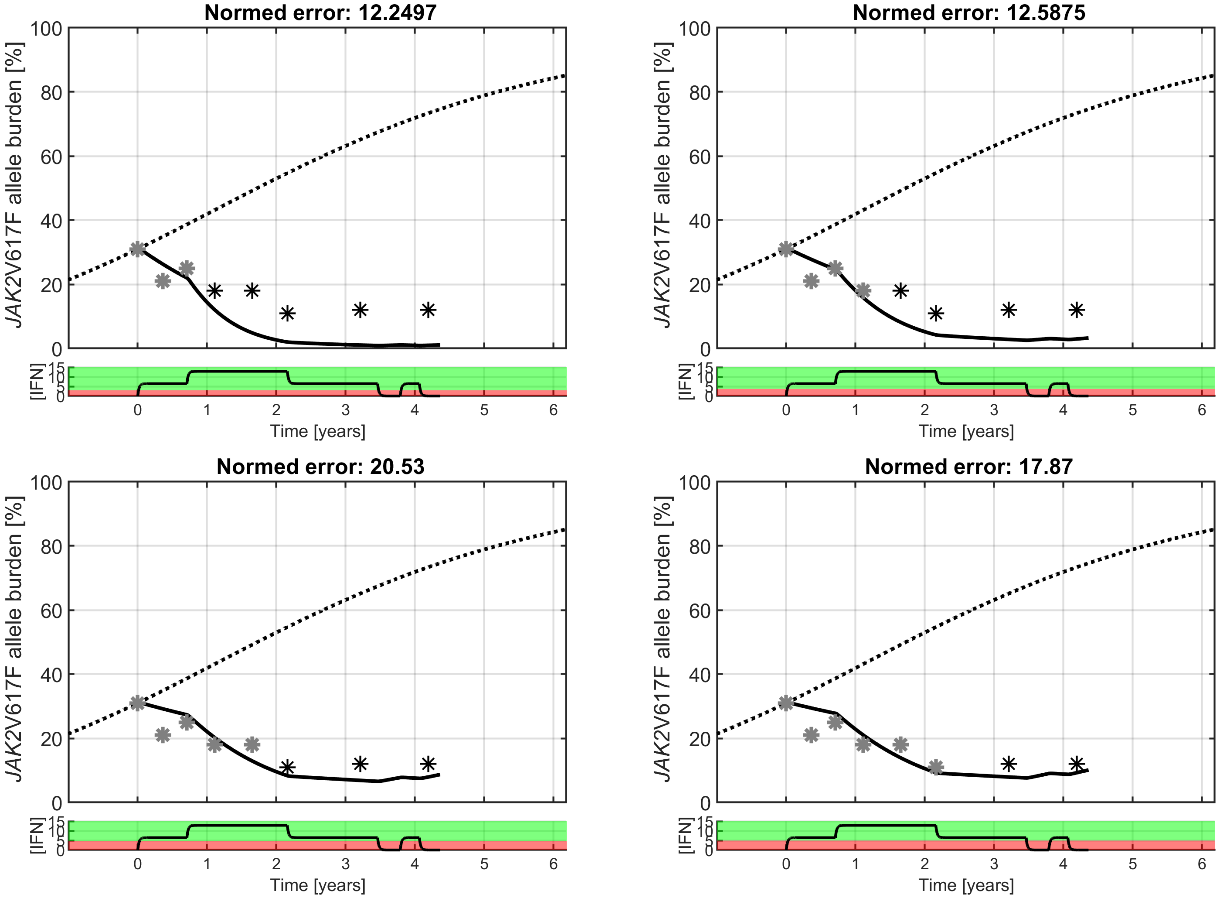

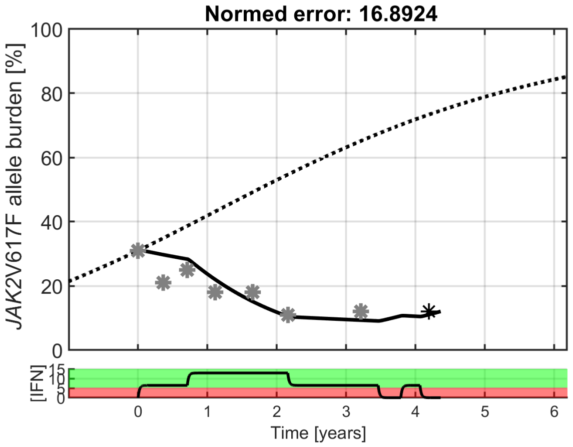

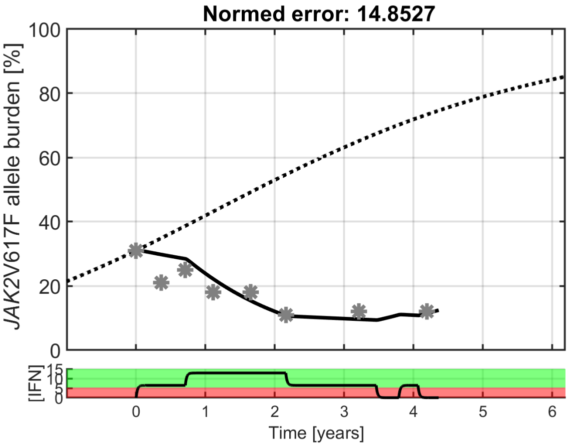

Patient P006

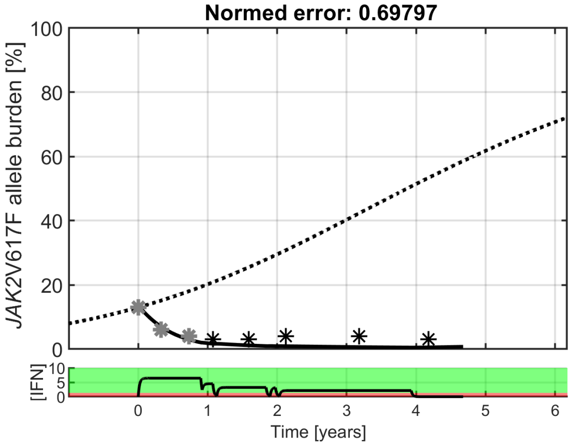

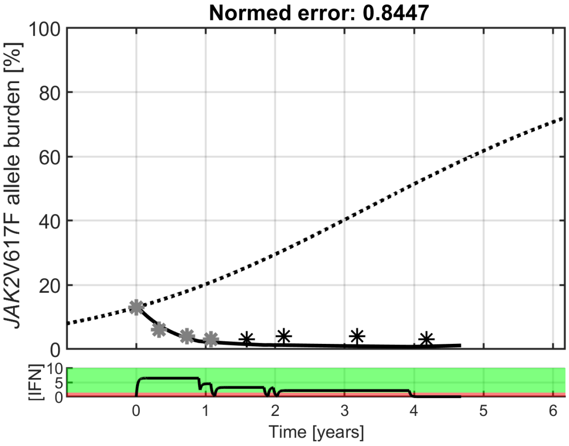

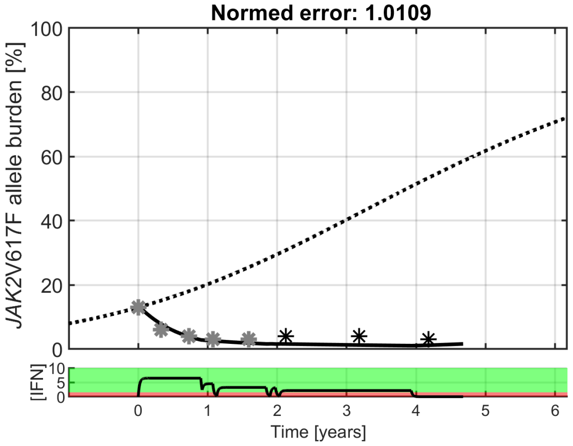

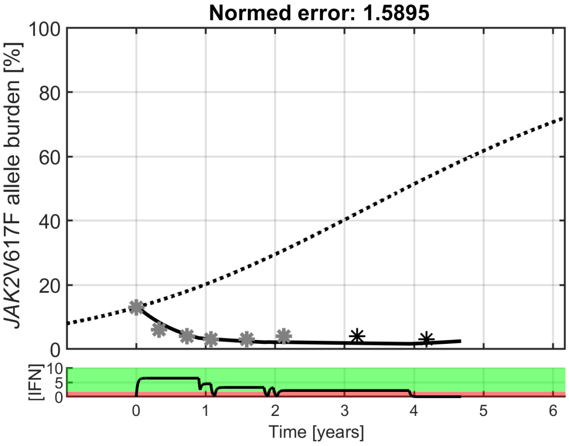

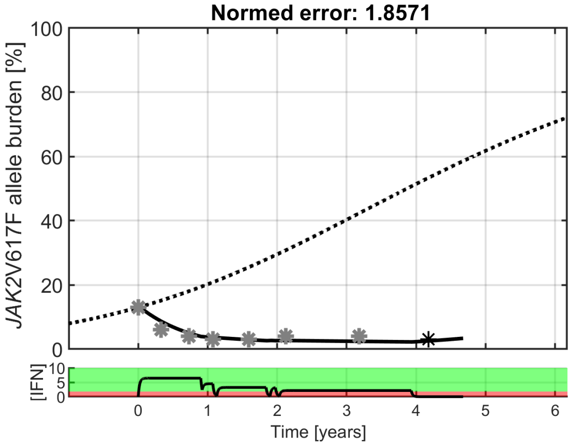

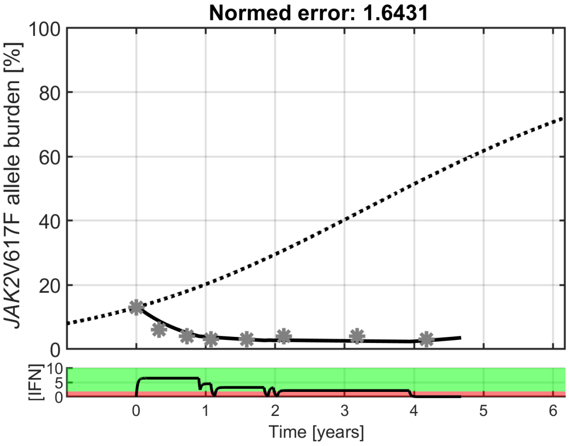

Patient P011

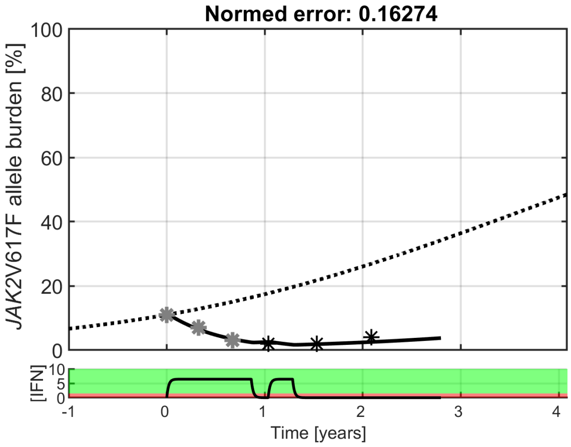

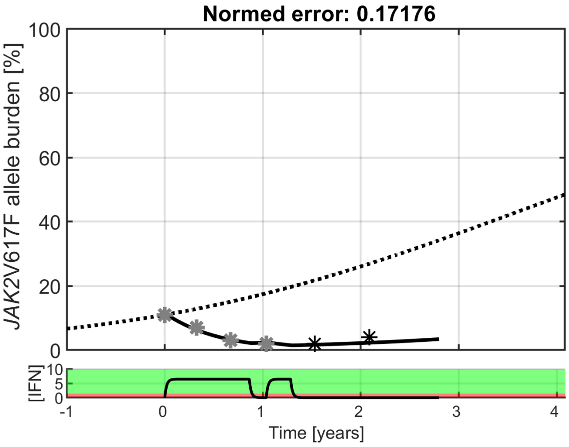

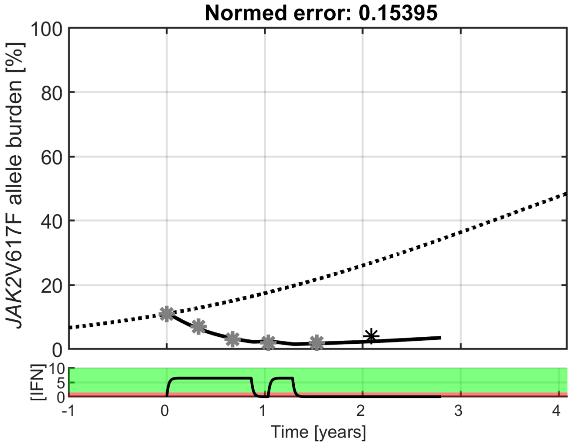

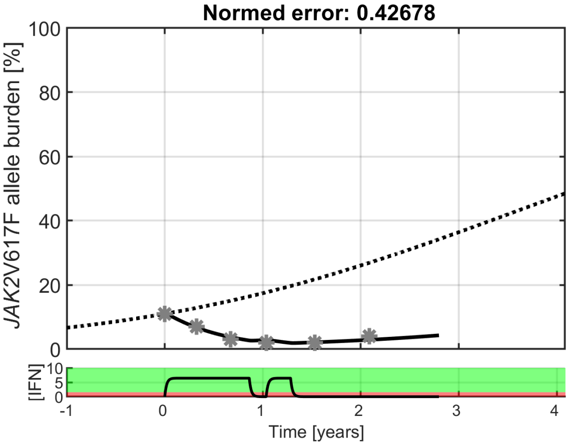

Patient P014

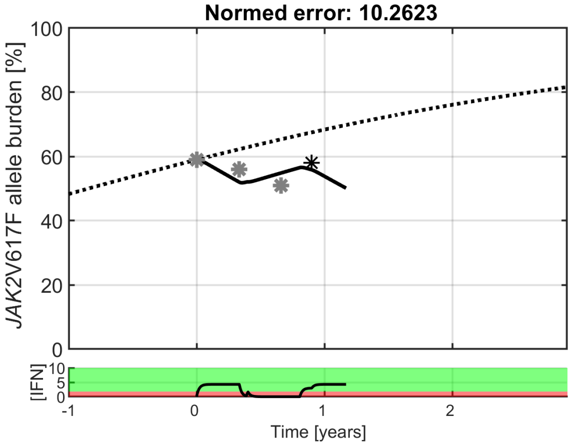

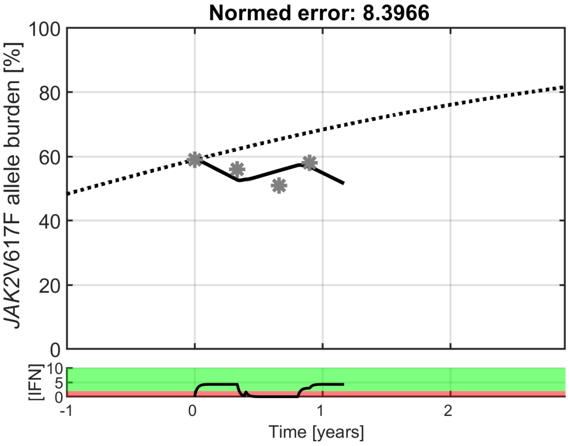

Patient P016

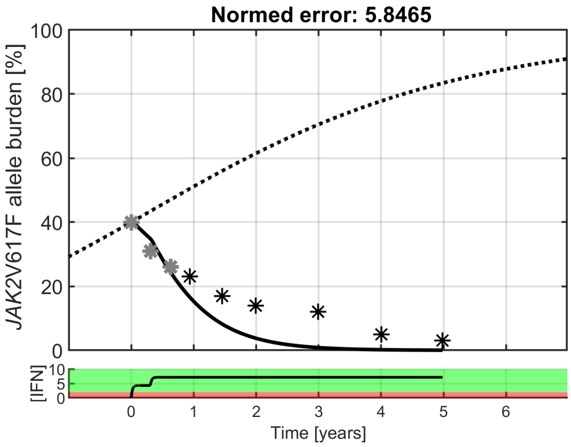

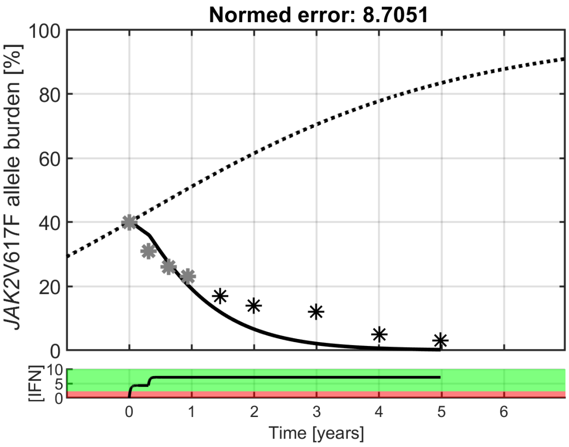

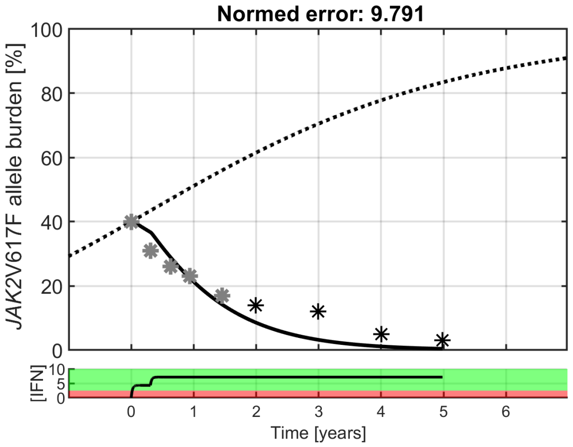

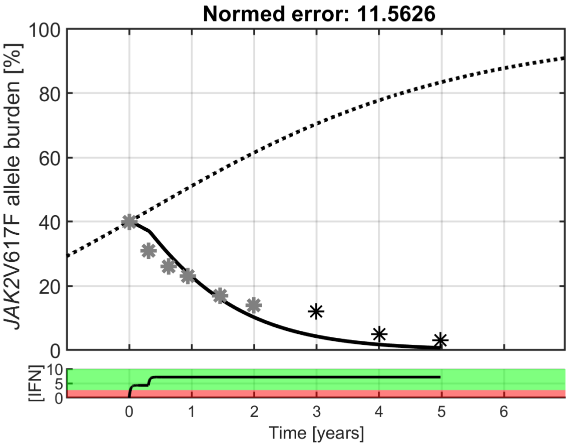

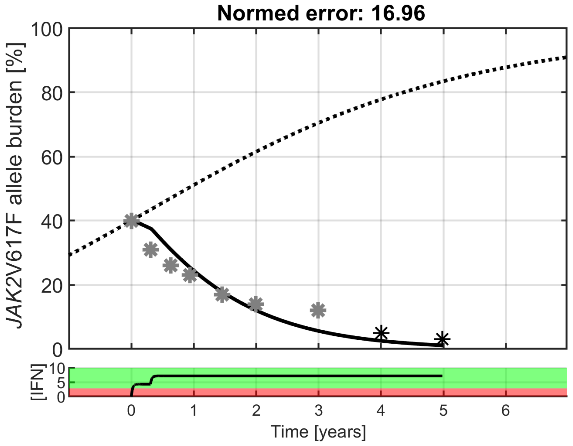

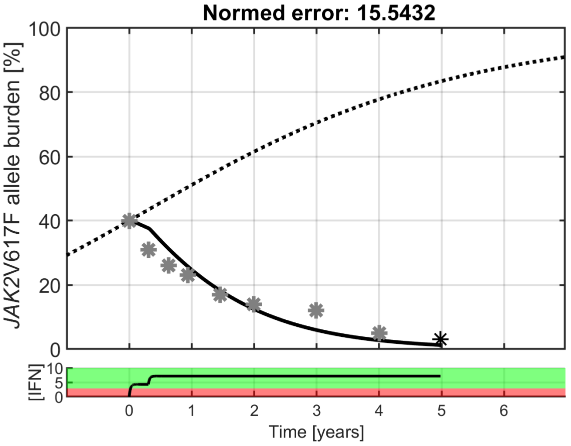

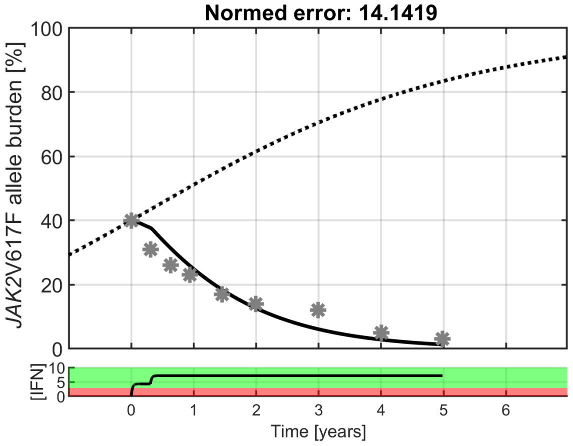

Patient P021

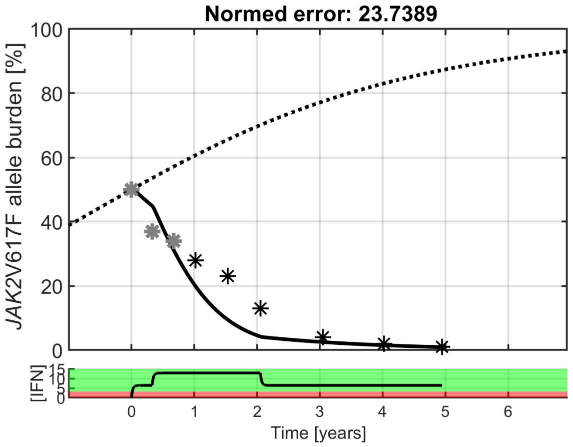

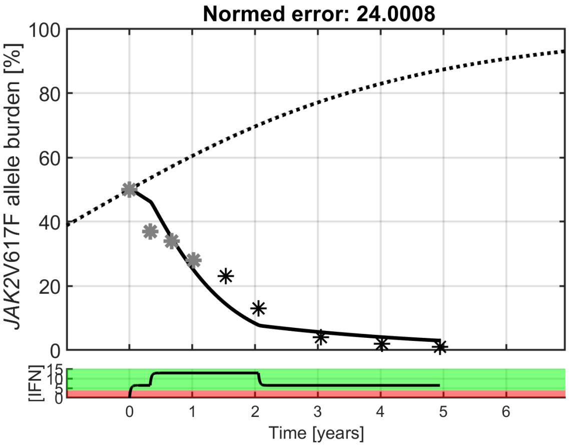

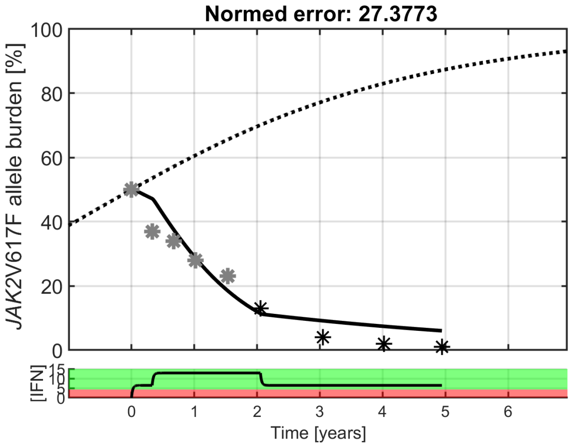

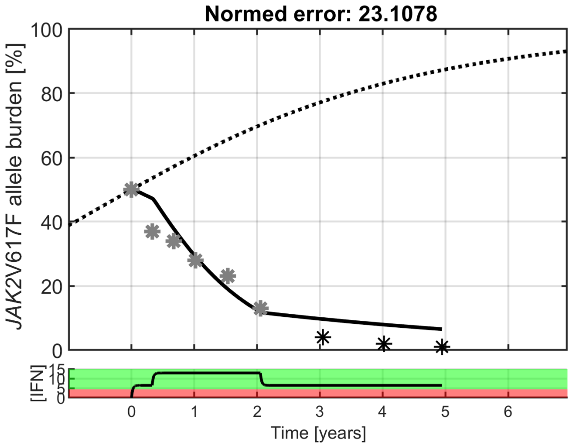

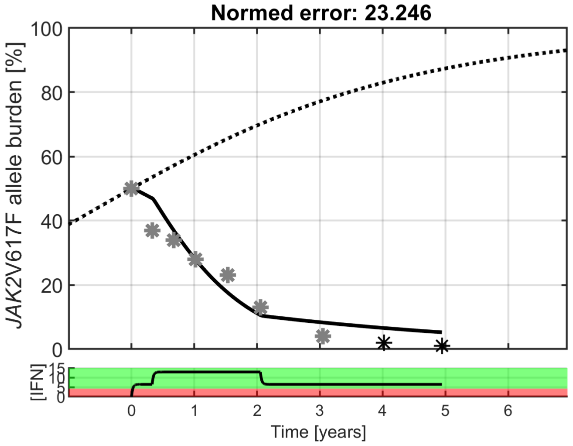

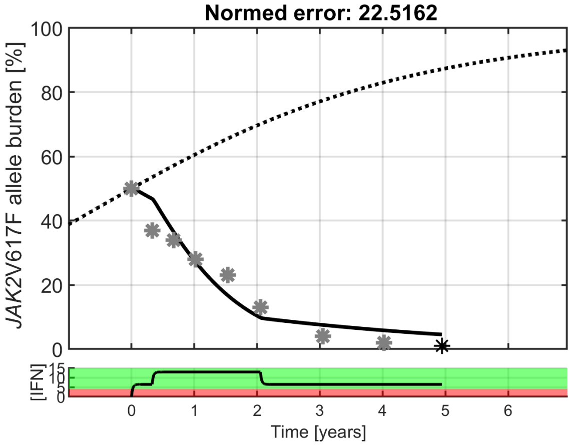

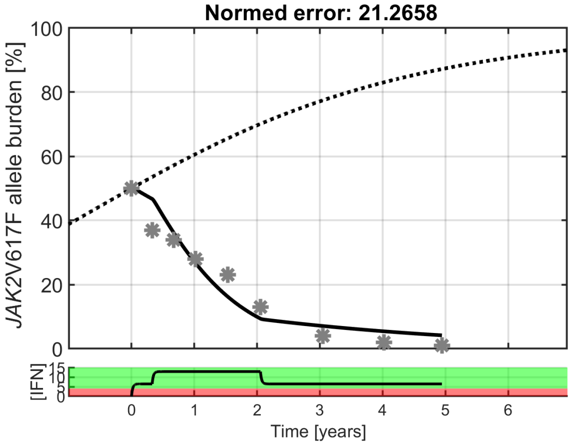

Patient P026

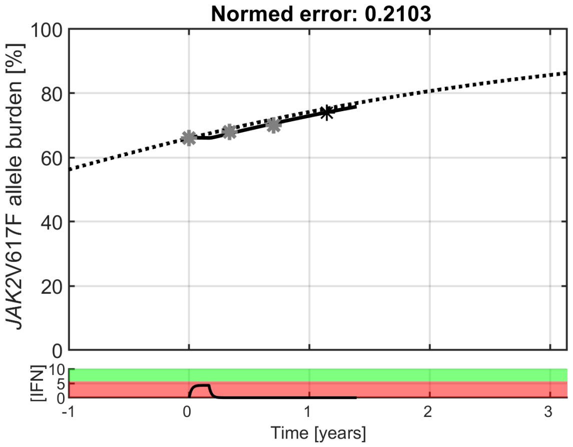

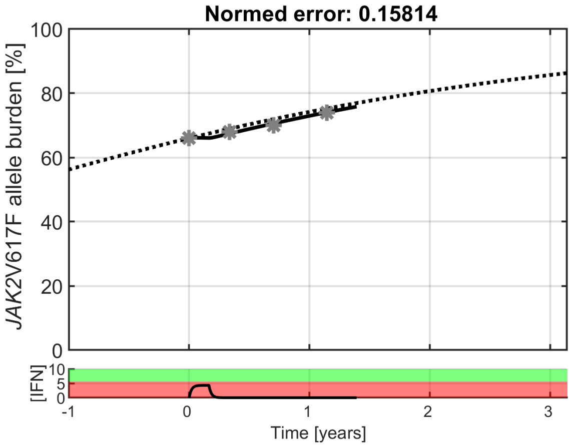

Patient P028

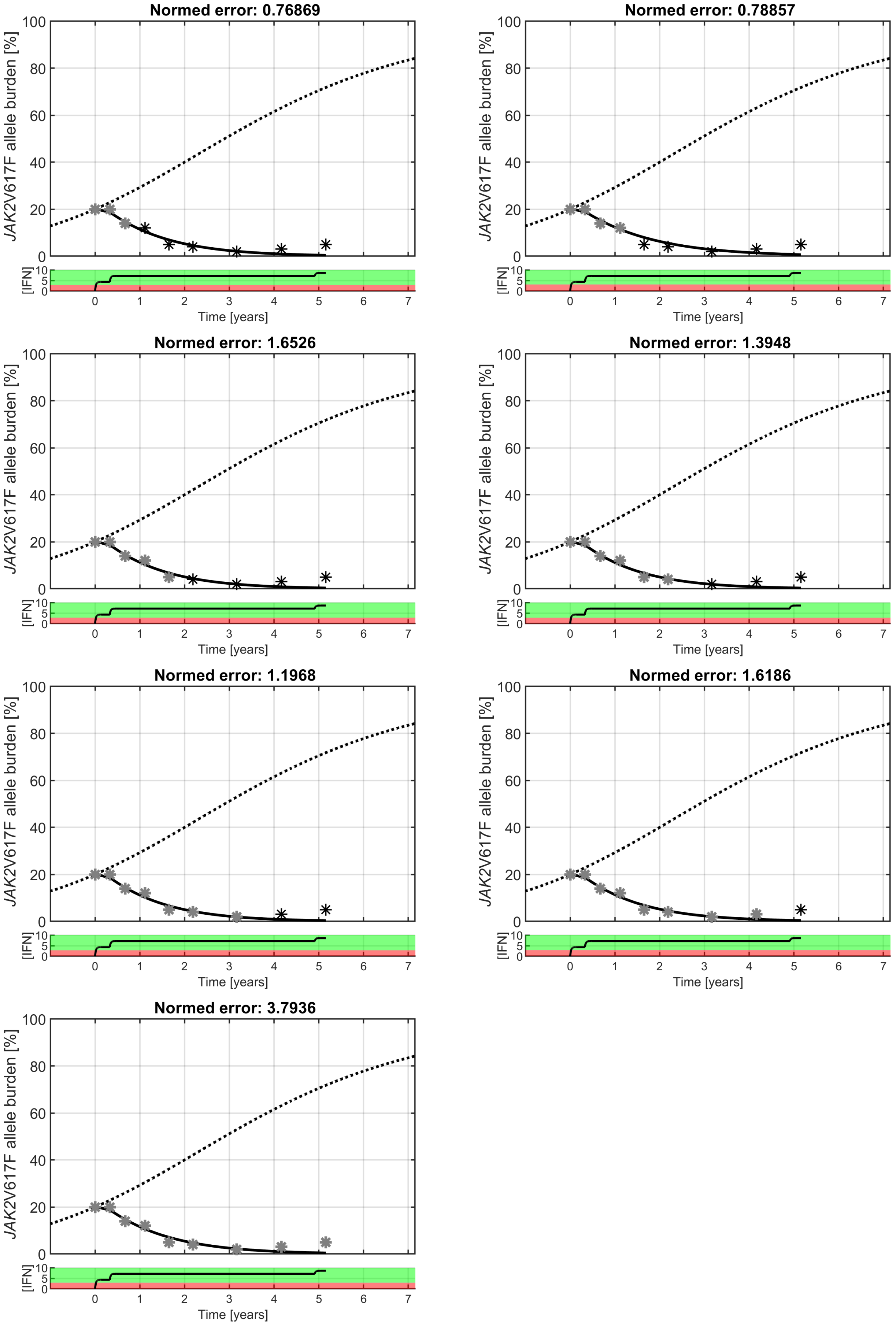

Patient P035

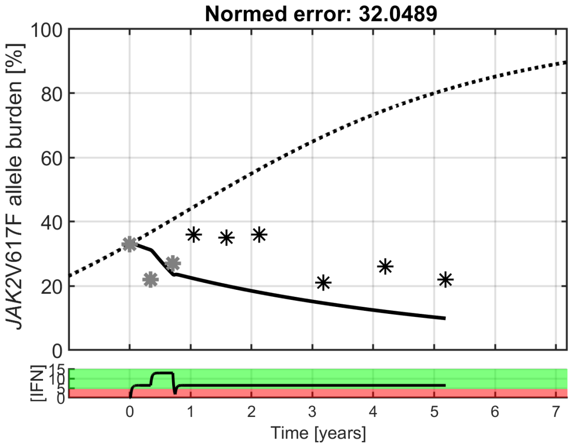

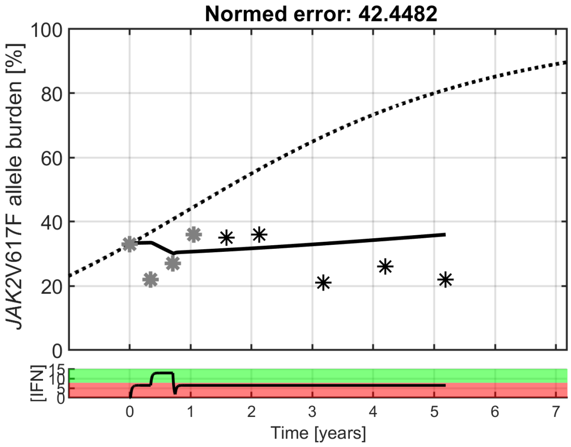

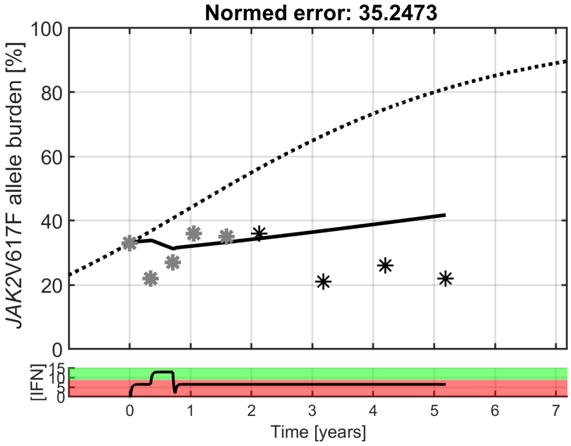

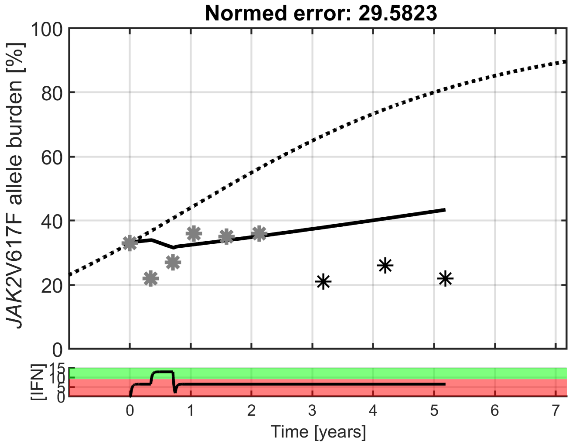

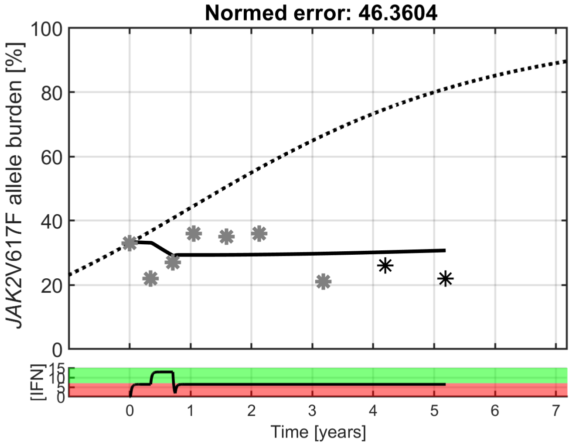

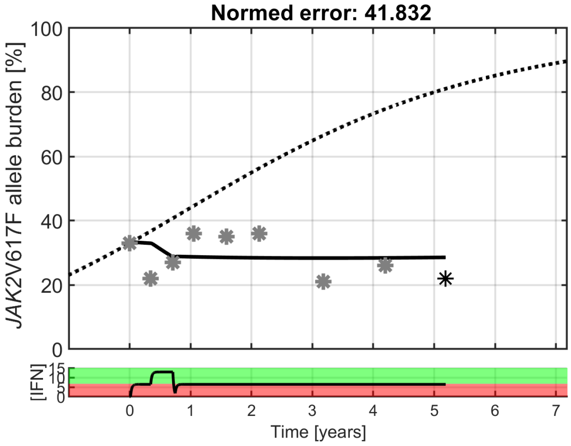

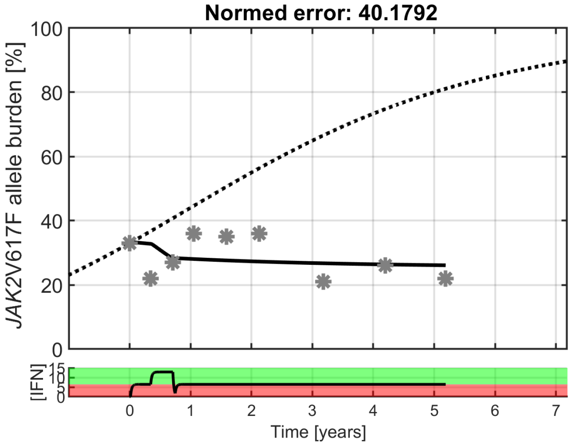

Patient P039

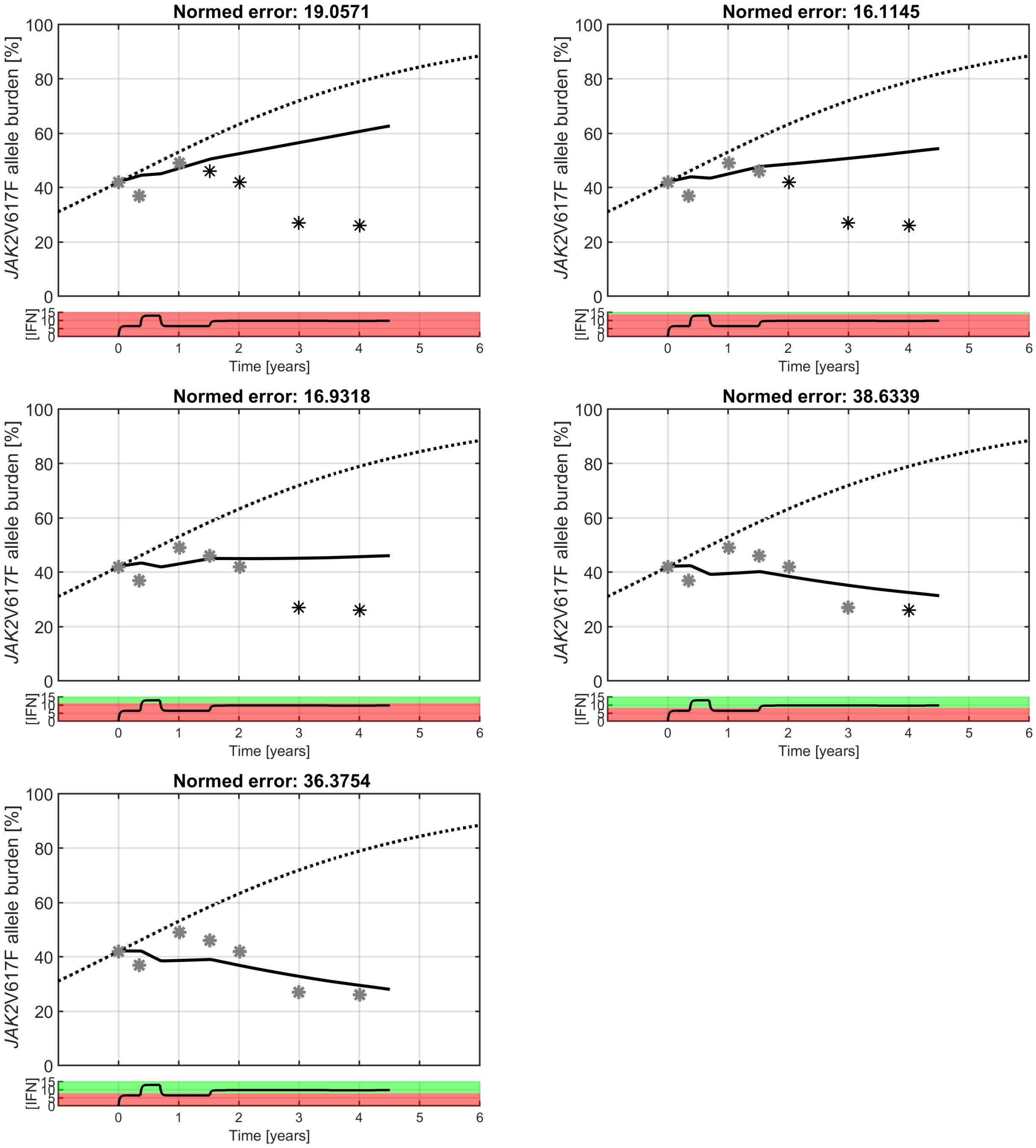

Patient P045

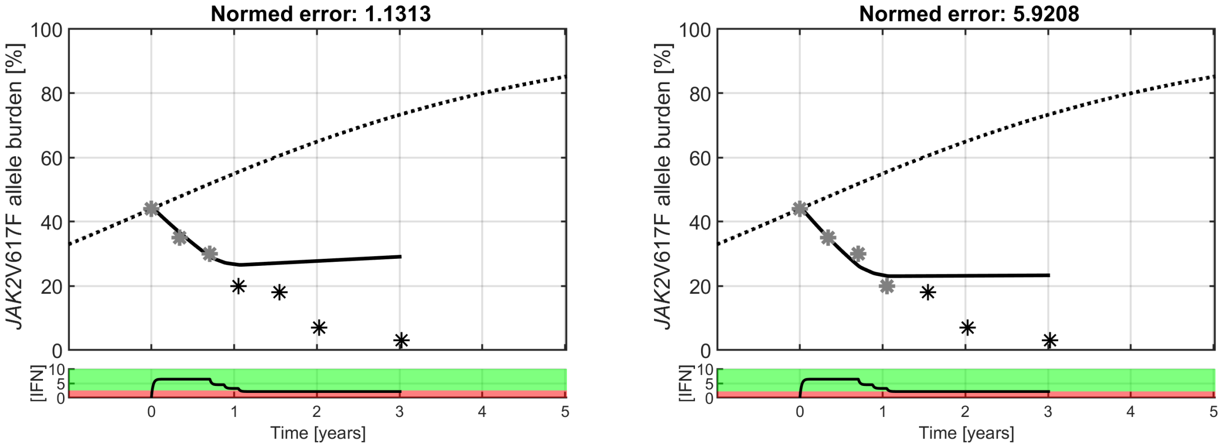

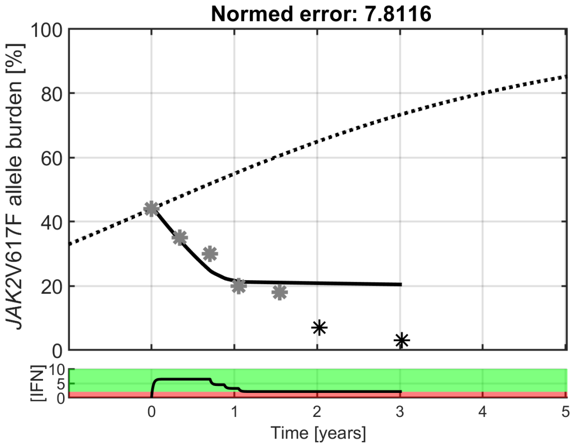

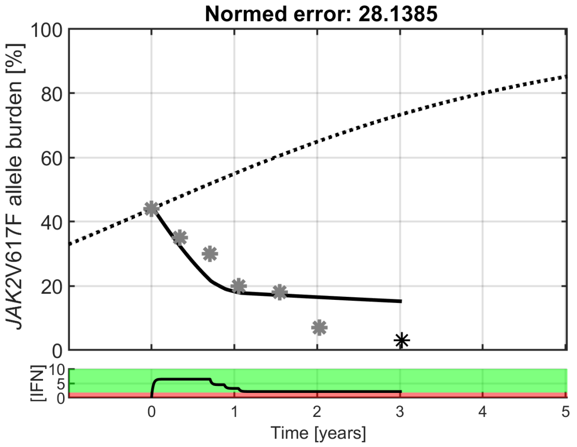

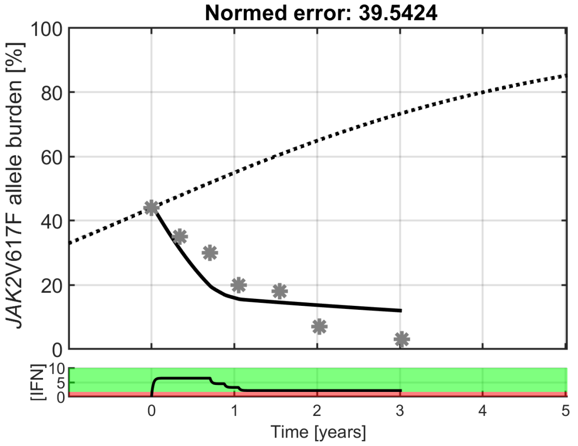

Patient P046

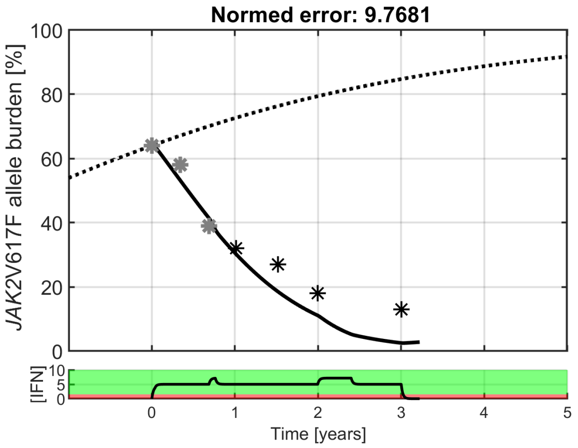

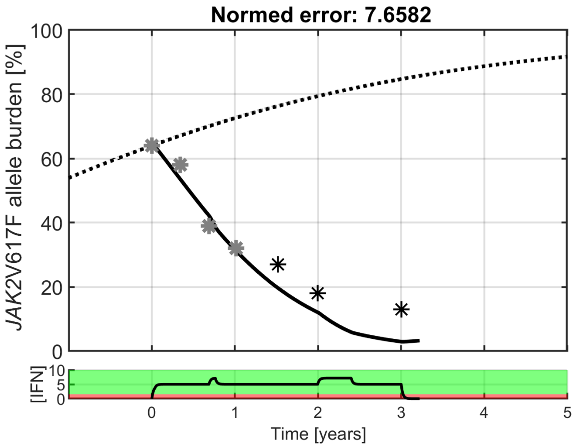

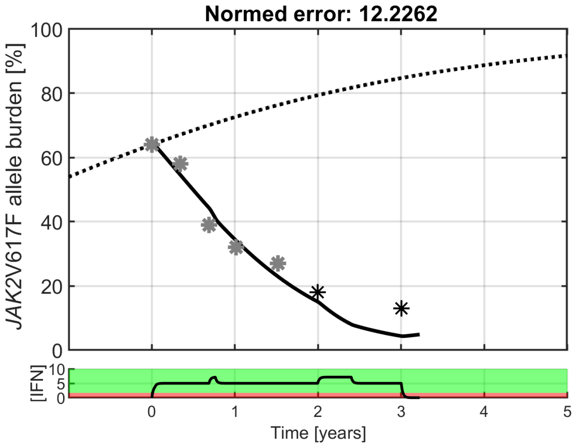

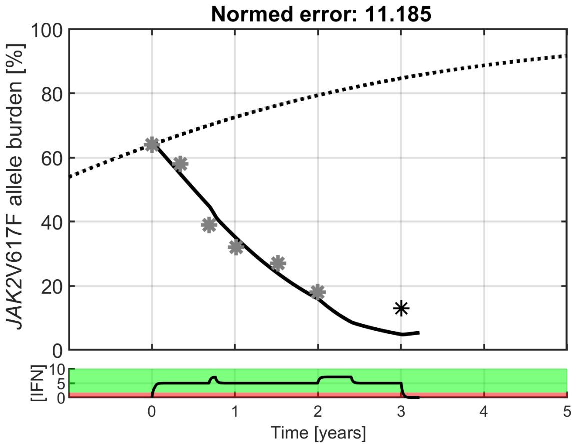

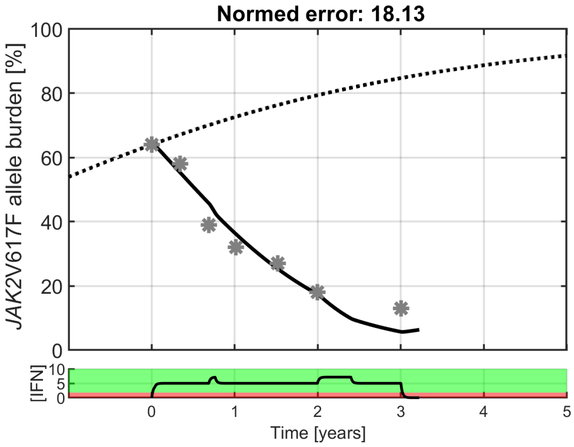

Patient P046

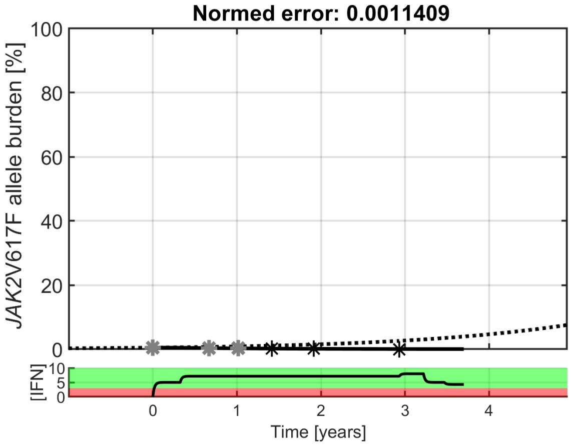

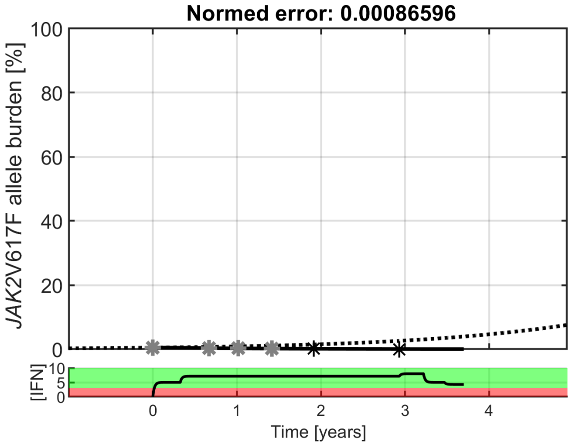

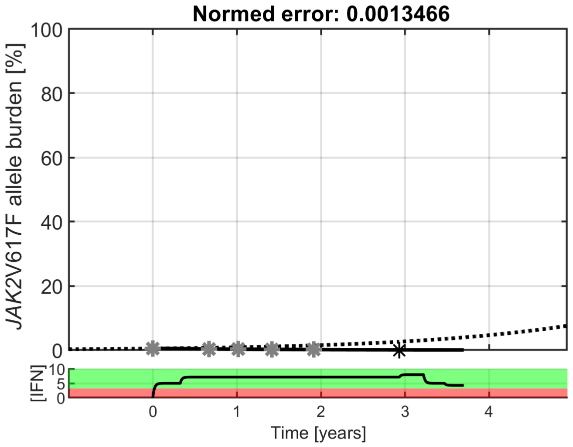

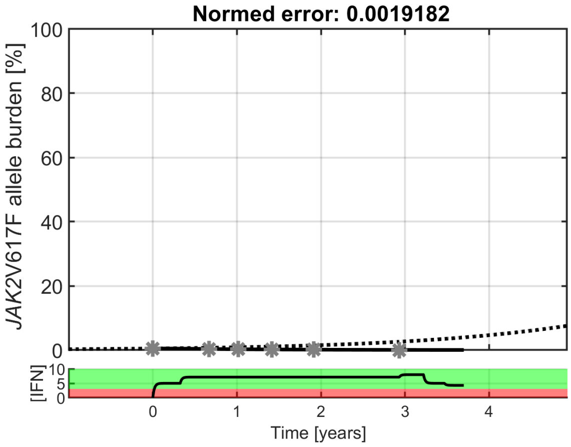

Patient P062

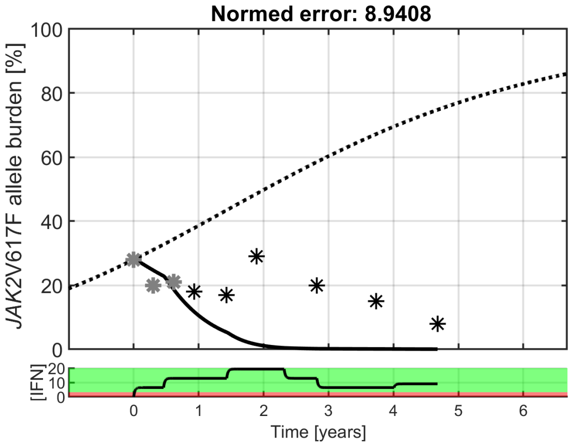

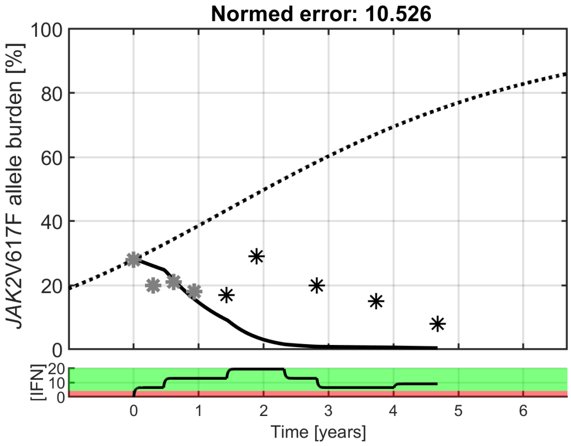

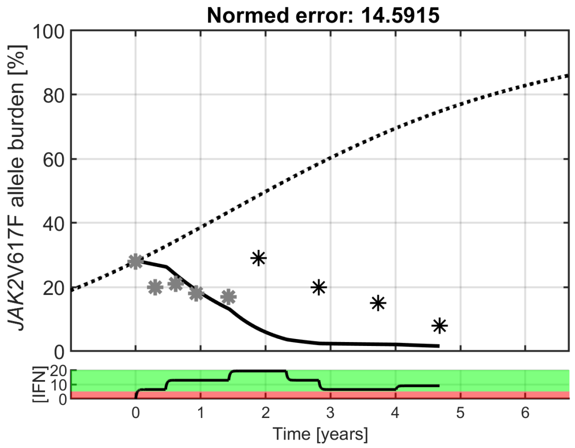

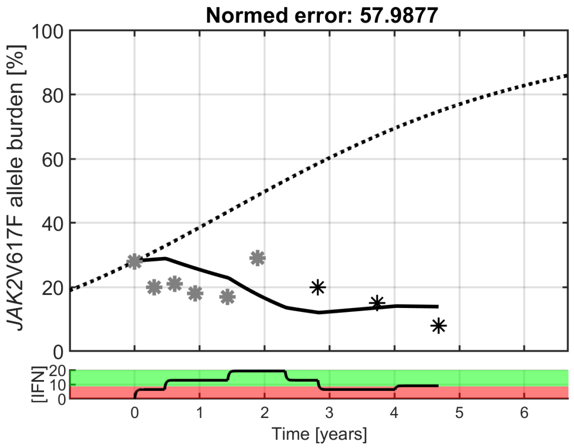

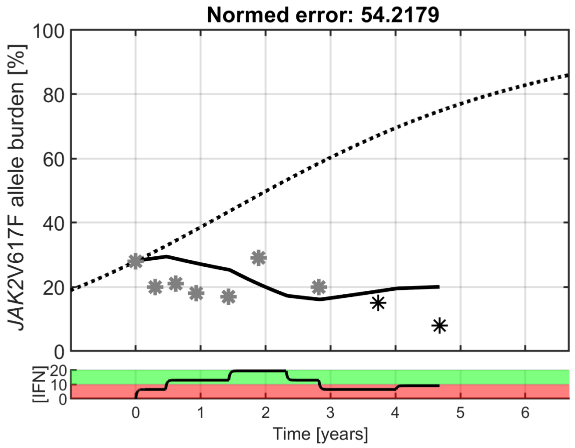

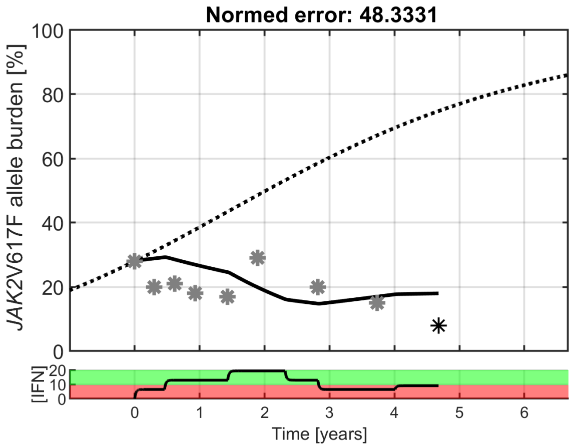

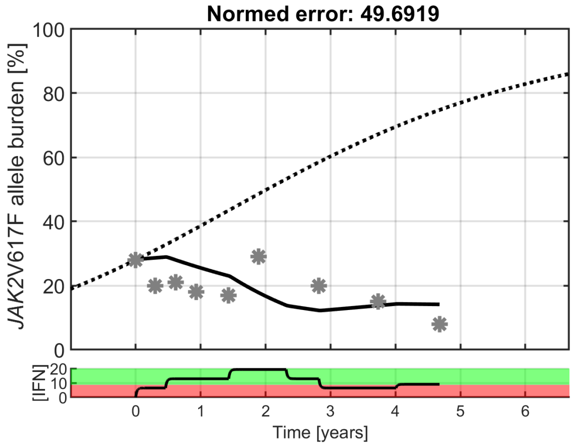

Patient P067

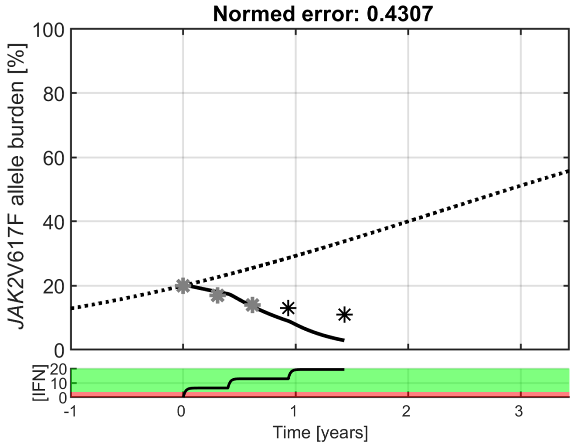

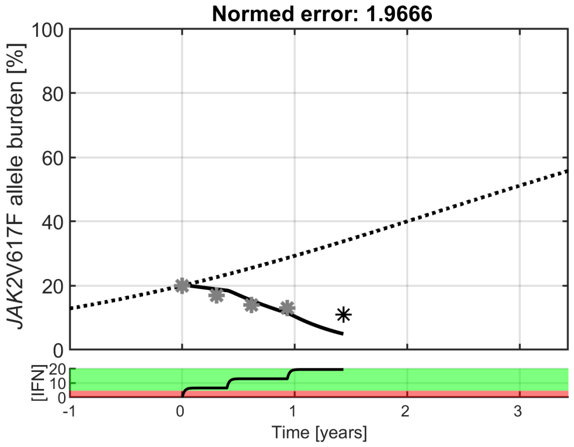

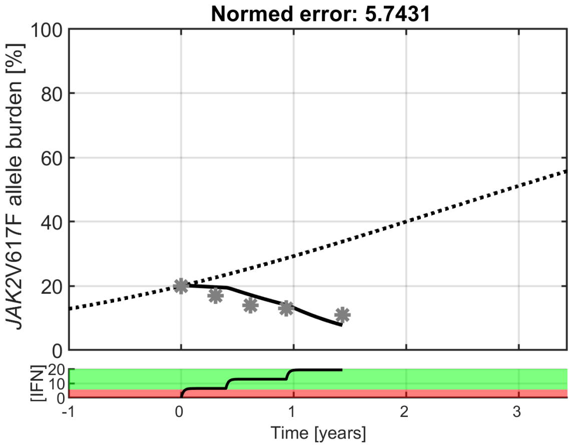

Patient P070

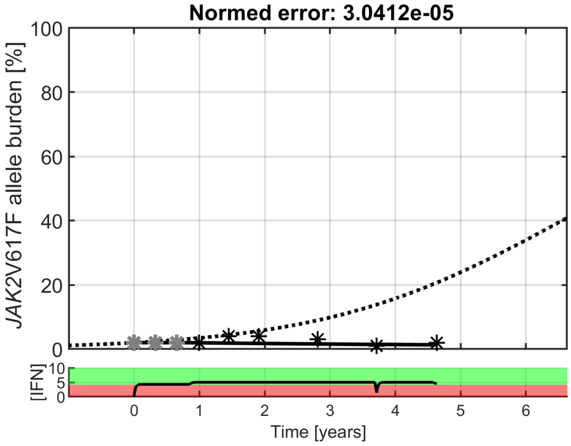

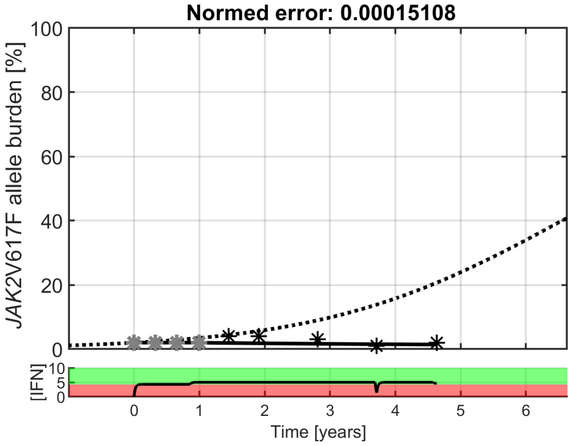

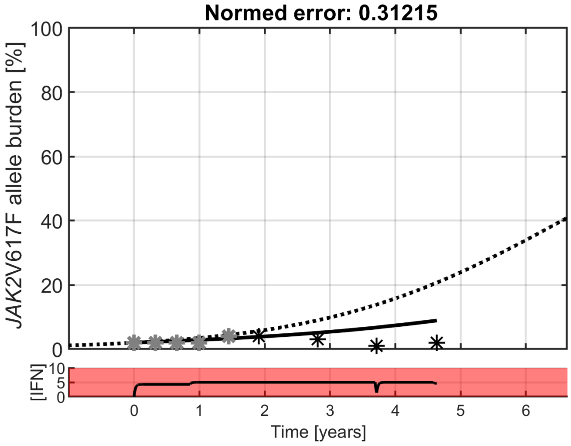

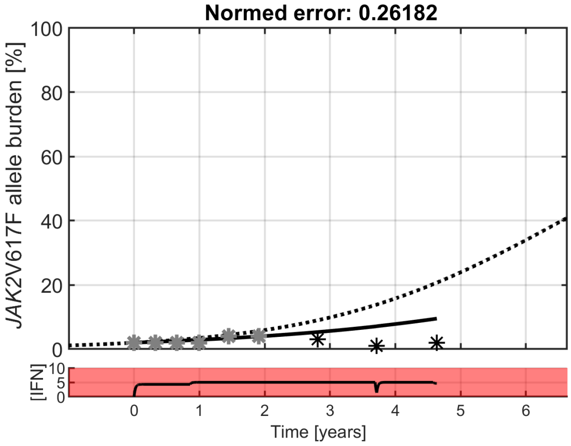

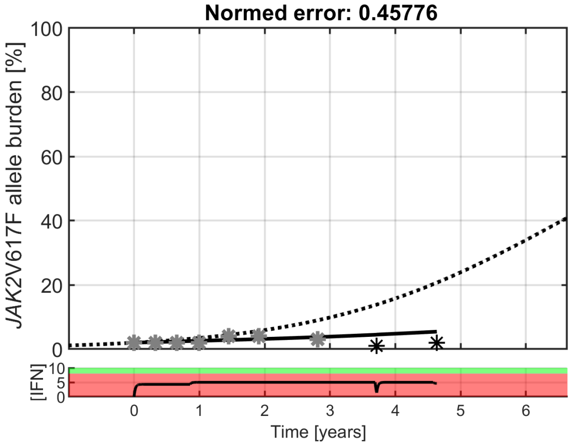

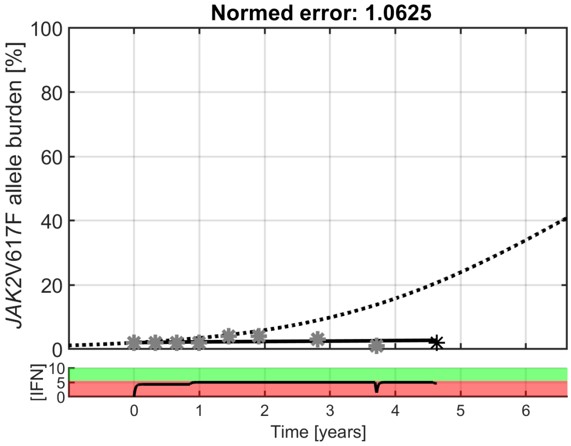

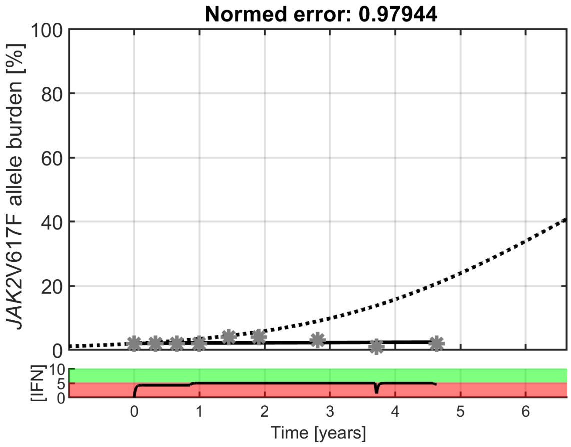

Patient P075

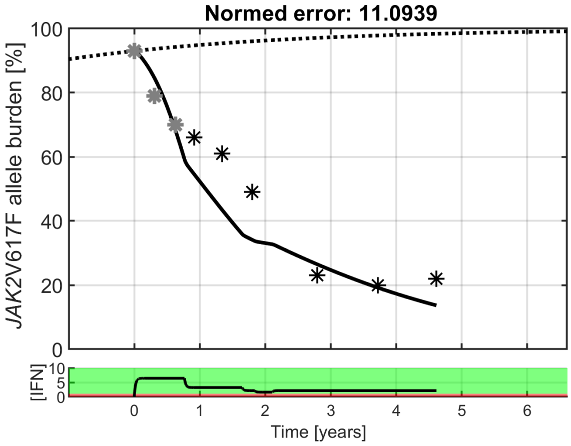

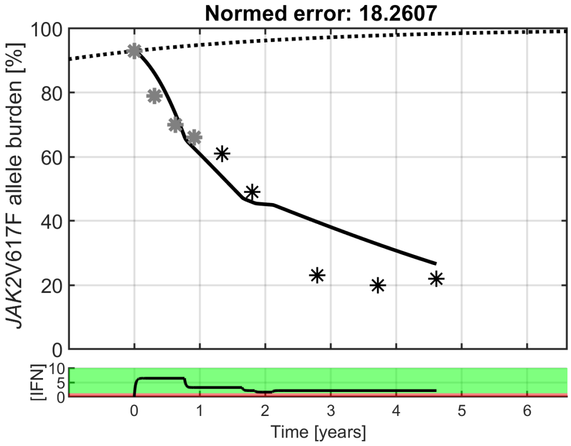

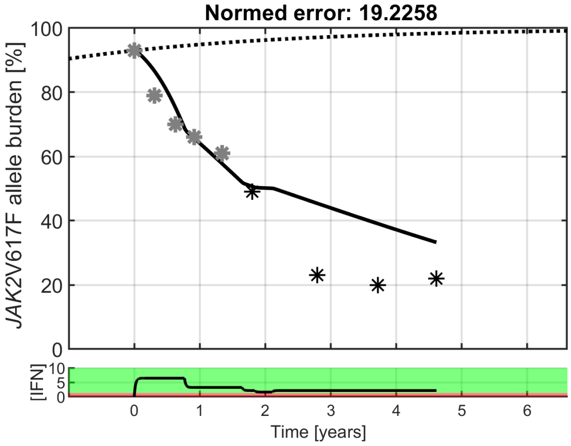

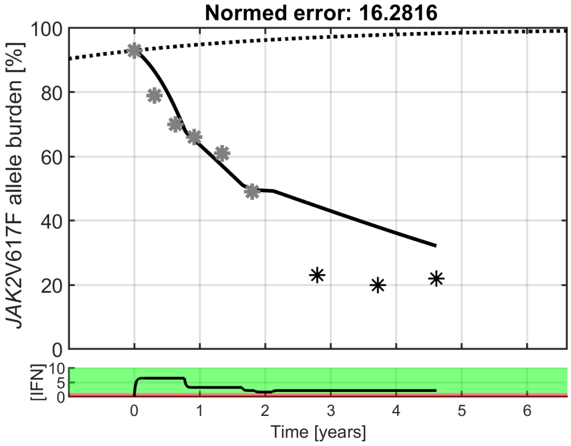

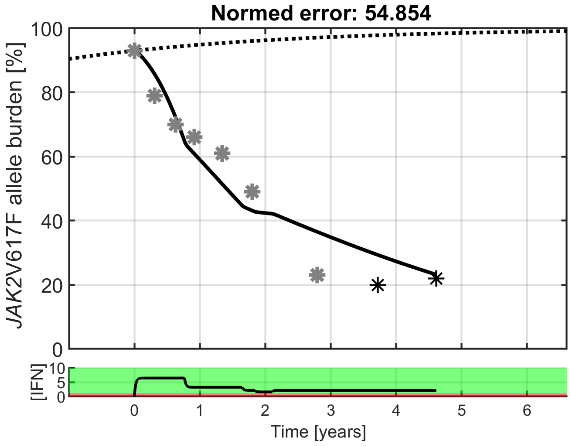

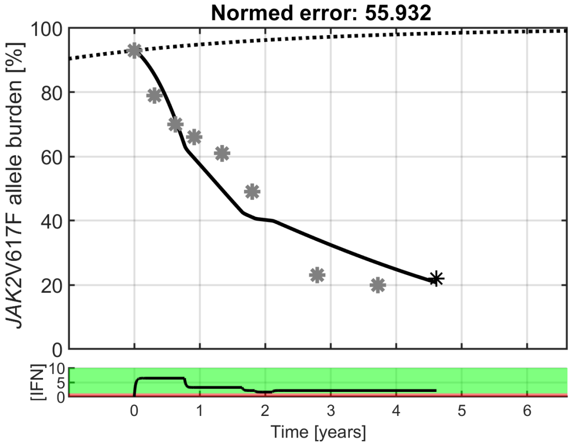

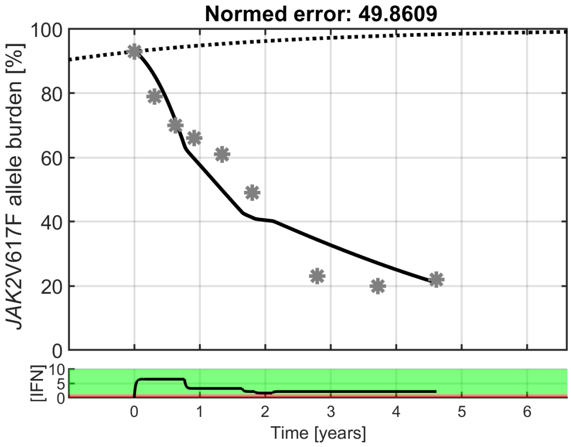

Patient P078

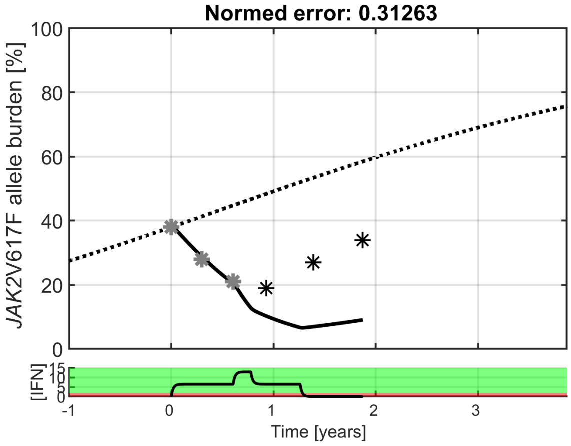

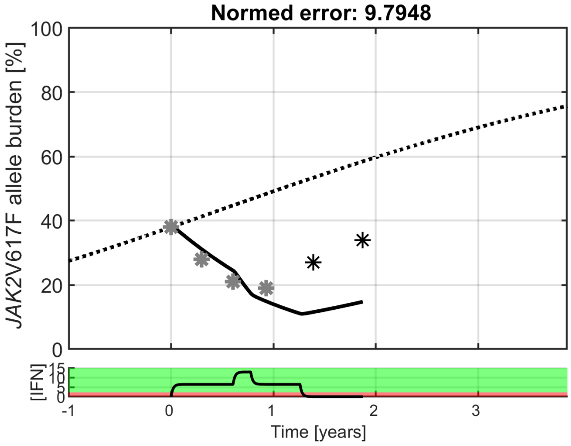

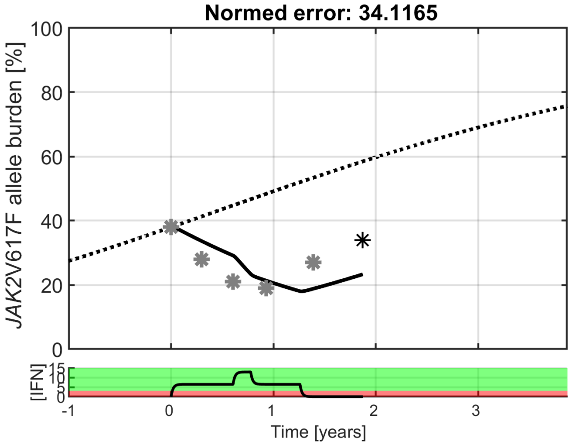

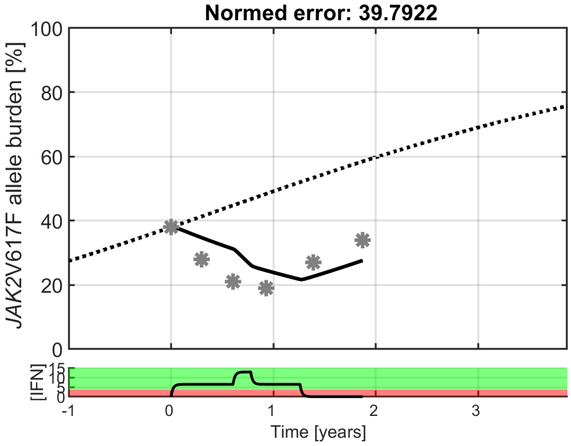

Patient P079

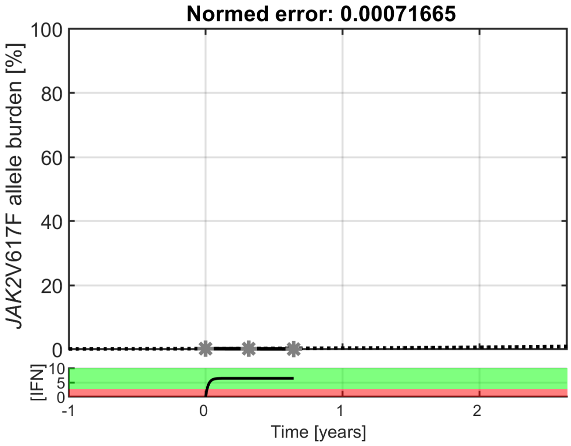

Patient P082

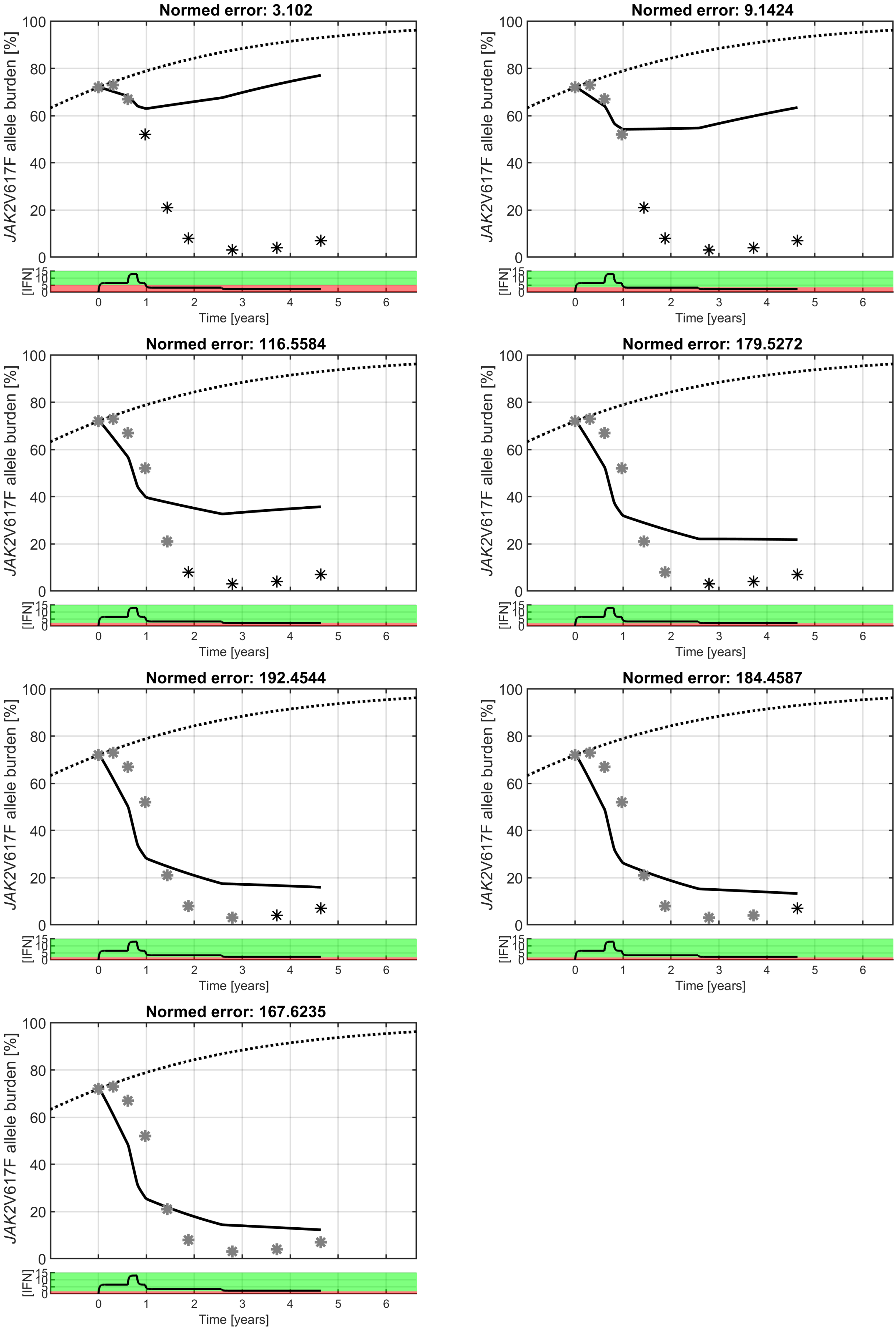

Patient P084

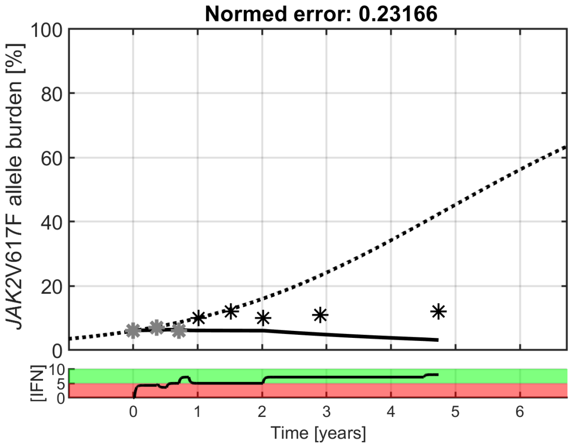

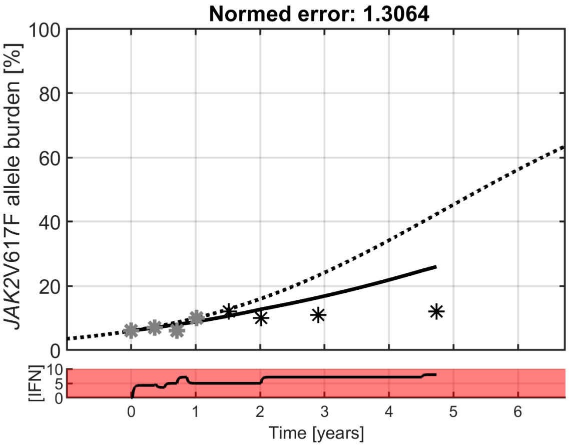

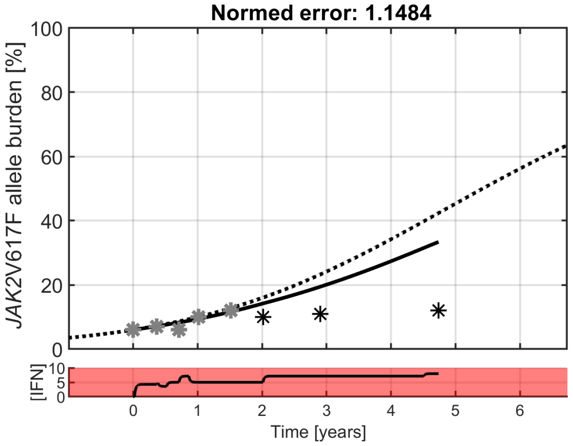

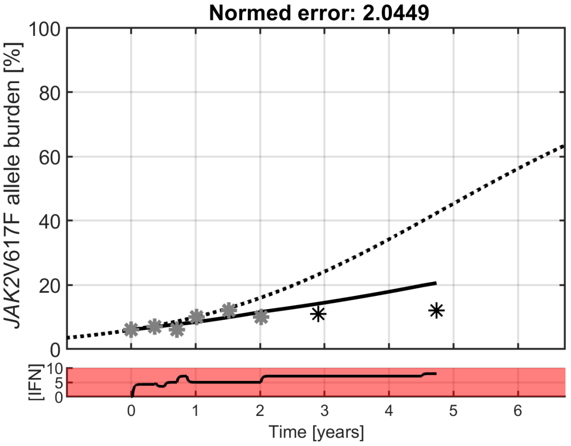

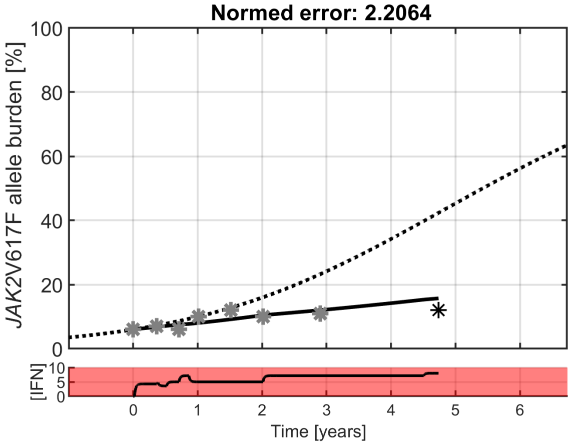

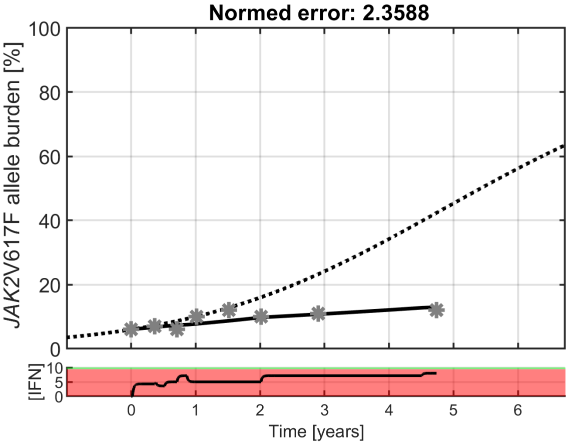

Patient P086

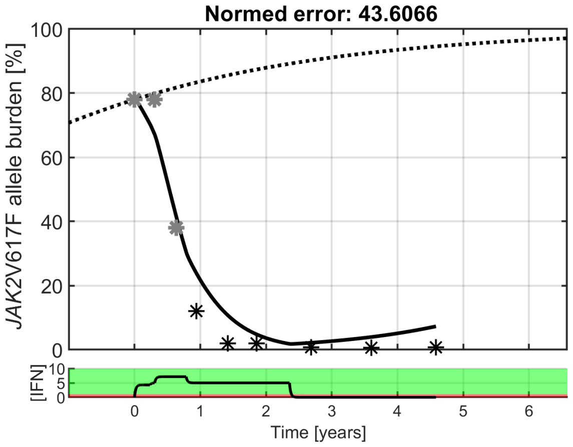

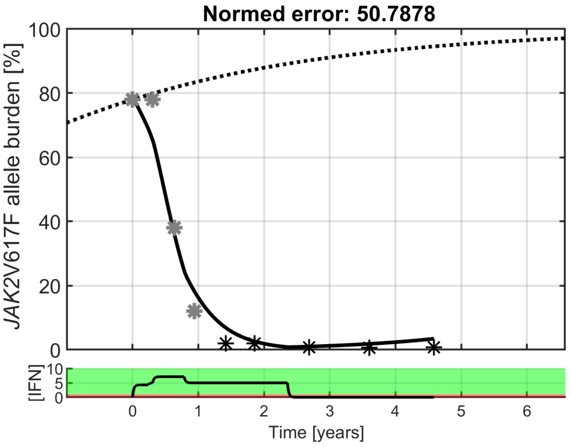

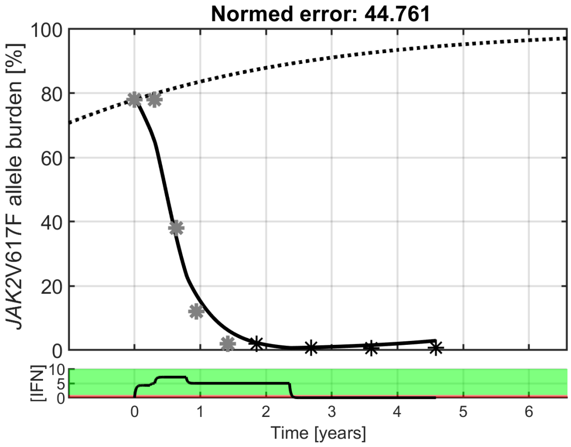

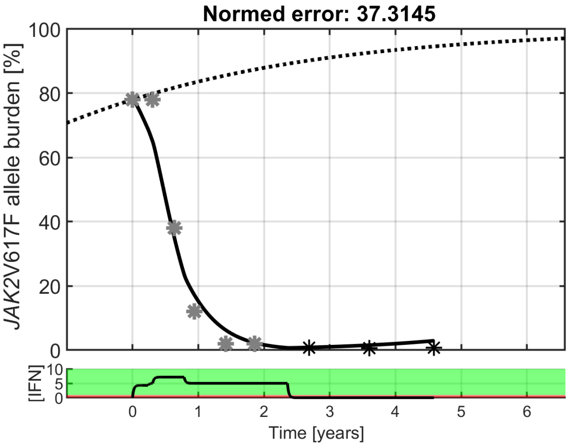

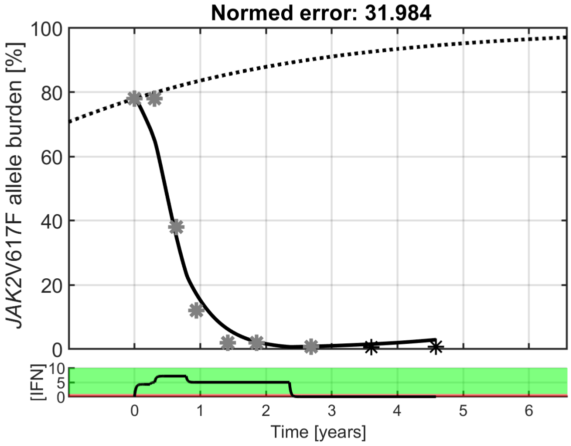

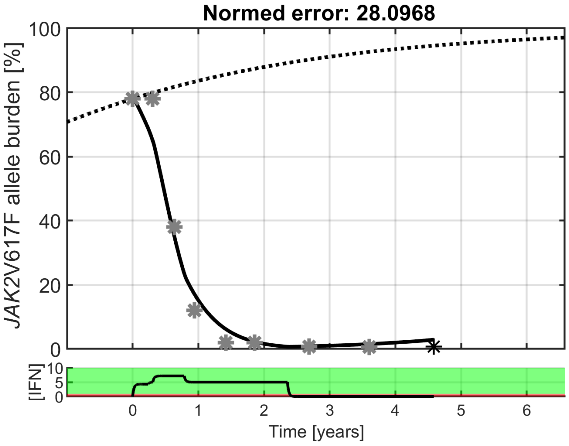

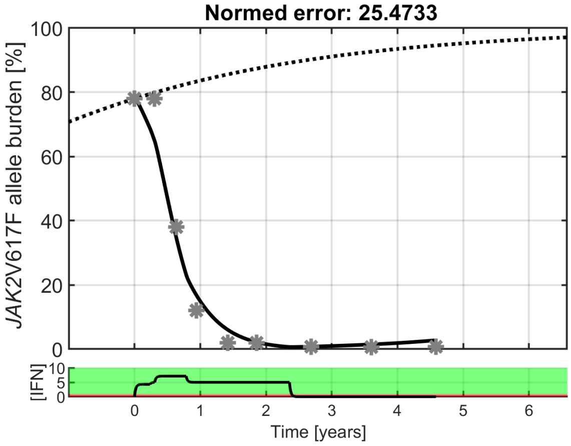

Patient P087

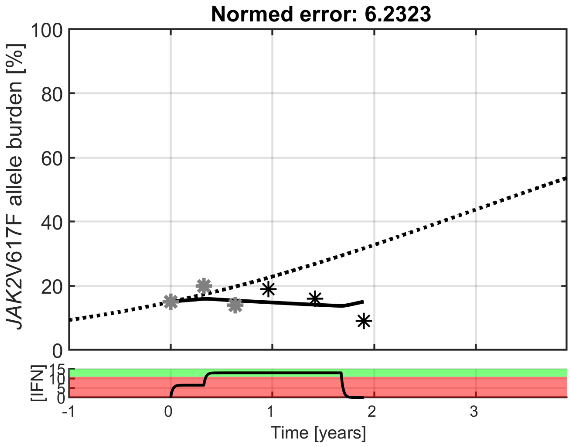

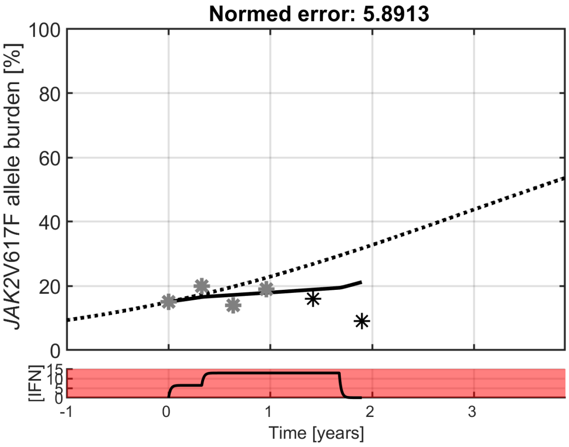

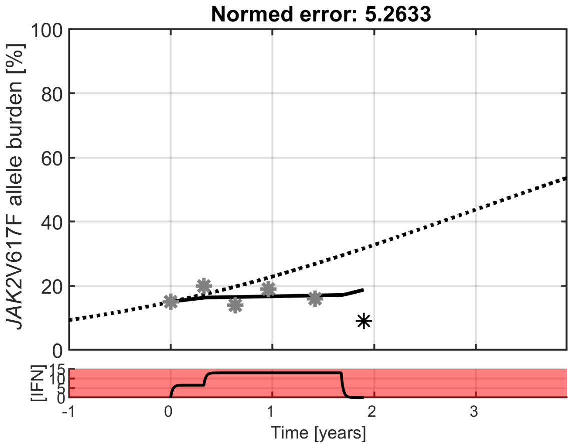

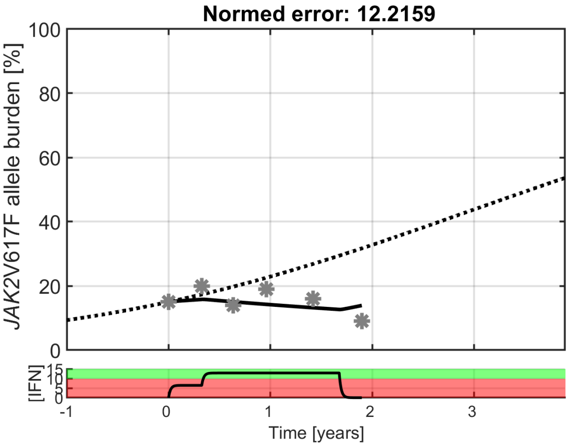

Patient P089

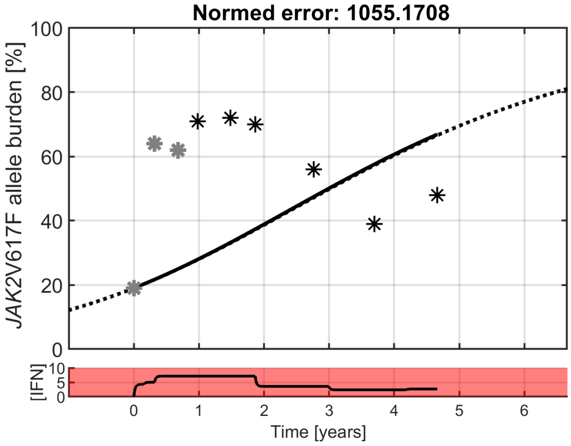

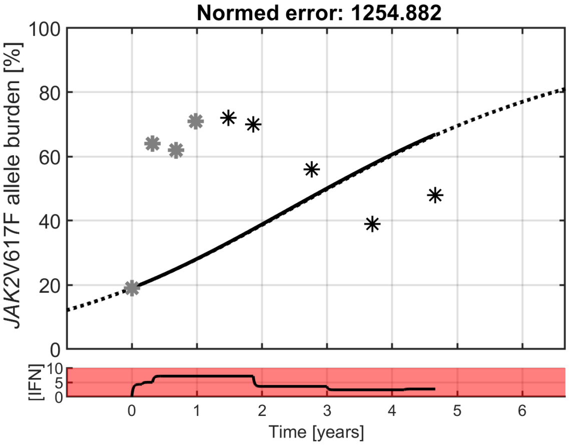

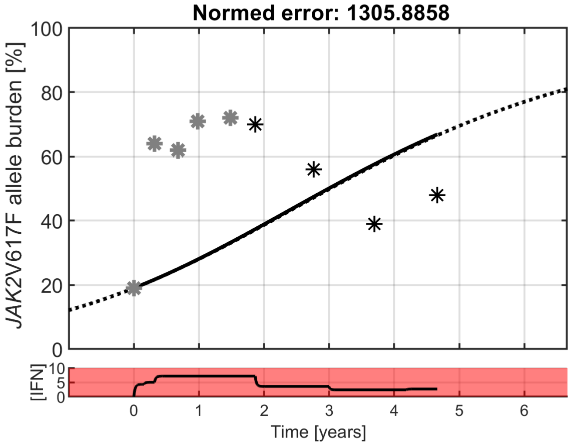

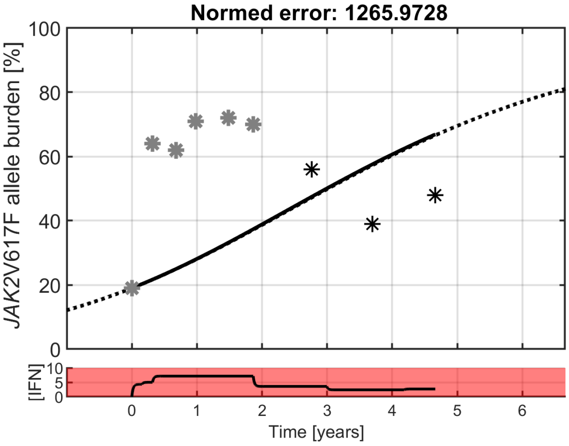

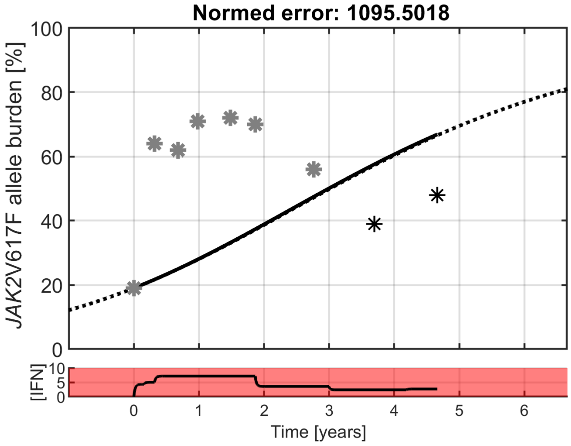

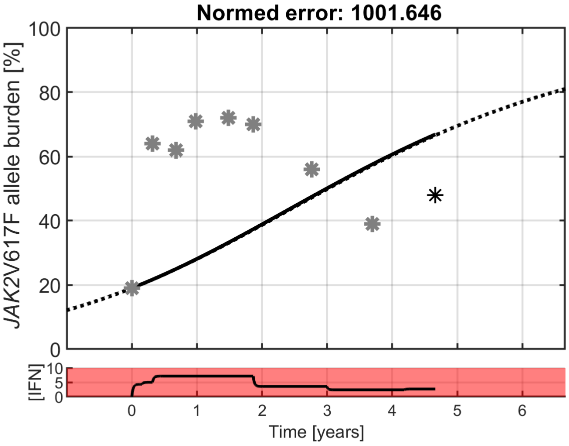

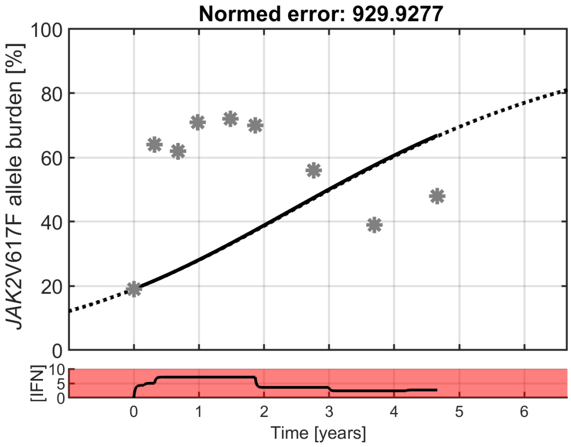

Patient P105

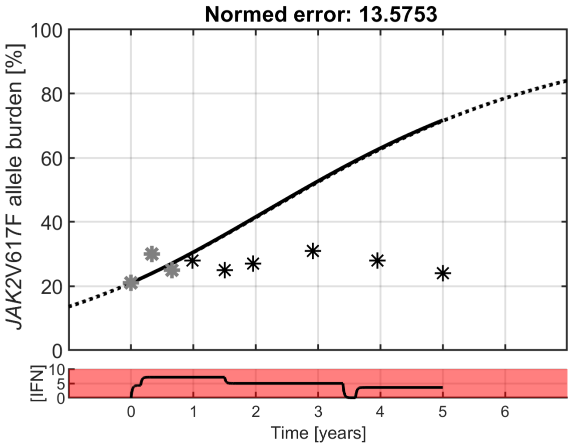

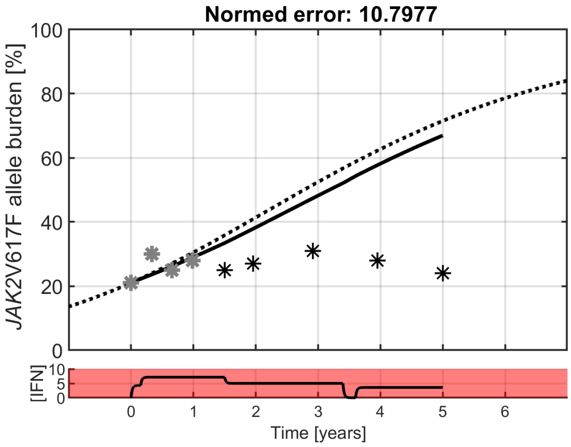

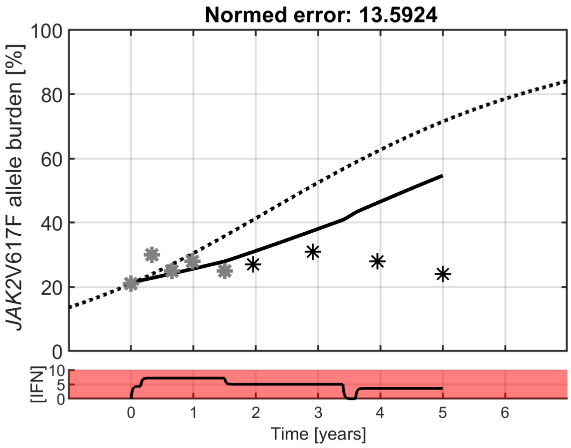

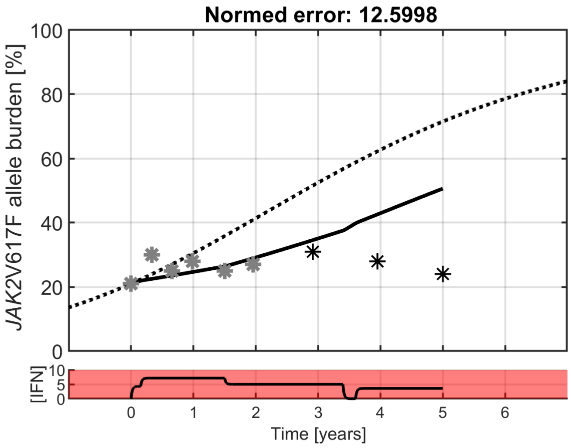

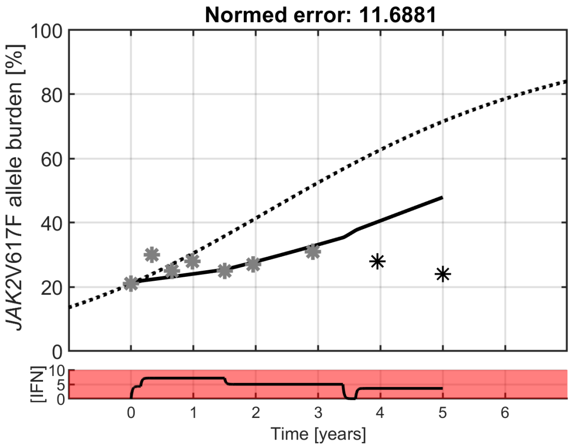

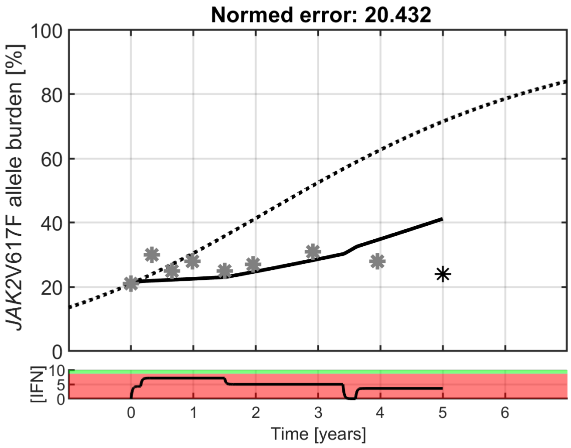

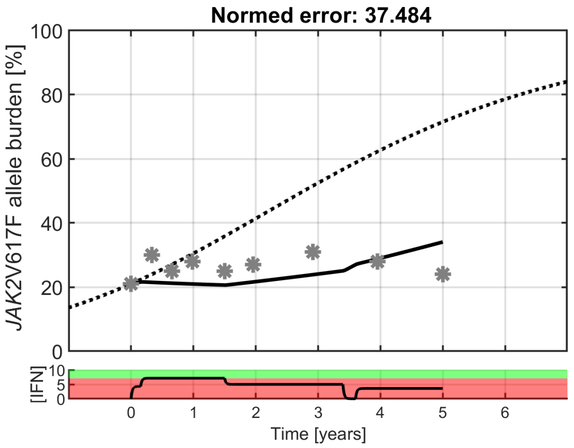

Patient P106

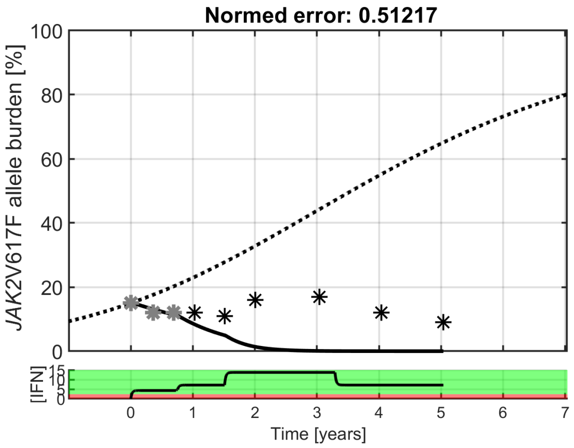

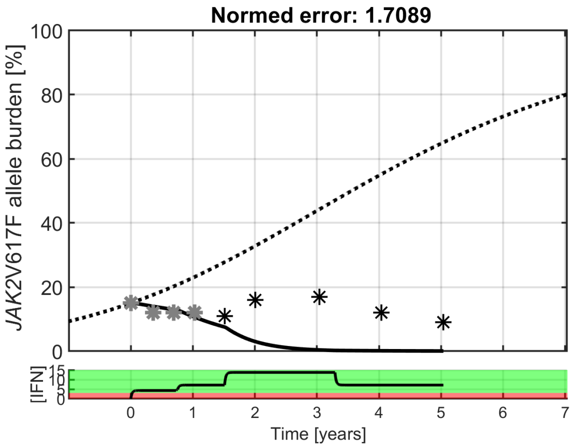

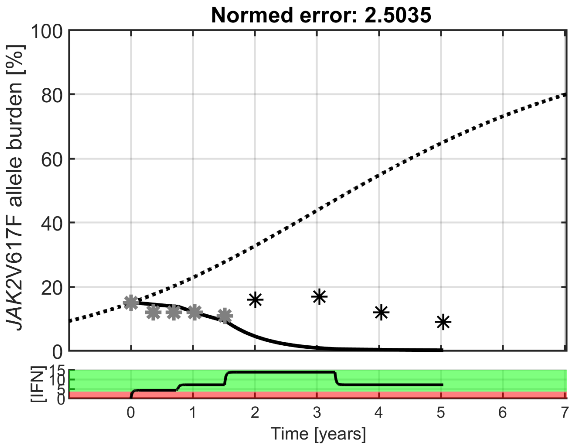

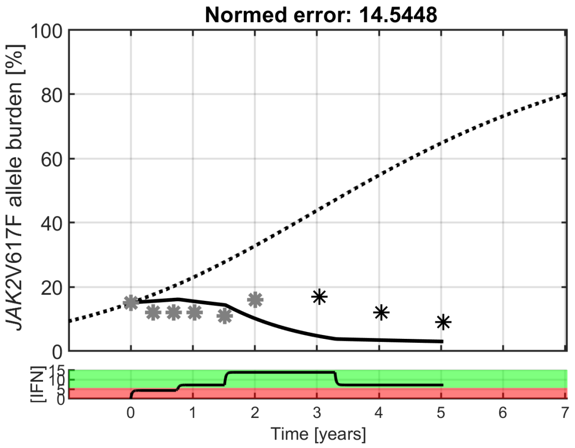

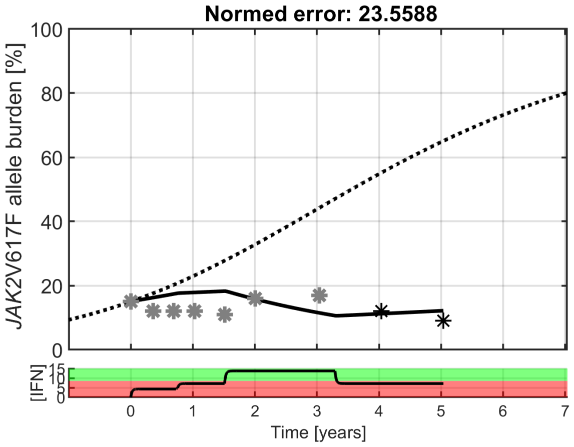

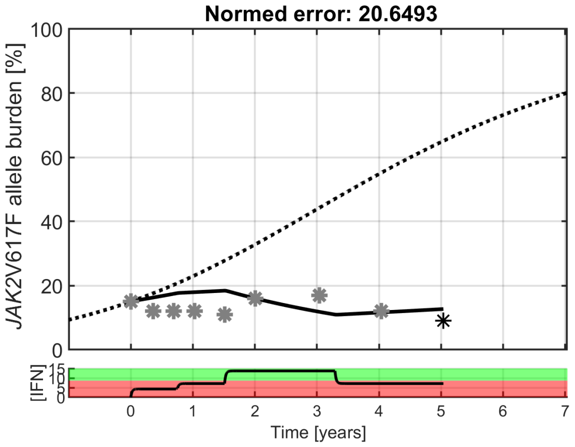

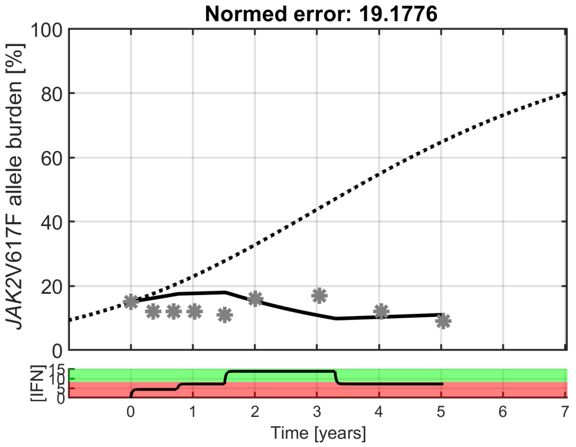

Patient P111

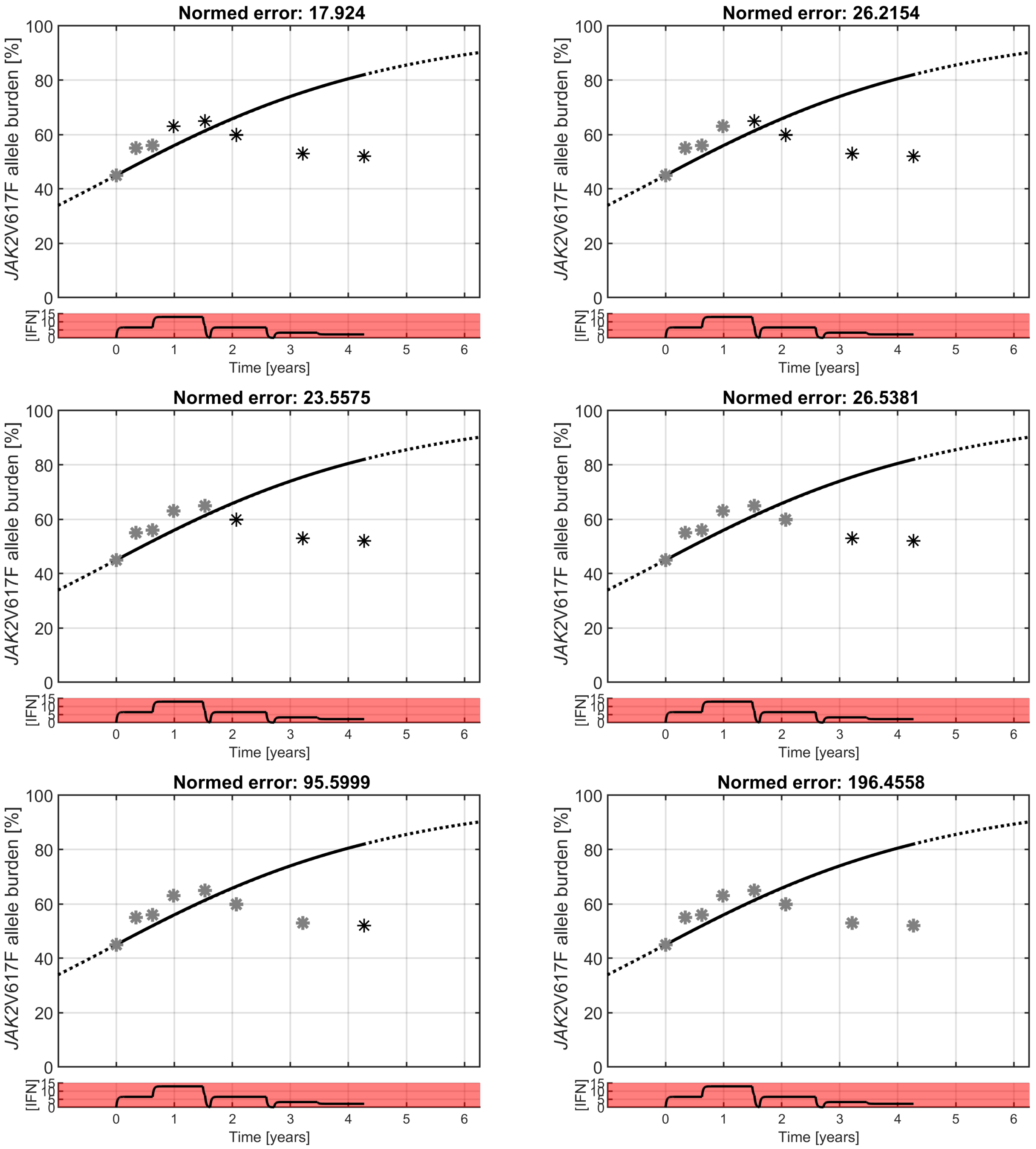

Patient P113

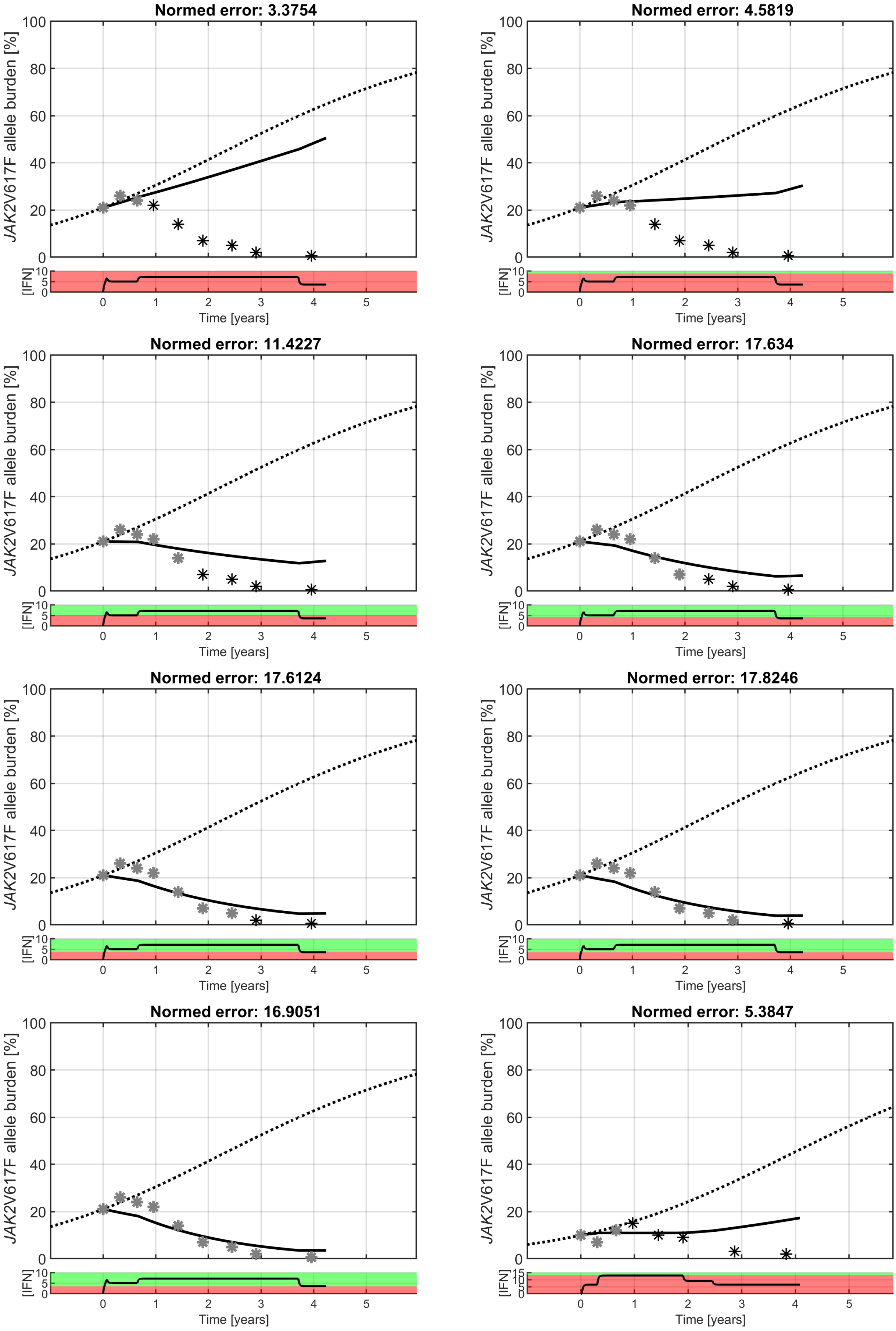

Patient P114

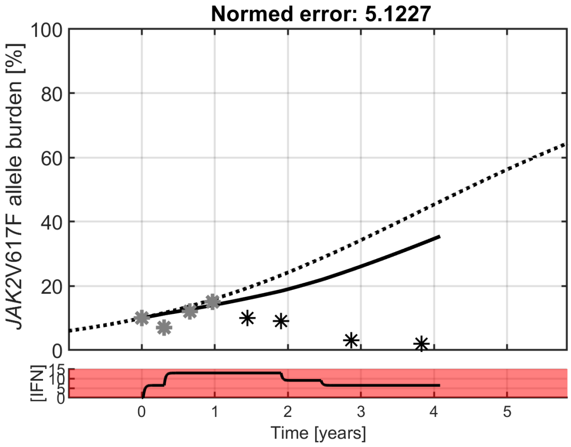

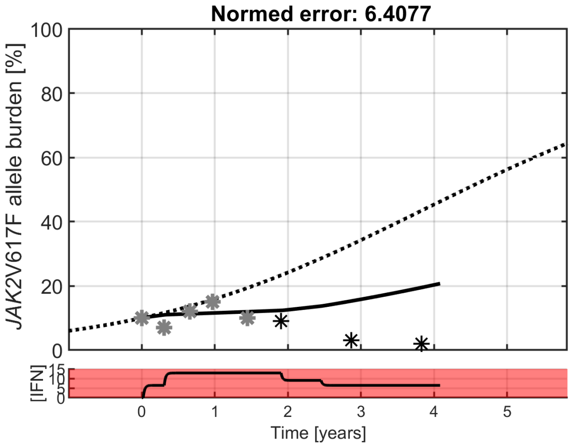

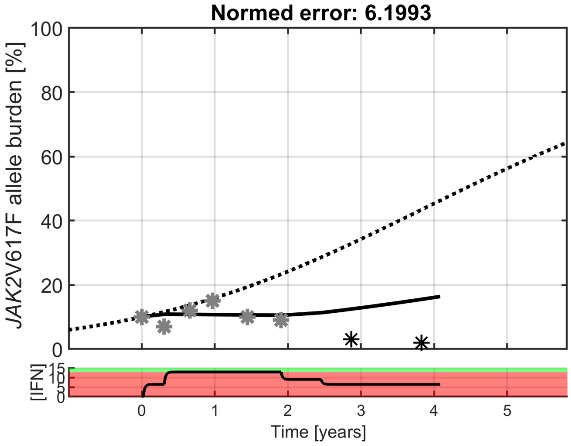

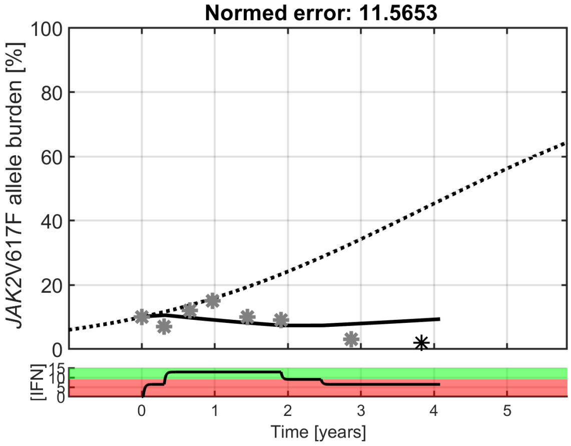

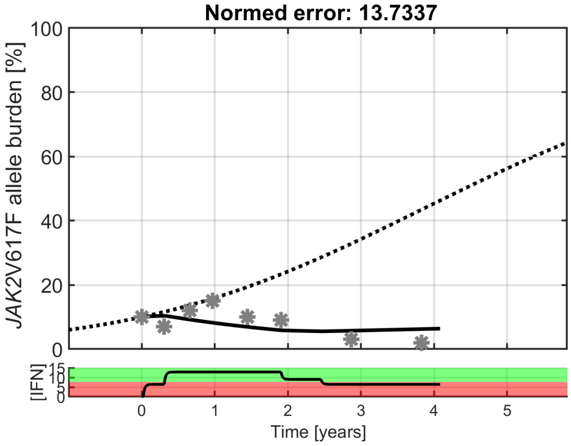

Patient P117

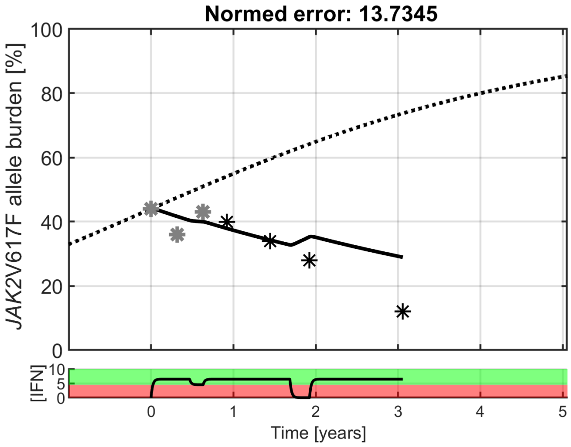

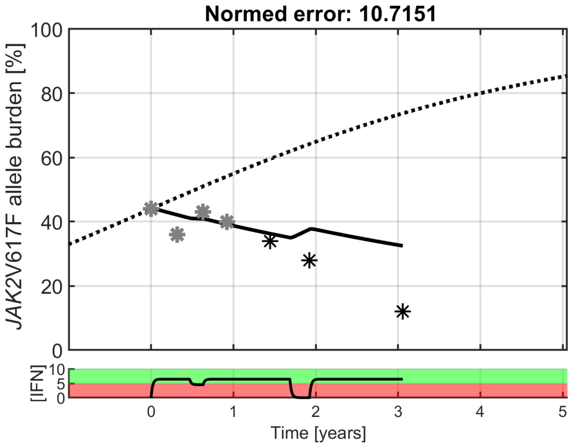

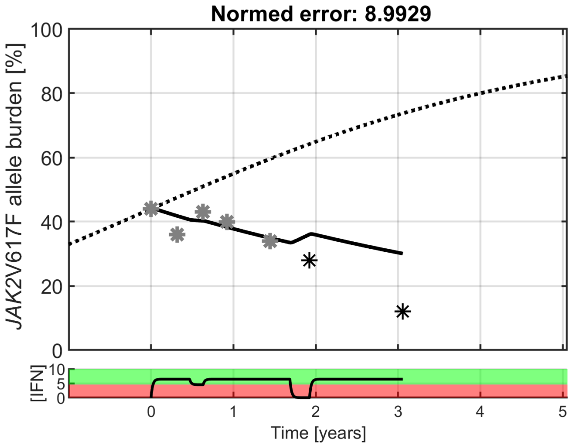

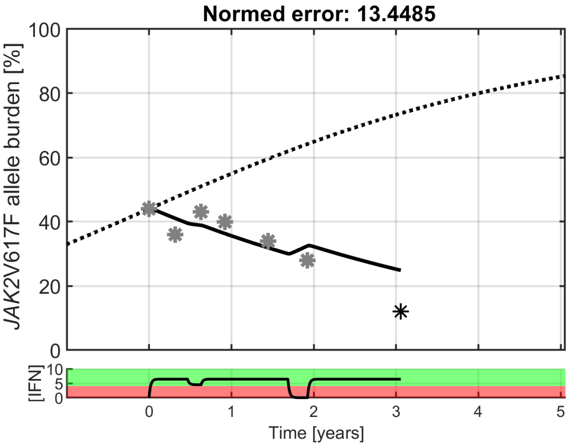

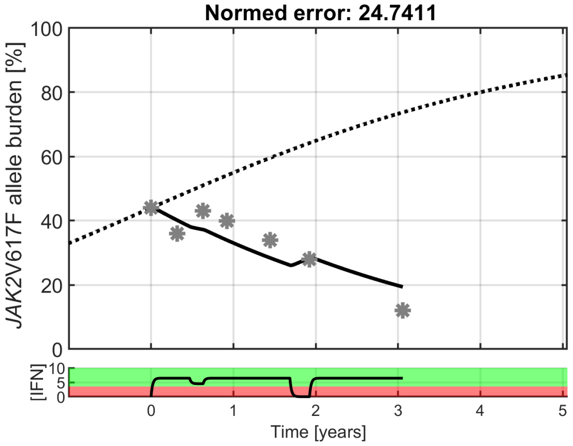

Patient P122

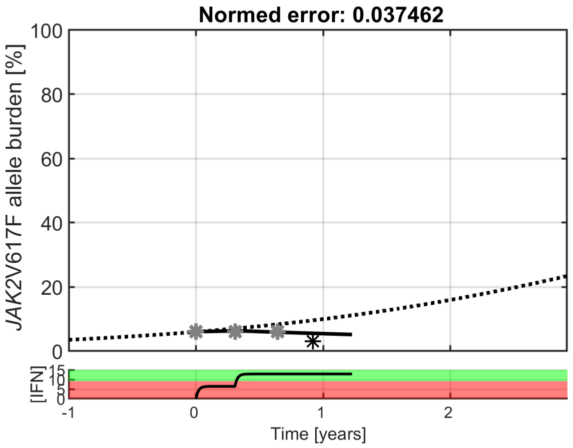

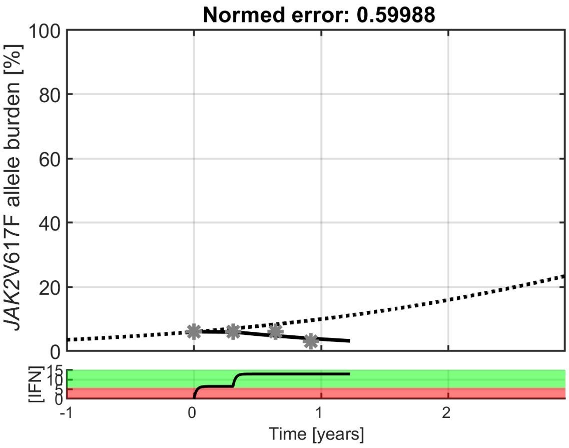

Patient P124

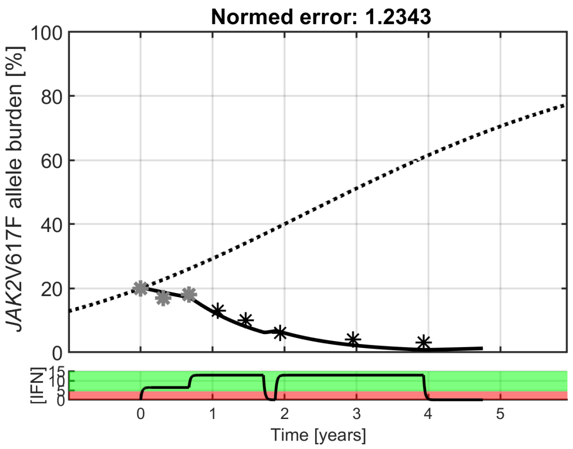

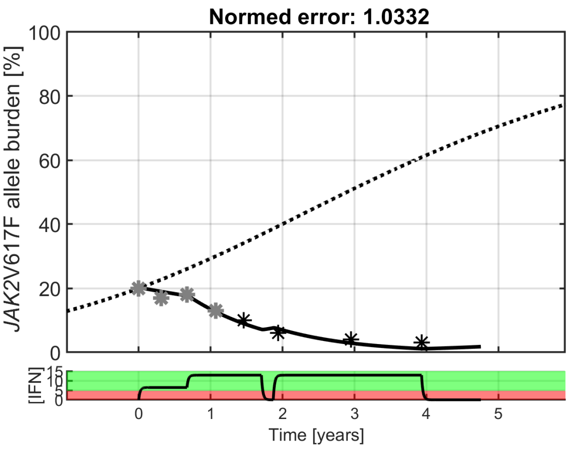

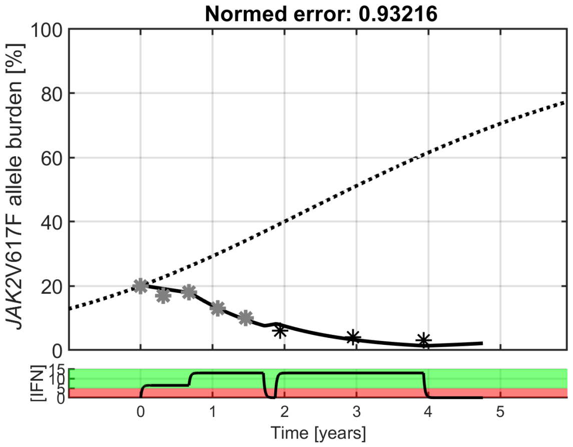

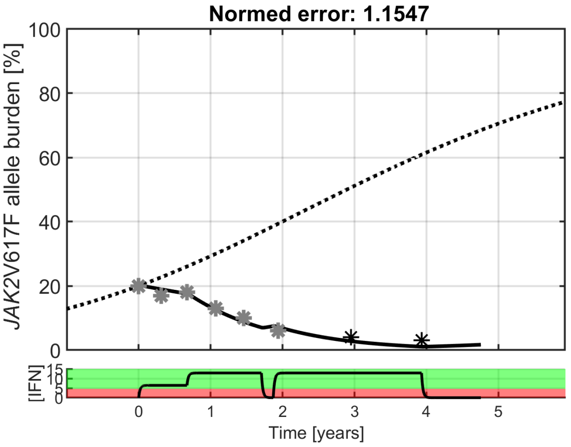

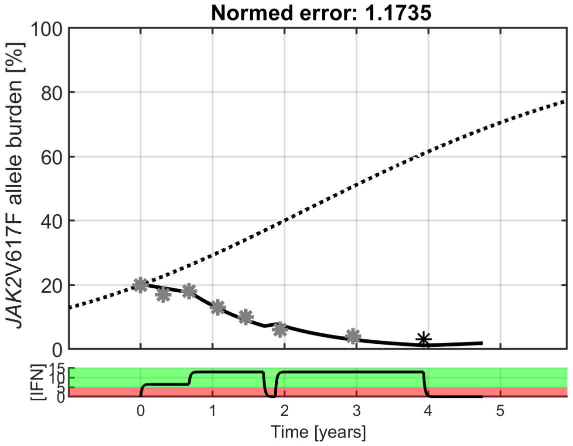

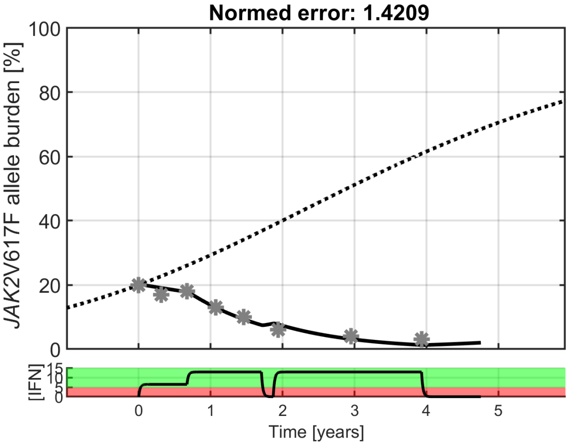

Patient P125

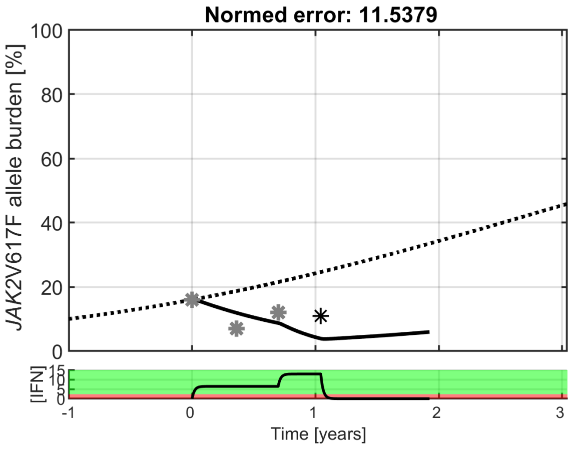

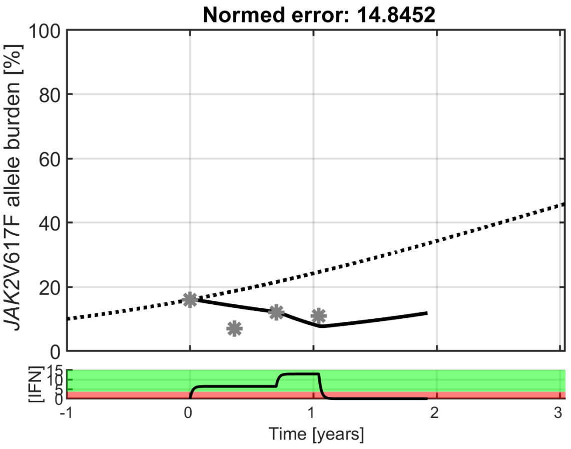

Patient P129

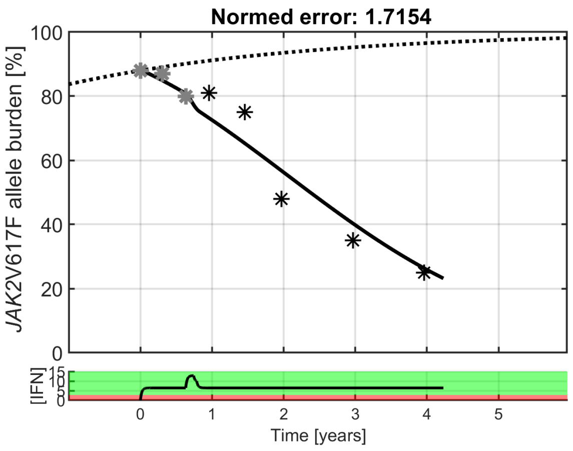

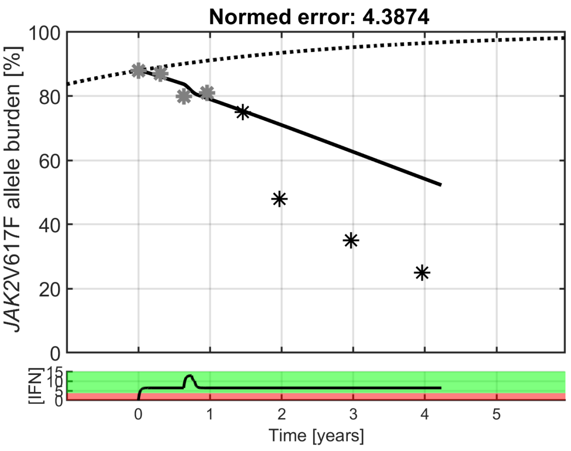

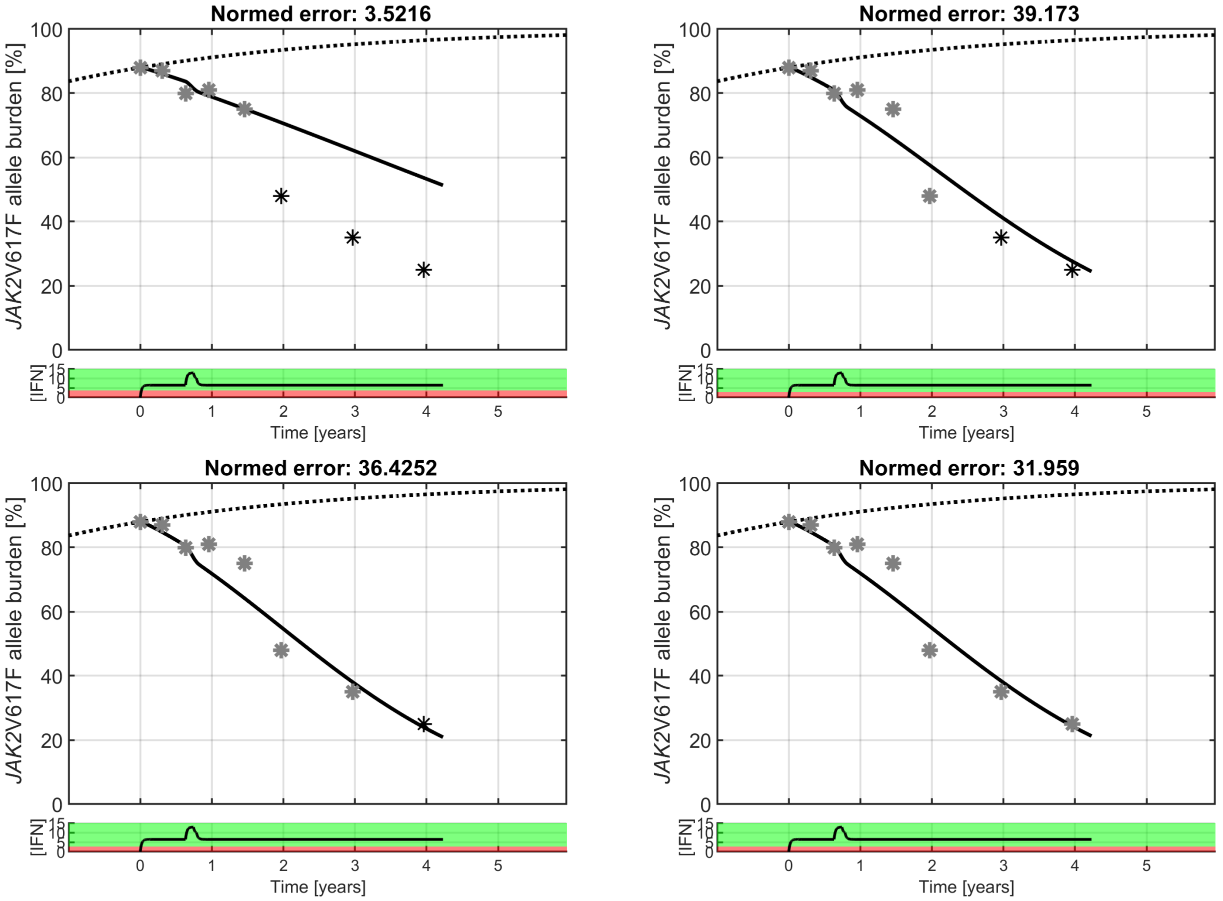

Patient P131

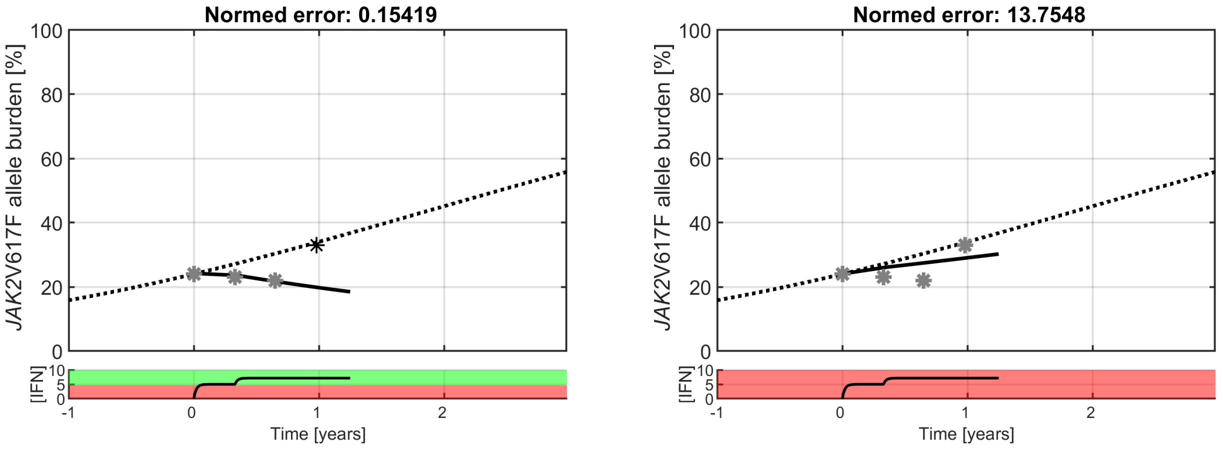

Patient P135

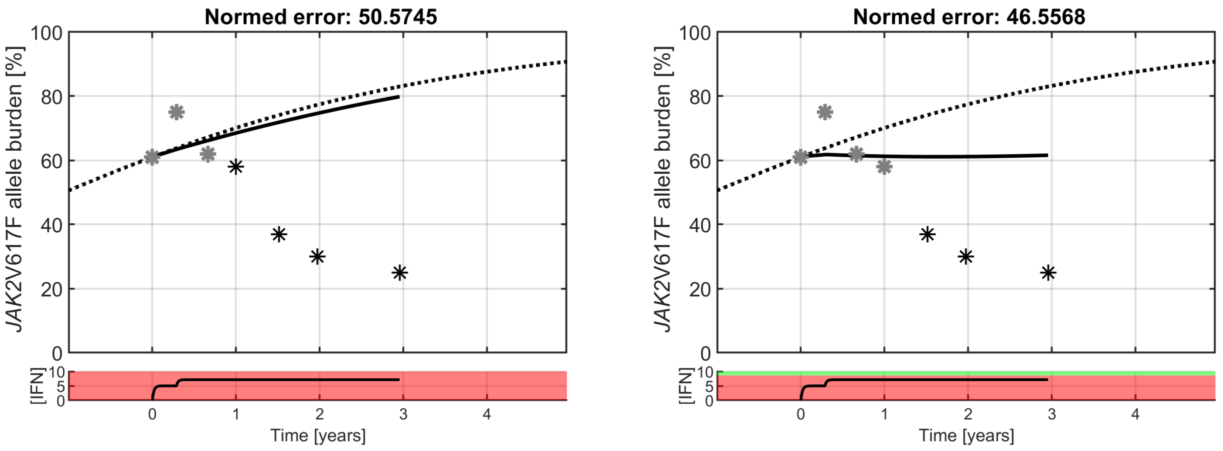

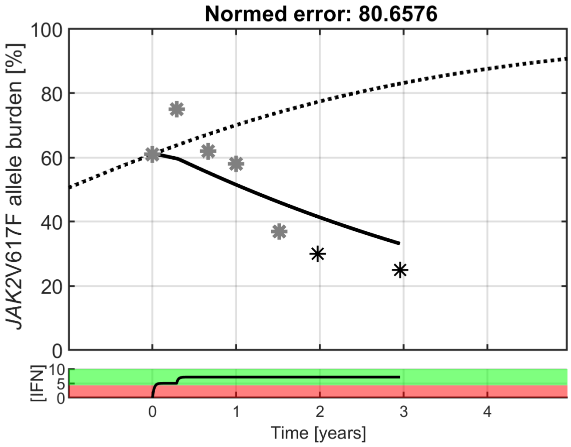

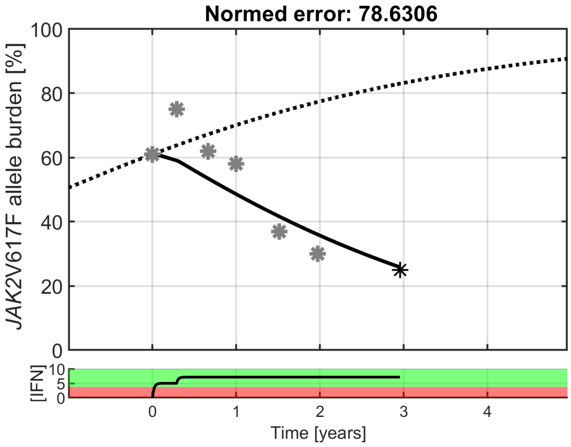

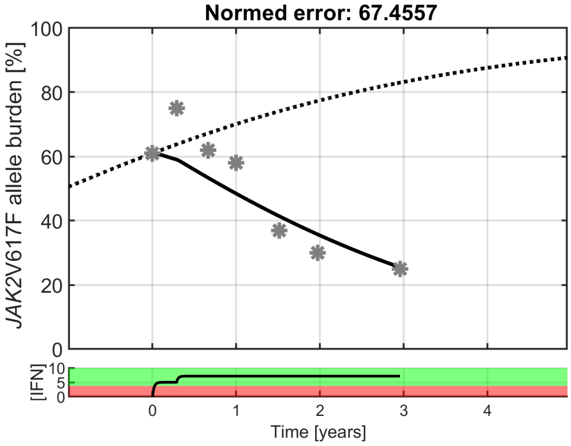

Patient P139

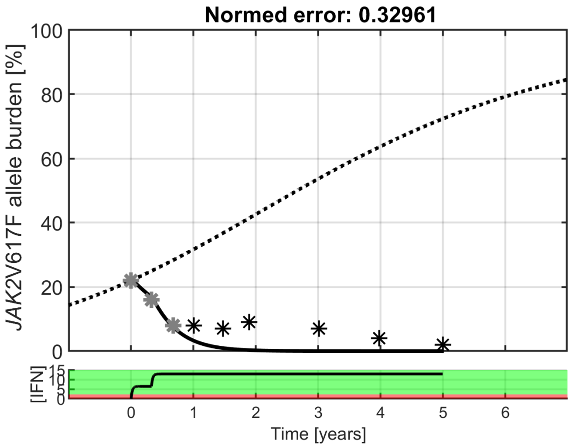

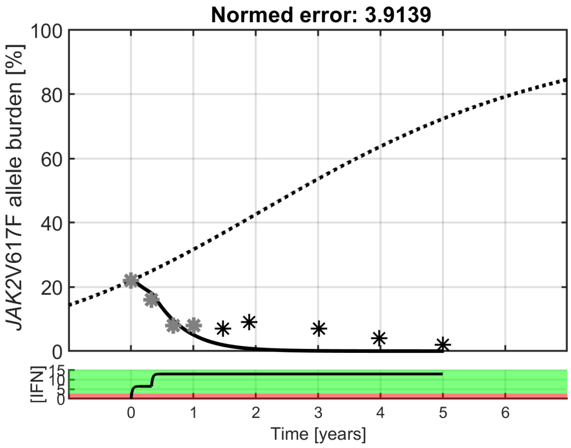

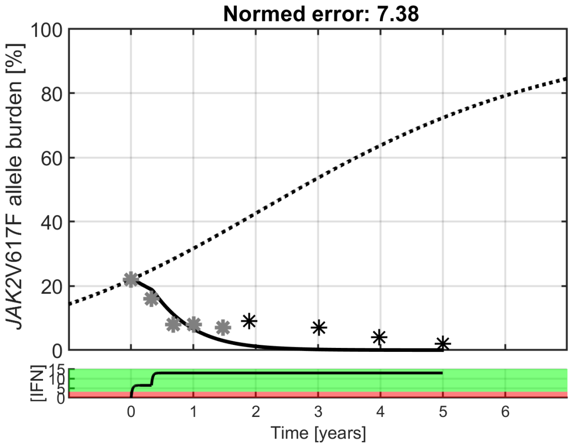

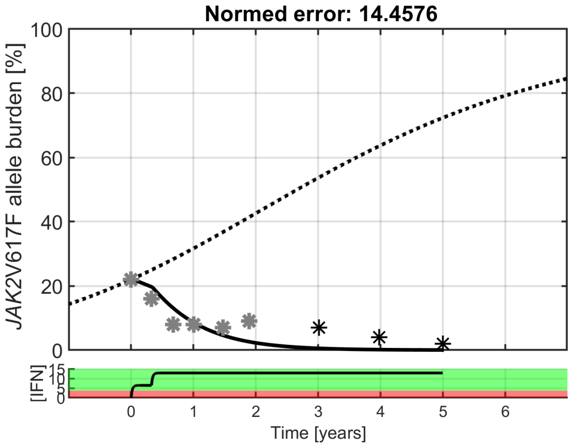

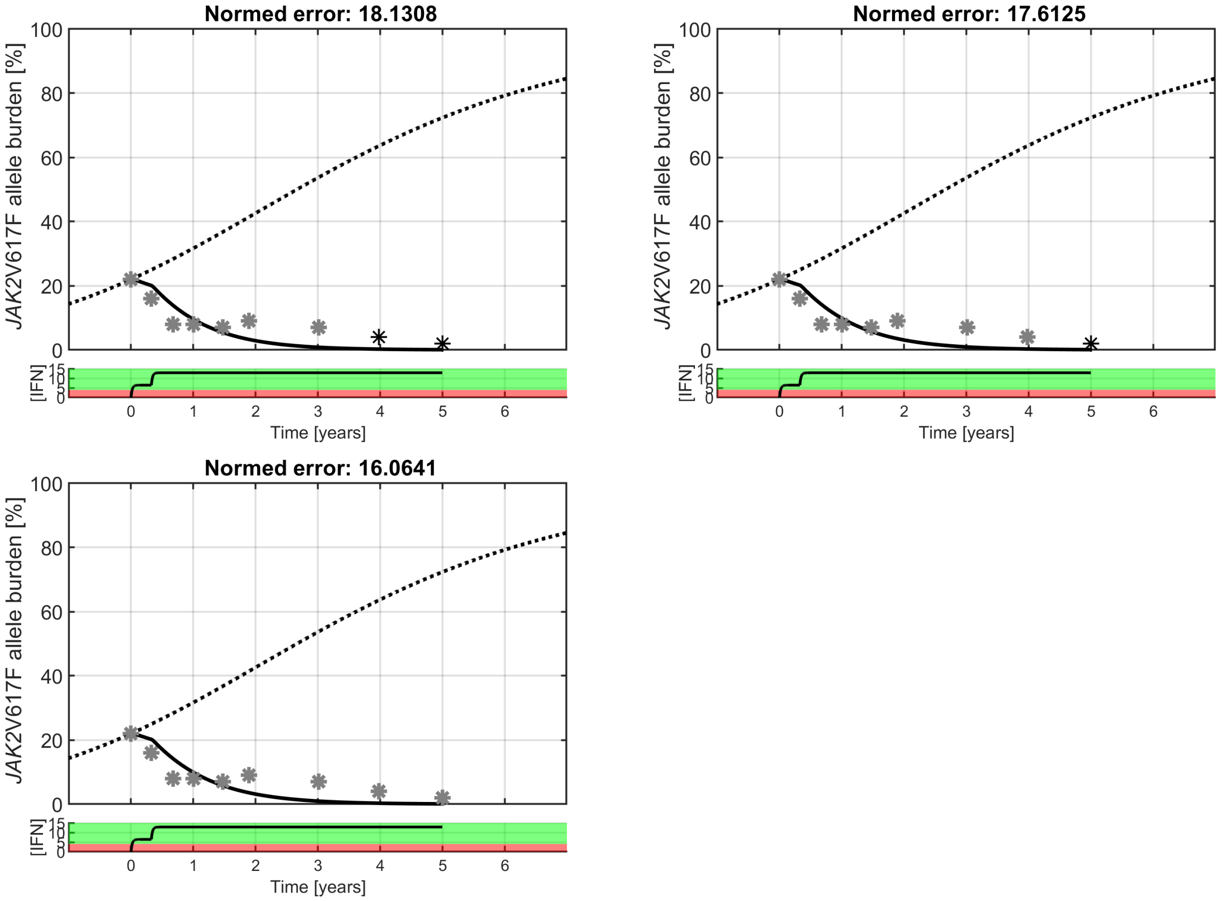

Patient P140

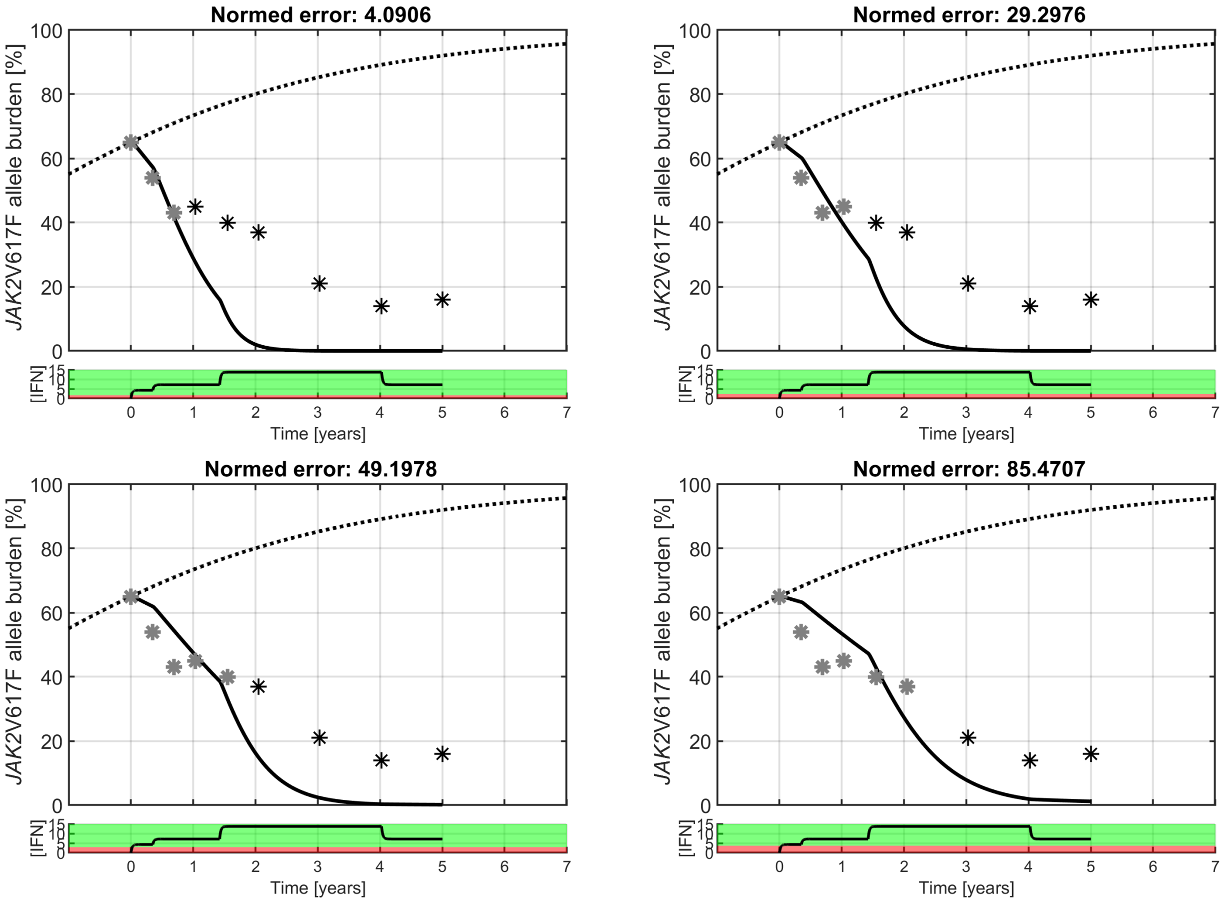

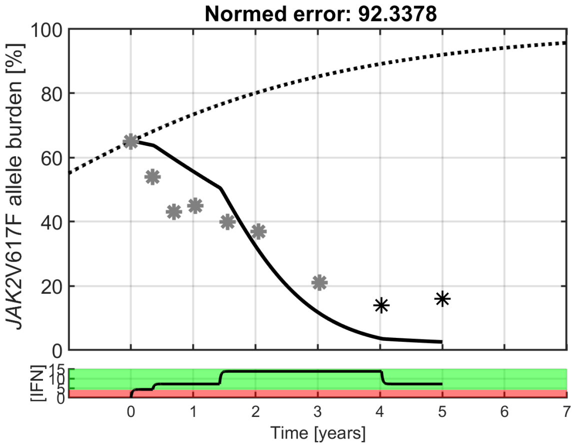

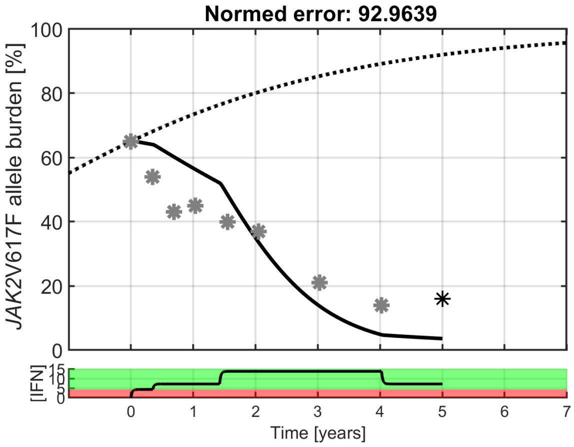

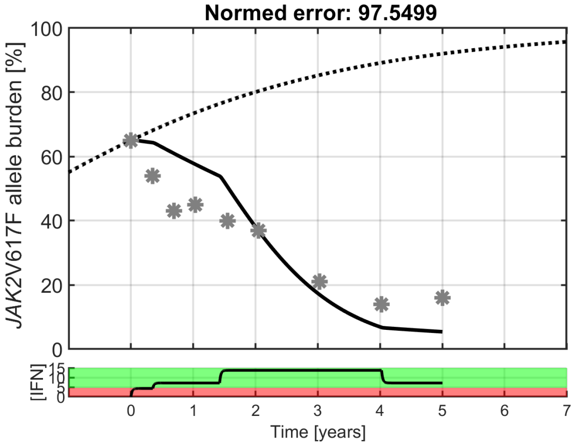

Patient P142

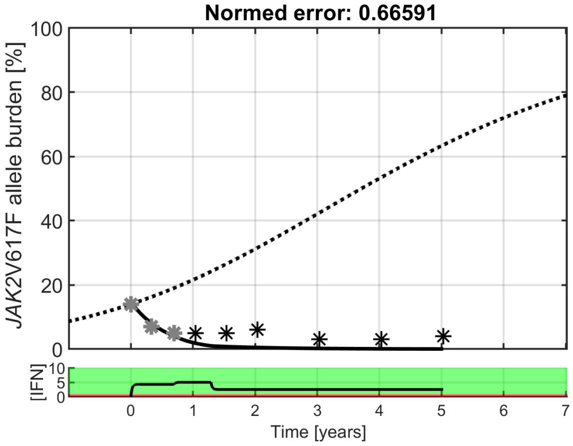

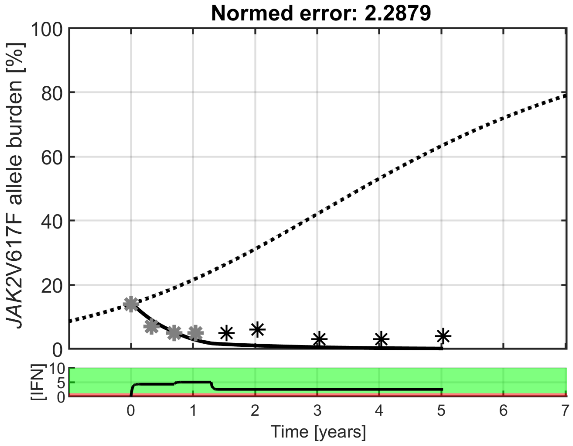

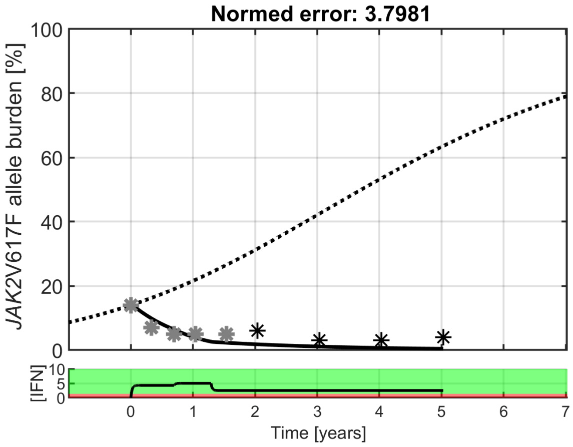

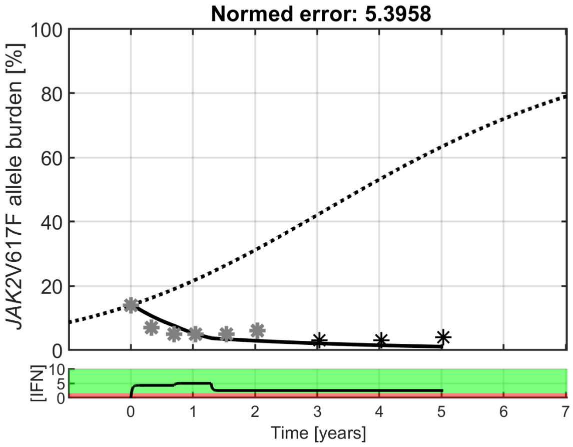

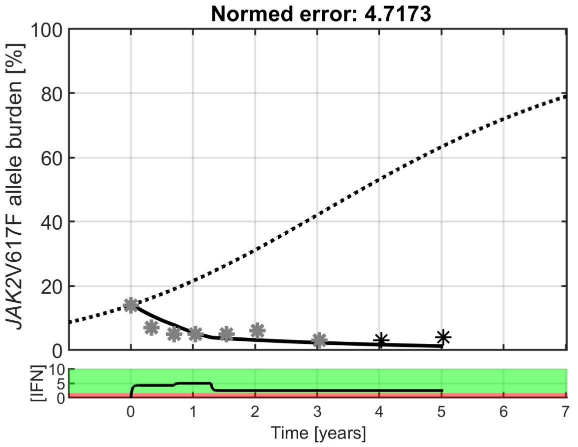

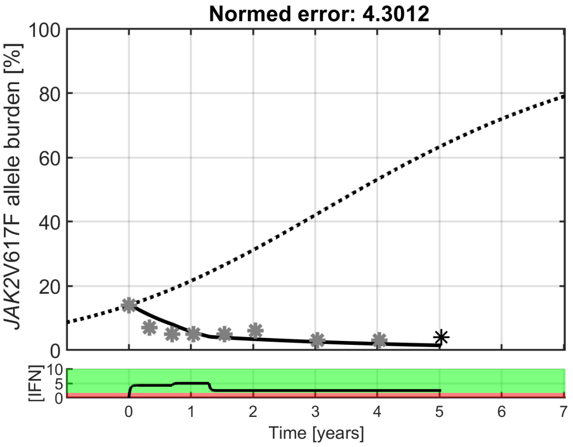

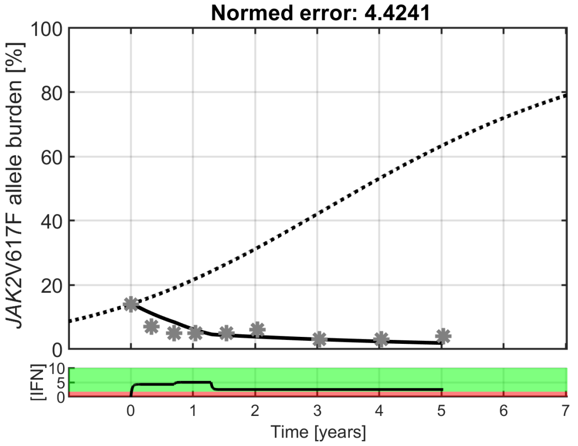

Patient P144

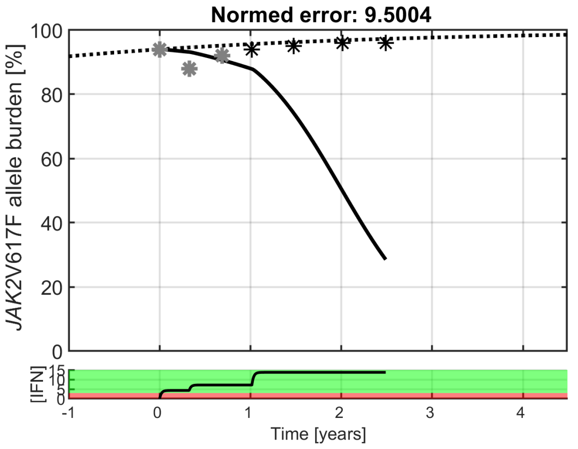

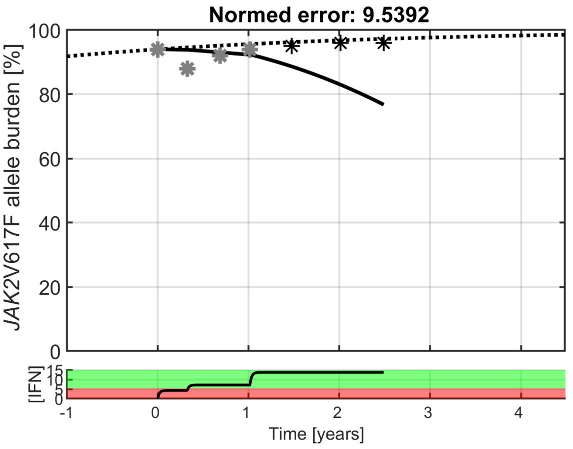

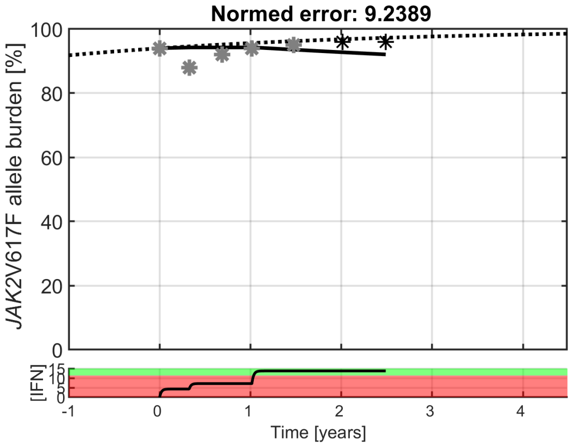

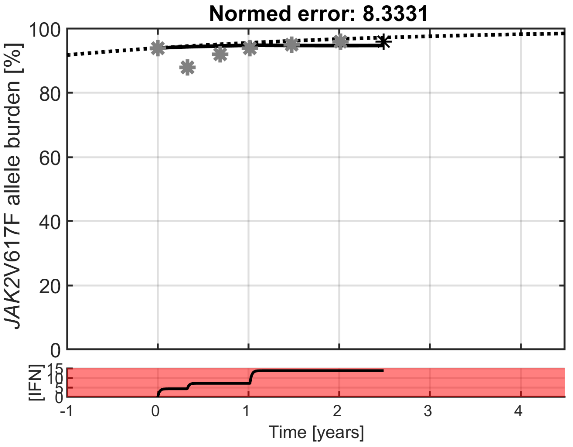

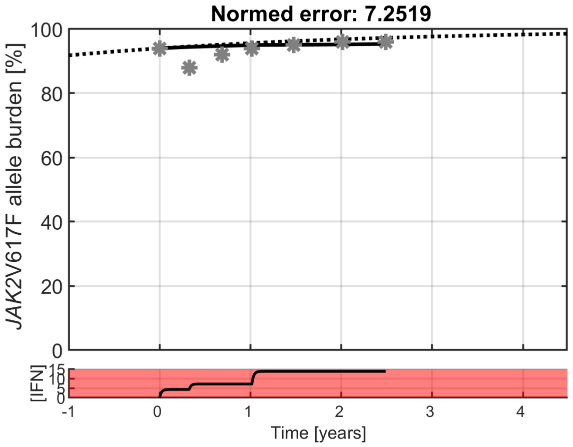

Patient P145

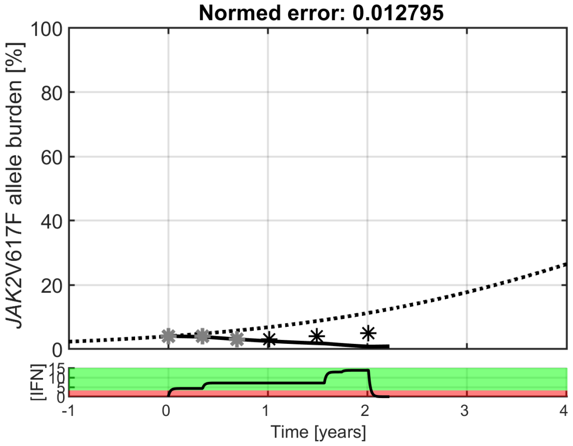

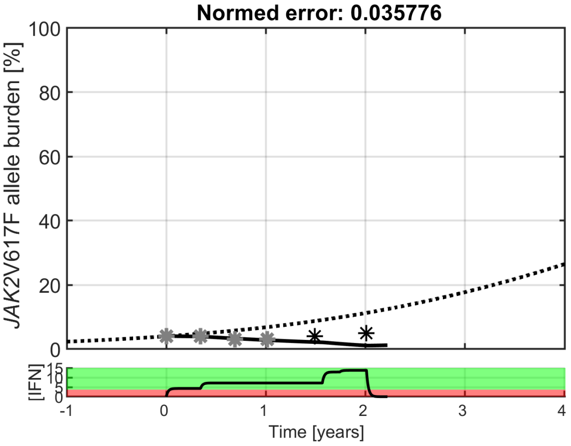

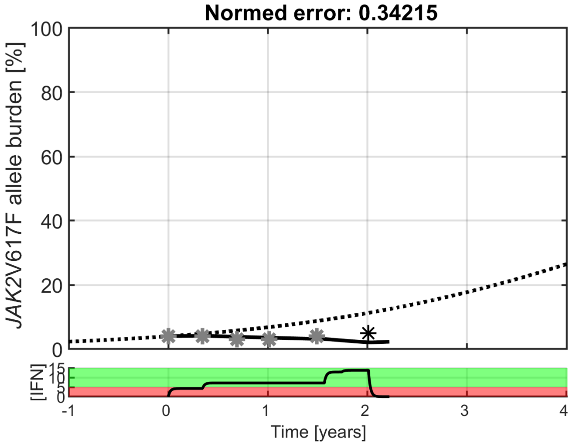

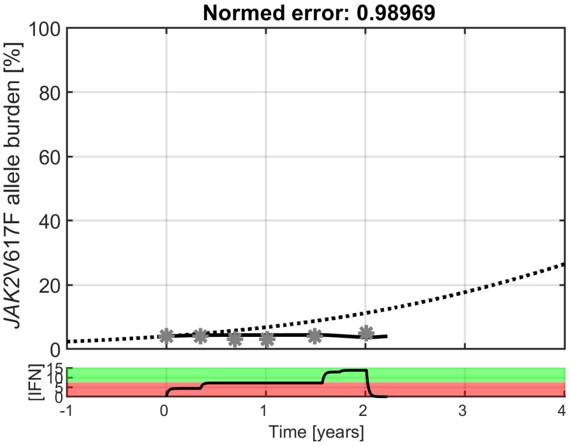

Patient P148

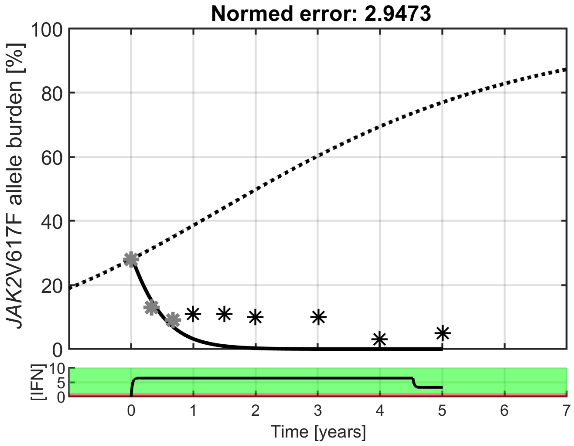

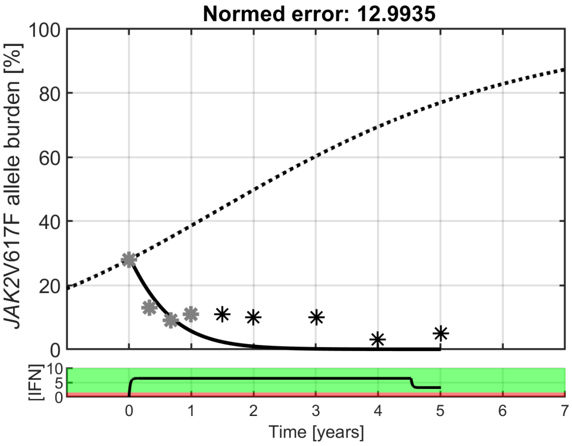

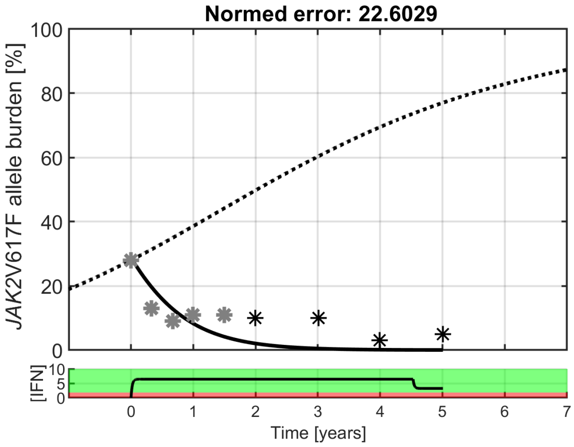

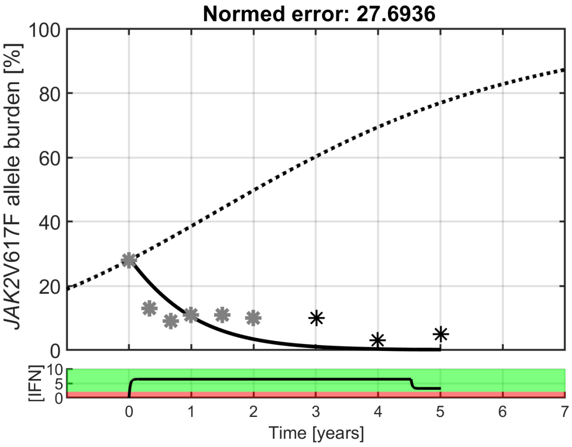

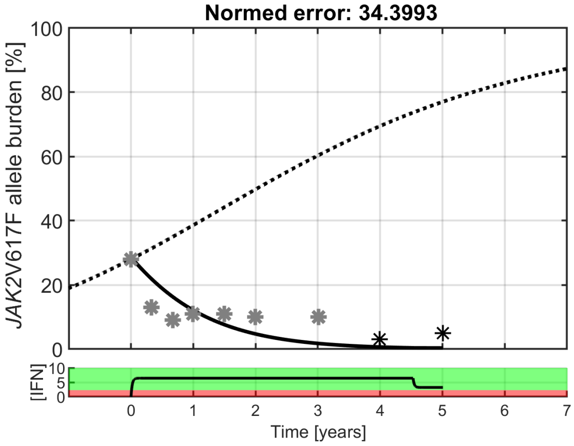

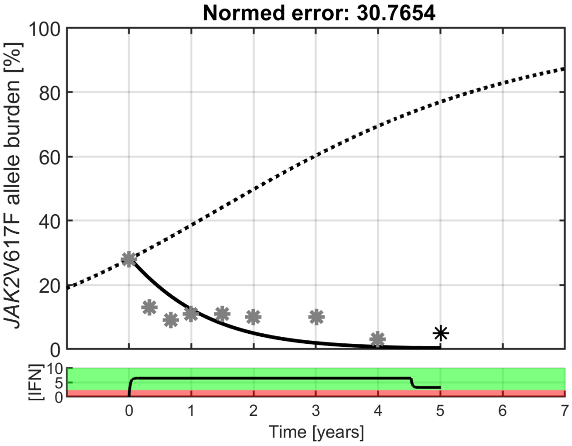

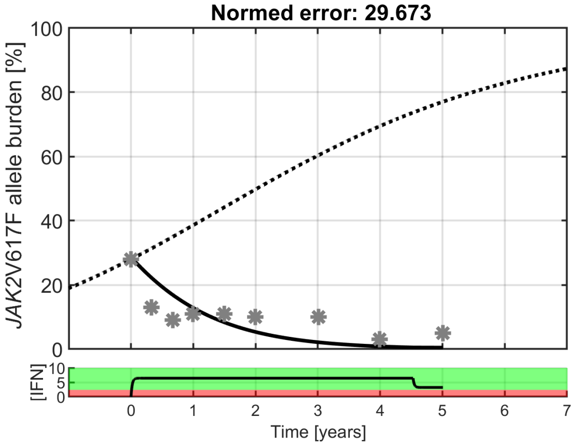

Patient P150

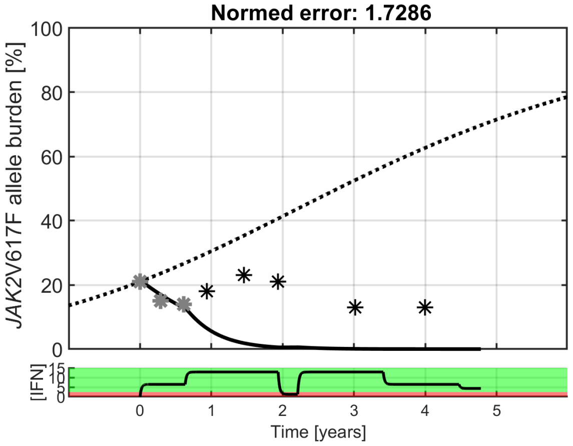

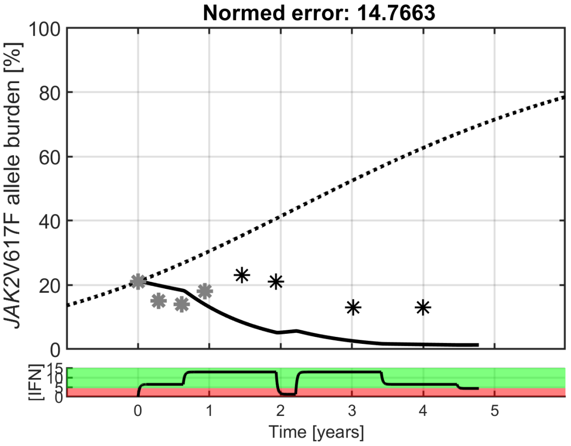

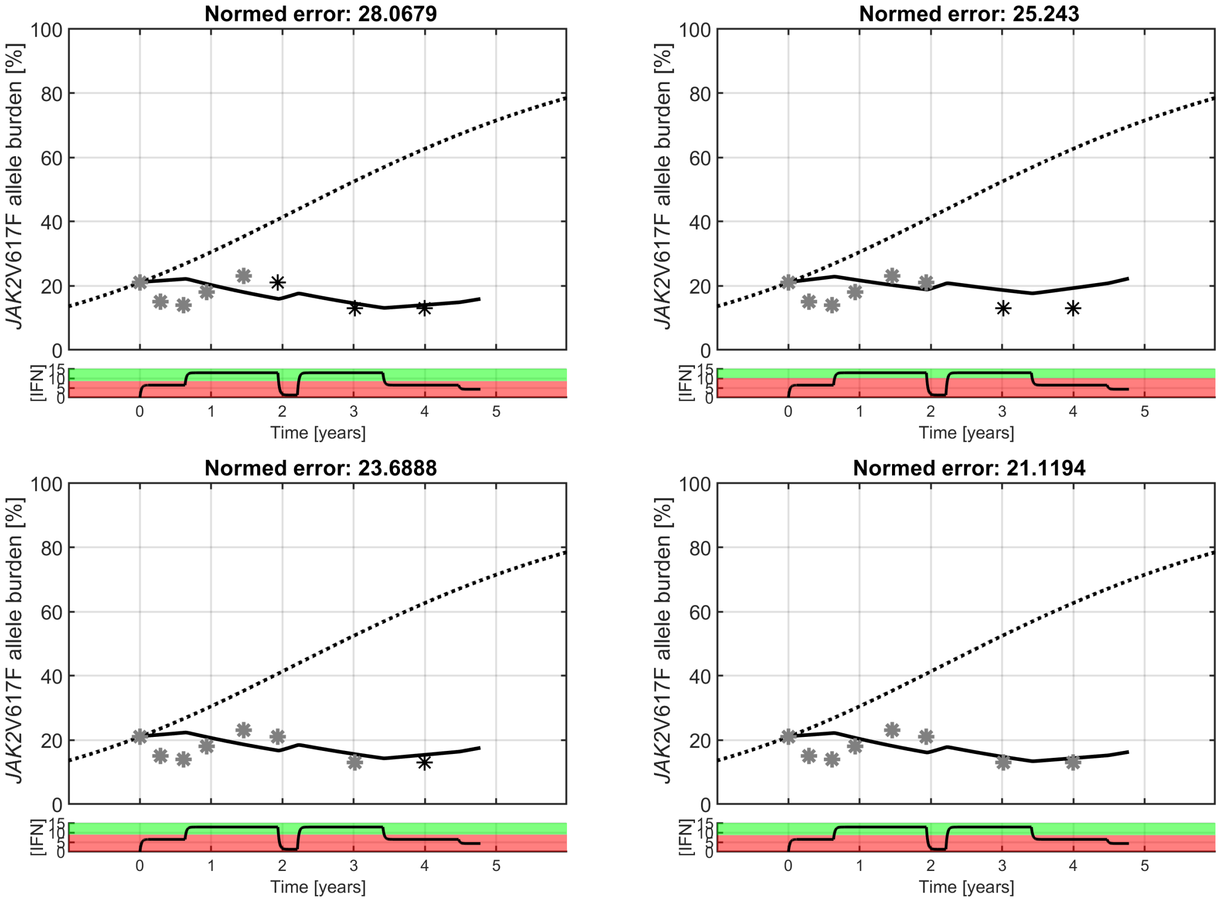

Patient P152

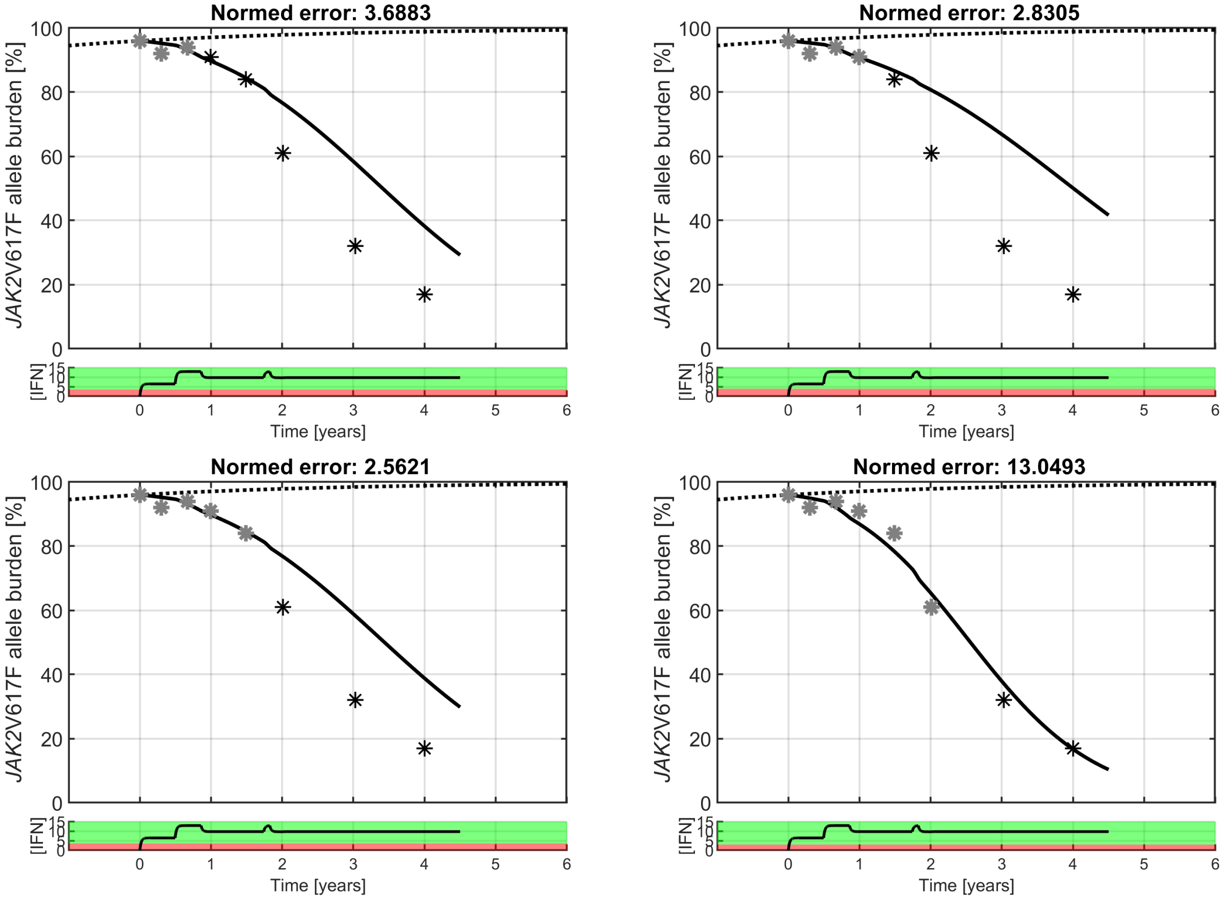

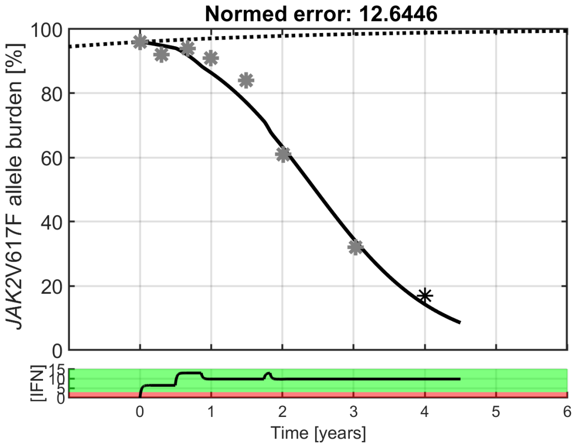

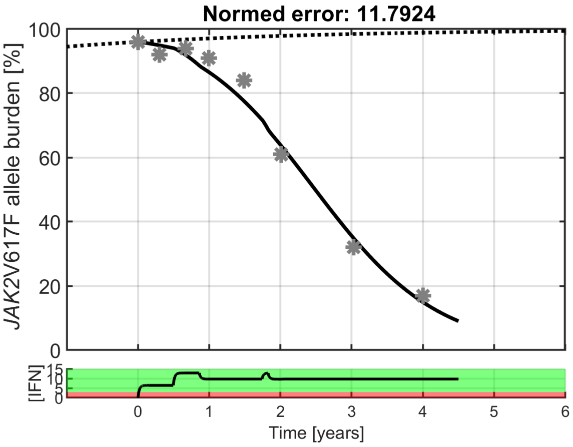

Patient P156

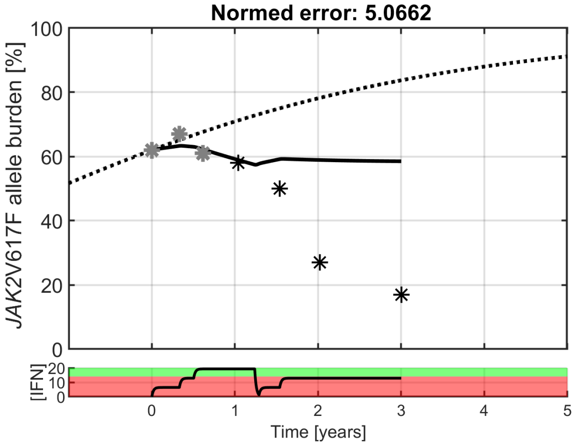

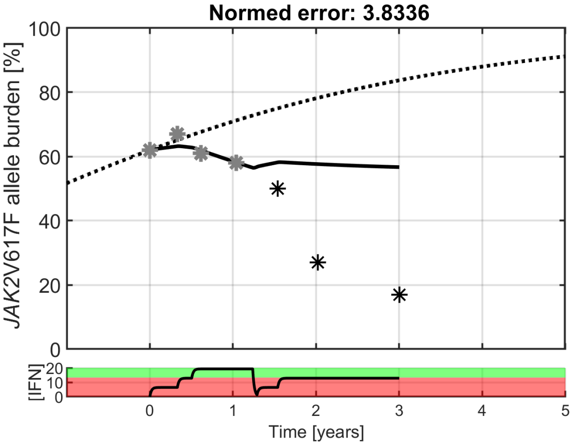

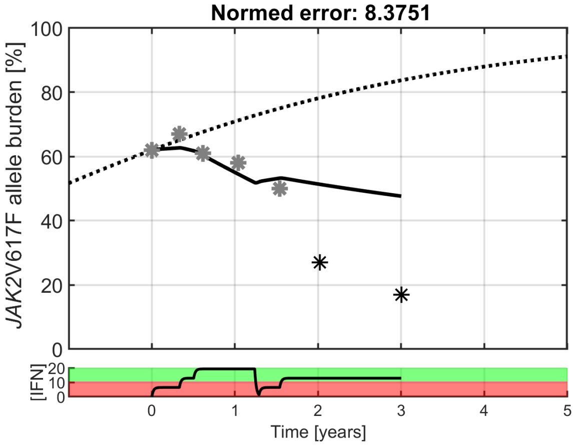

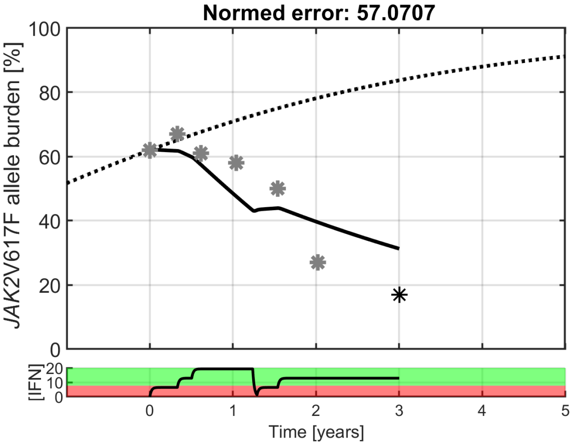

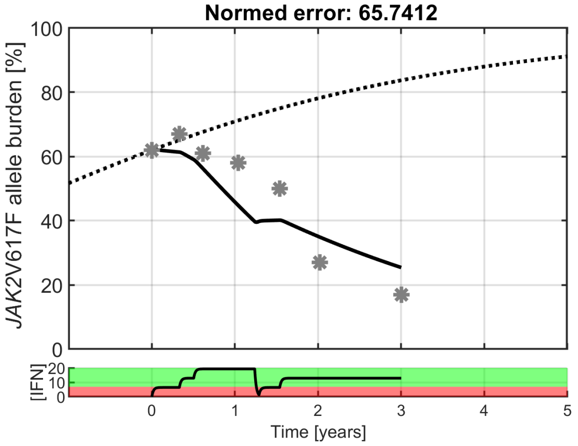

Patient P158

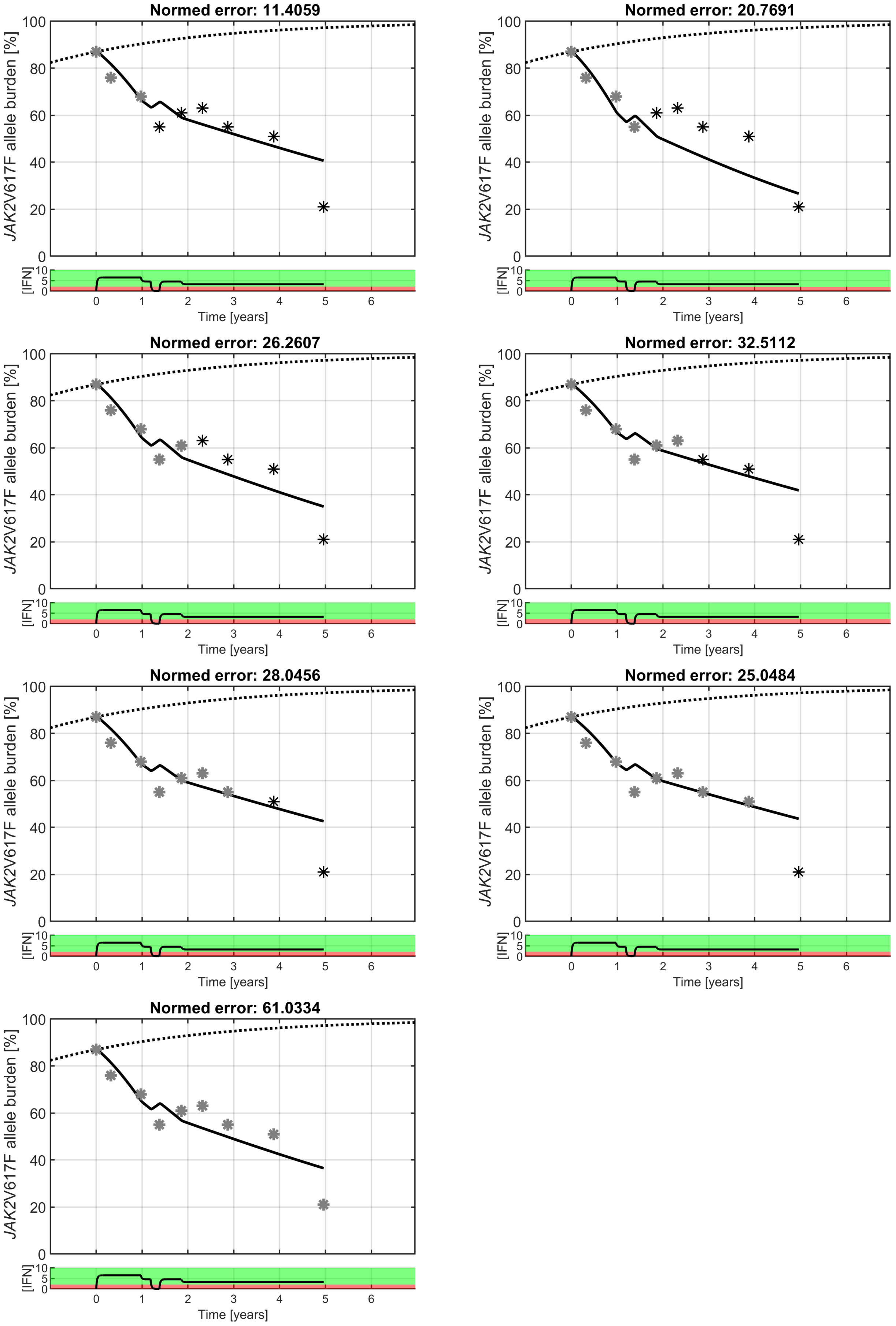

Patient P162

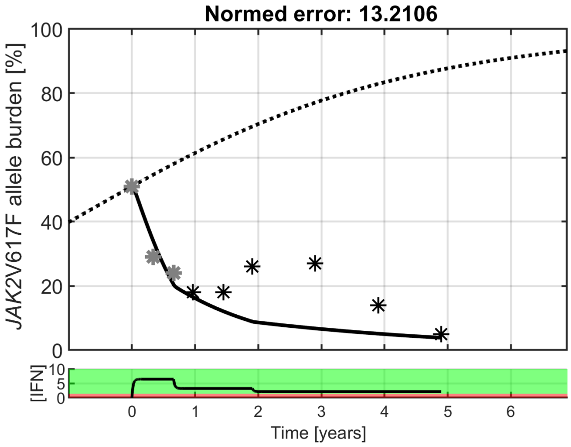

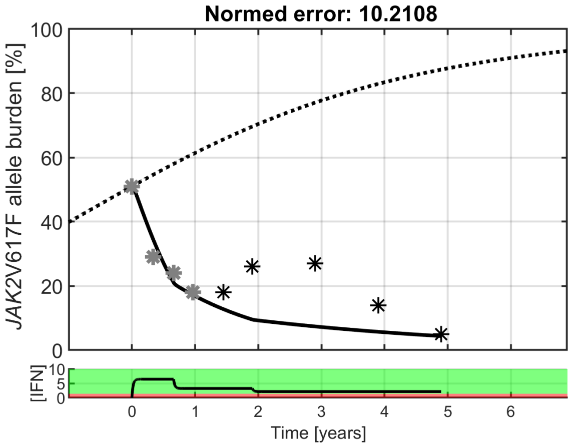

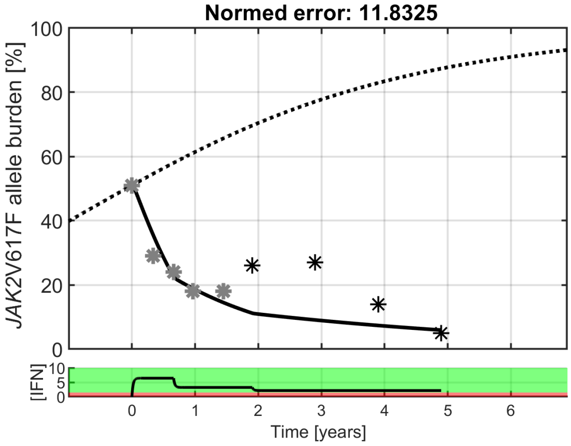

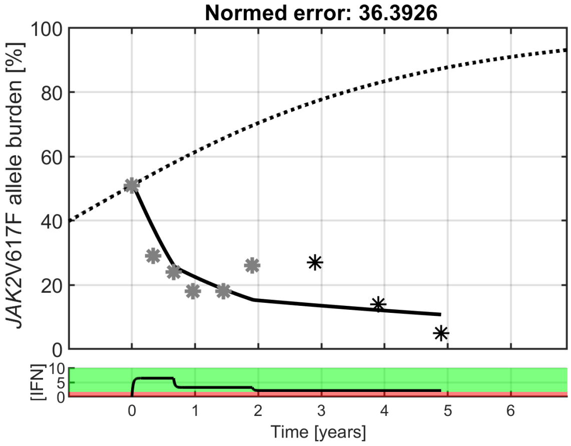

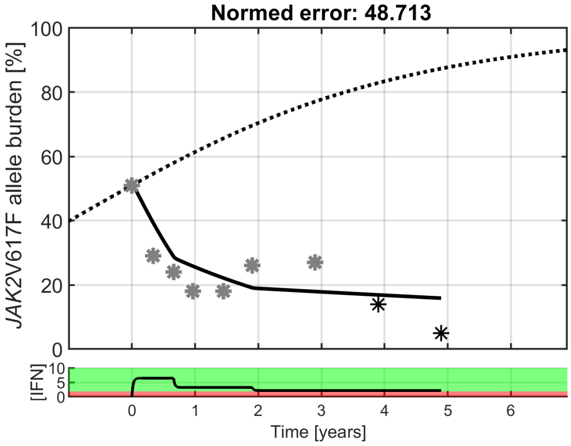

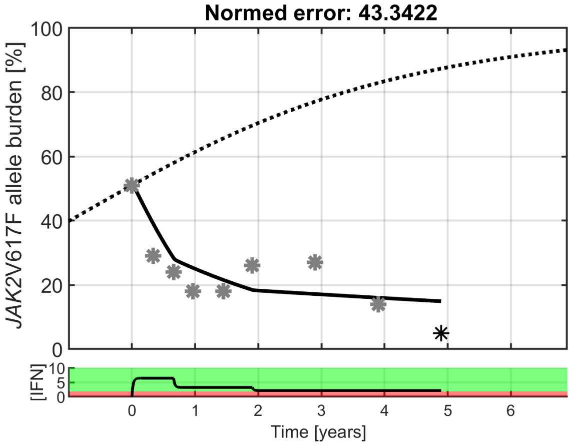

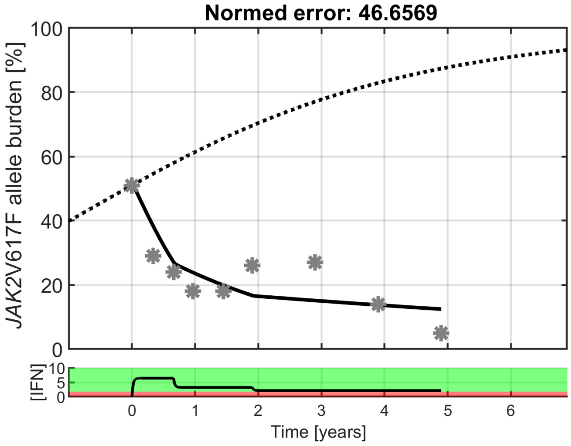

Patient P163

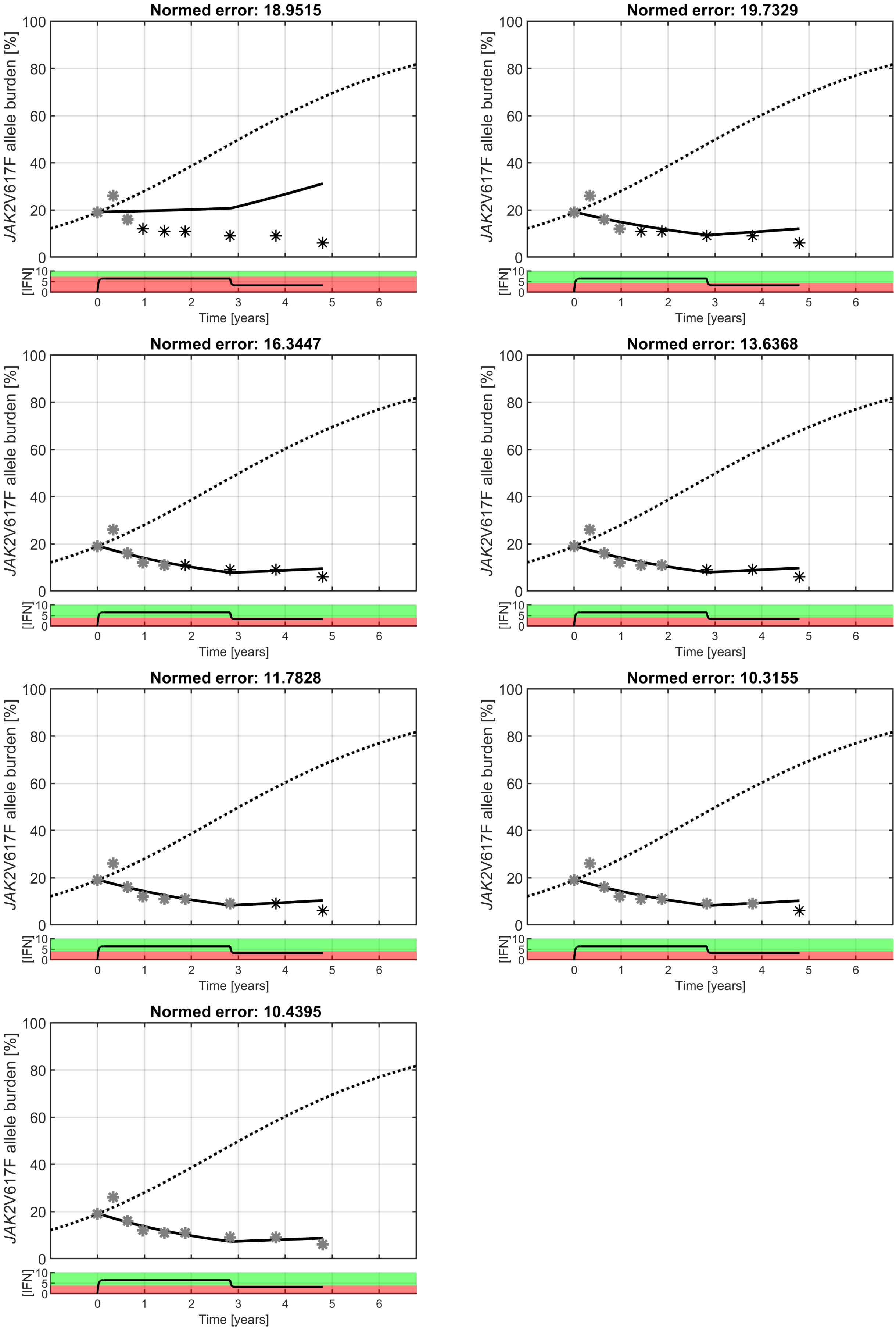

Patient P164

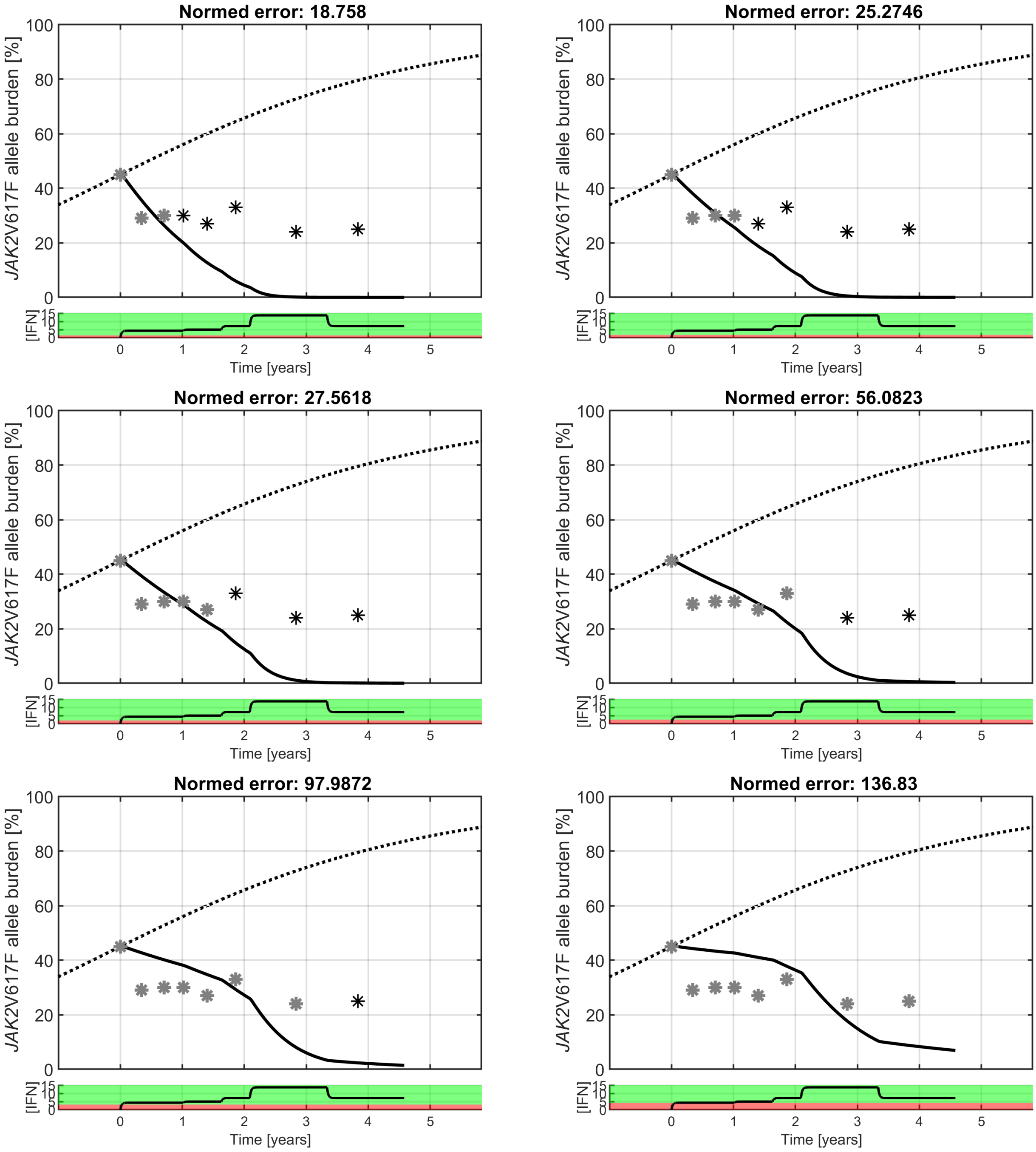

Patient P167

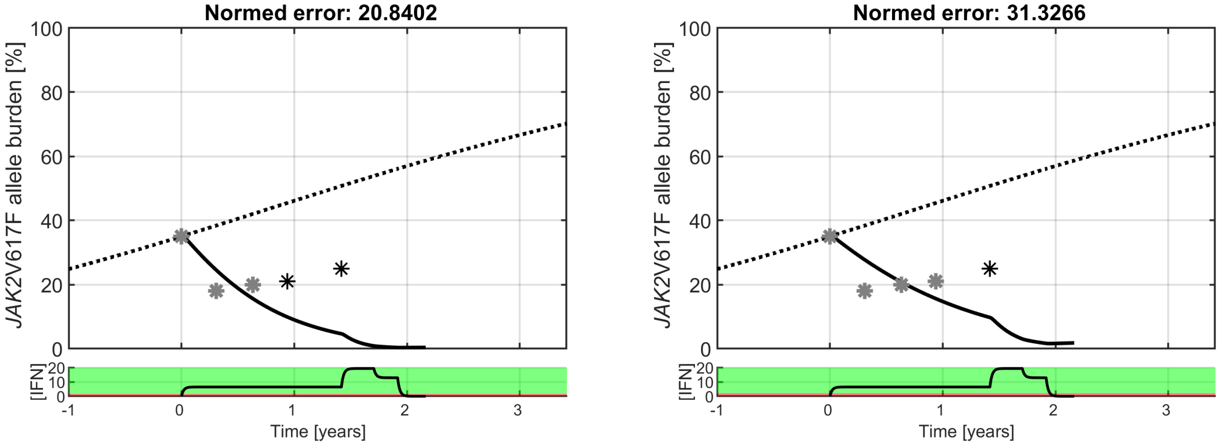

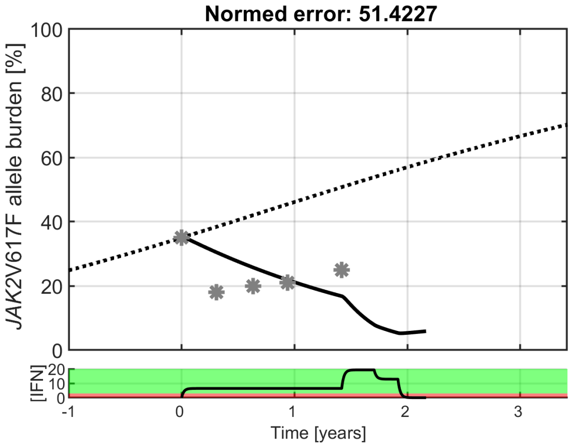

Patient P184

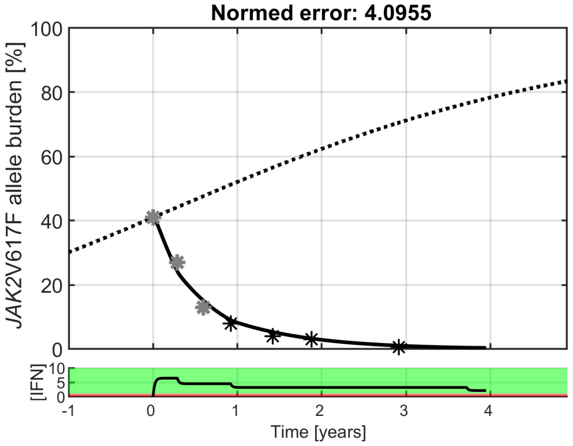

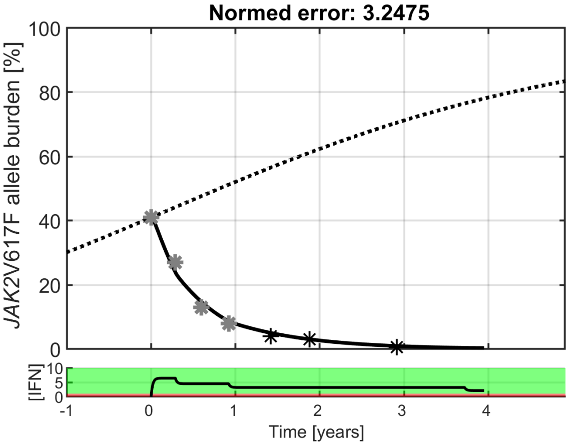

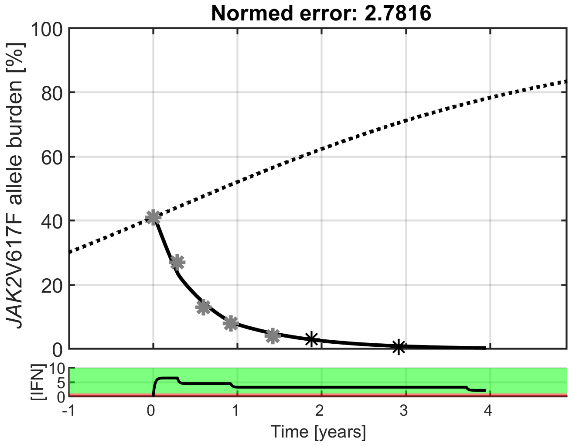

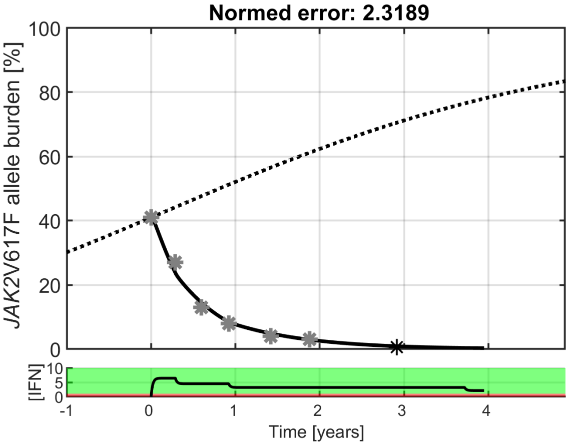

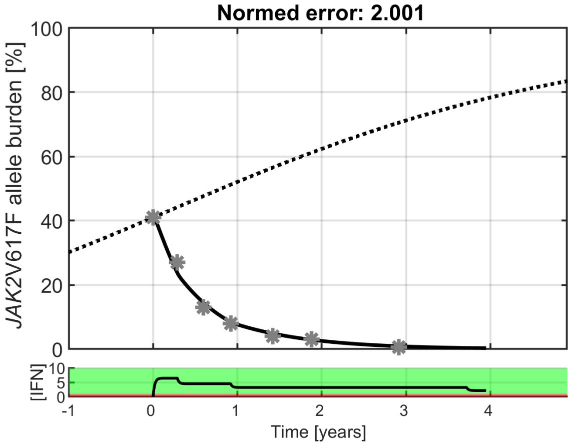

Patient P187

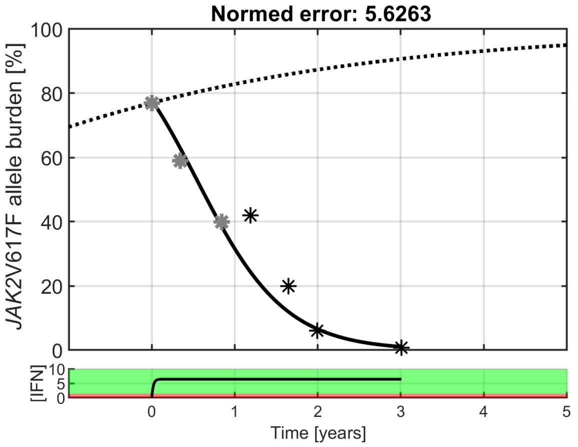

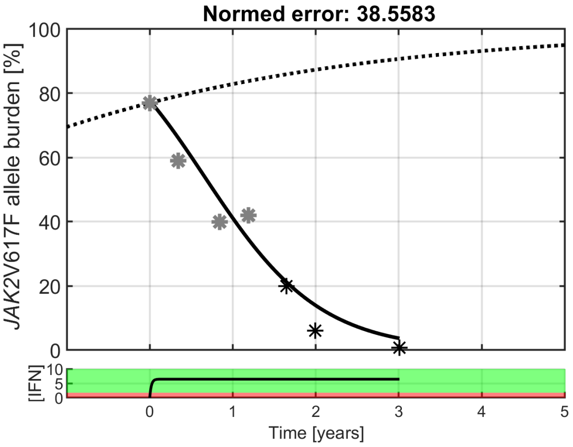

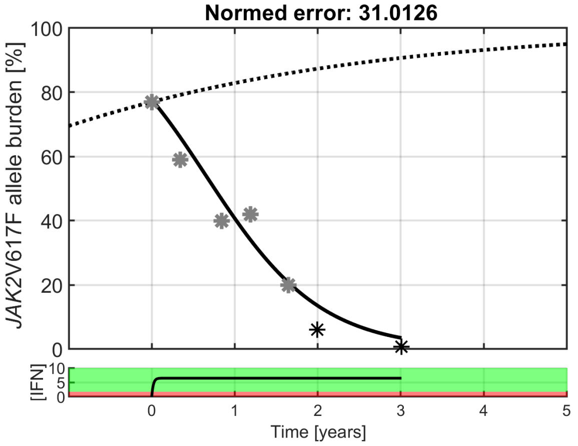

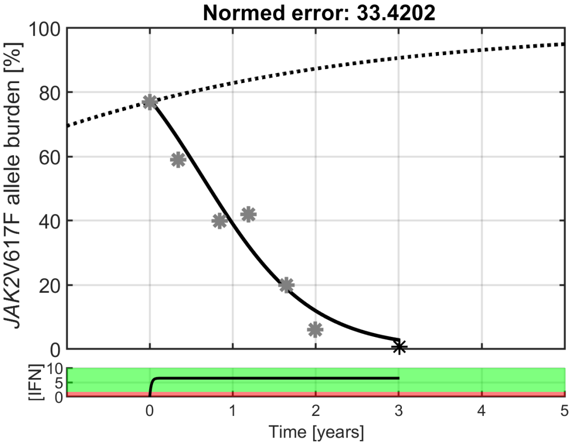

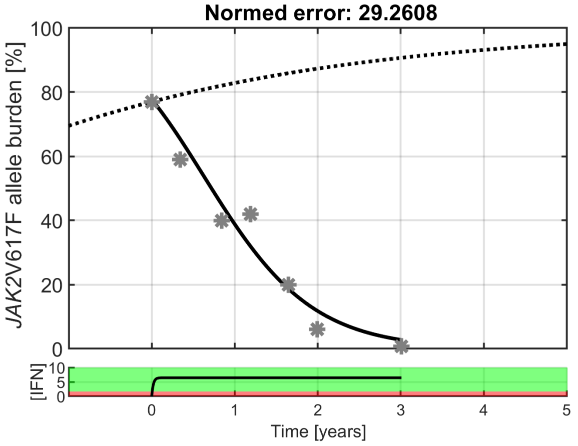

Patient P190

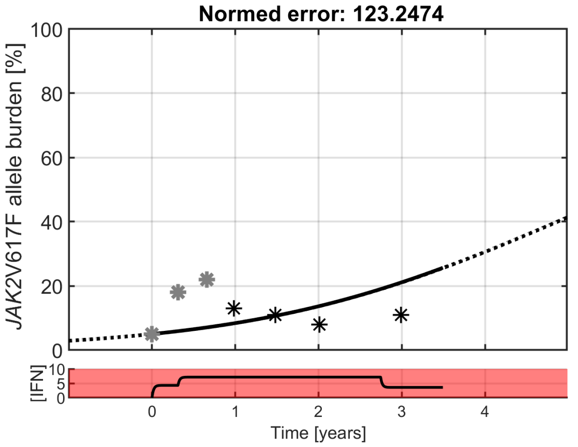

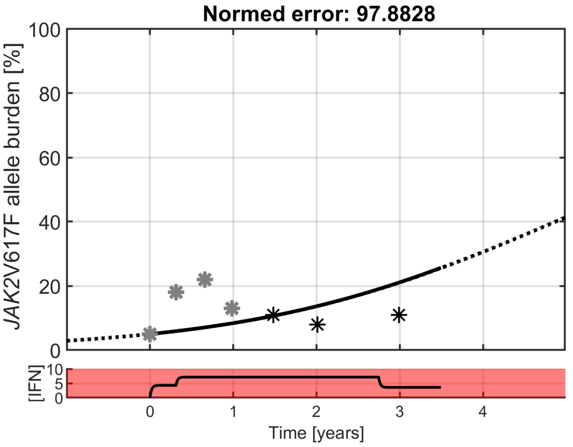

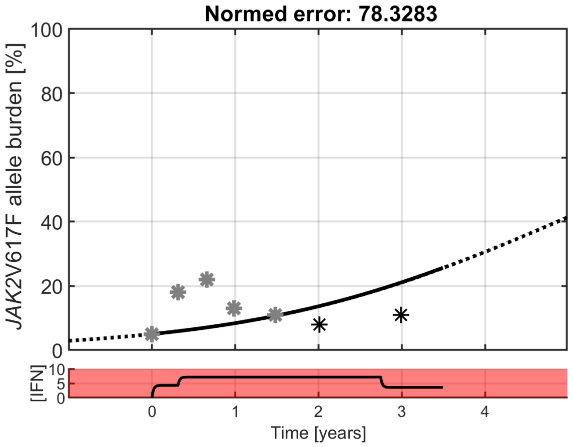

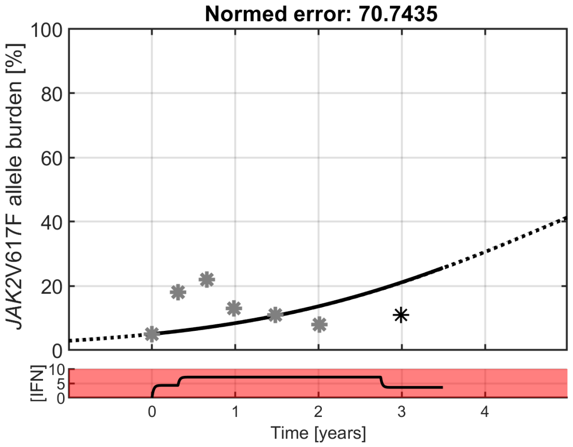

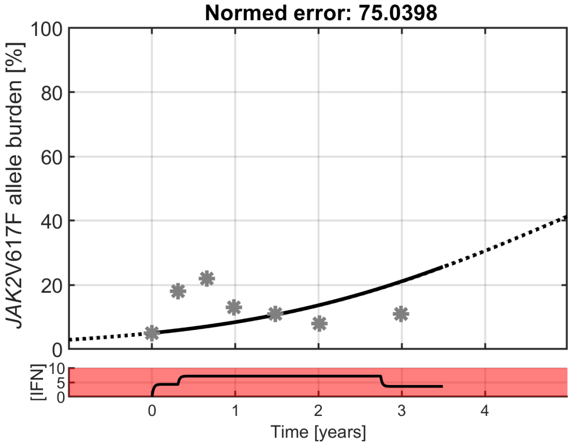

Patient P196

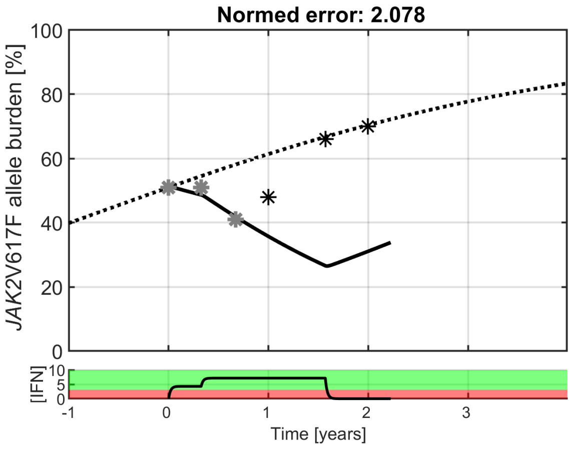

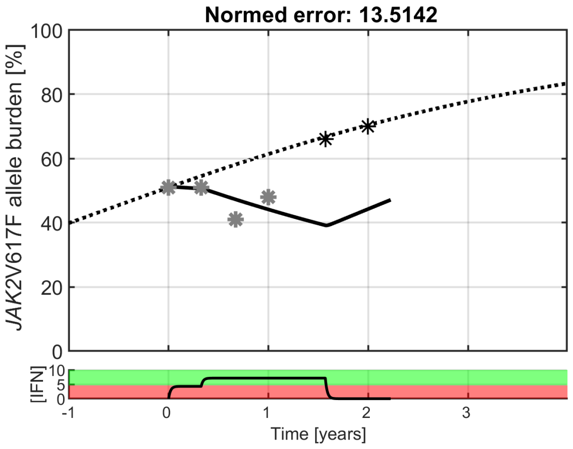

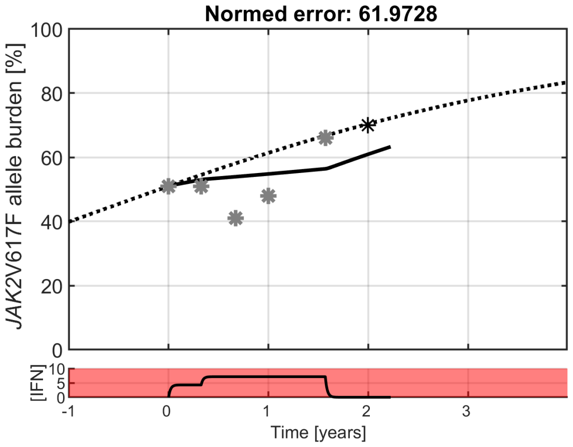

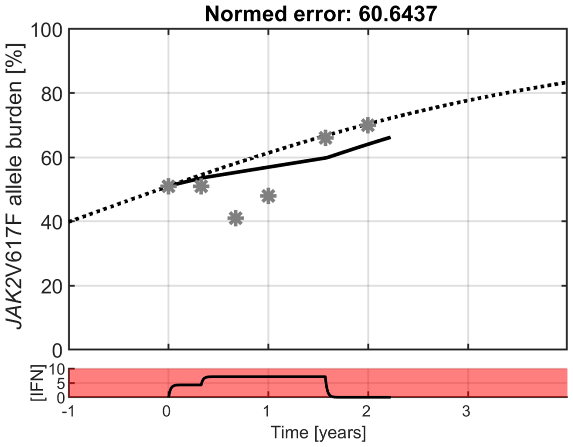

Patient P197

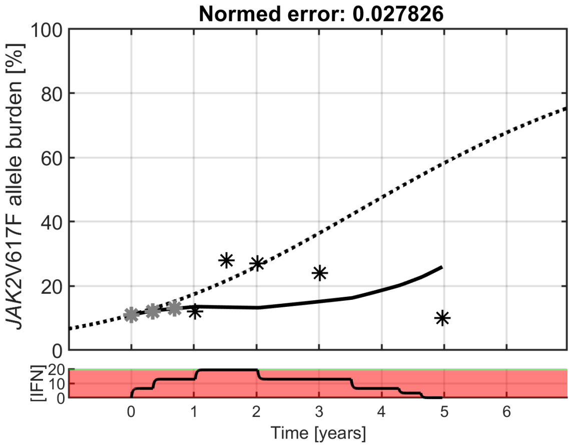

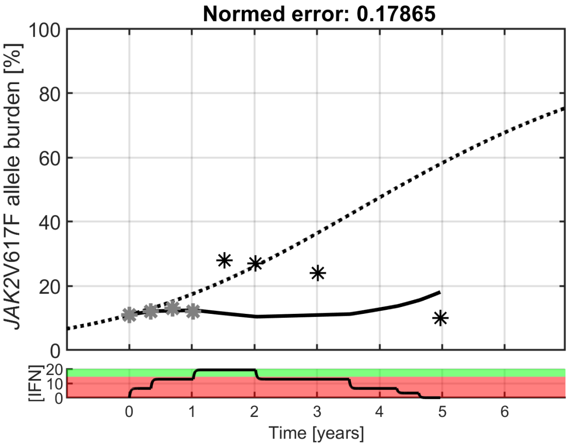

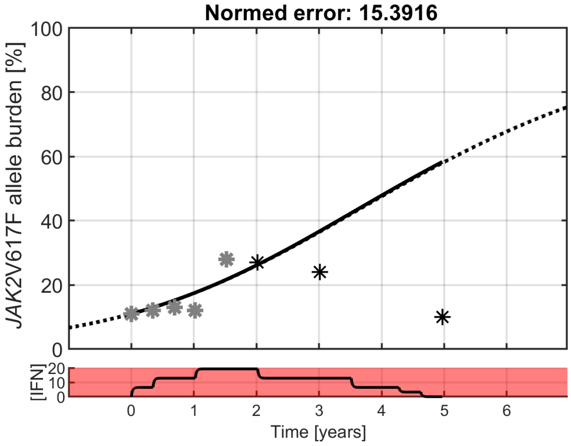

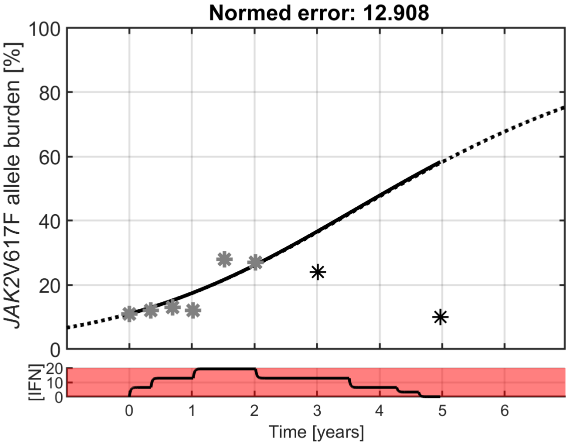

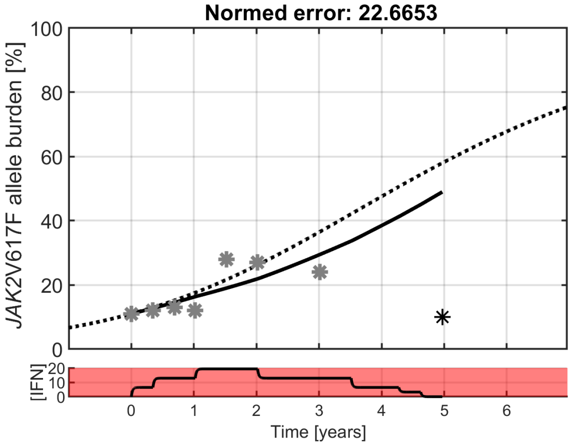

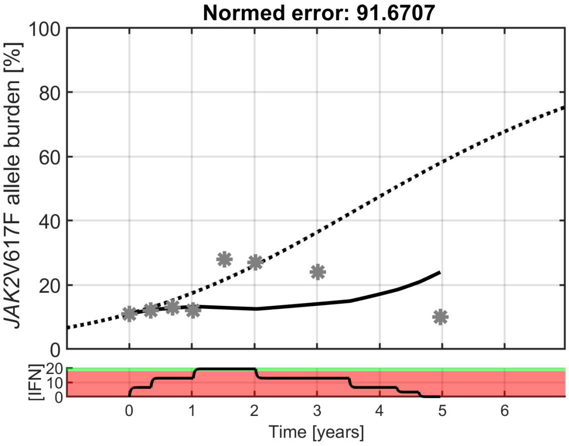

Patient P198

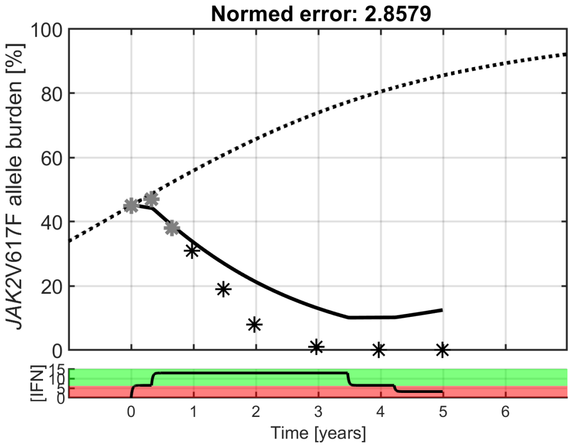

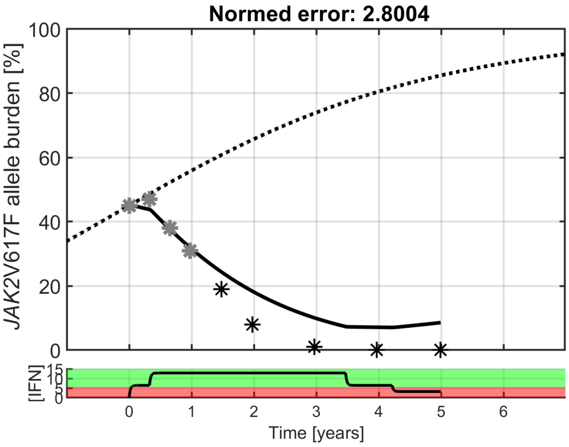

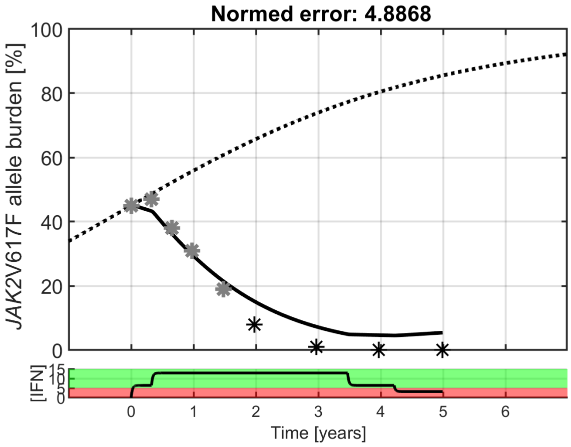

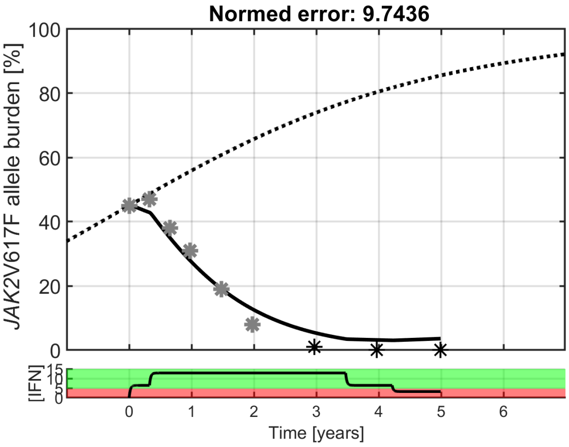

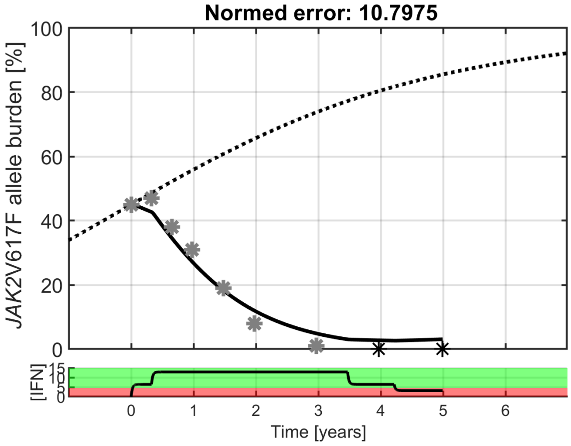

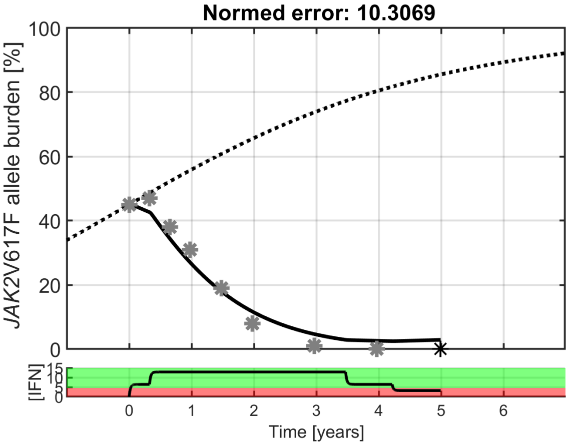

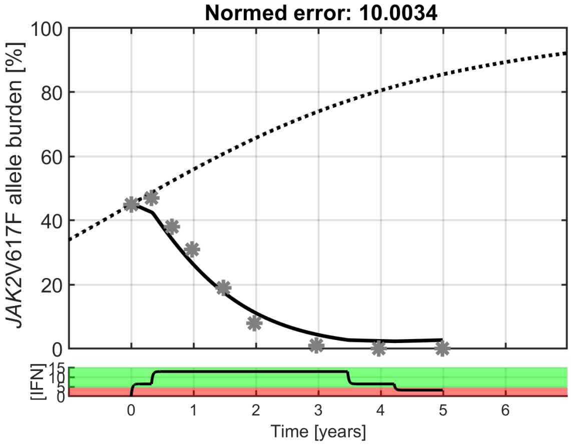

Patient P199

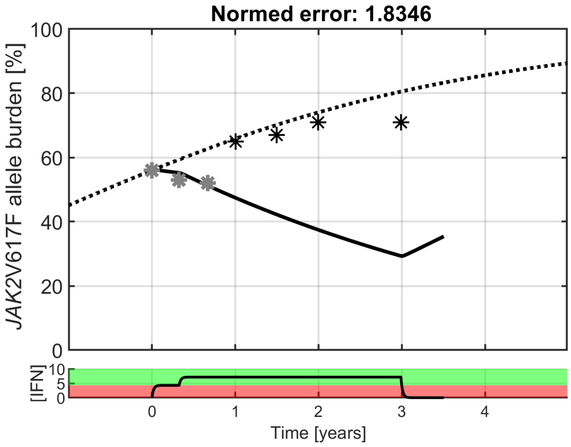

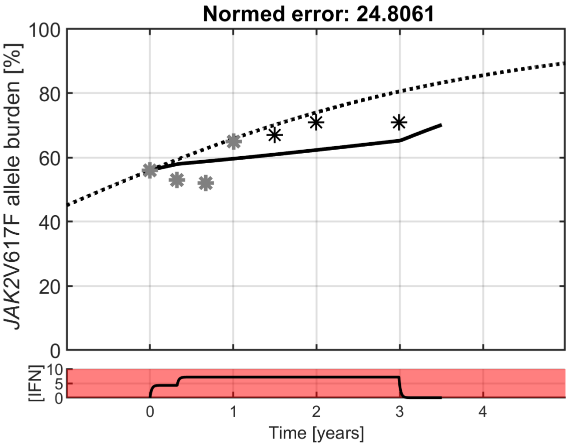

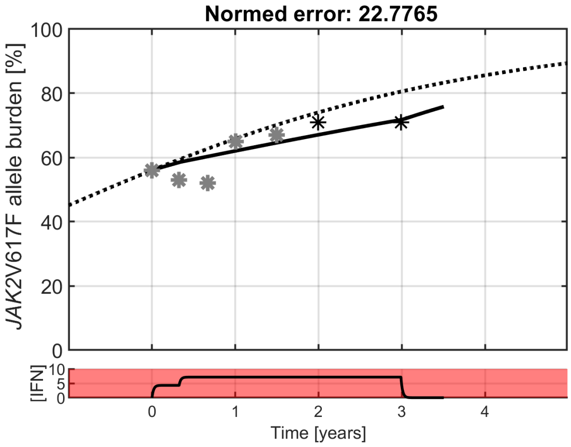

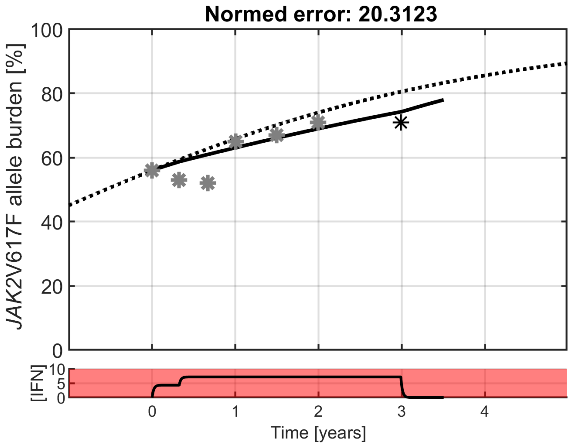

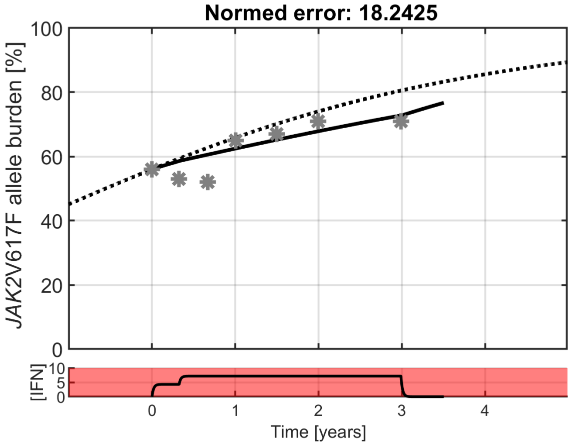

Patient P201

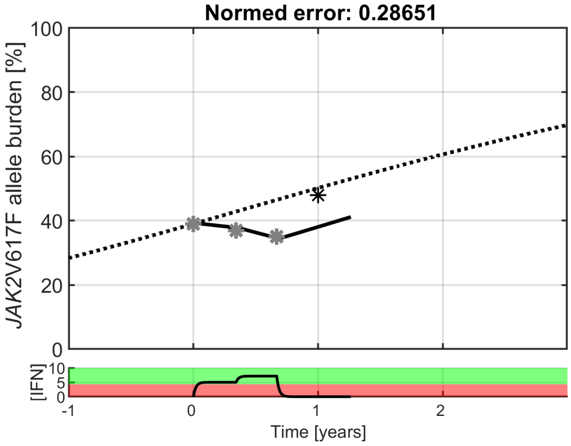

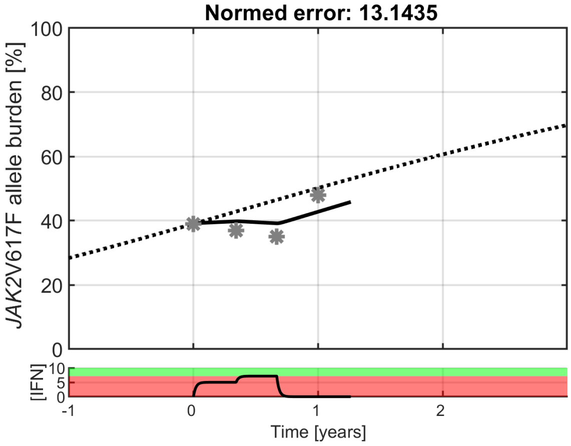

Patient P202

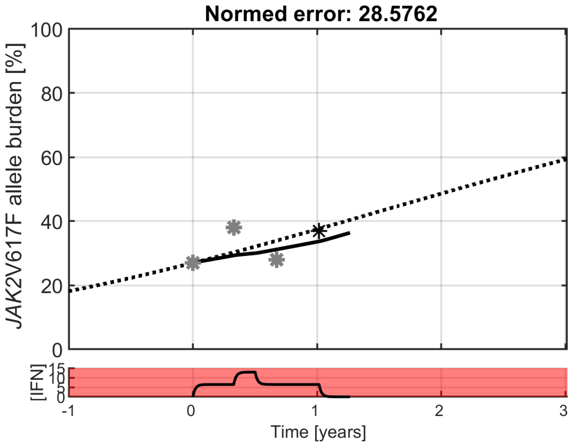

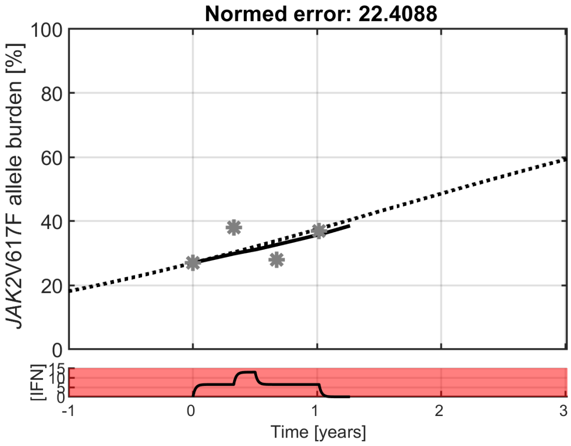

Patient P203

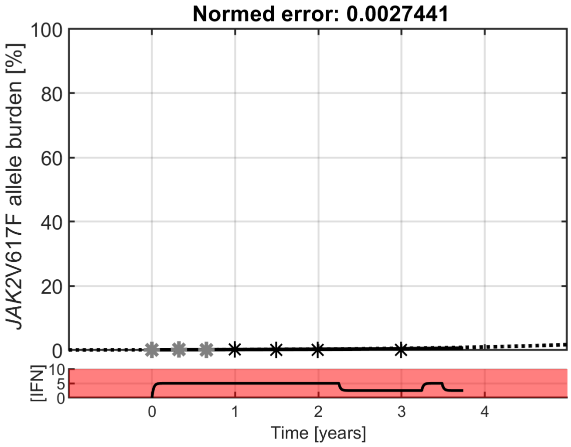

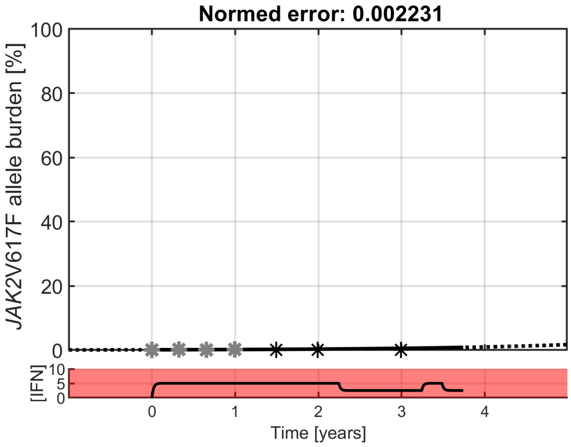

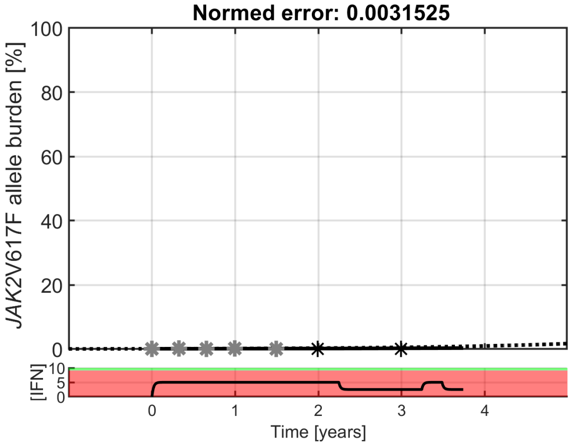

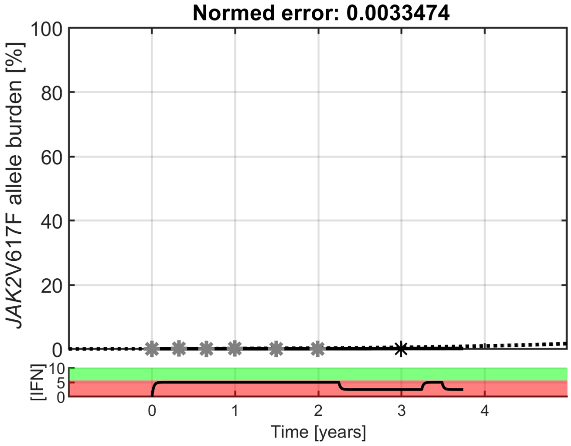

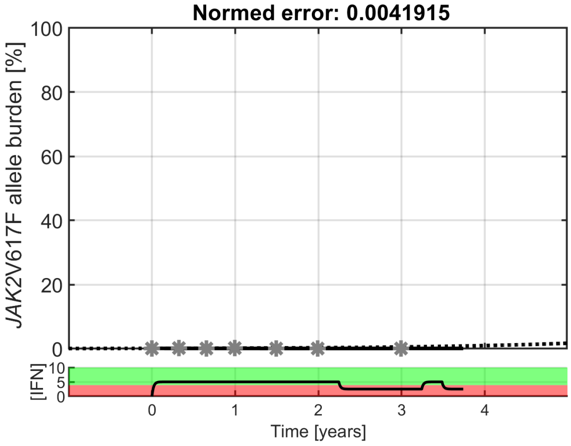

Patient P205

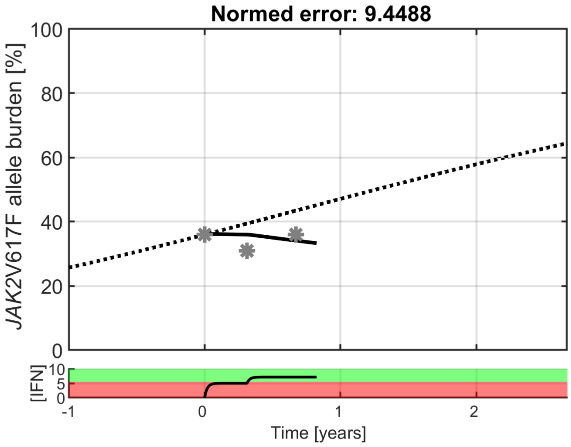

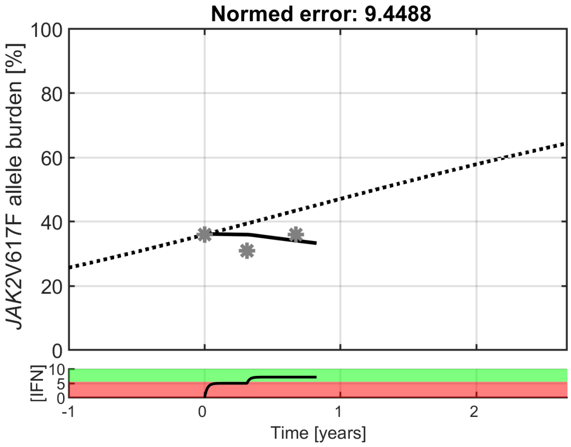

Supplementary material C: Fits to leukocyte and thrombocyte data

Here we show the best fit to *JAK2*V617F allele burden, along with the subsequent fits to leukocytes and thrombocytes.

The right-hand panels displays the full model fit to all data, see the description in supplementary material B. The two left-hand panels display the sum of mature cells in the corresponding model simulation, scaled to fit with either the leukocytes or the thrombocyte. The resulting scaling-factor hence describes the assumed fraction of mature cells that correspond to the given cell-type. In the figures, the approximate healthy intervals are also shown, defined as between $4 \cdot 10^3$ to $11 \cdot 10^3$ per microliter of blood for leukocytes and $145 \cdot 10^3$ to $390 \cdot 10^3$ per microliter of blood thrombocytes. We emphasize that the patient-specific response to IFN in the model was only fit to the data for the *JAK2*V617F allele burden, and the scaling of the mature cell counts was done subsequently, independent of the IFN blood-concentration.

At the top of the leukocytes and thrombocyte figures, a goodness-of-fit-metric of the best fit is shown, defined as the sum of squared errors, divided by the number of points used in the fit. A low number signifies a good fit. Note that the error metric depends on the order of magnitude of the data, and hence the metric is typically higher for thrombocytes than for leukocytes.

Patient P002

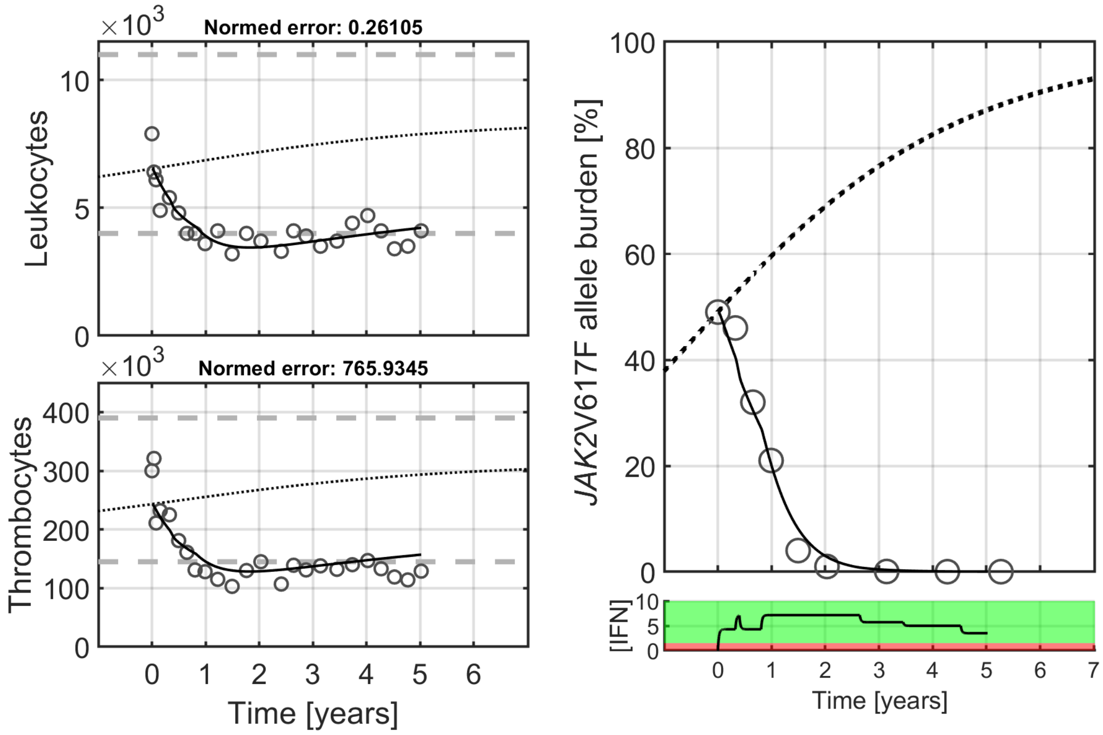

Patient P003

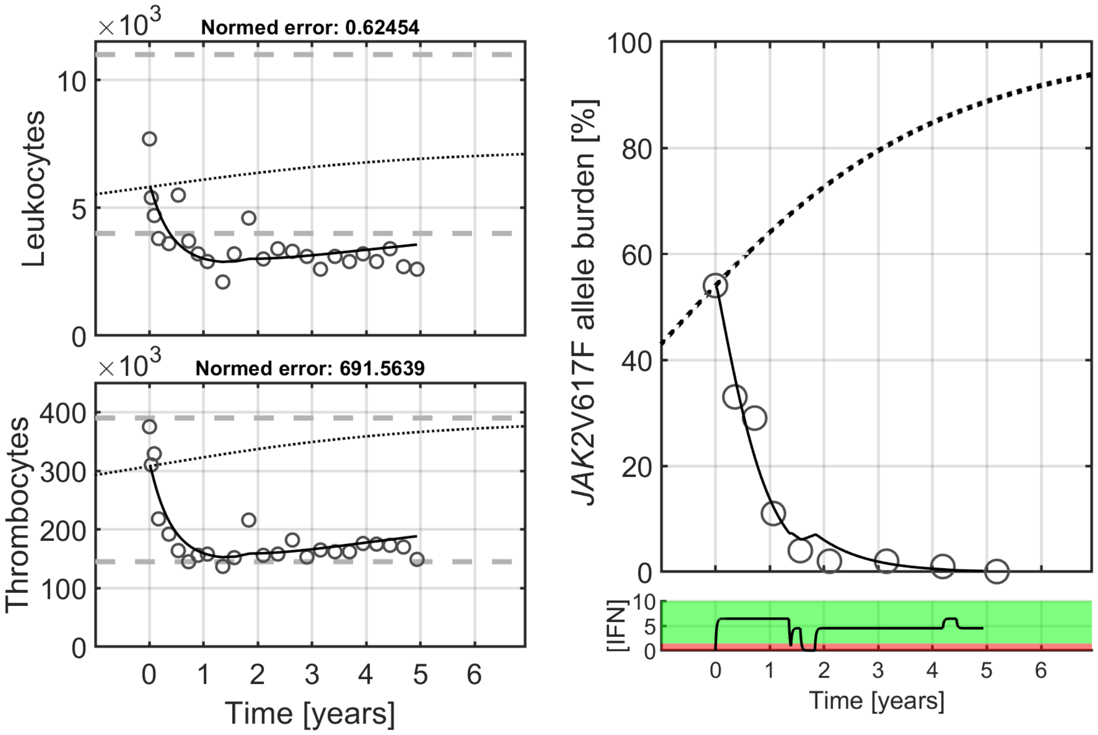

Patient P004

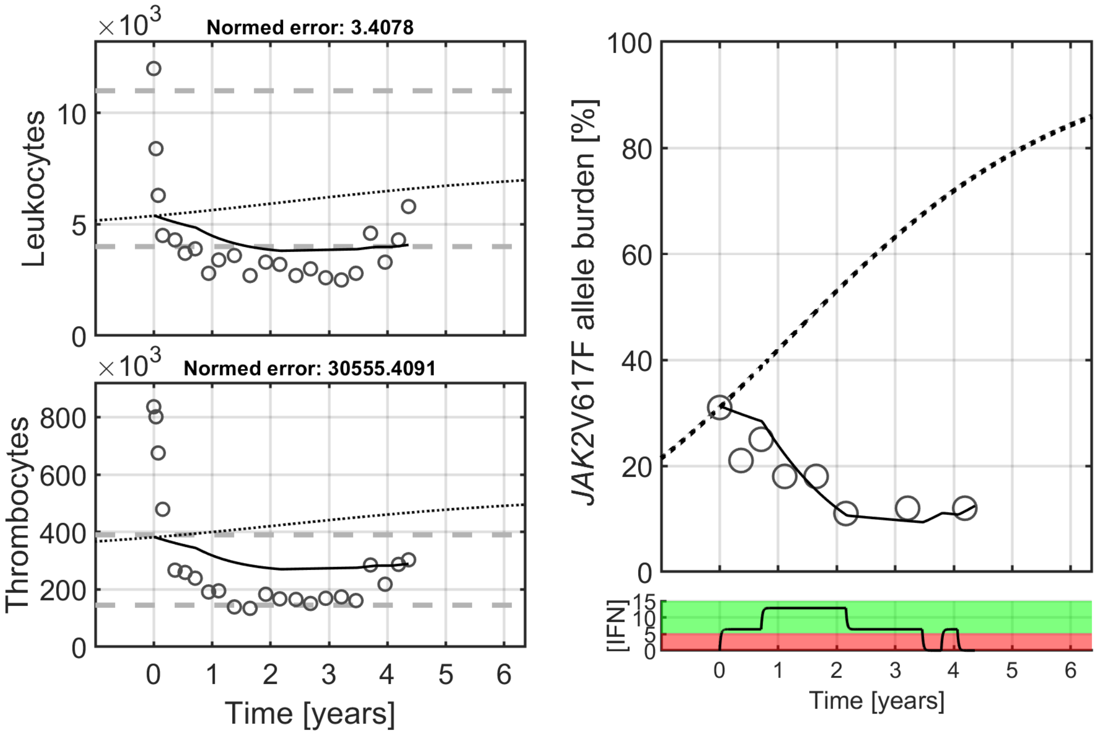

Patient P006

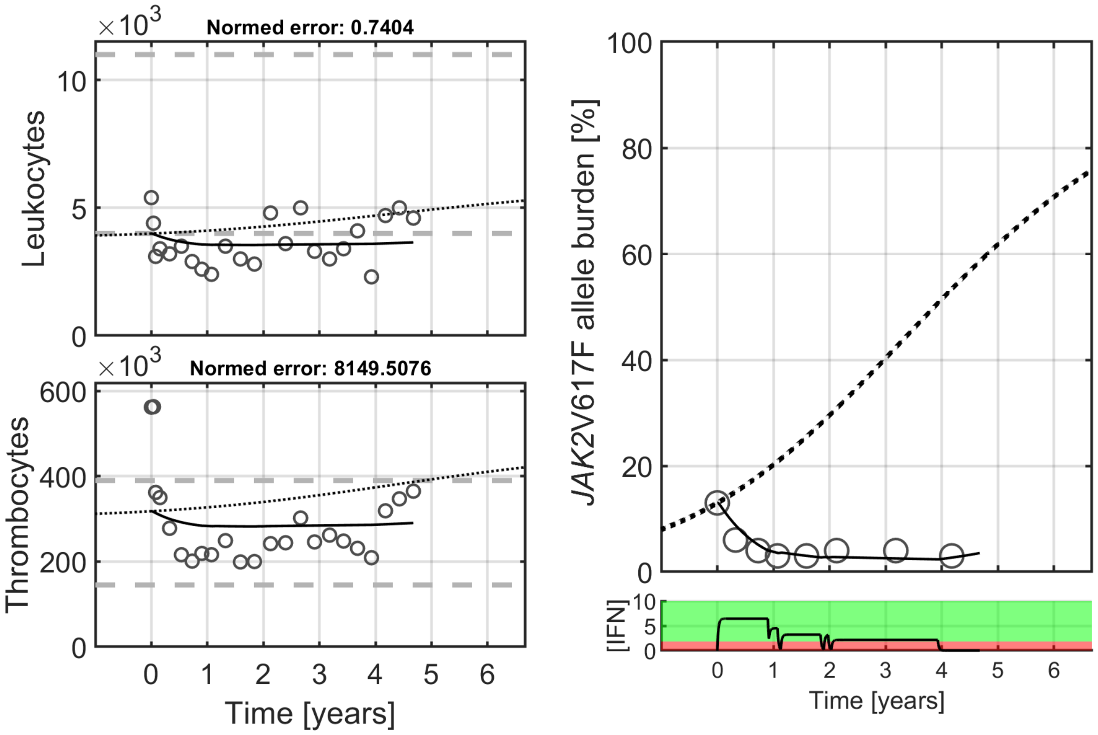

×10^3
Normed error: 0.7404
Leukocytes
10
5
0
×10^3
Normed error: 8149.5076
Thrombocytes
600
400
200
0
0 1 2 3 4 5 6
Time [years]
JAK2V617F allele burden [%]
100
80
60
40
20
0
[IFN]
10
5
0
0 1 2 3 4 5 6
Time [years]

Patient P011

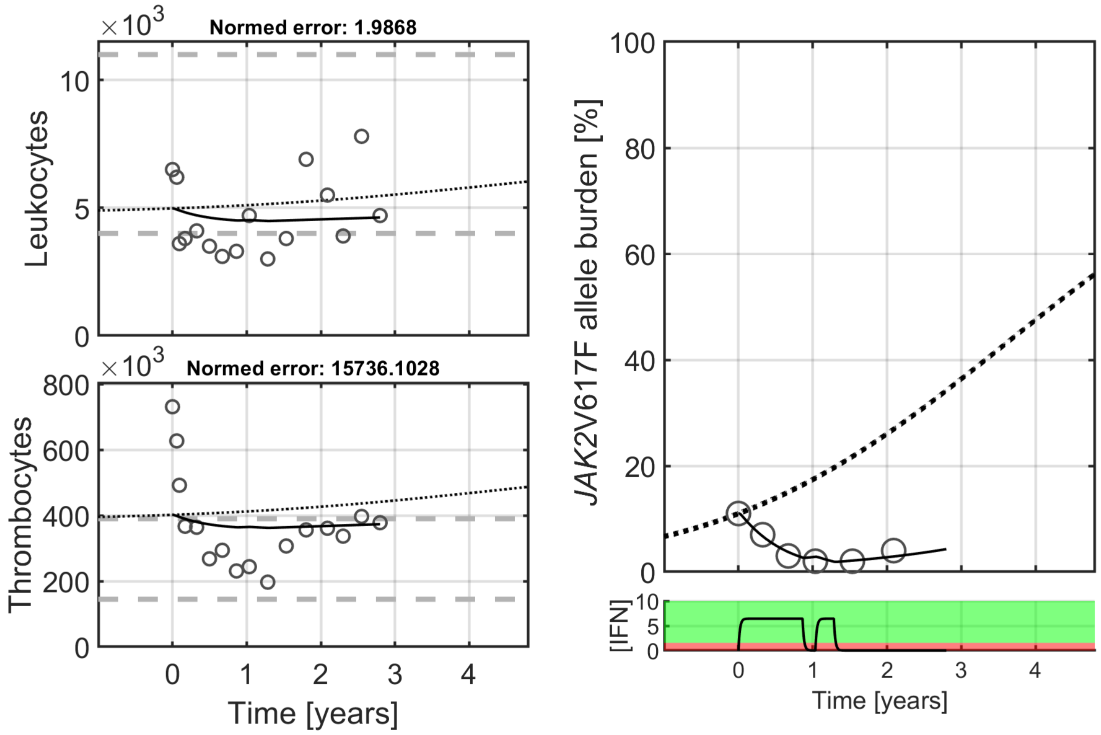

Patient P014

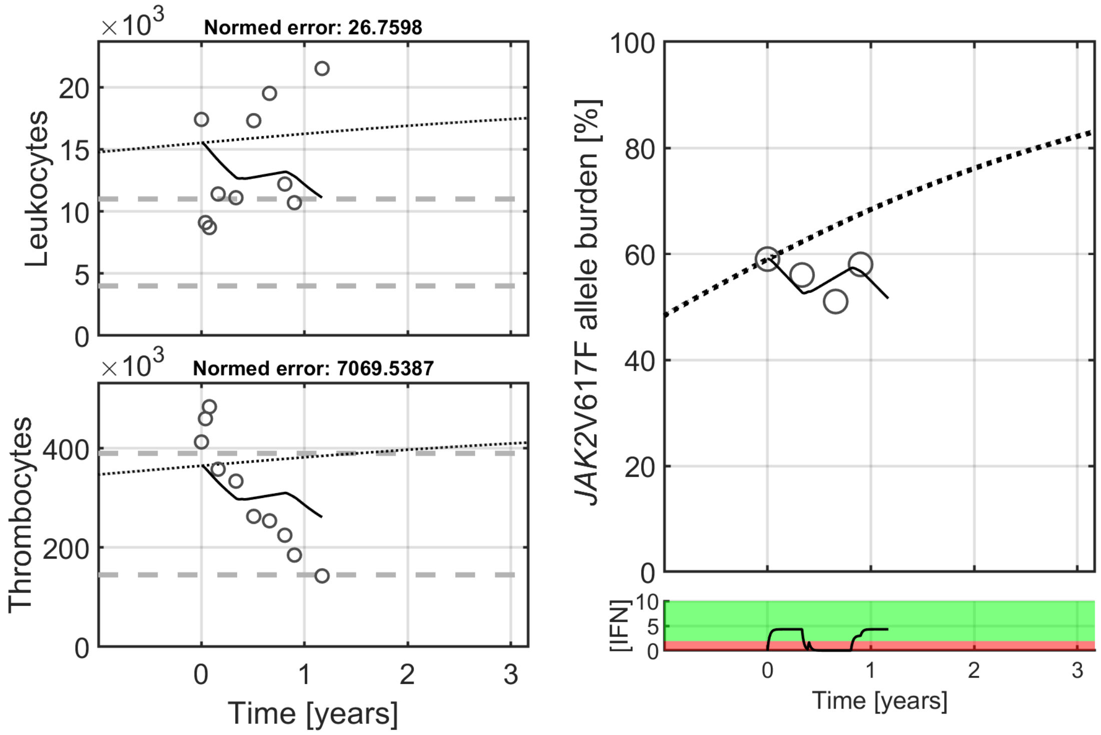

Patient P016

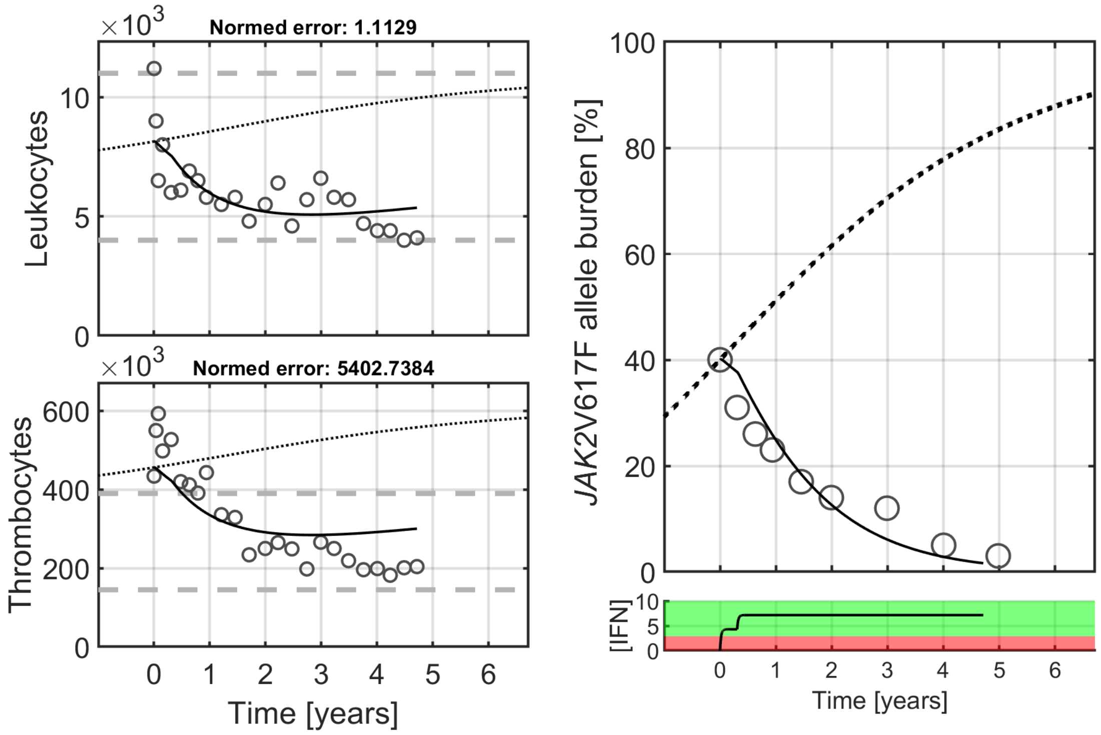

Patient P021

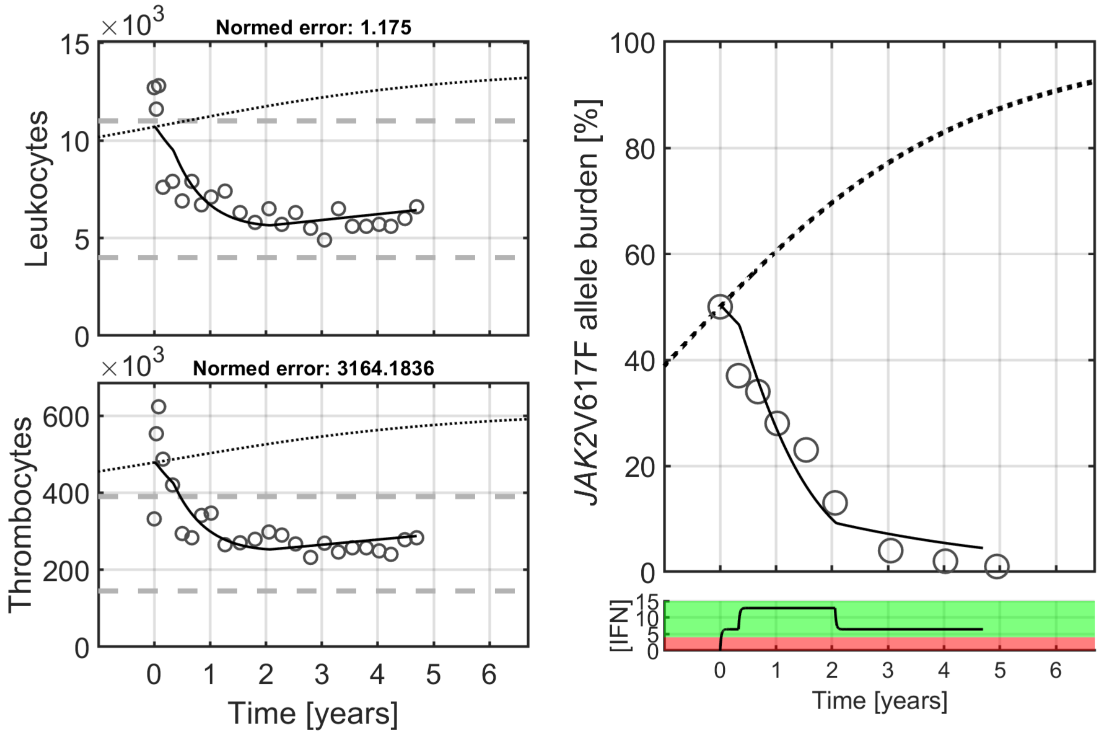

Patient P026

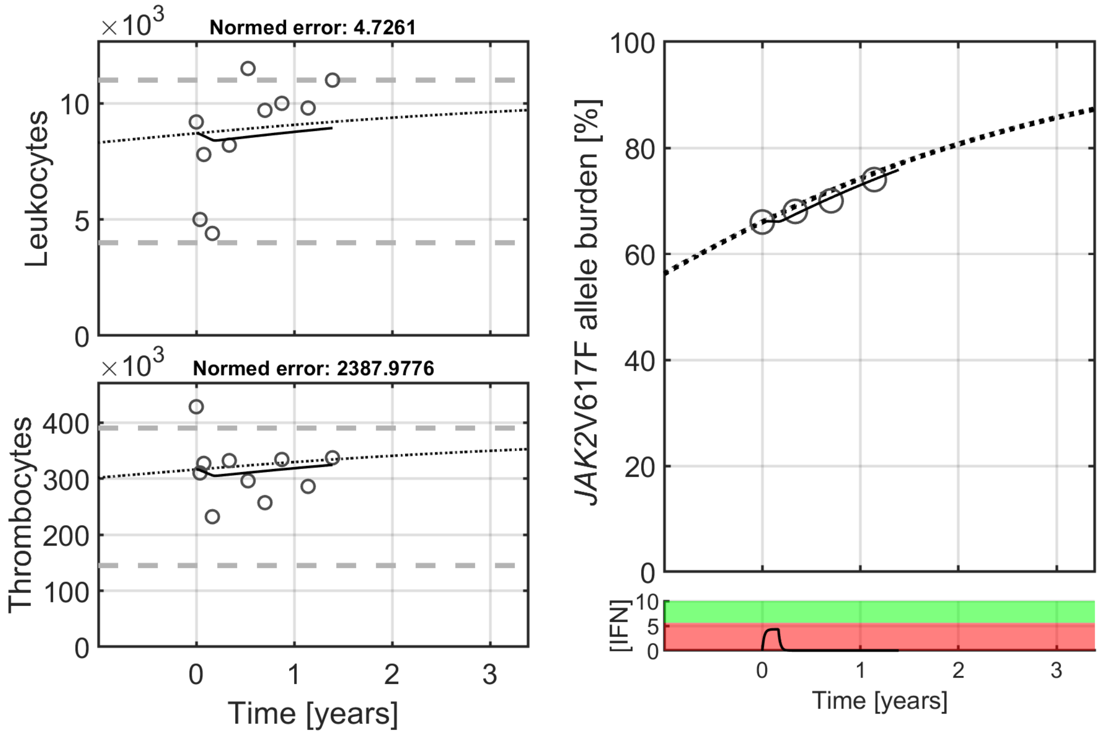

Patient P028

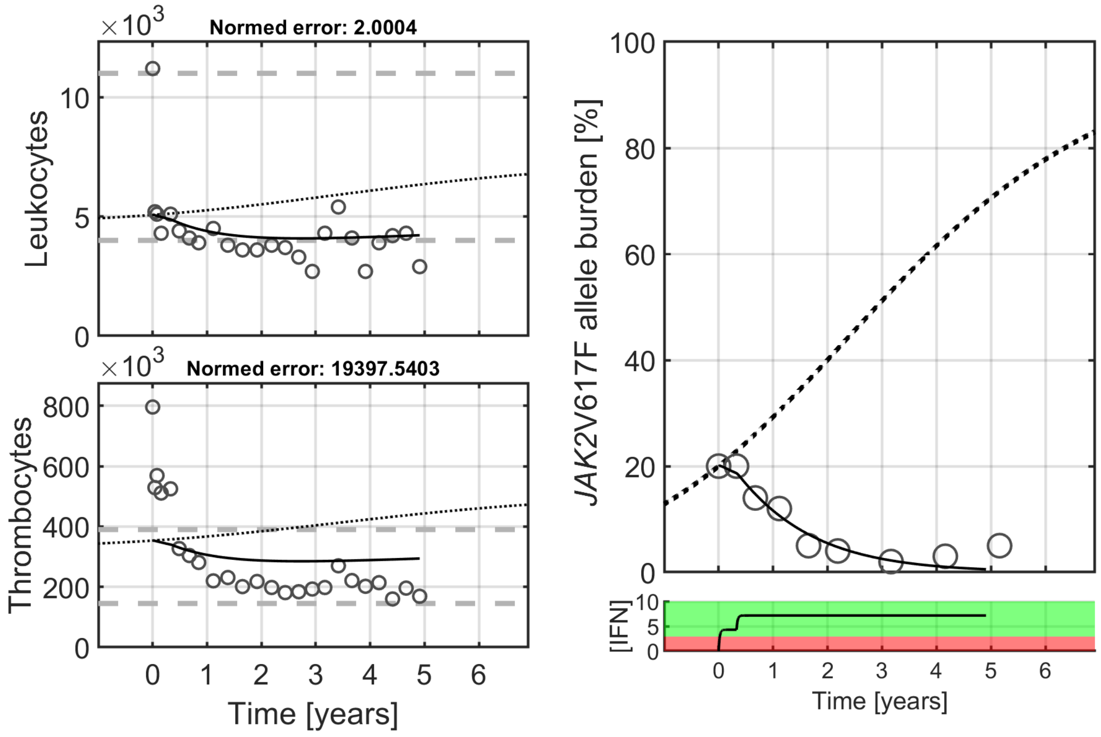

Patient P035

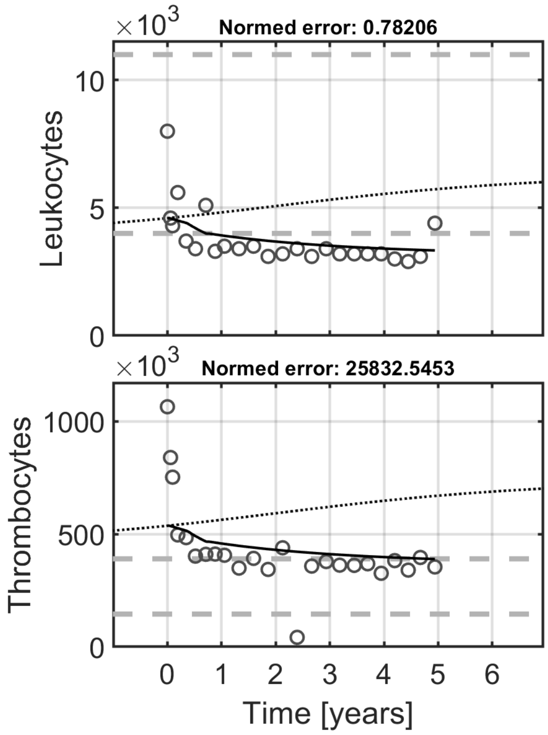

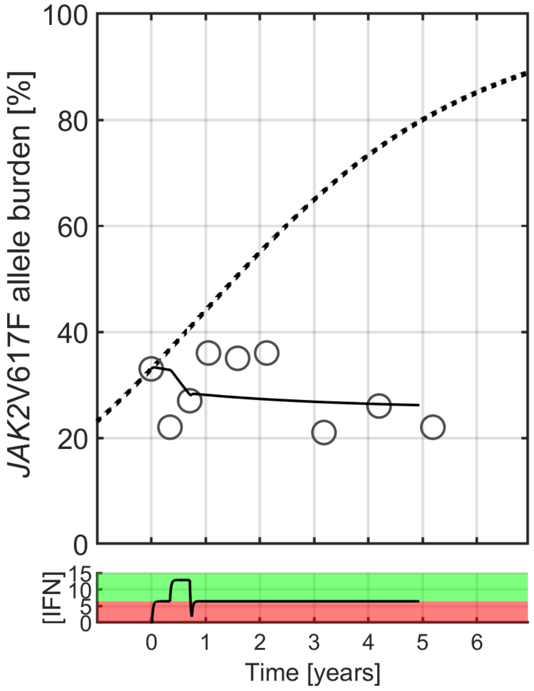

Patient P039

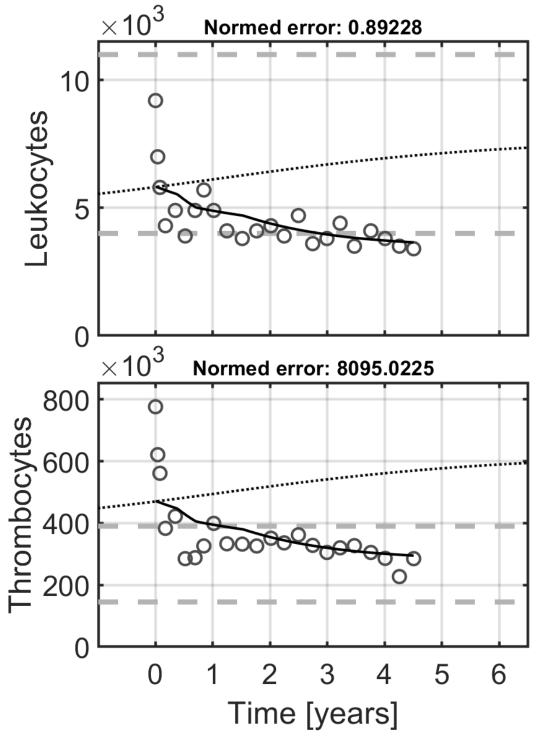

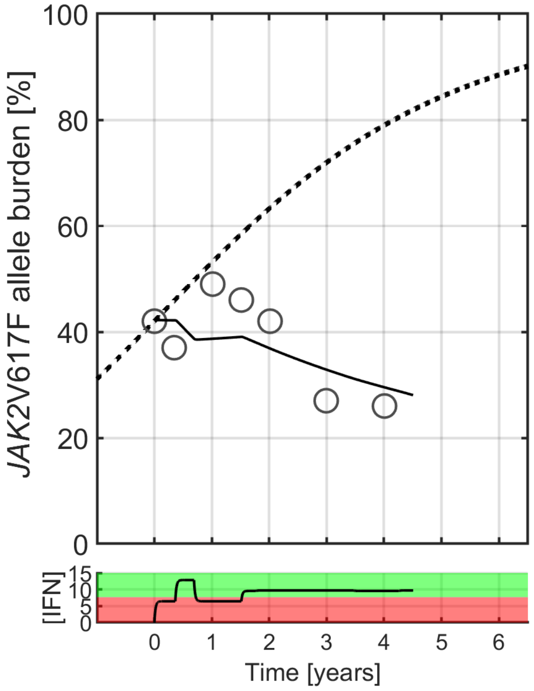

Patient P045

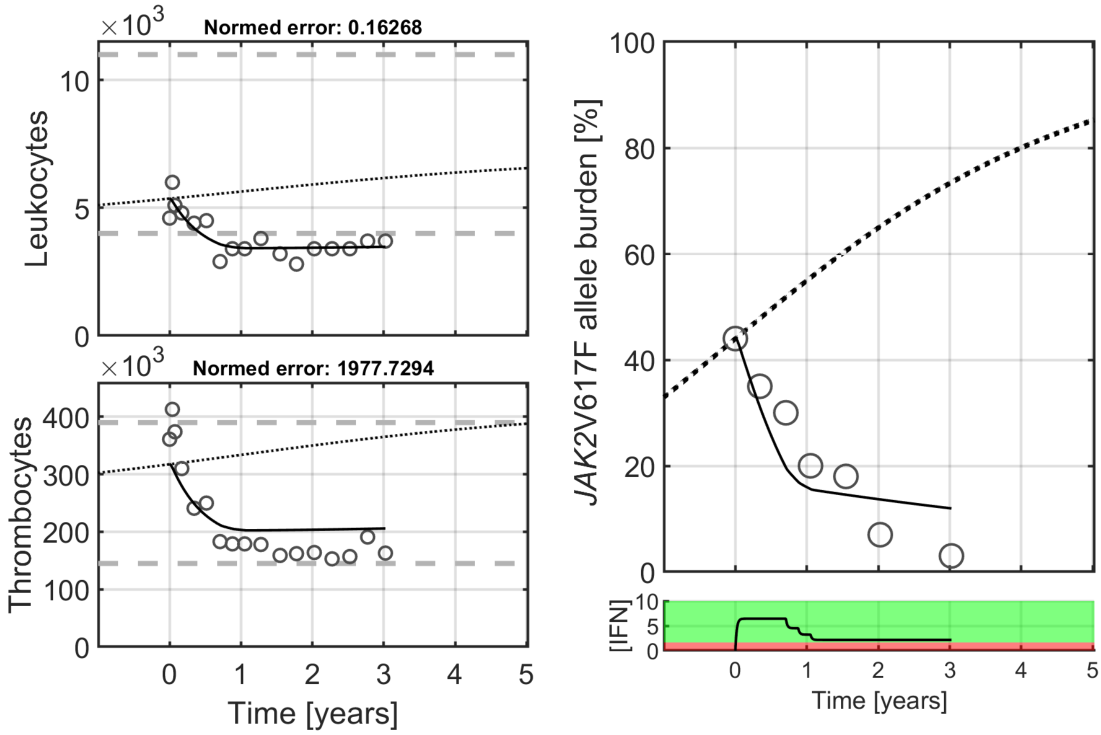

Patient P046

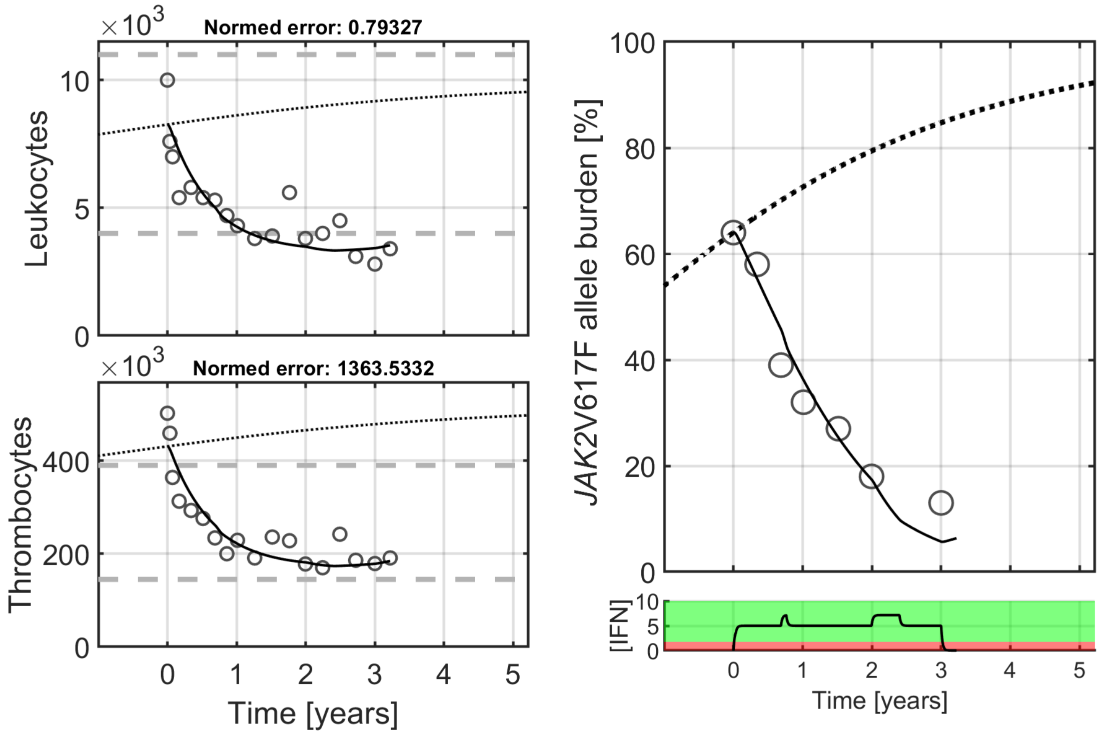

Patient P049

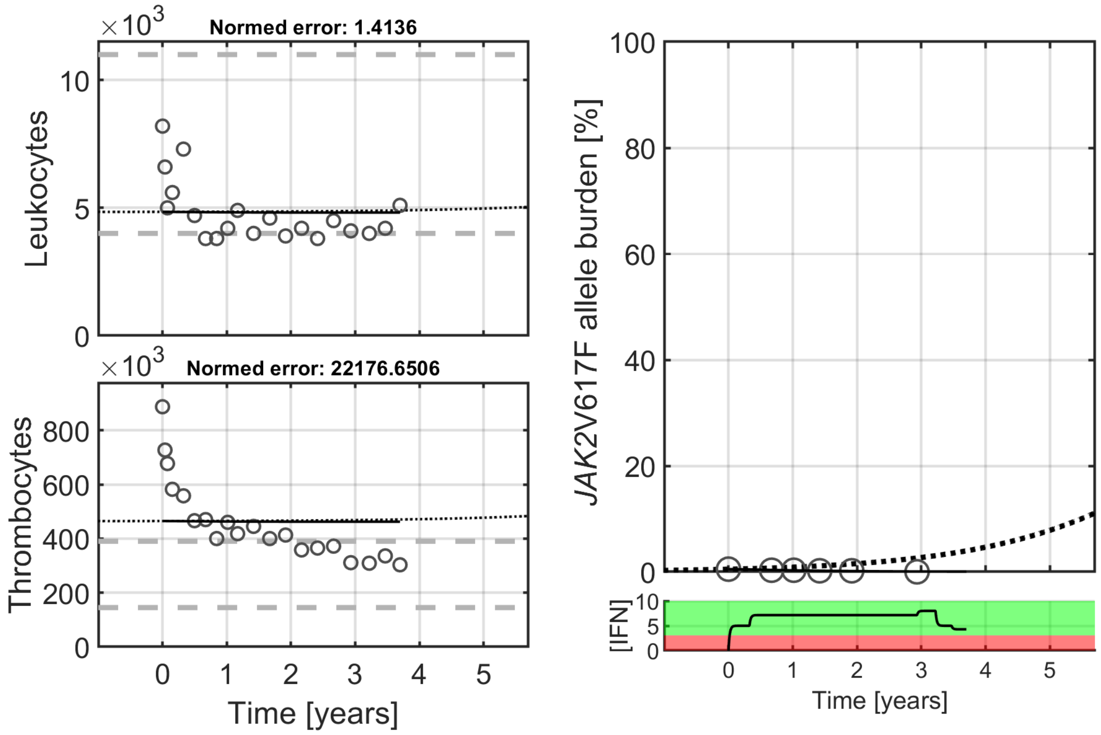

Patient P062

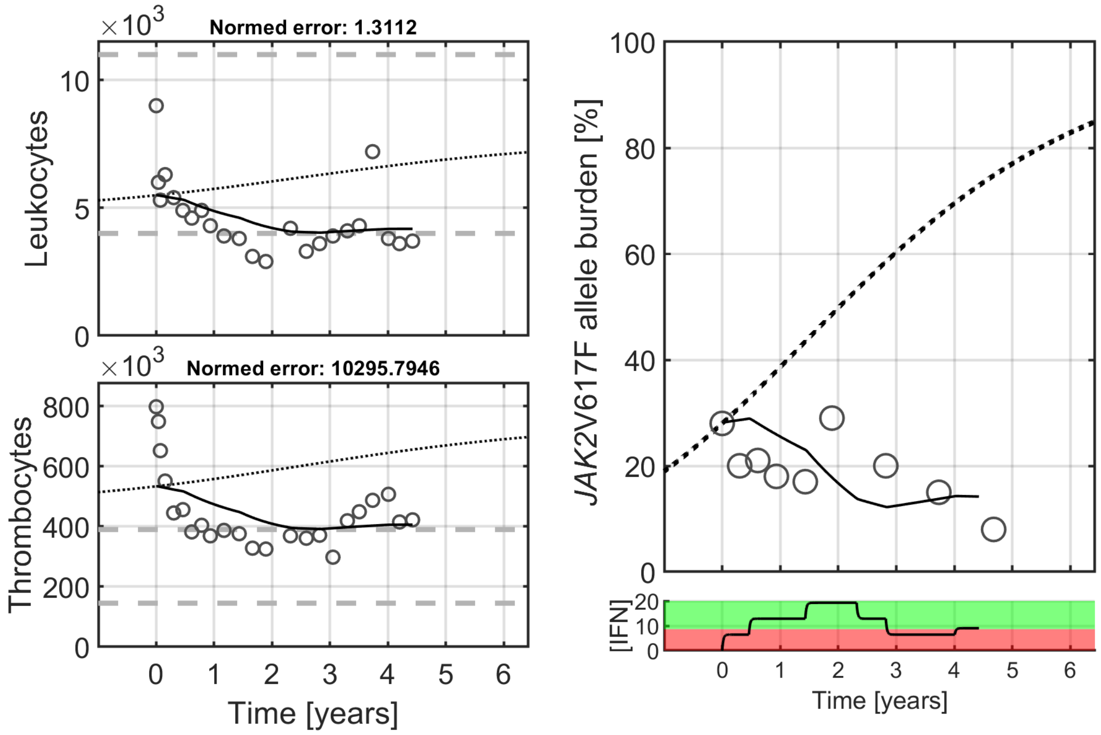

Patient P067

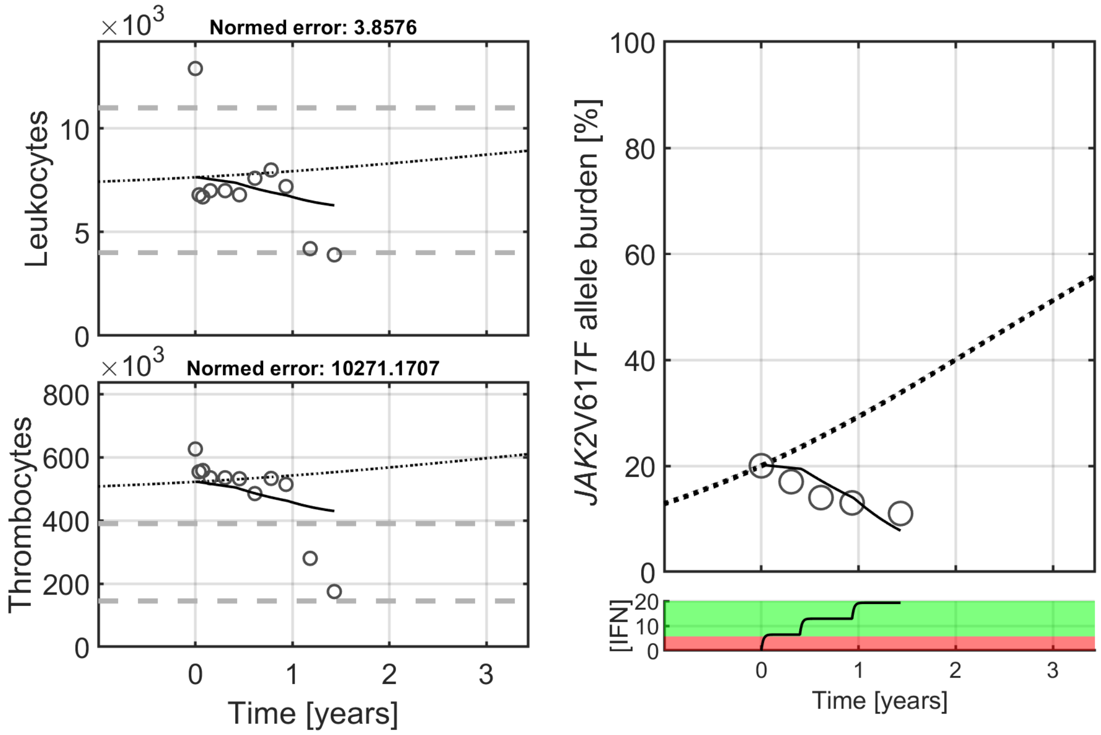

Patient P070

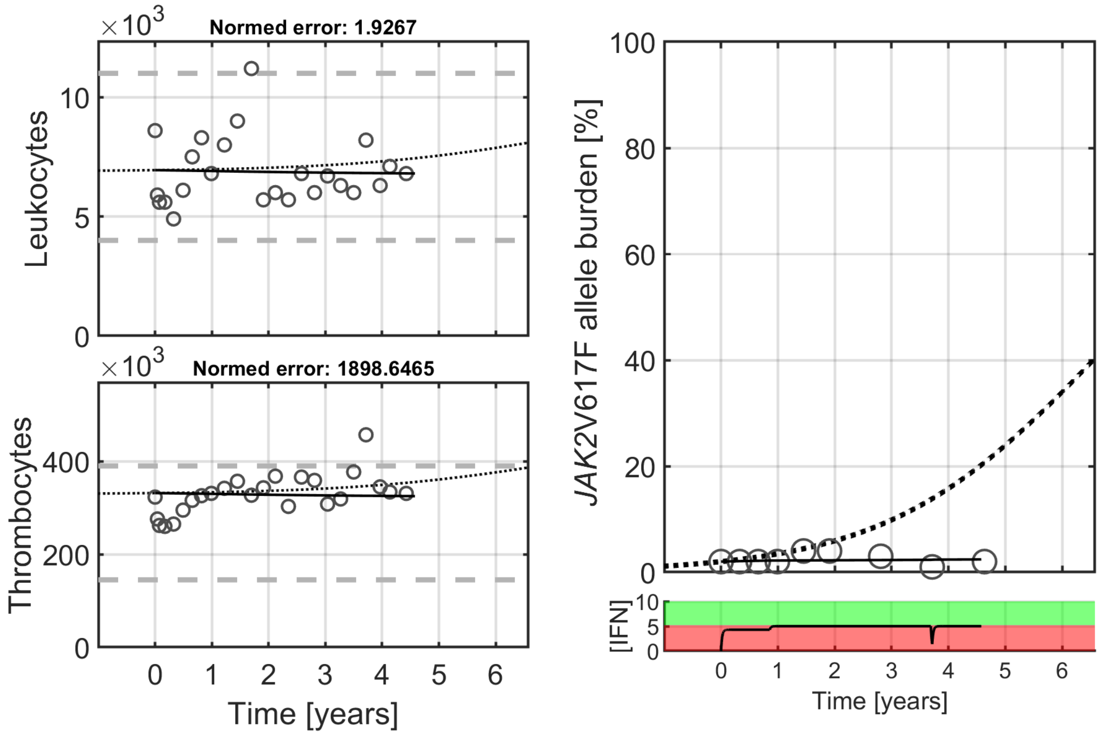

Patient P075

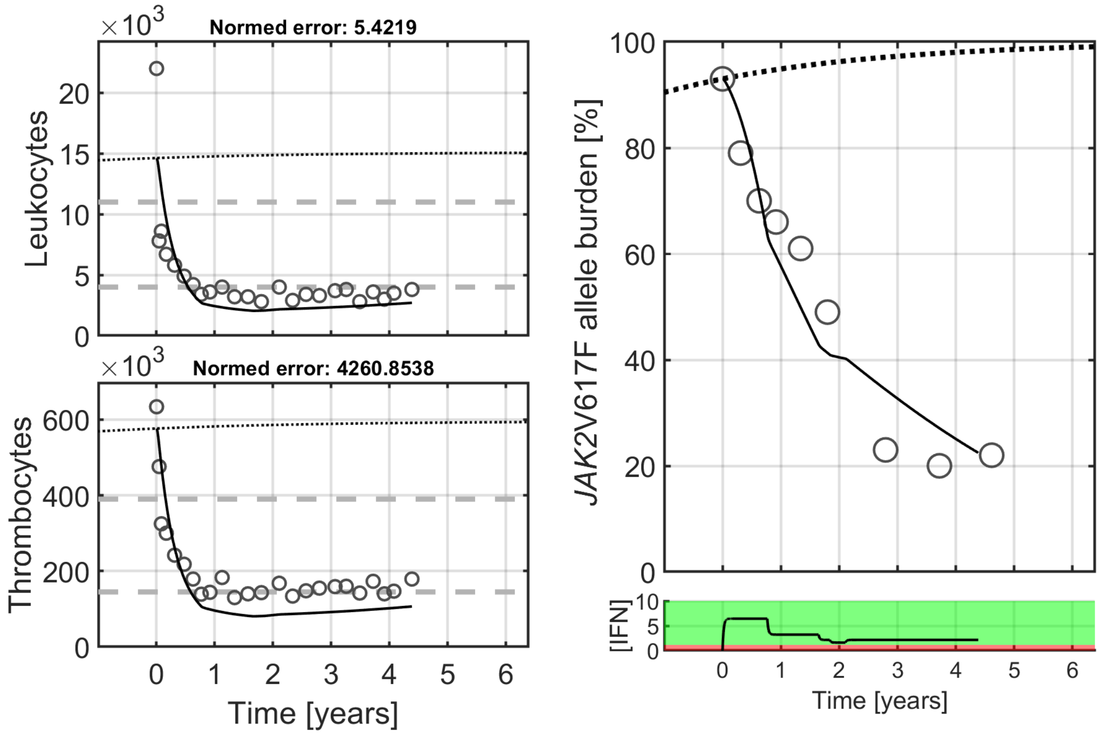

Patient P078

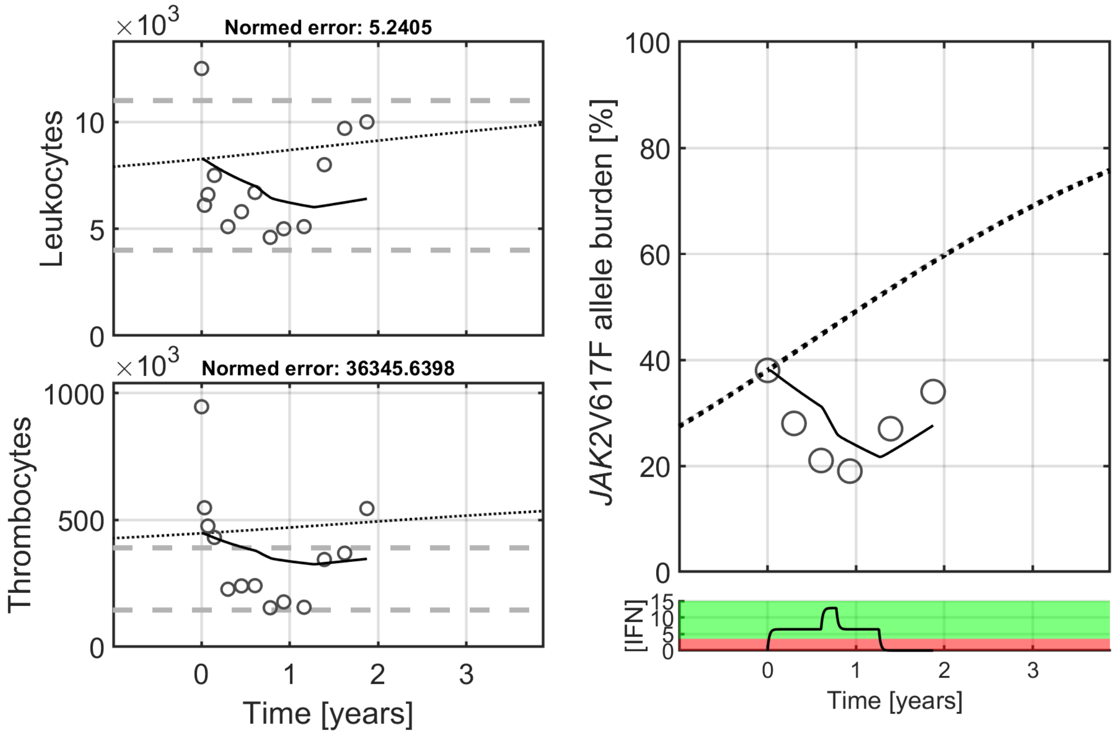

Patient P079

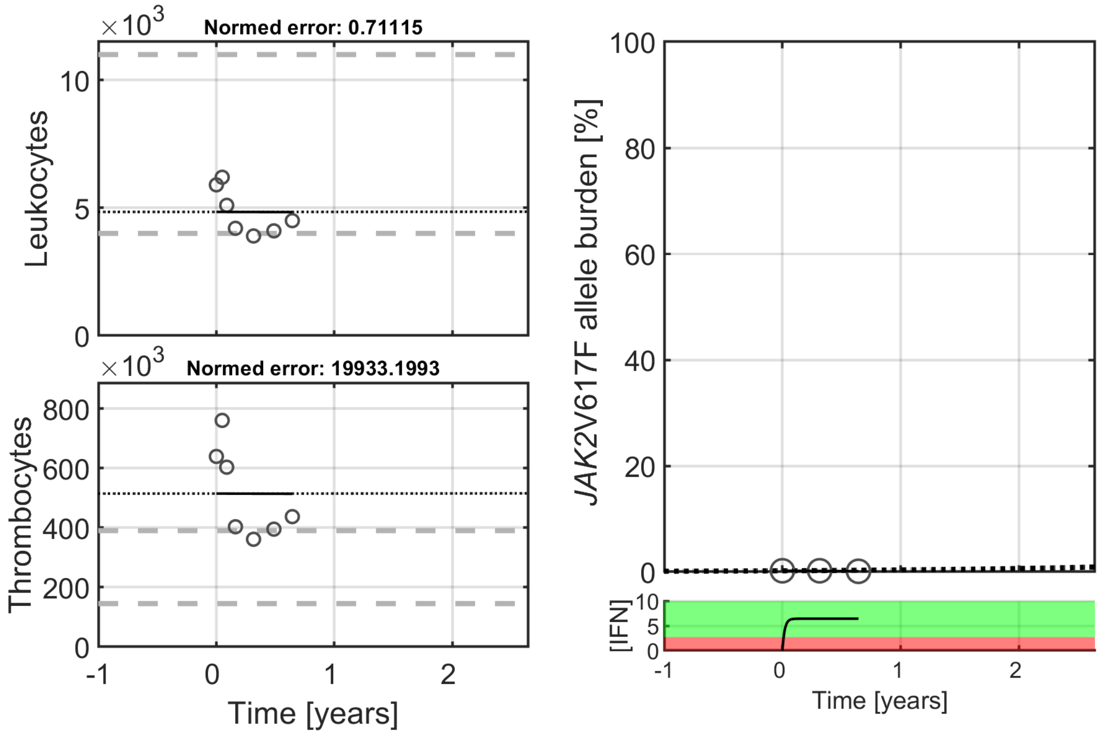

Patient P082

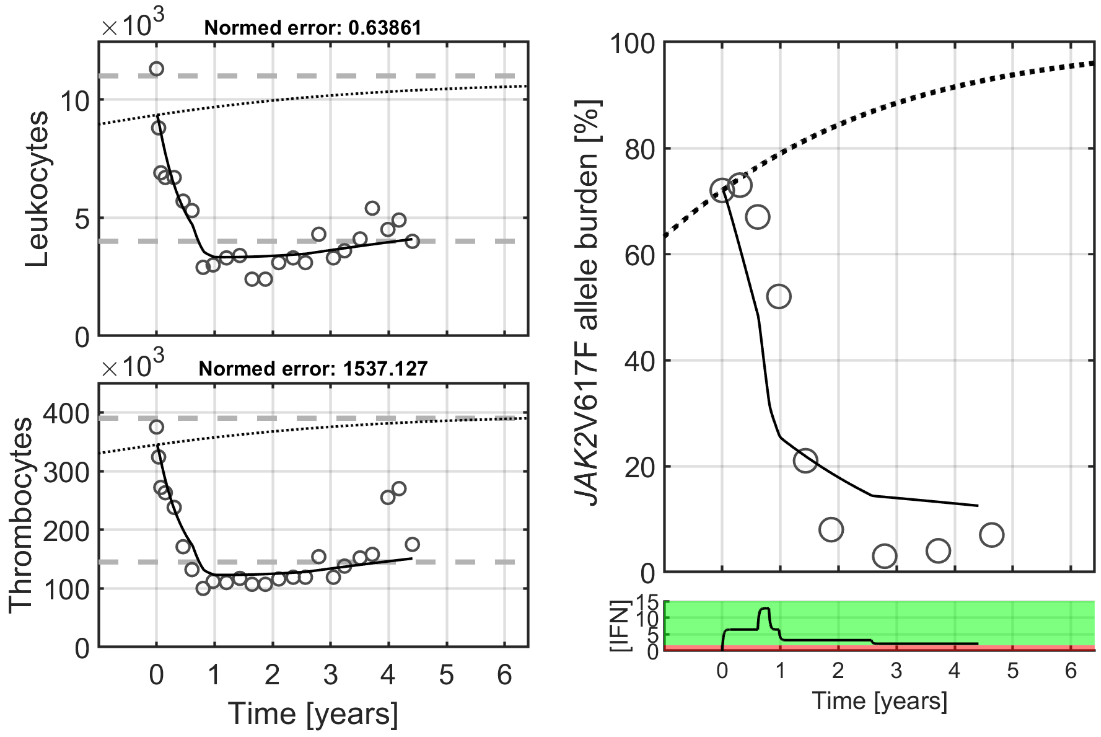

Patient P084

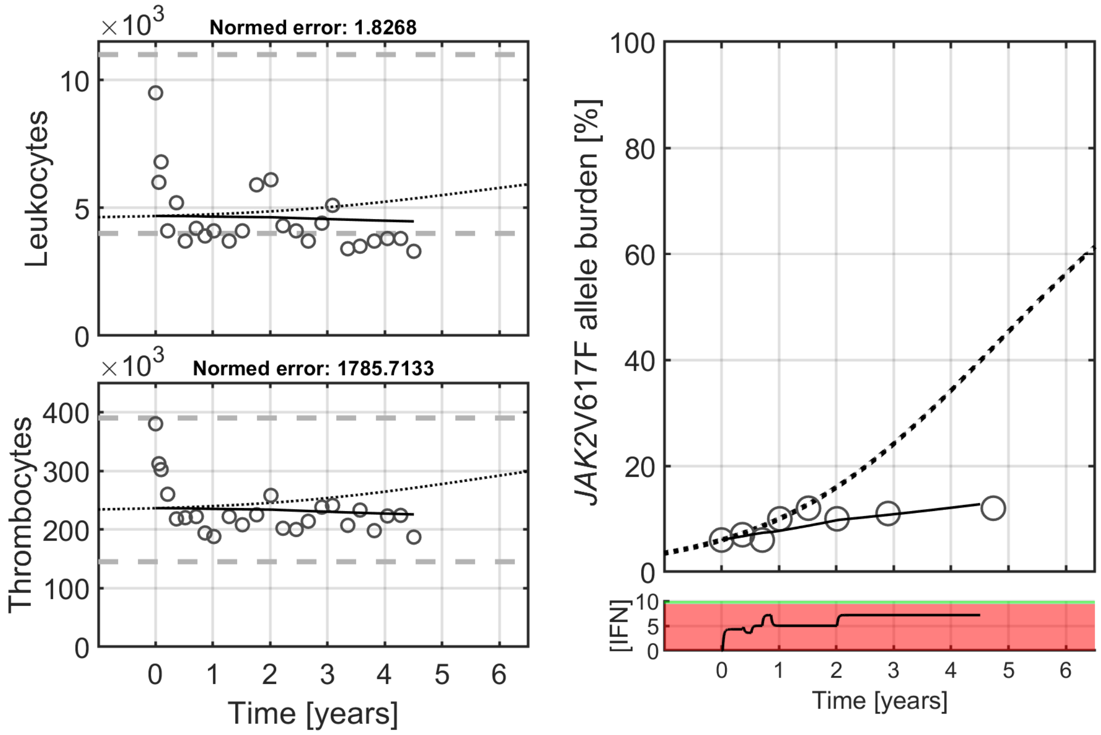

Patient P086

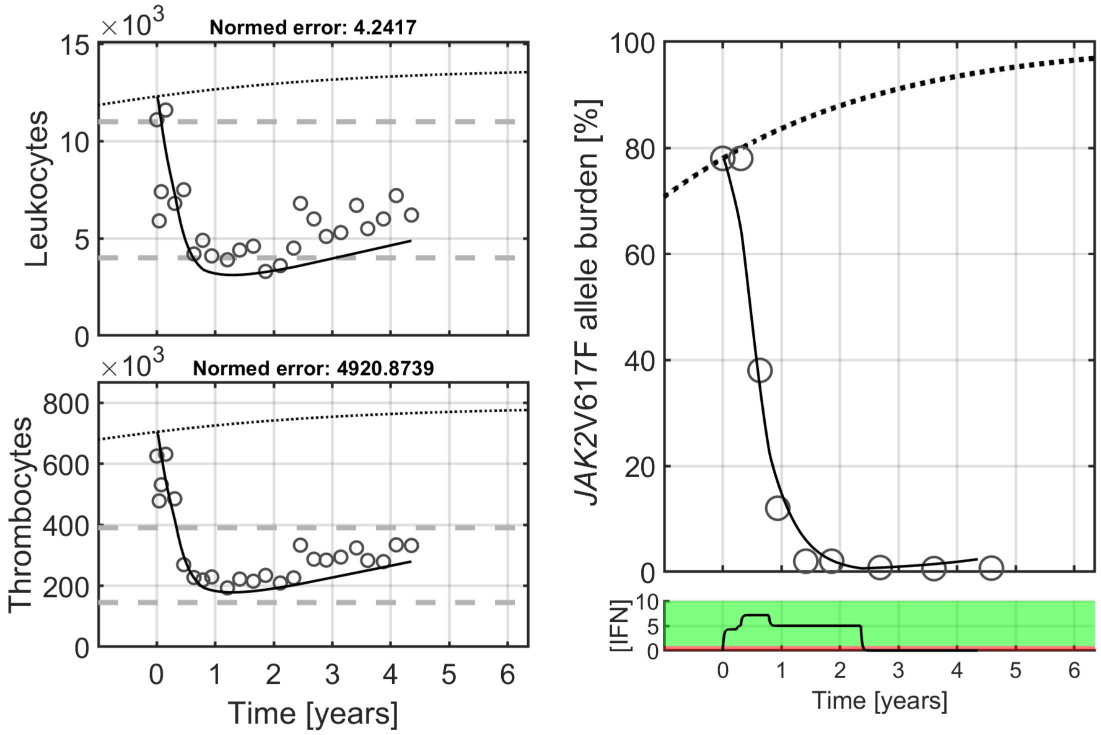

Patient P087

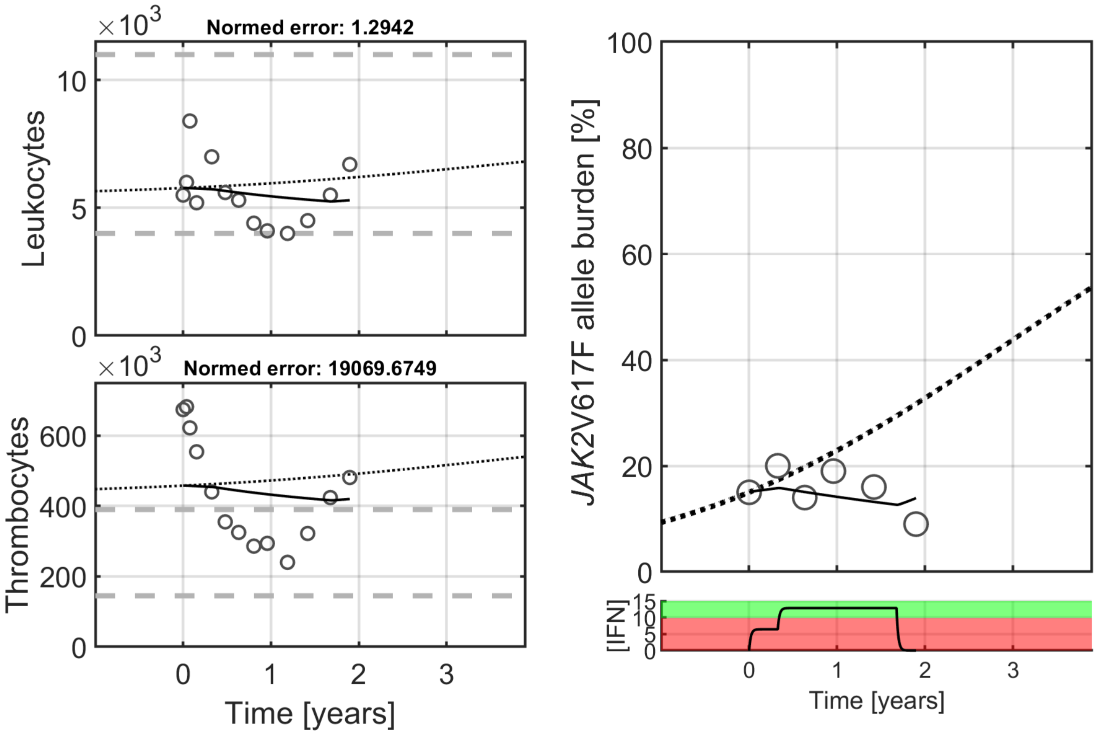

Patient P089

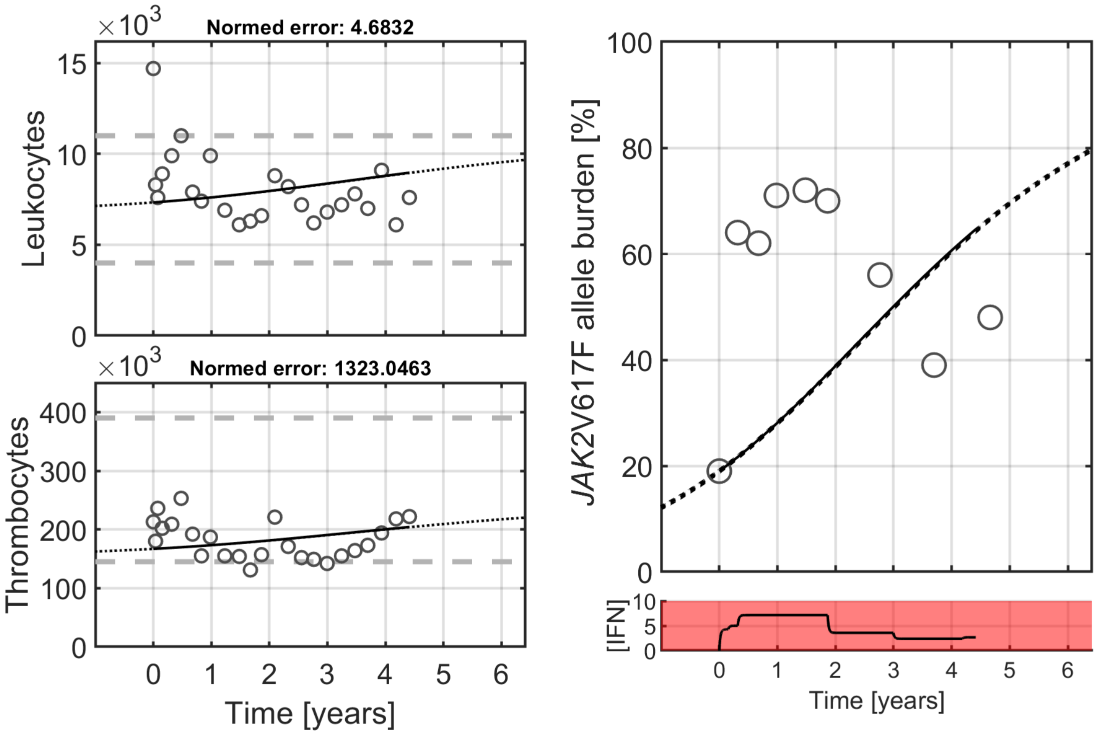

Patient P105

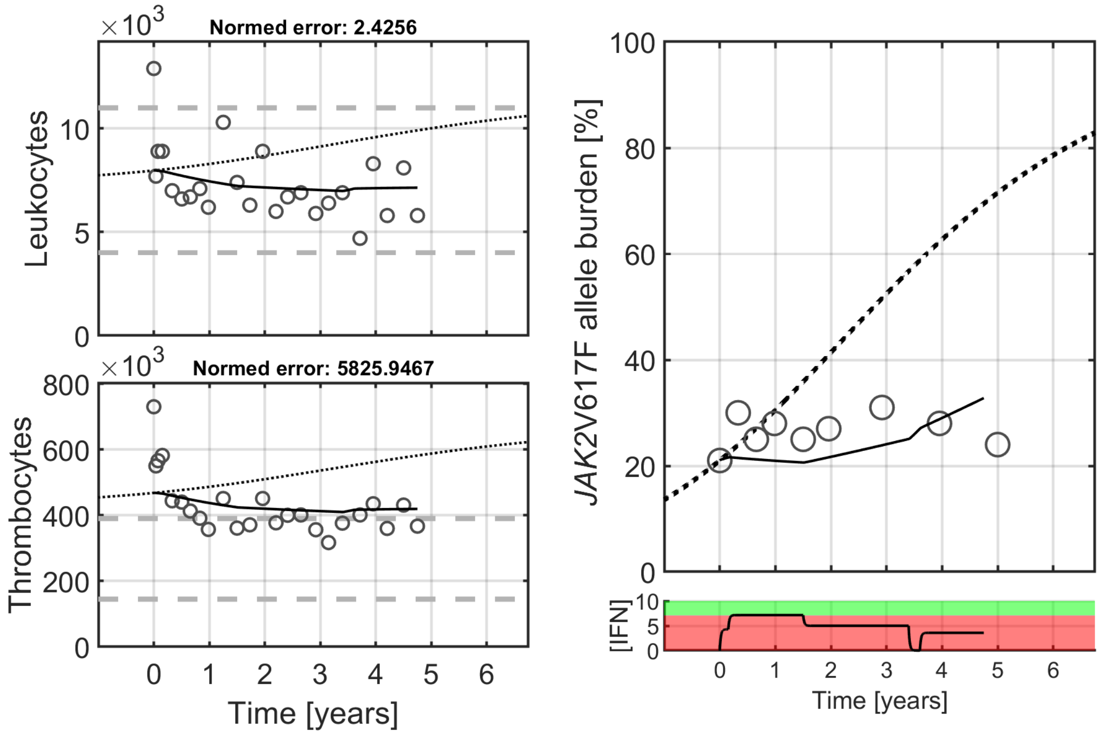

Patient P106

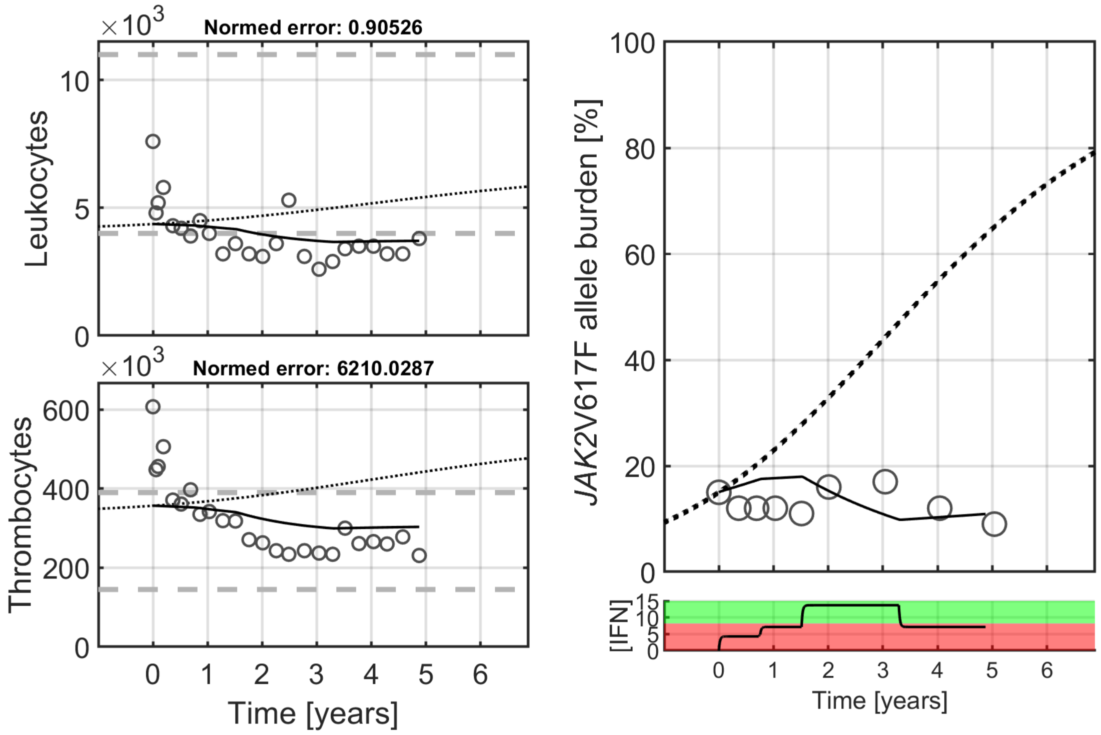

Patient P111

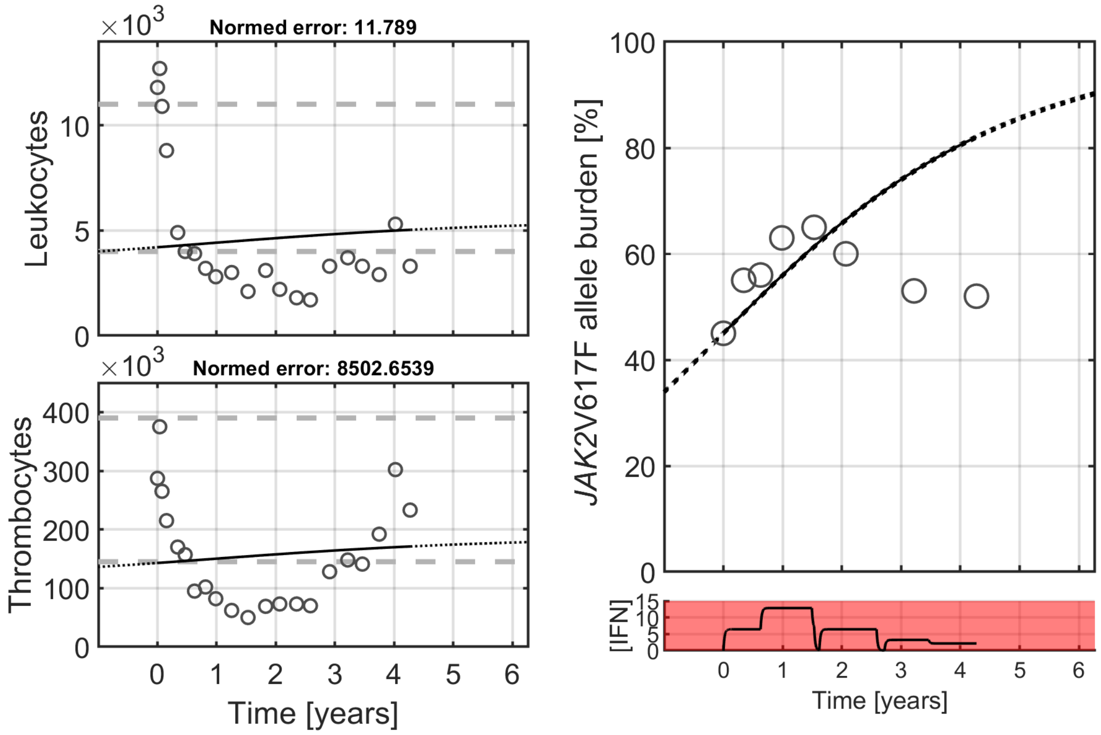

Patient P113

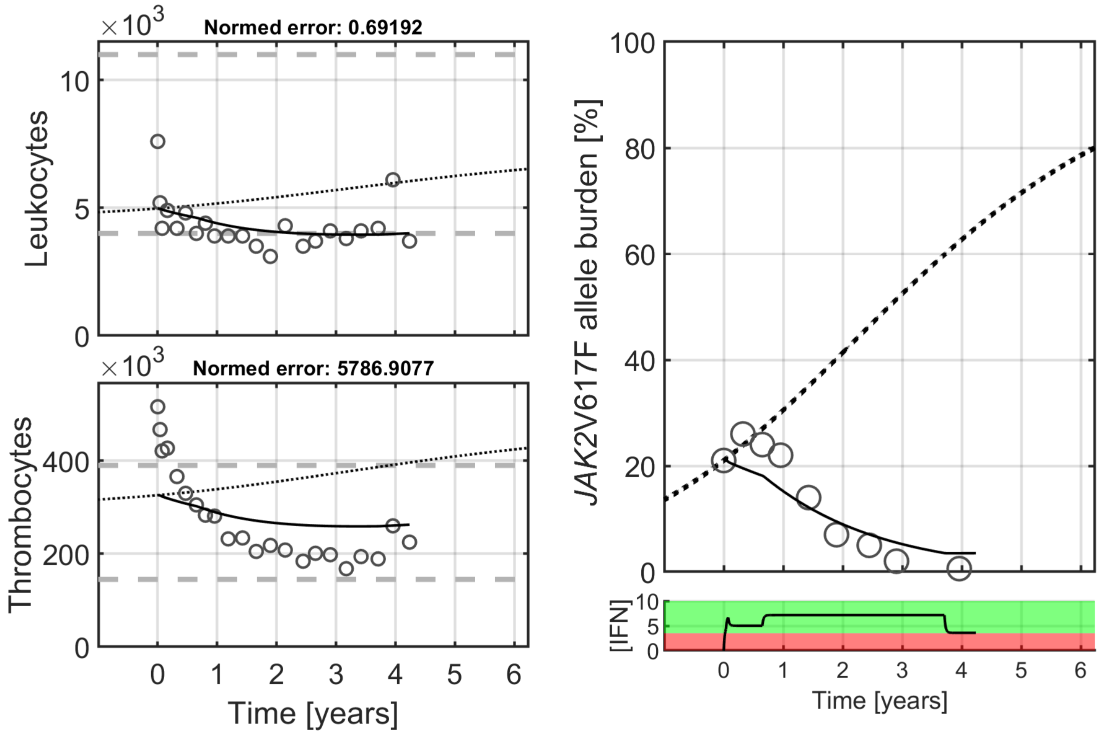

Patient P114

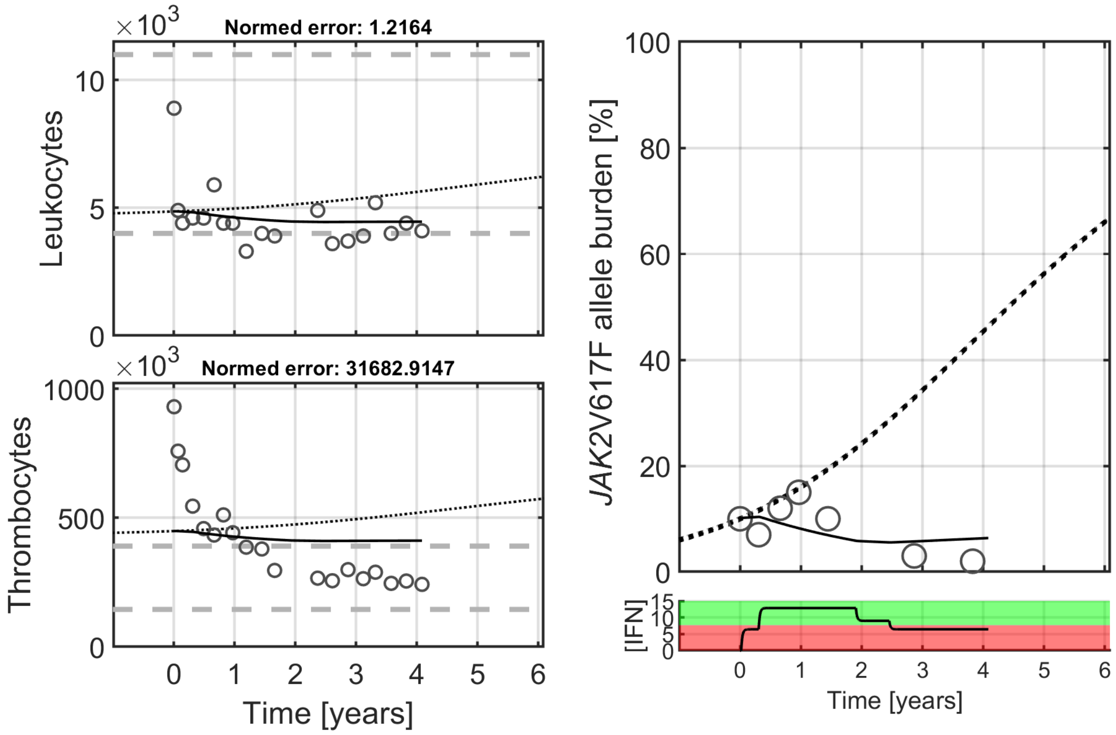

Patient P117

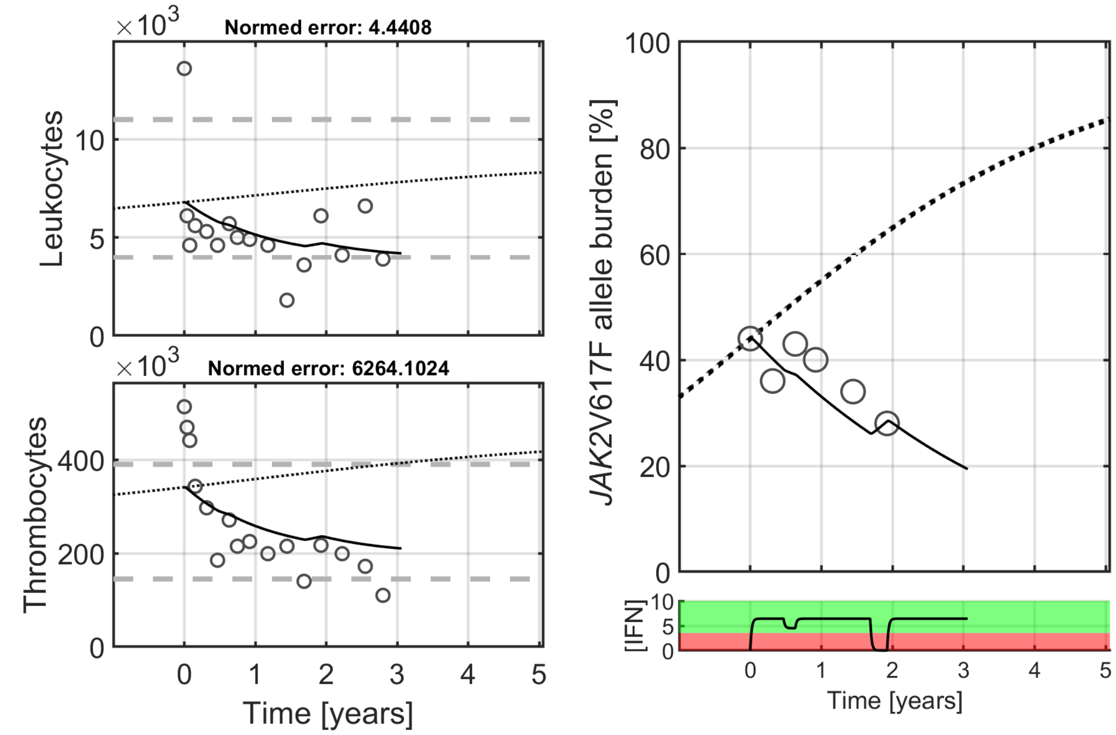

Patient P122

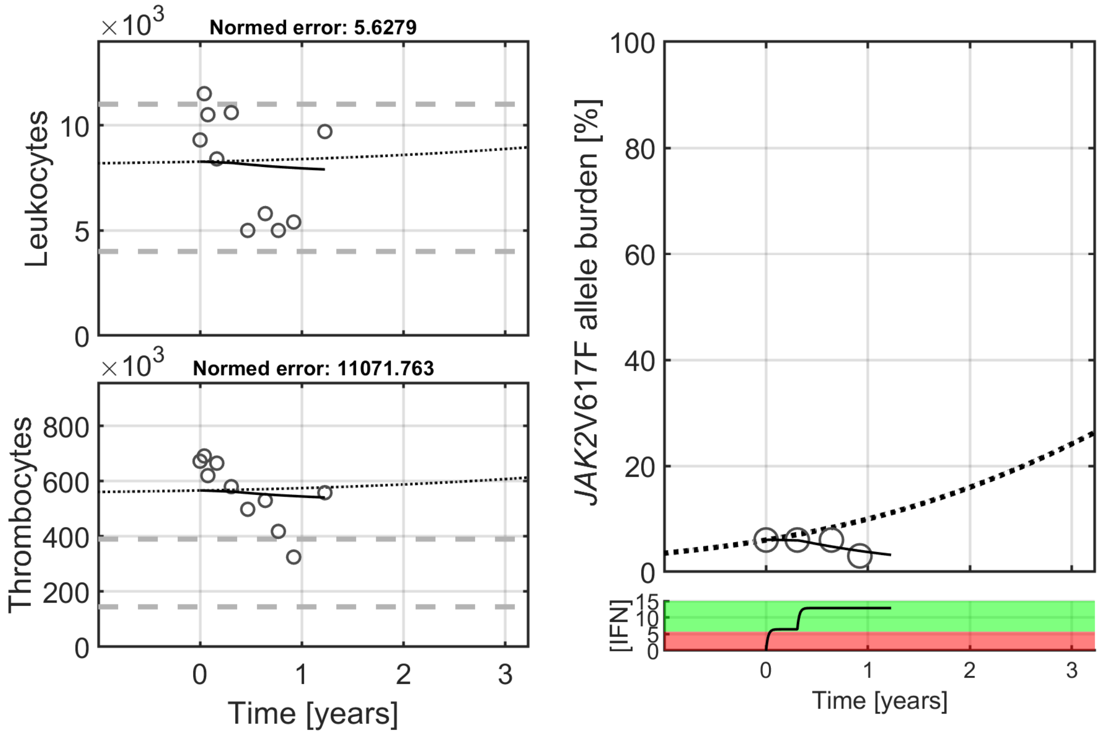

Patient P124

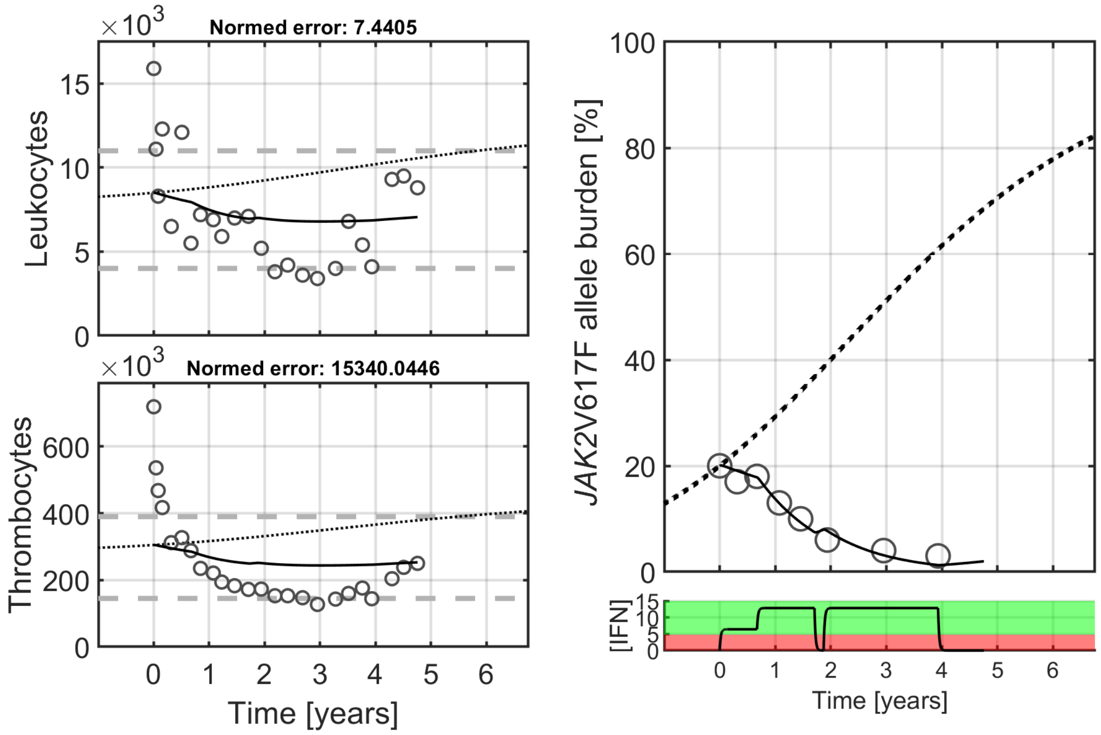

Patient P125

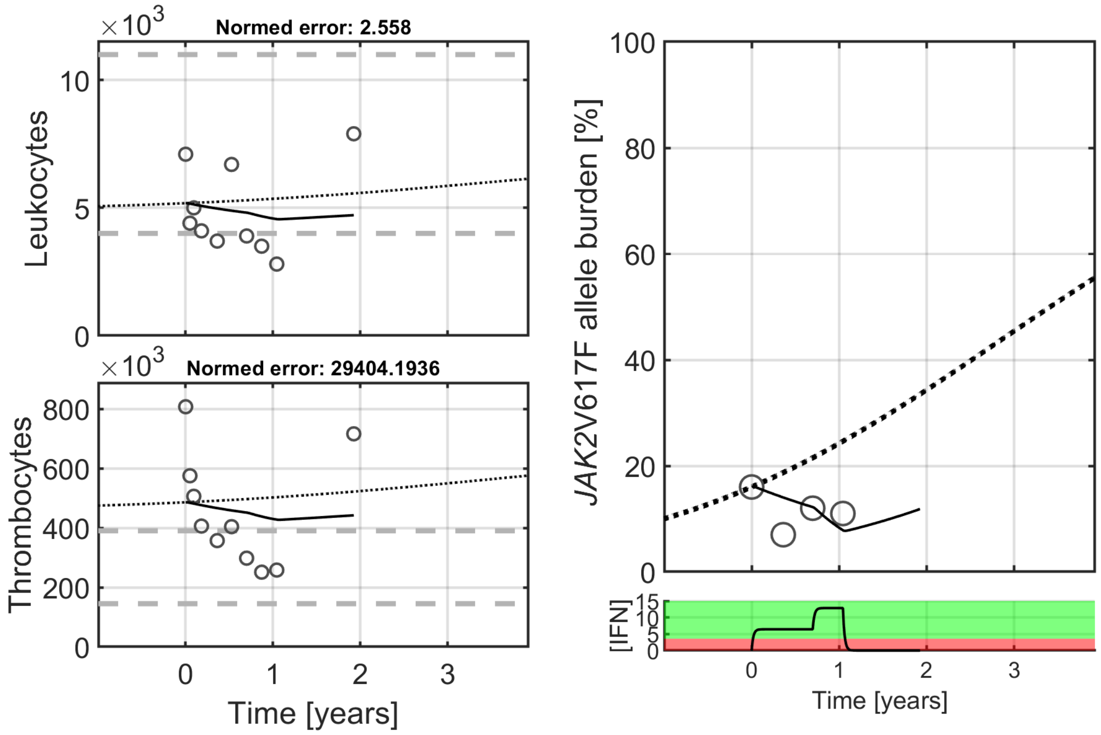

Patient P129

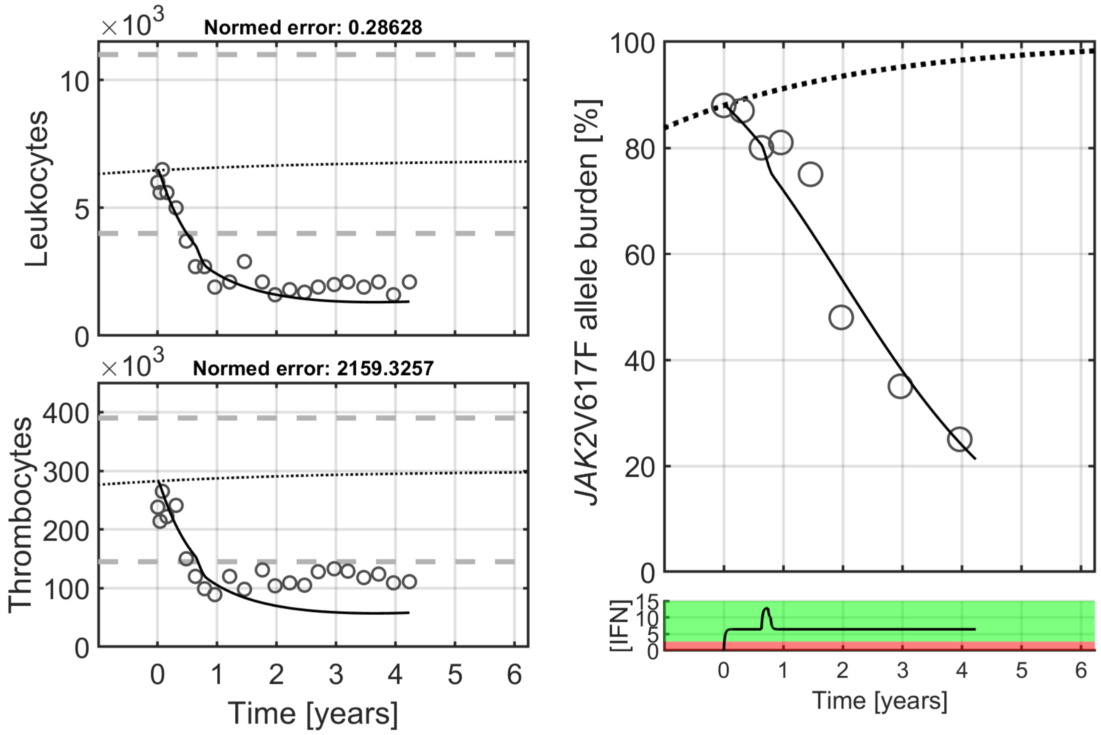

Patient P131

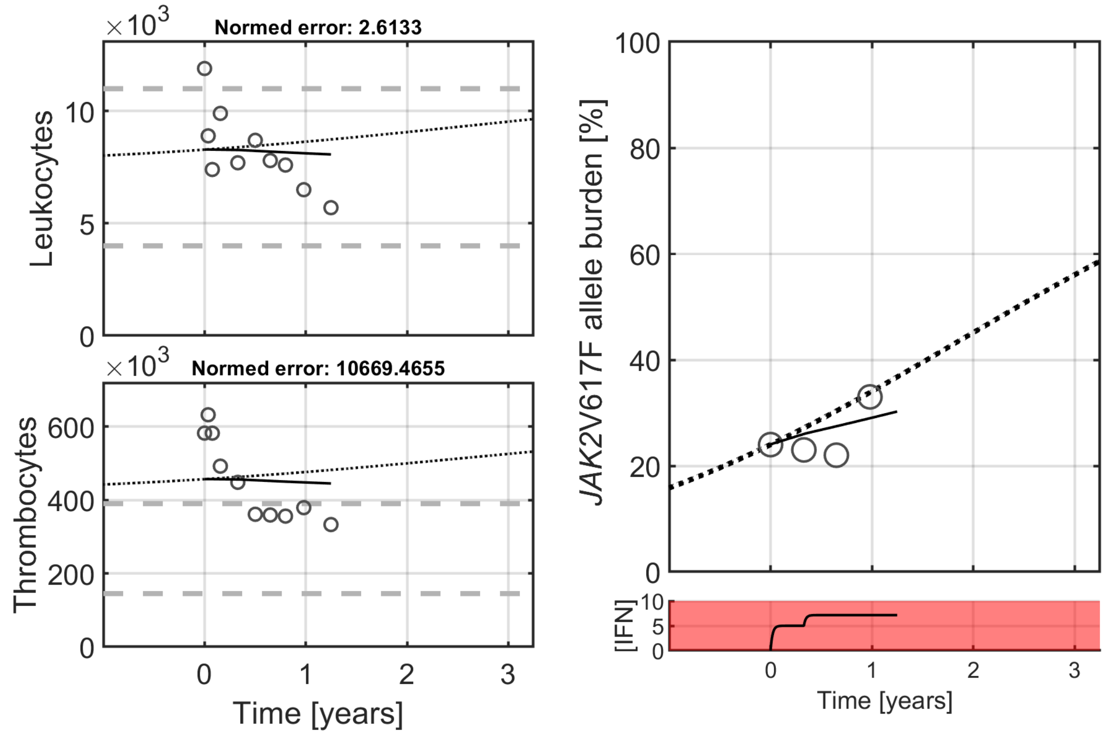

Patient P135

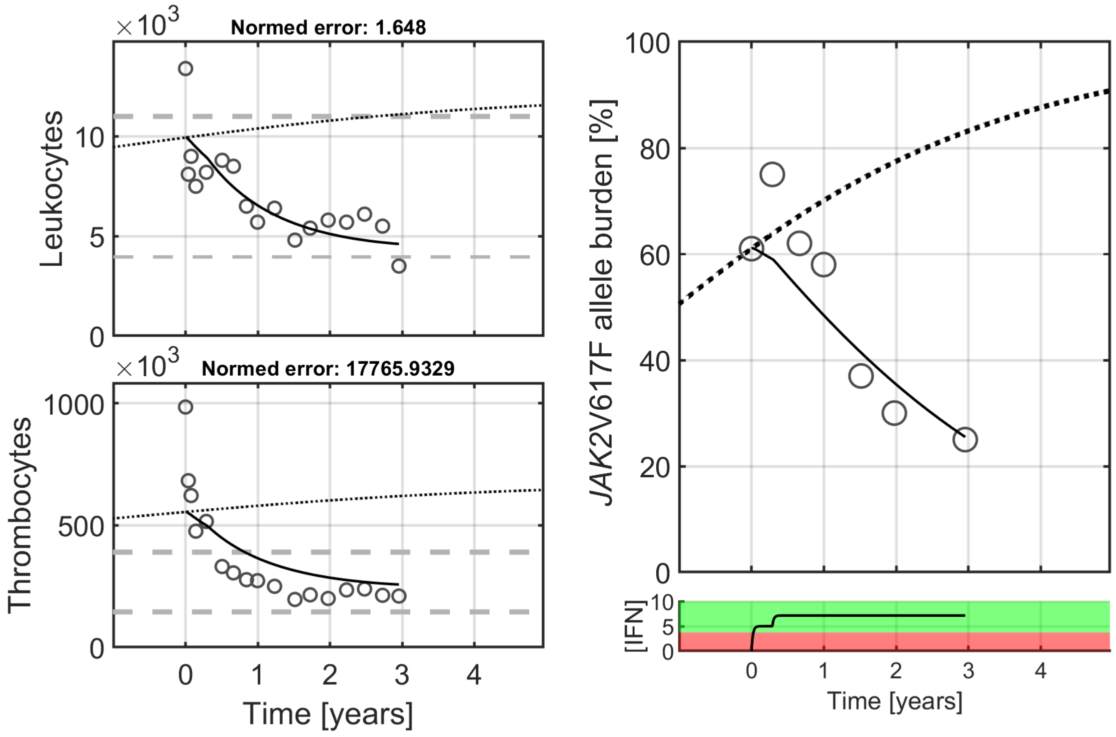

Patient P139

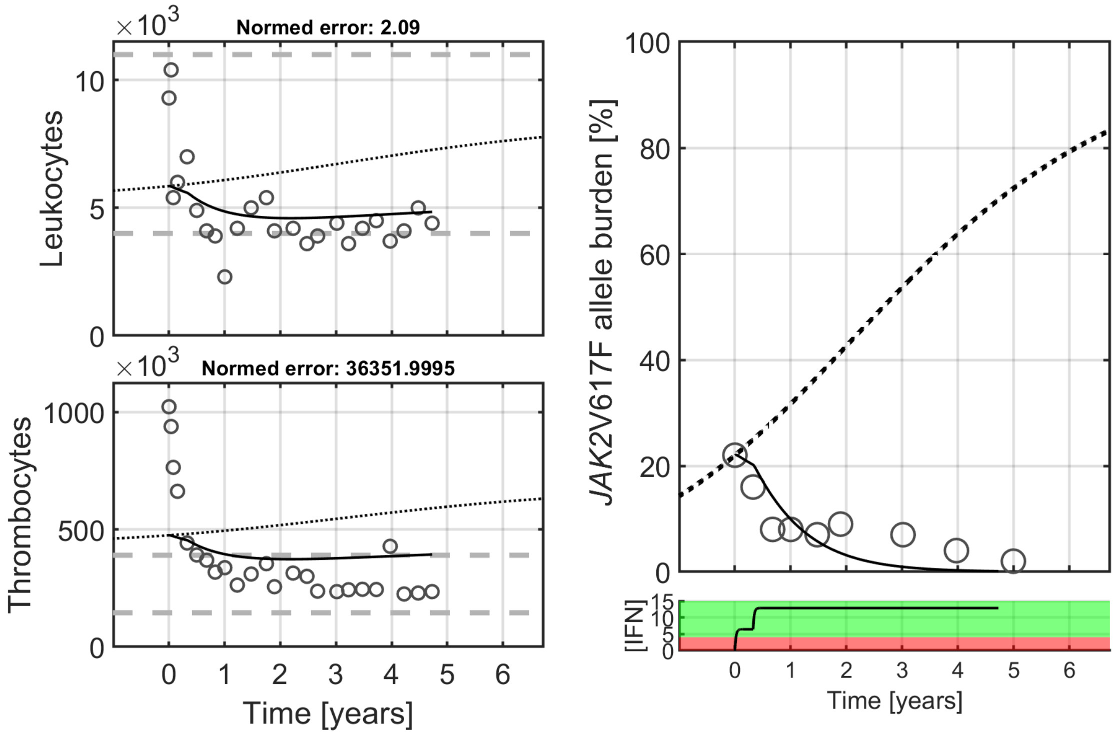

Patient P140

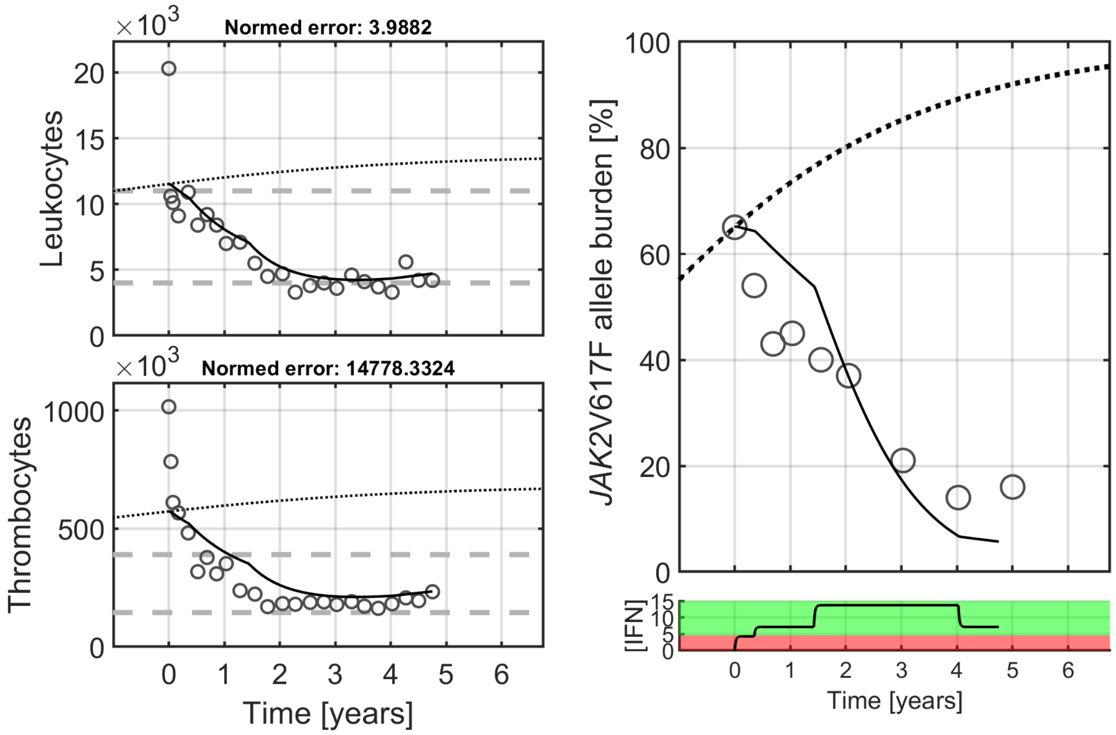

Patient P142

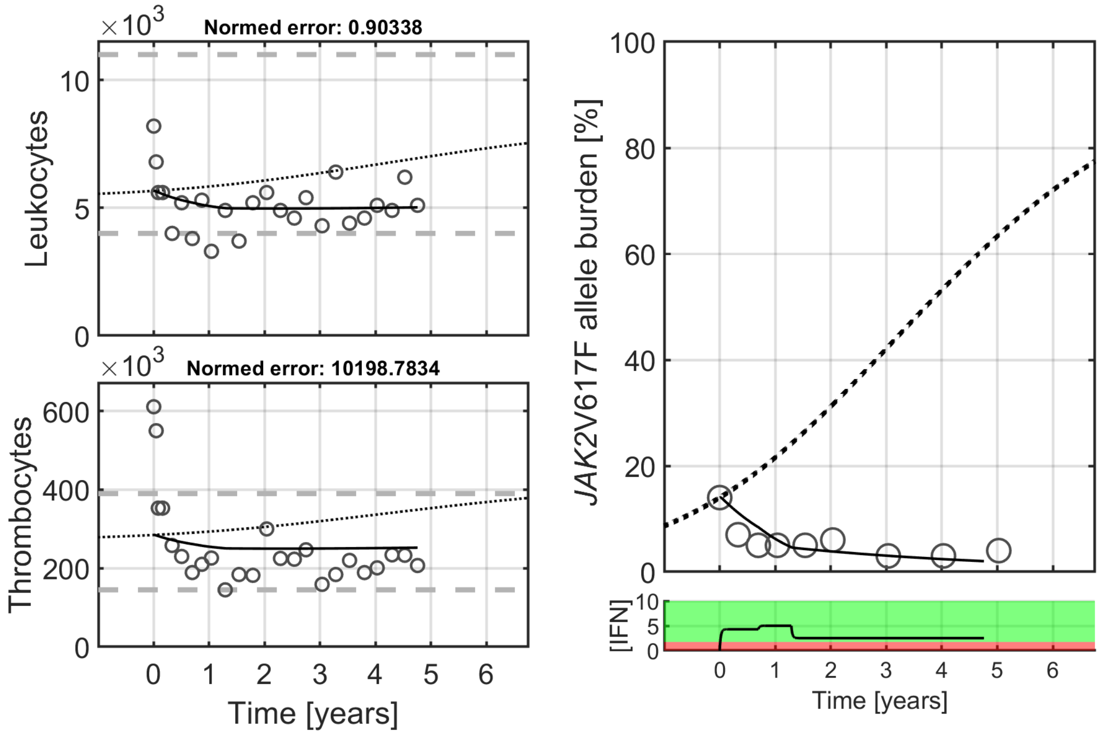

Patient P144

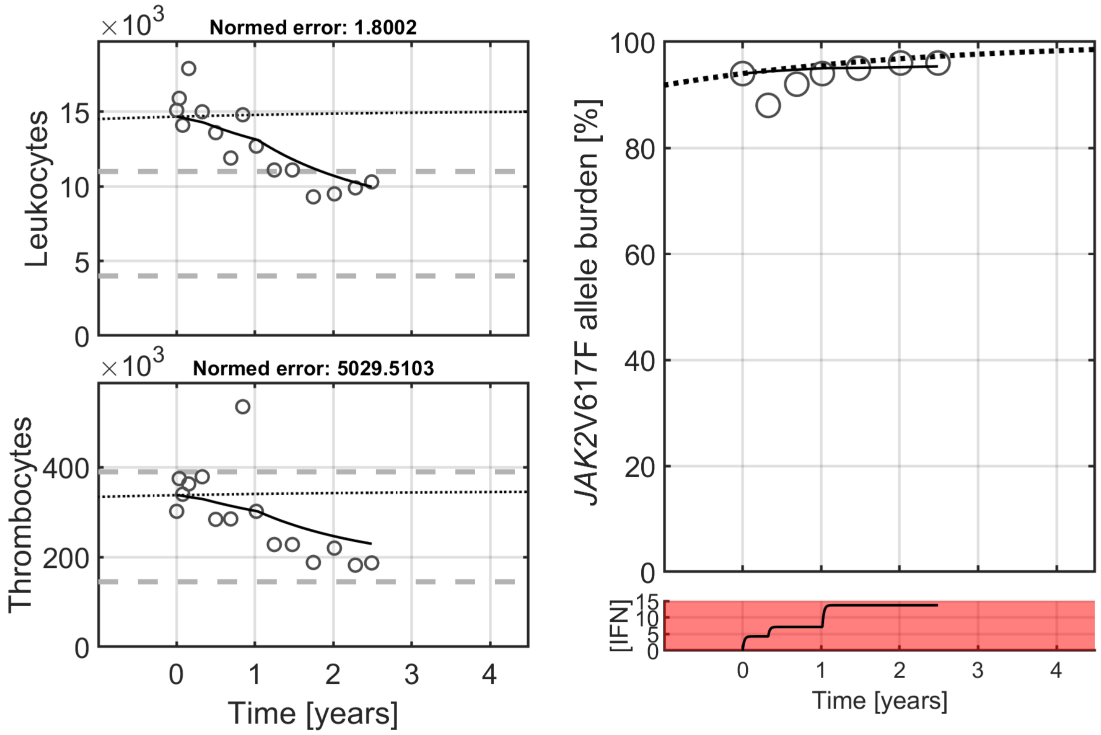

Patient P145

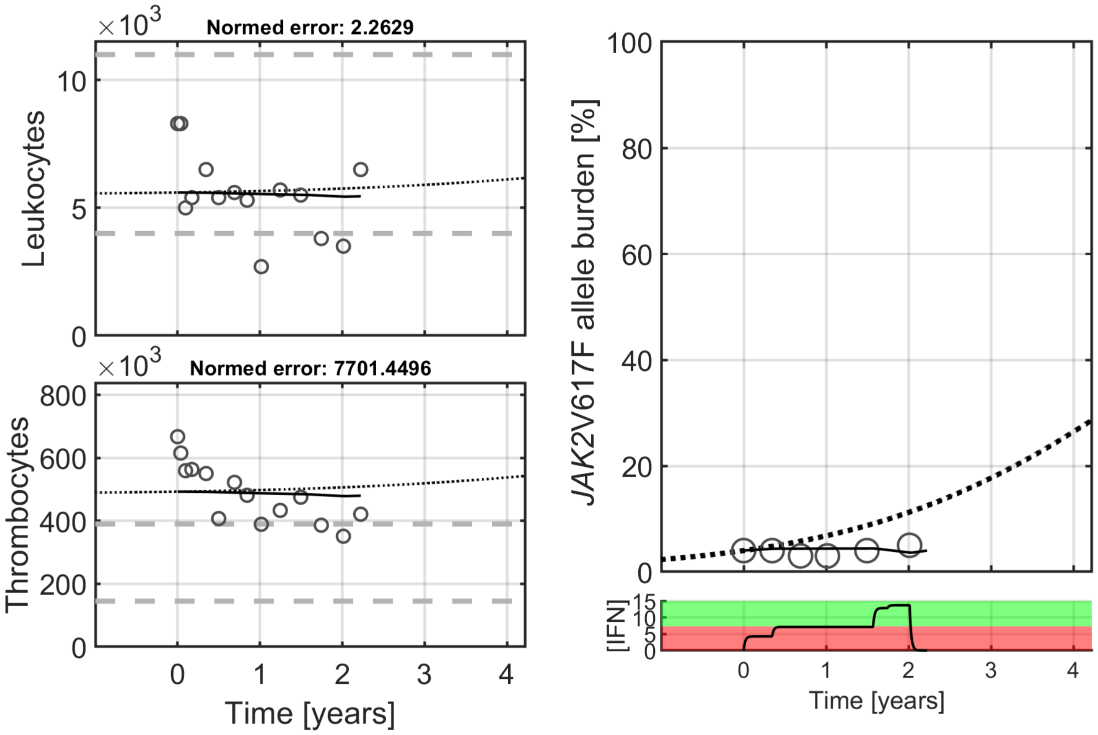

Patient P148

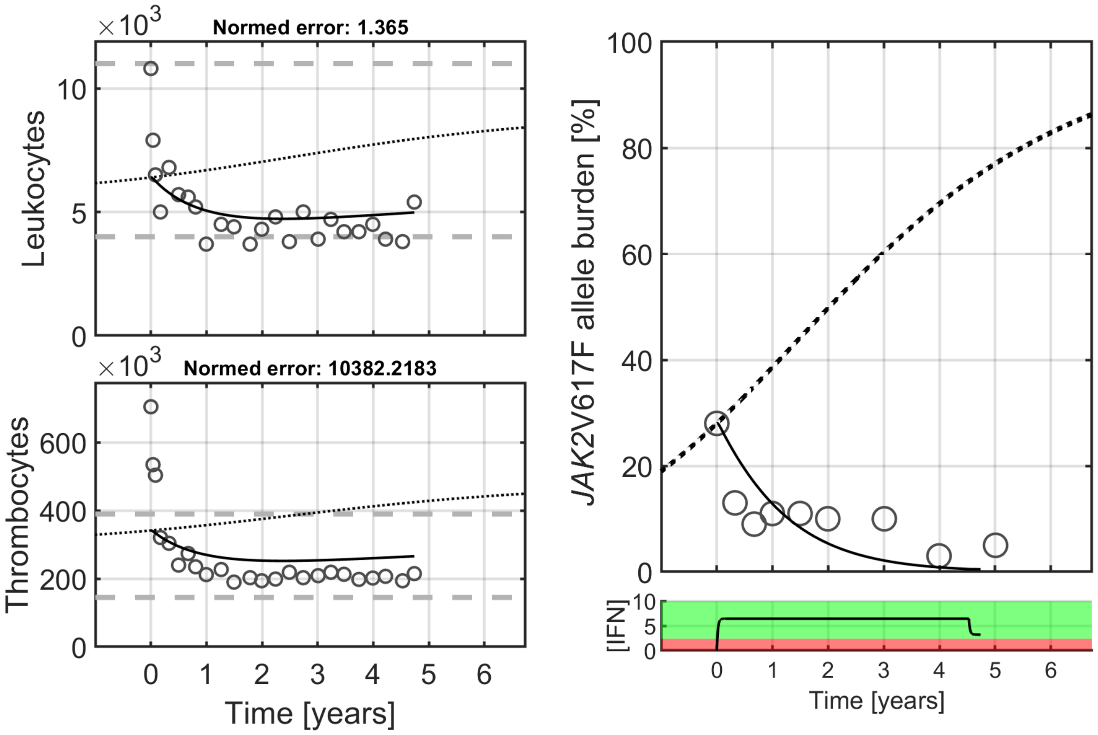

Patient P150

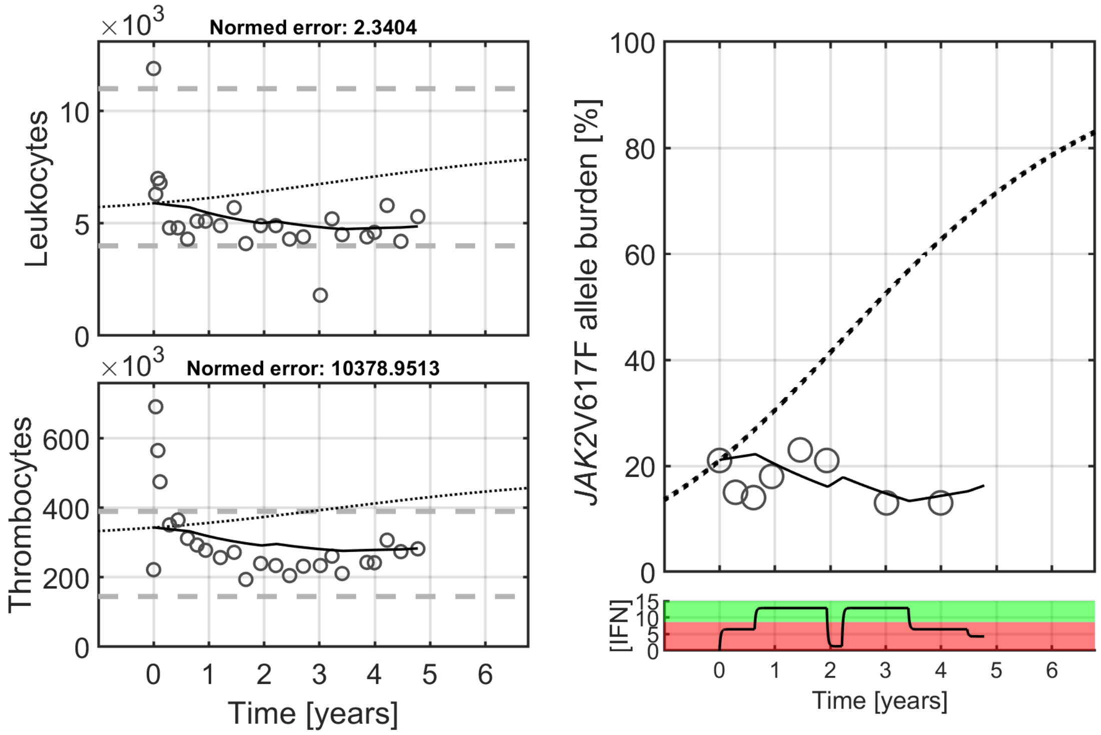

Patient P152

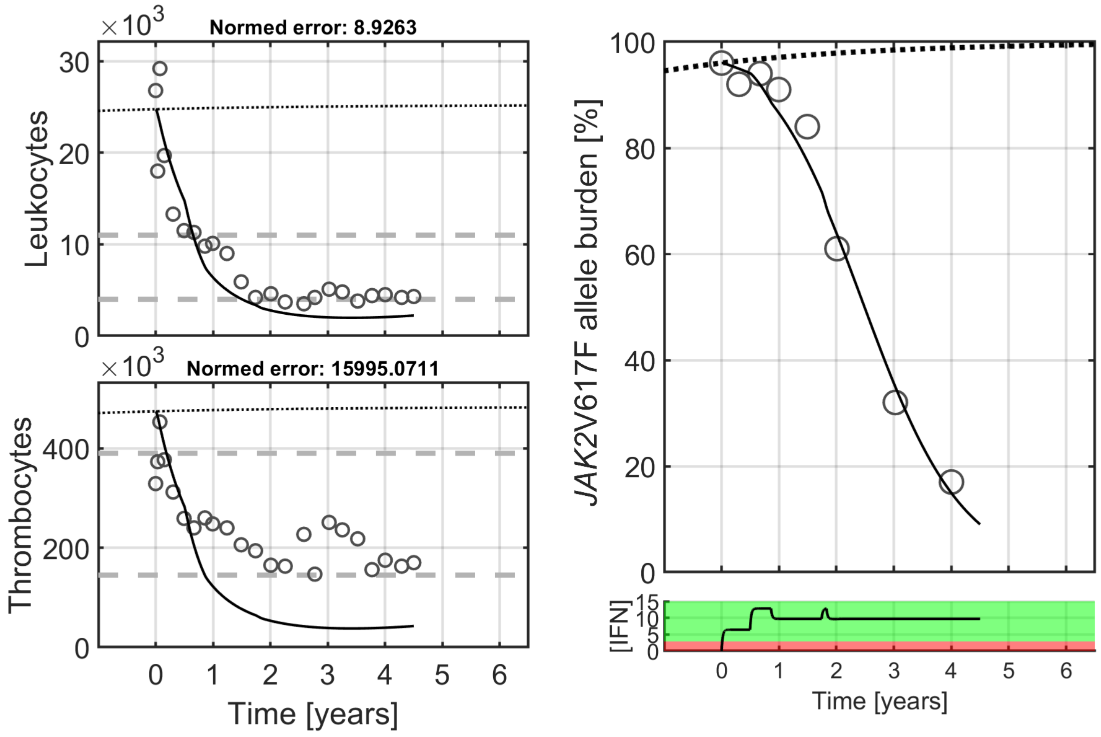

Patient P156

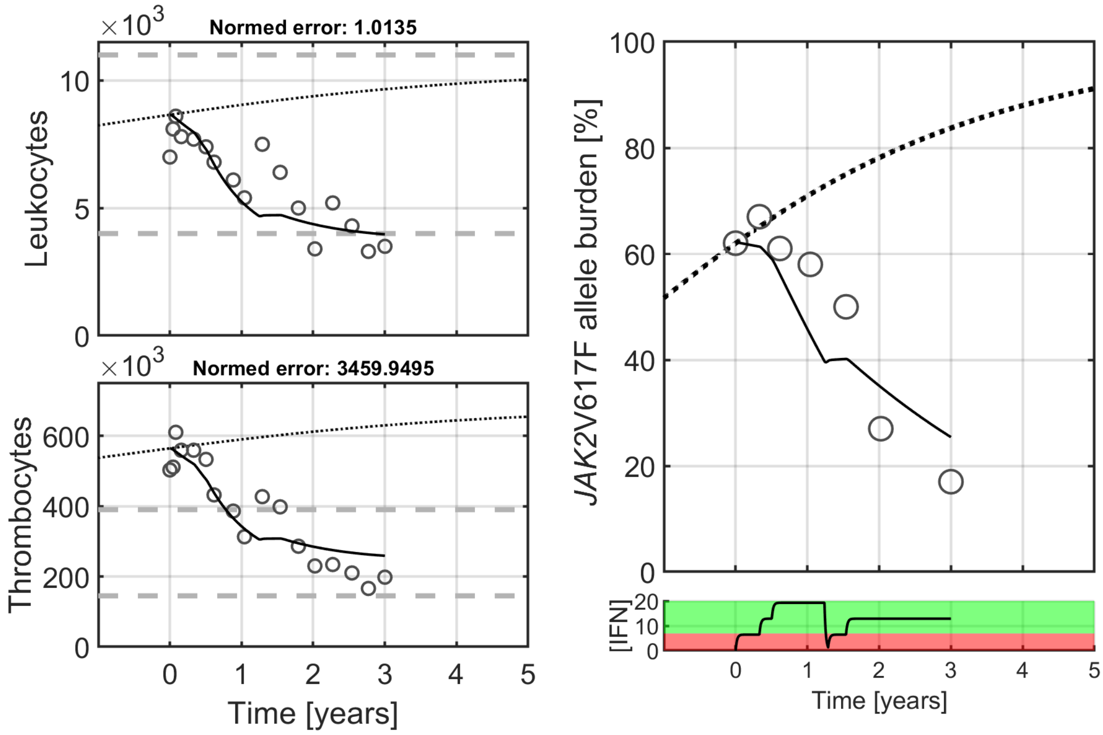

Patient P158

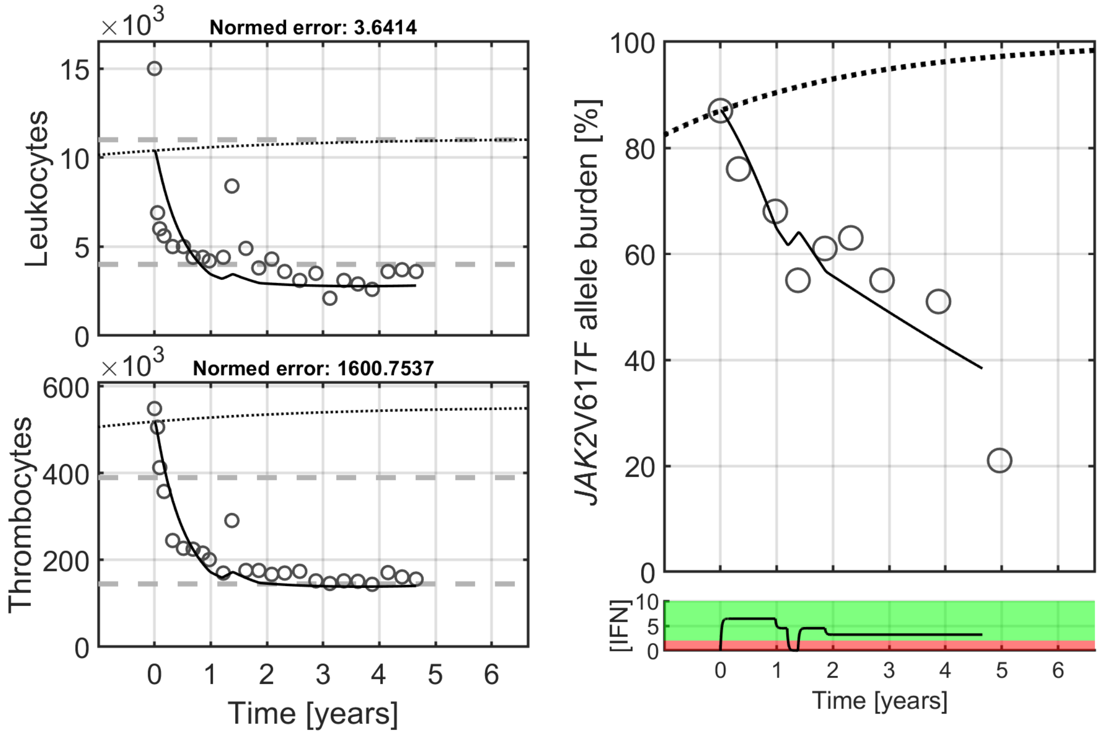

Patient P162

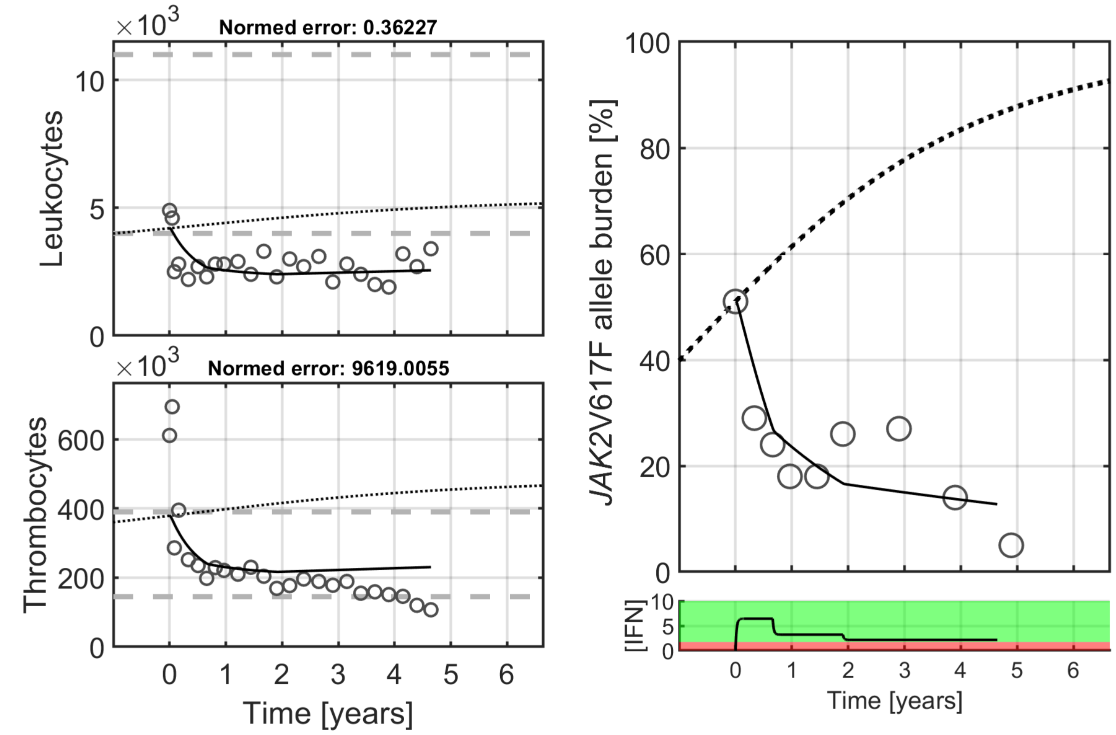

Patient P163

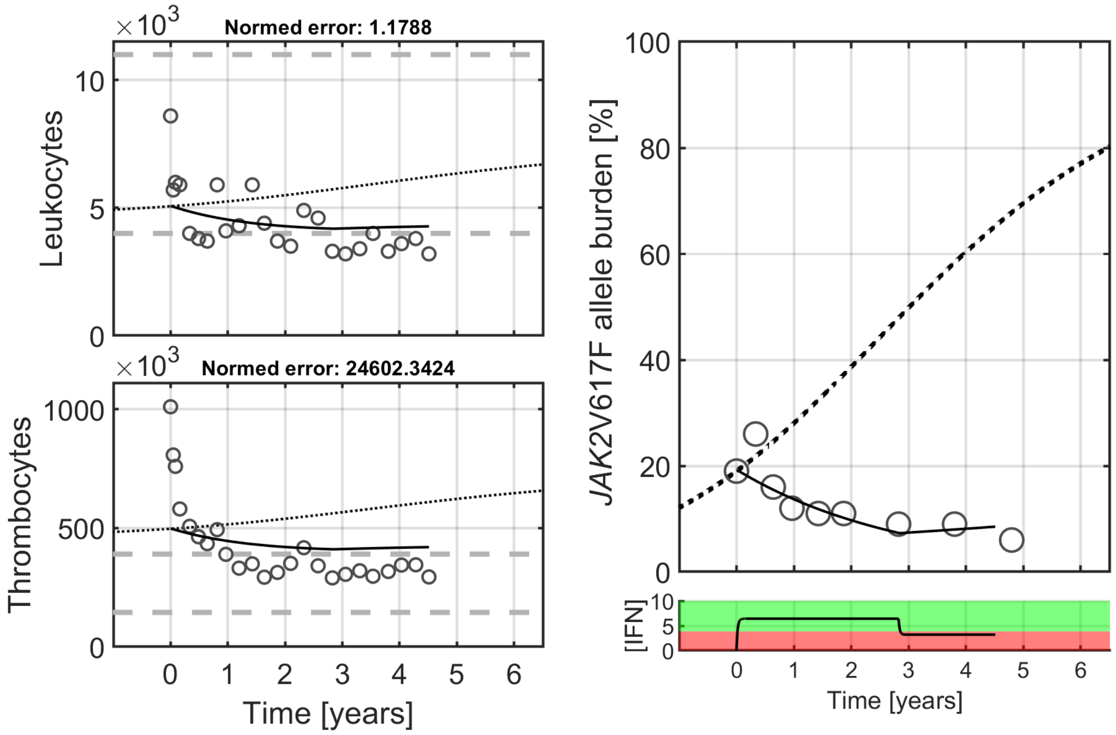

Patient P164

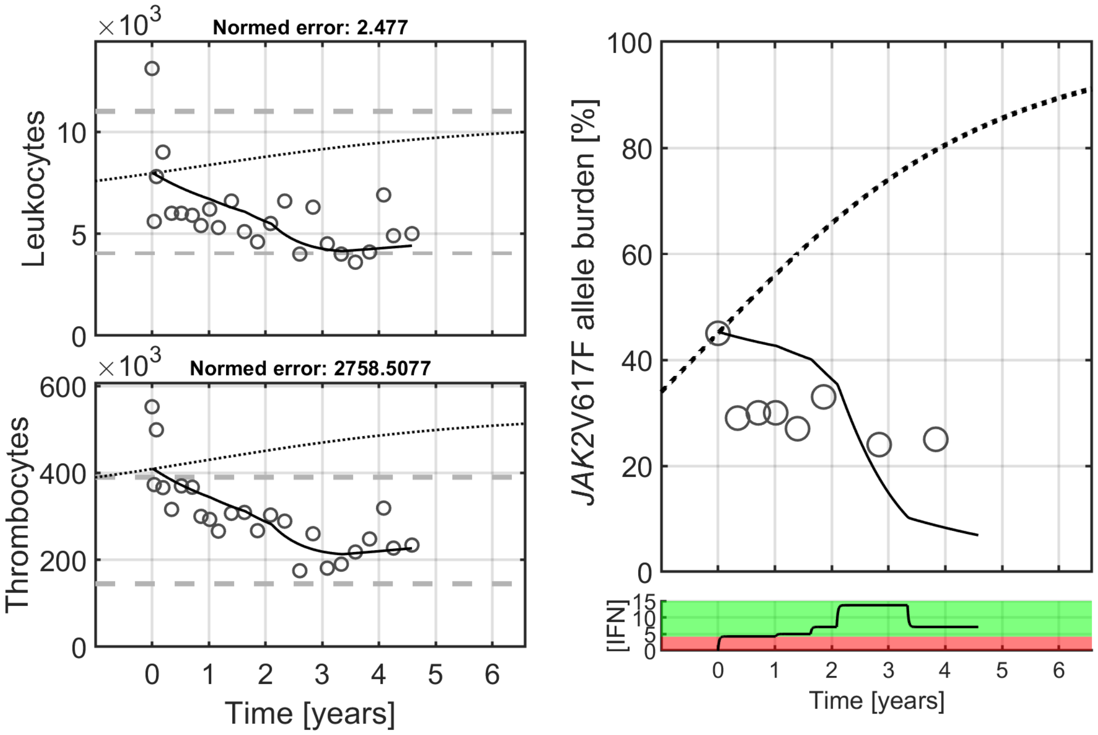

Patient P167

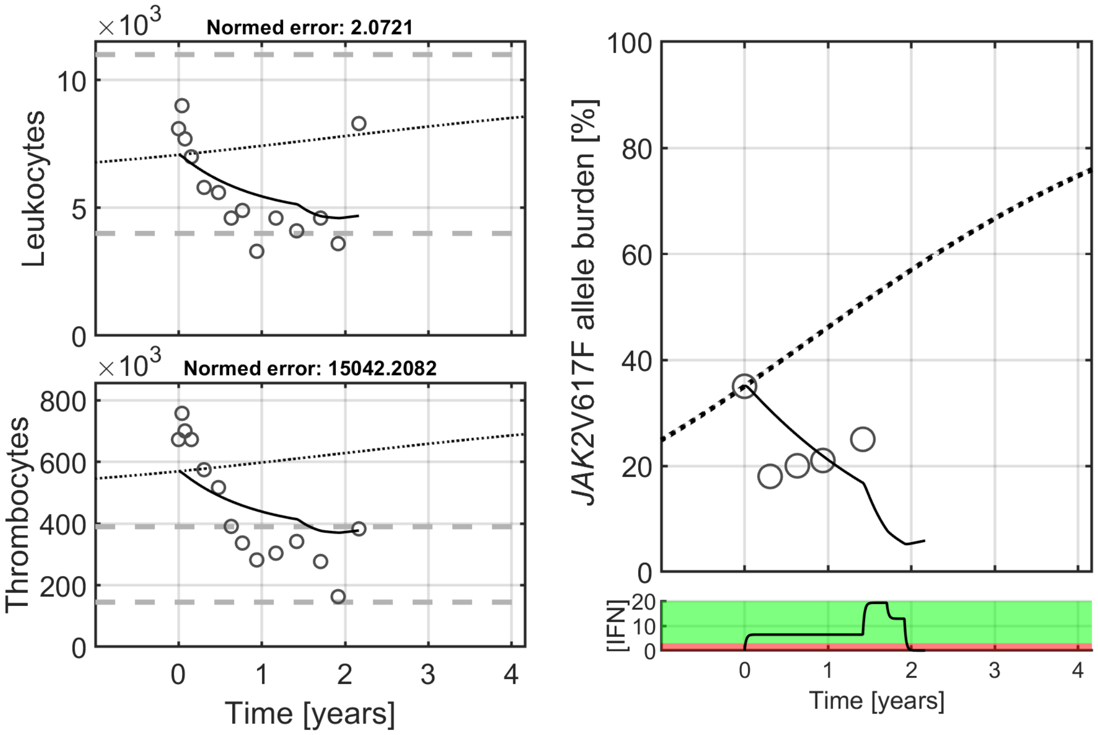

Patient P184

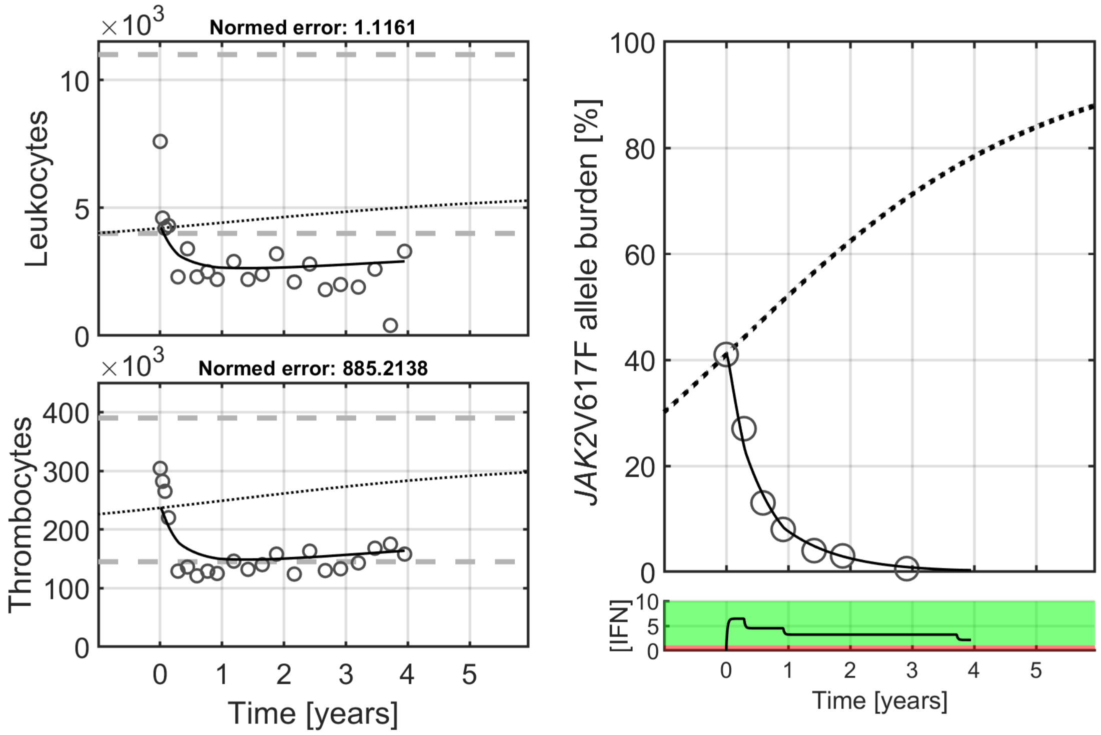

Patient P187

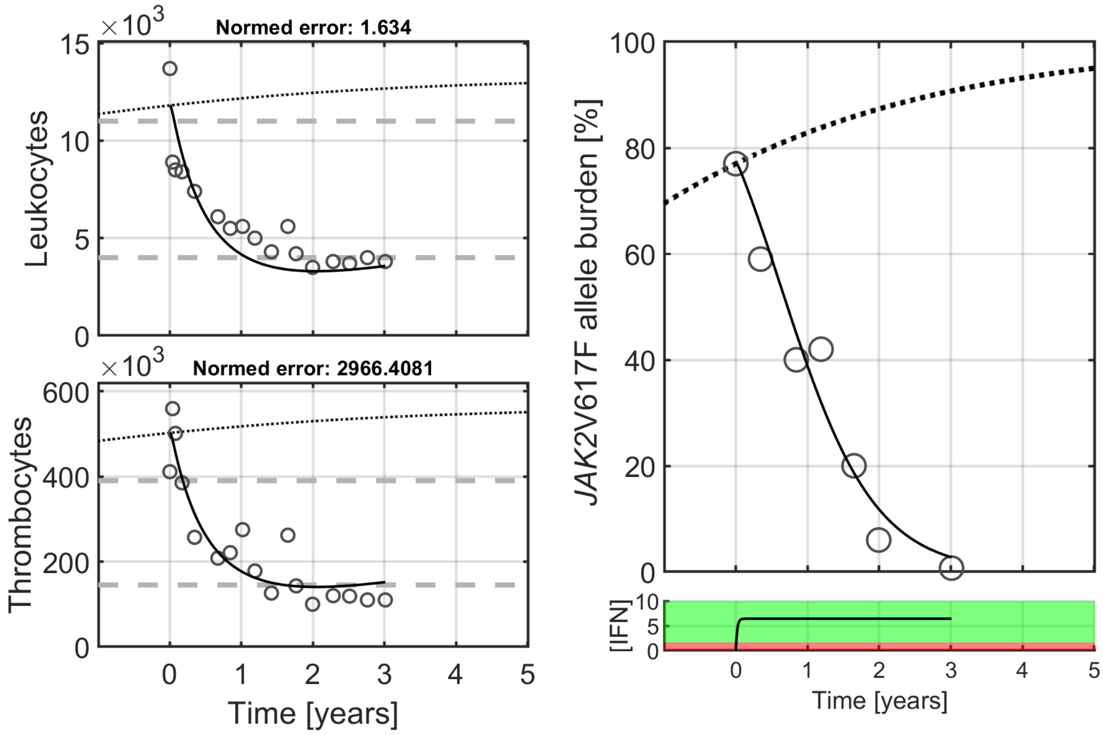

Patient P190

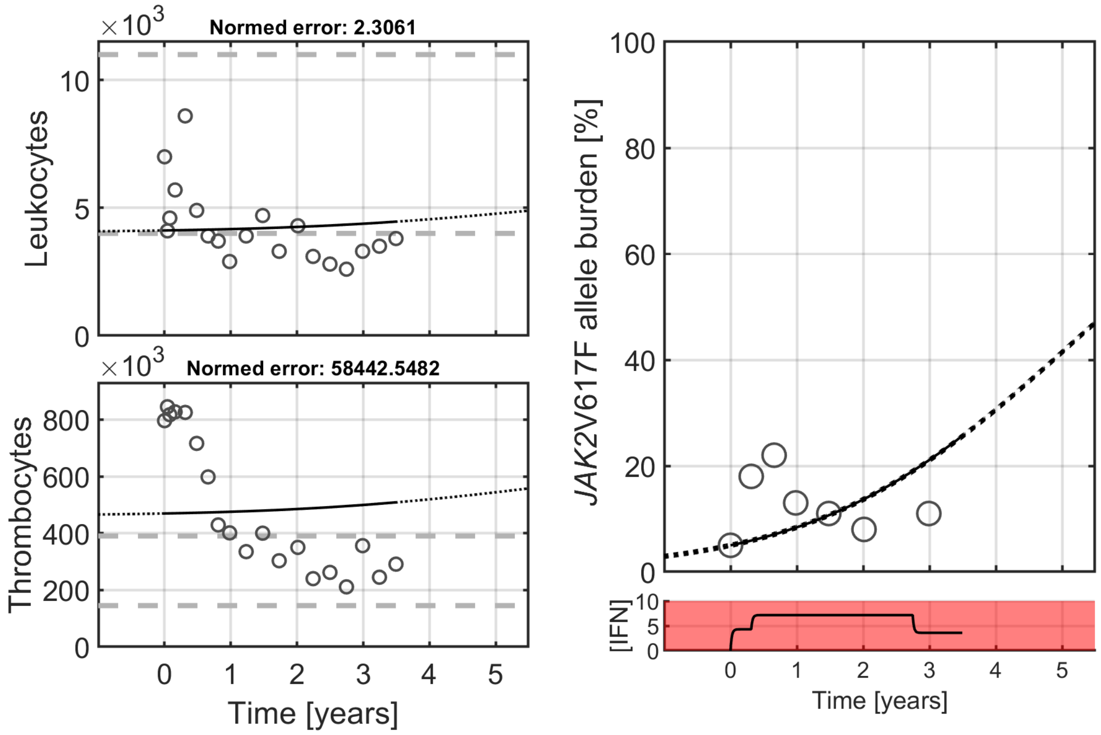

Patient P196

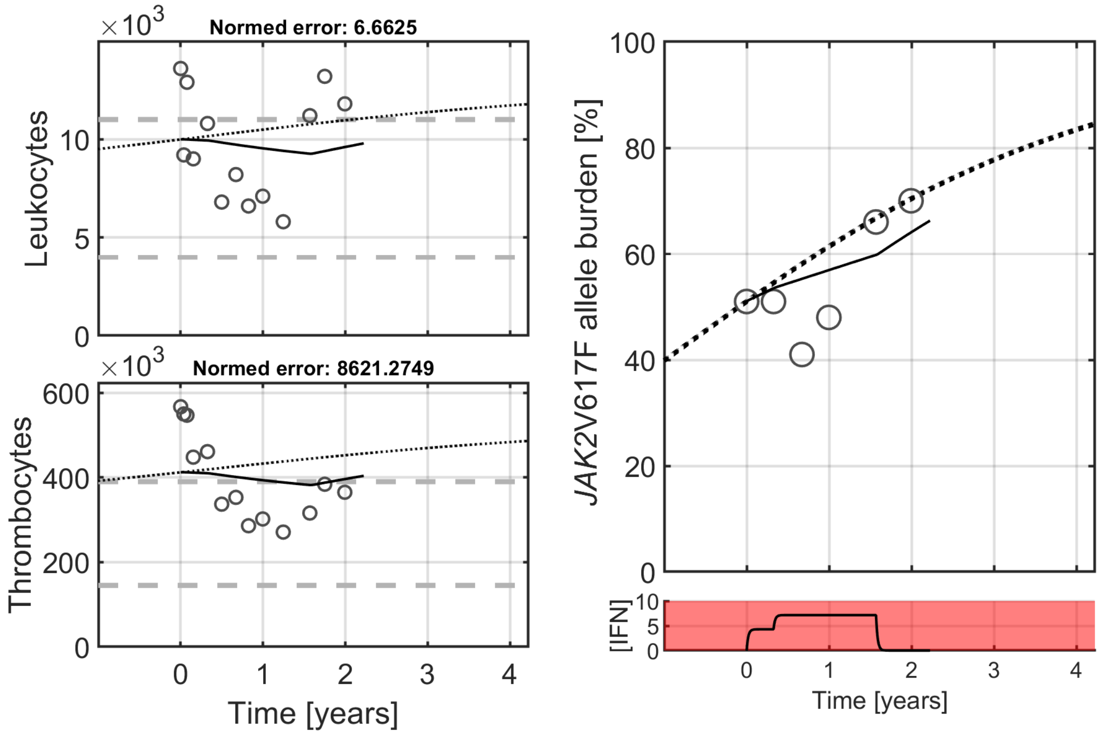

Patient P197

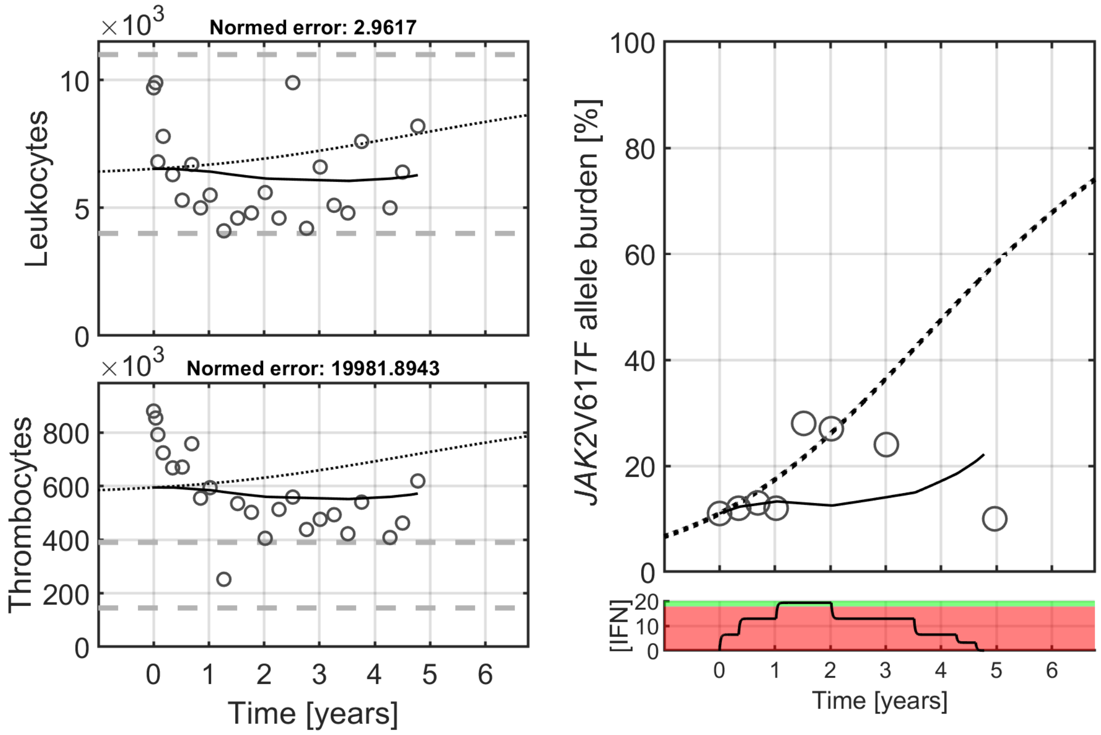

Patient P198

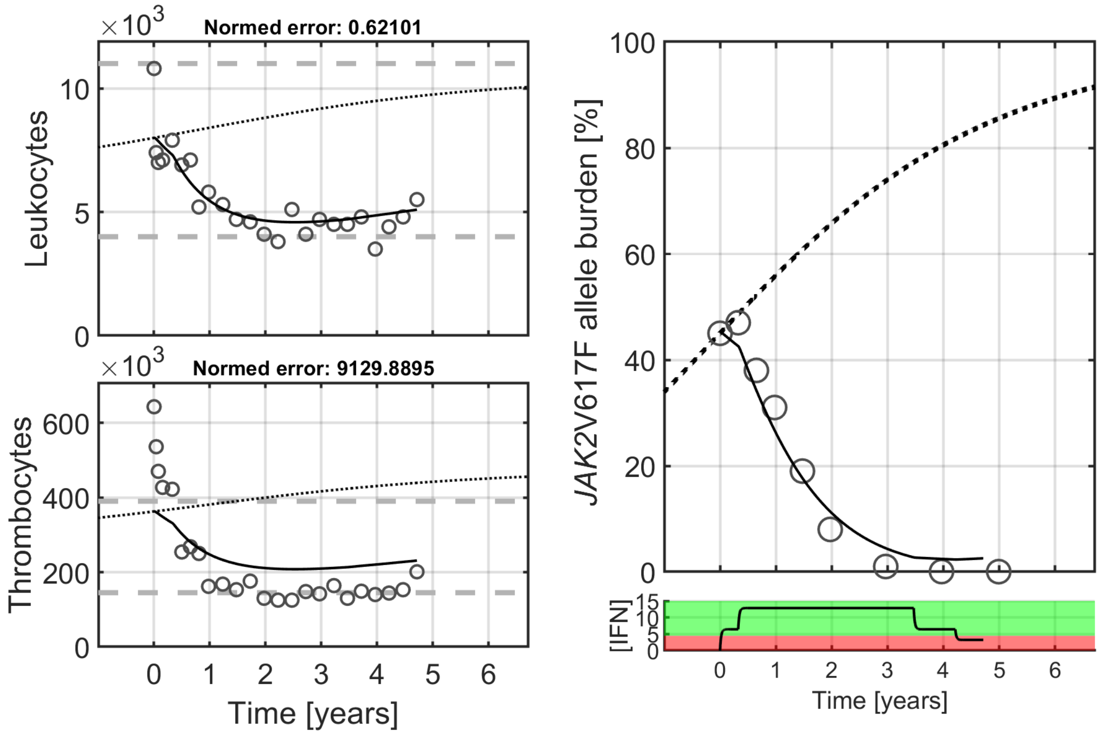

Patient P199

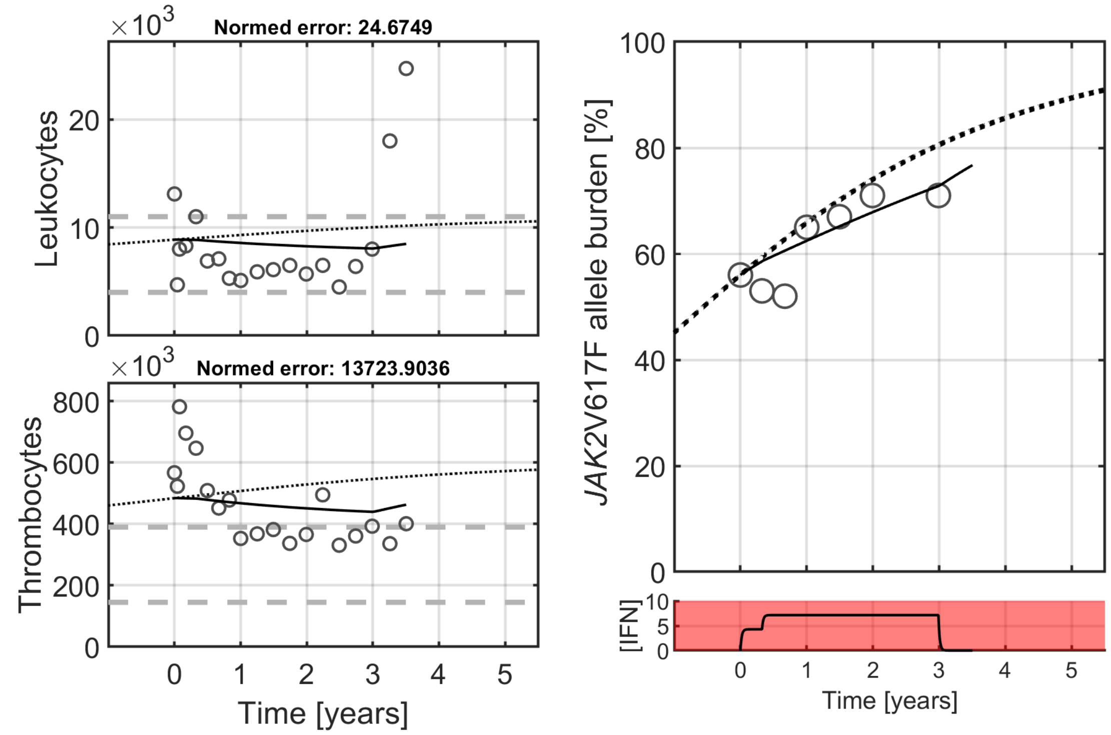

Patient P201

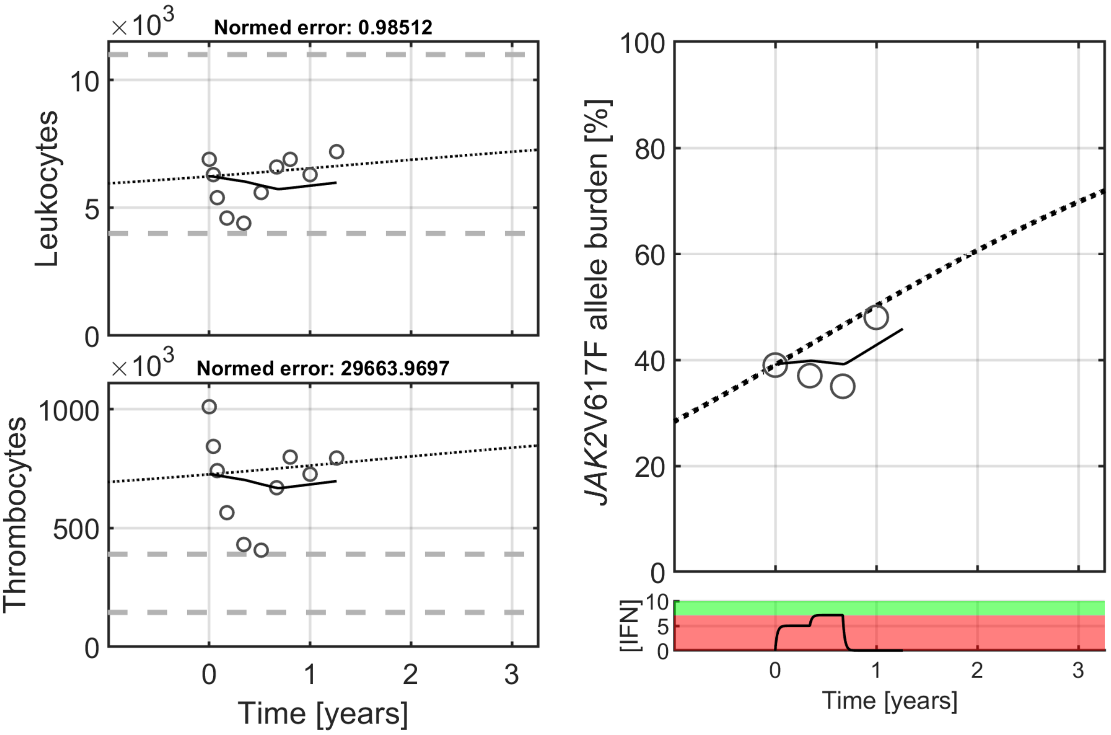

Patient P202

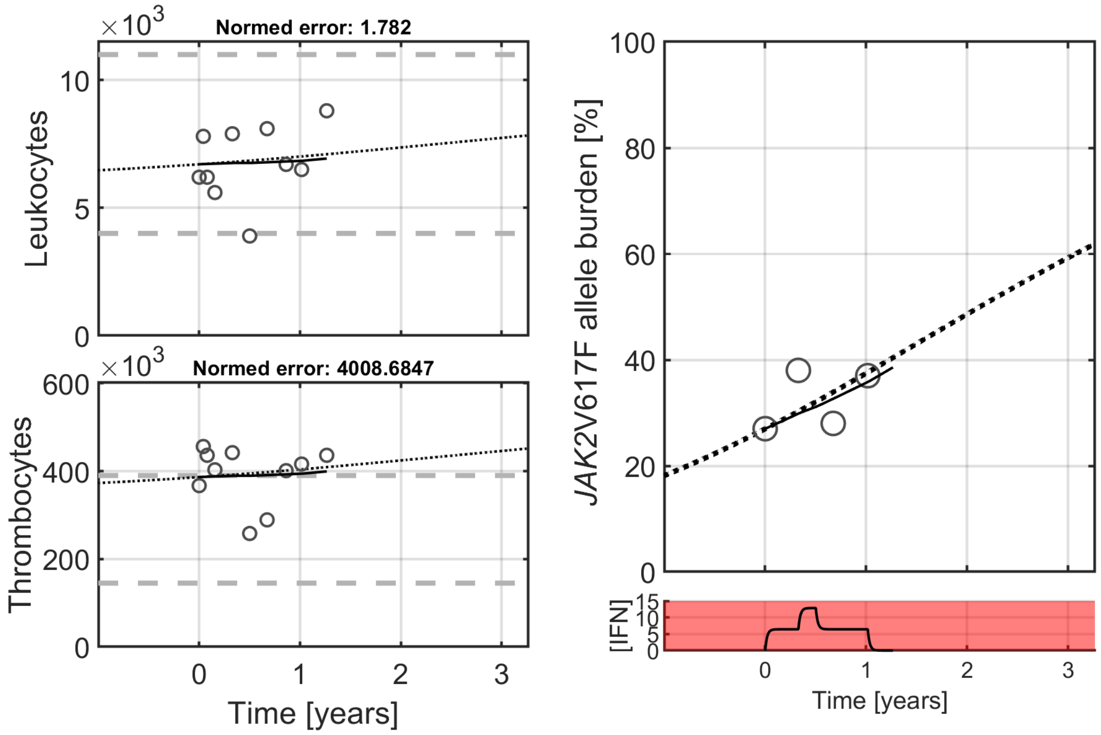

Patient P203

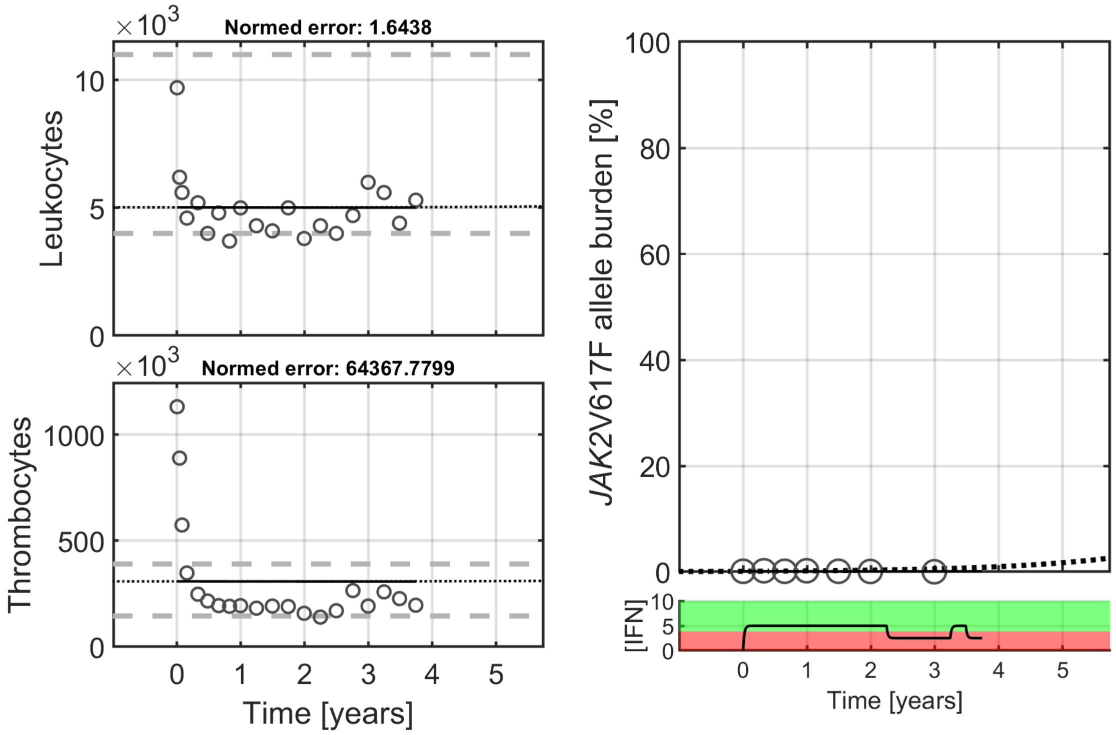

Patient P205

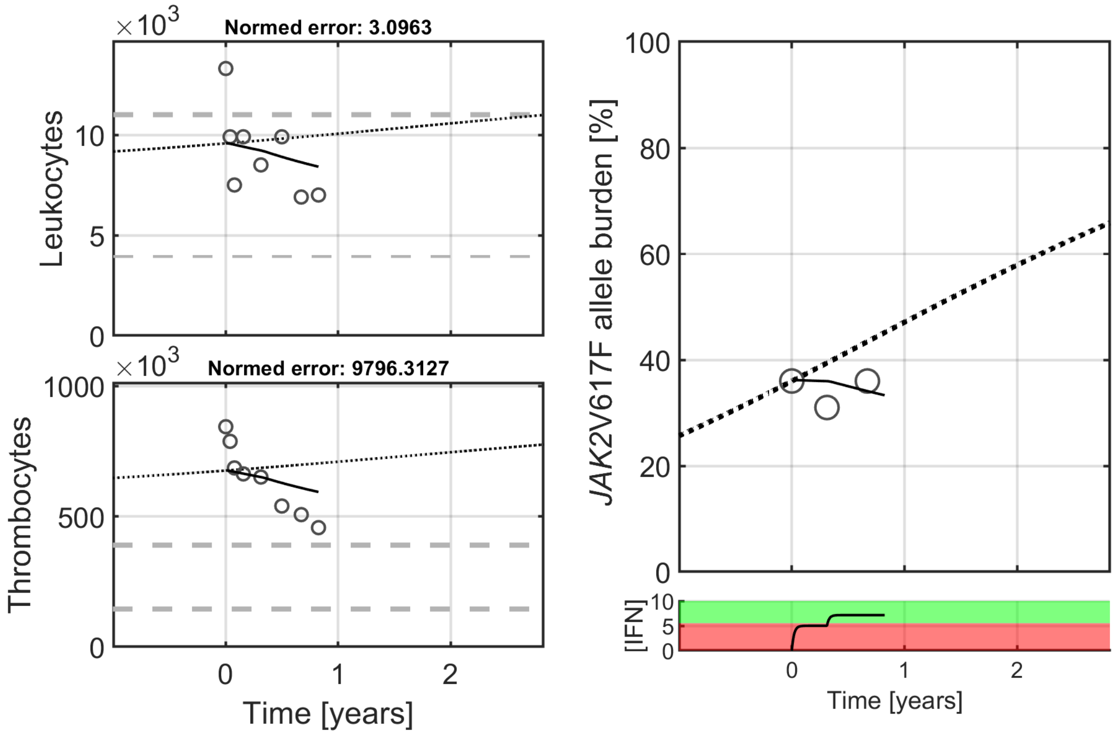

Supplementary material D: Predictive plots of *JAK2*V617F

In this supplementary material, we show figures similar to those shown in supplementary material B. The only difference is that here predictions of continued treatment is also considered.

The full black curves show the model prediction following the actual real dosing, as in supplementary material B. The blue and pink curve displays the hypothetical model predictions for difference dosing schedules, see the bottom panels. The blue curve displays a dosing schedule where the current treatment at the last grey data-point is continued (i.e. if the patient was administered an average daily dose of 5 μg of IFN at the last data-point used in the fit, the simulation is continued with 5 μg of IFN indefinitely). The pink curve displays the model prediction of a treatment-stop scenario, in which treatment is discontinued after the last grey data-point. As total extinction of malignant stem cells is only approached asymptotically, treatment-stop will always lead to future disease growth in the model. If the malignant stem cell counts are sufficiently low however, the time before symptoms arise can be far longer than the average life-span. The bottom panels show both the PK-modelling of the blood-concentration of IFN based on the actual dosing, as in Supplementary Material B, as well as the modelled hypothetical concentrations. The color-coding corresponds to the upper panel, with blue displaying an unchanging dose and pink displaying a treatment-stop scenario.

At the top of all figures, a goodness-of-fit-metric of the best fit is shown, defined as the sum of squared errors, divided by the number of points used in the fit. A low number signifies a good fit. Only the error of points used (the grey points) were included in the error estimate.

Patient P002

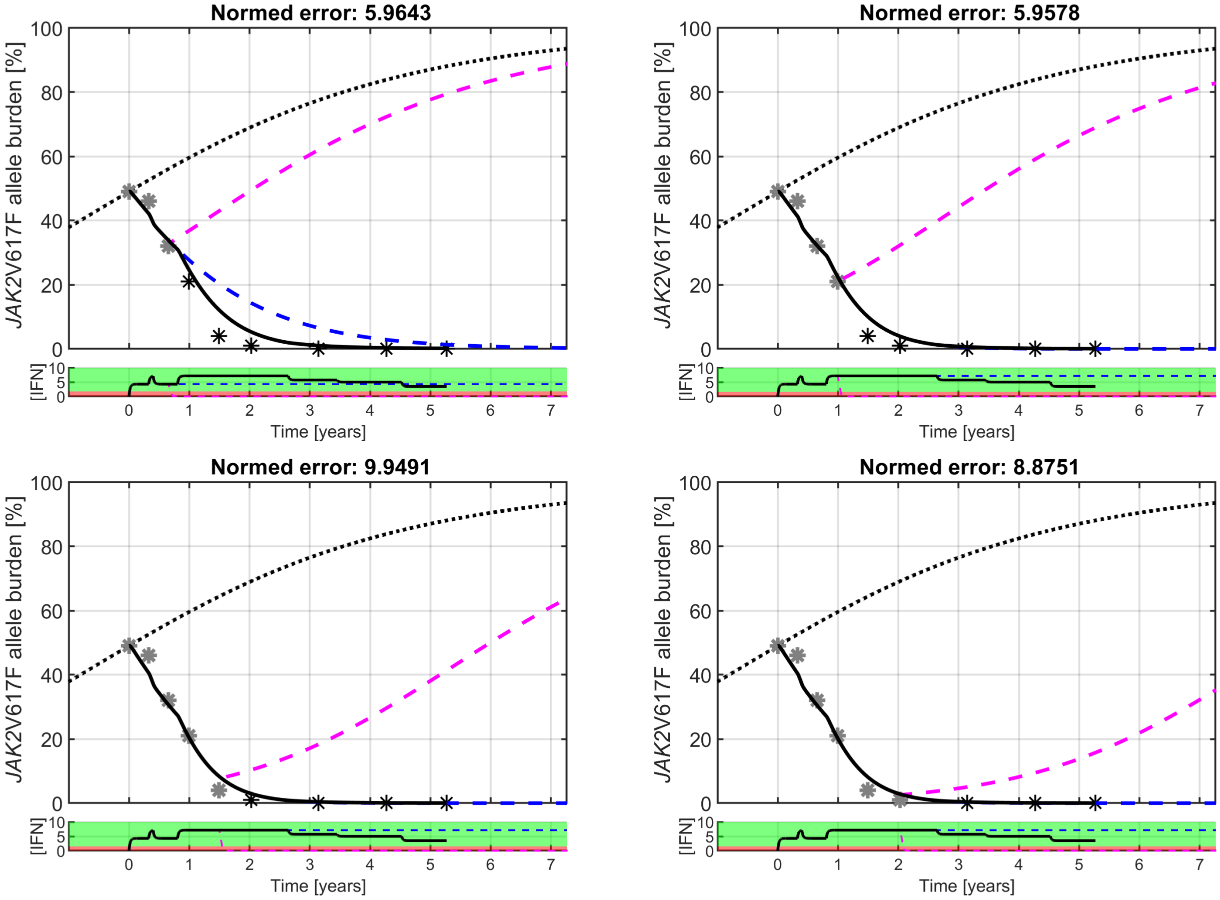

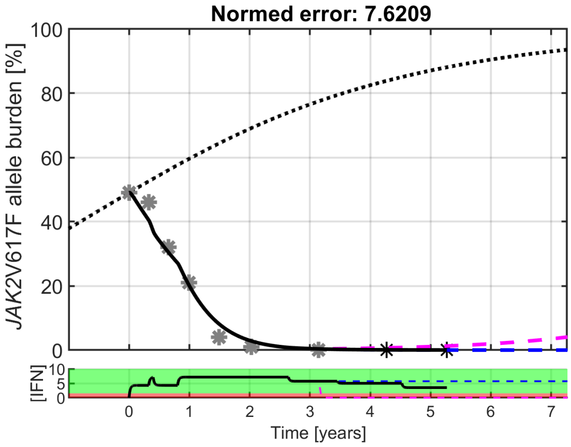

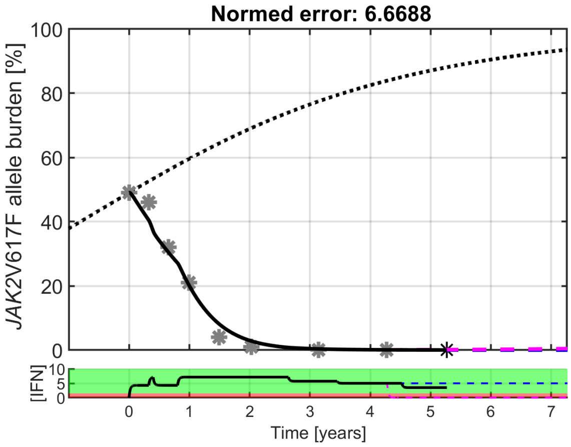

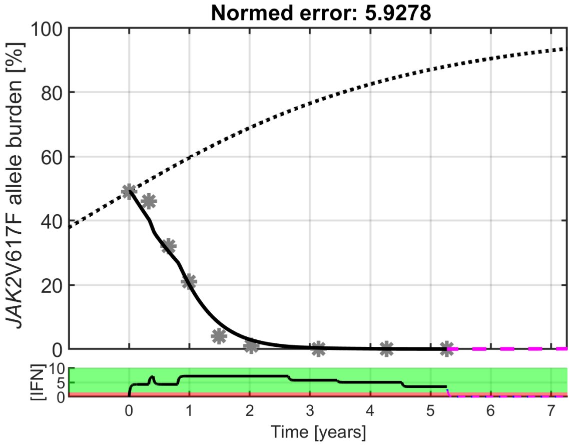

Patient P003

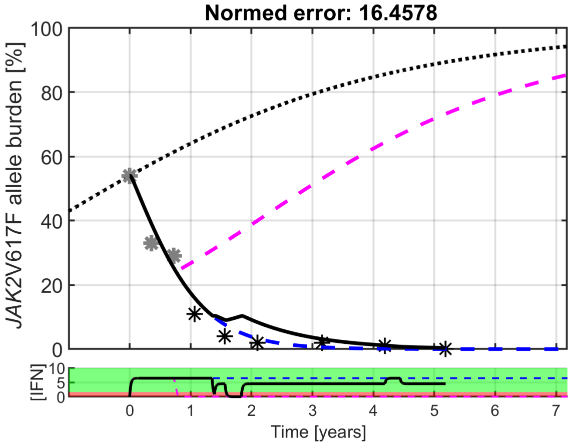

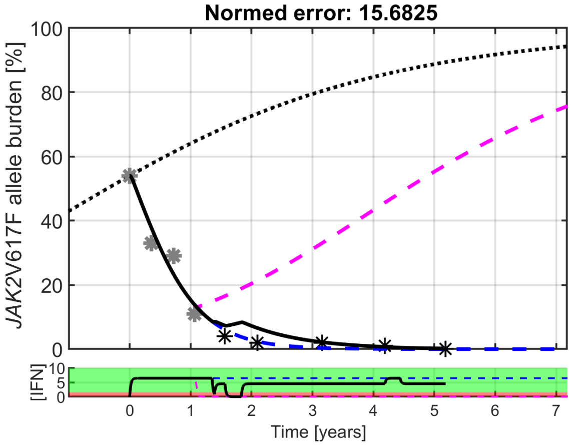

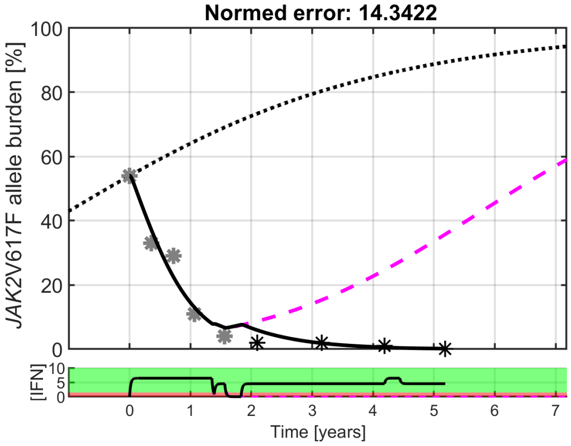

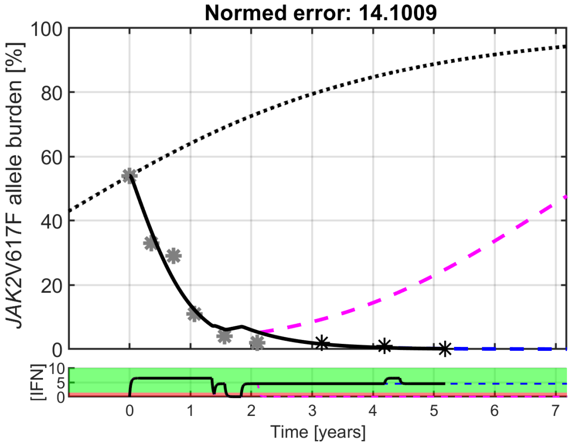

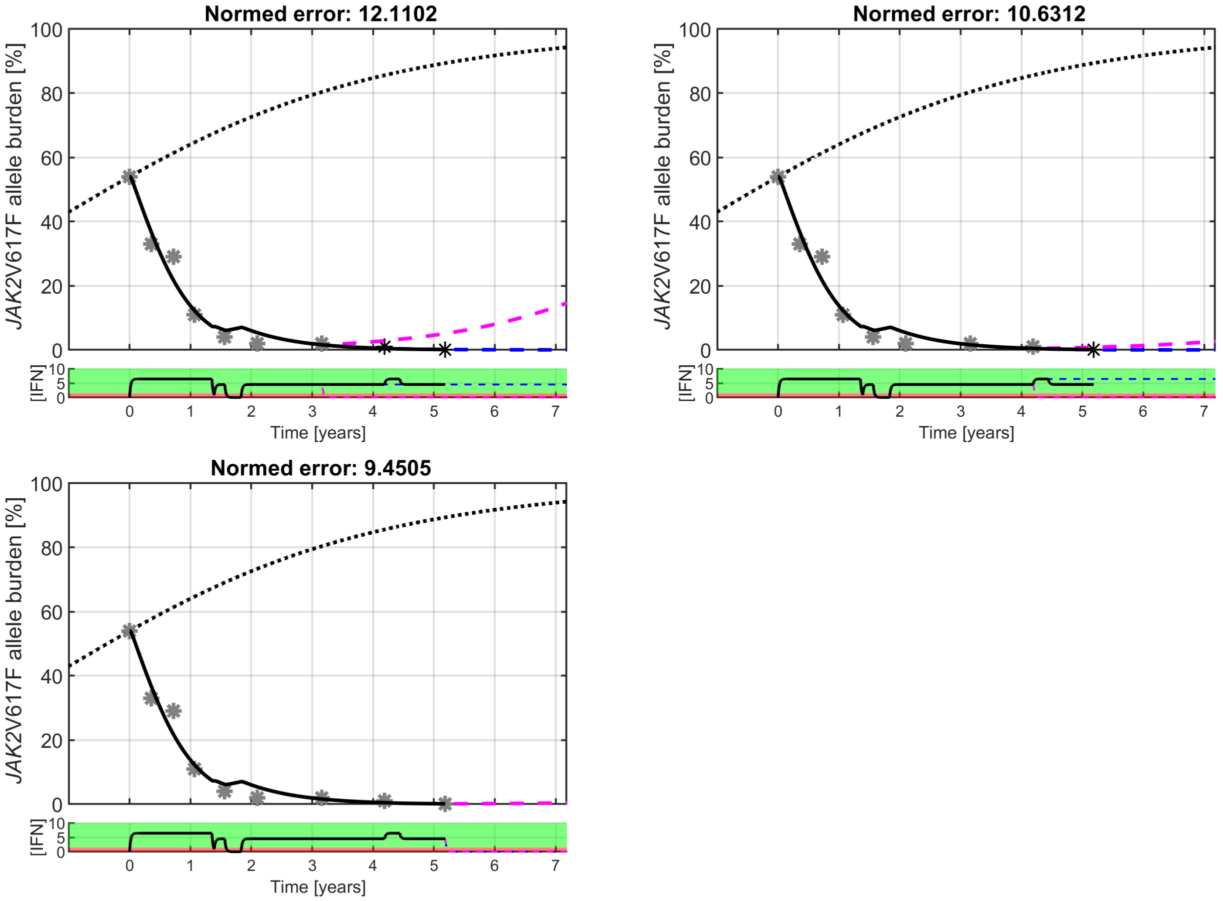

Patient P004

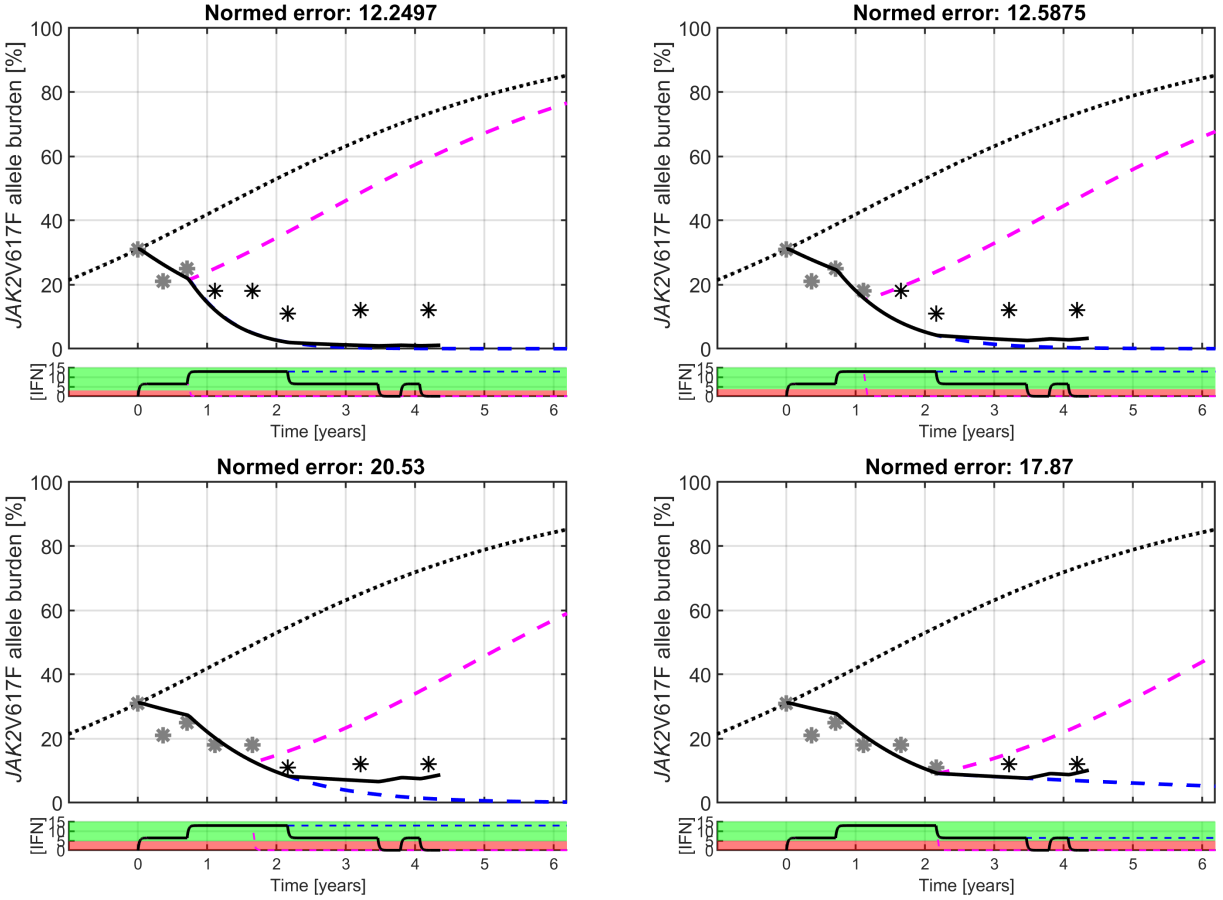

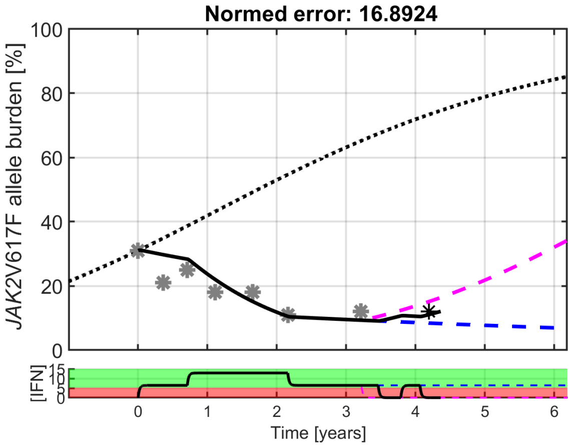

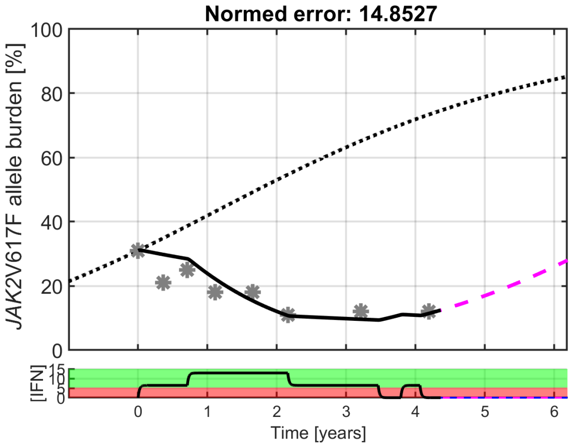

Patient P006

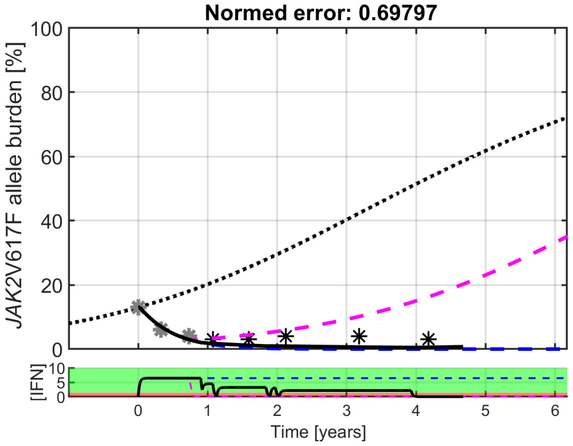

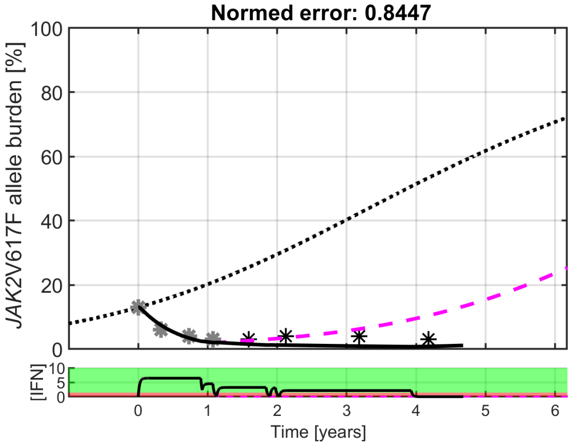

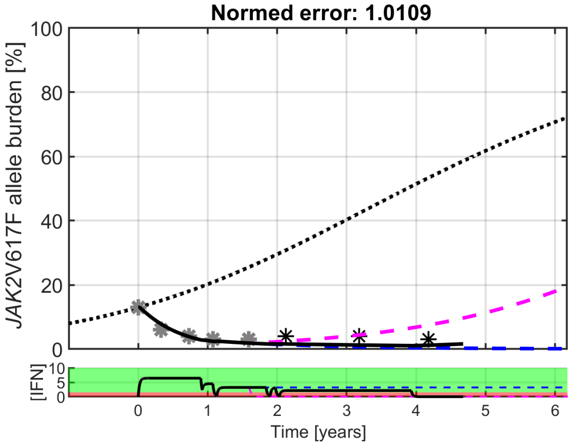

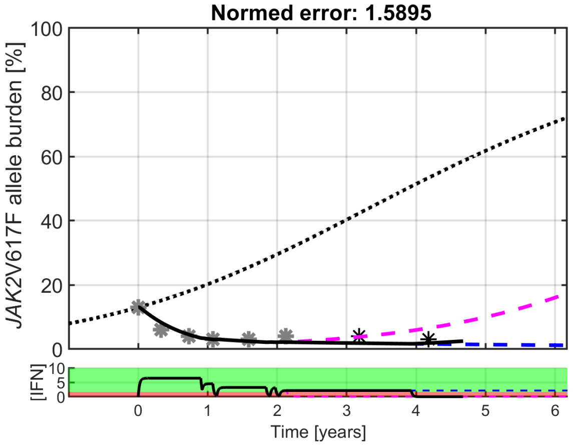

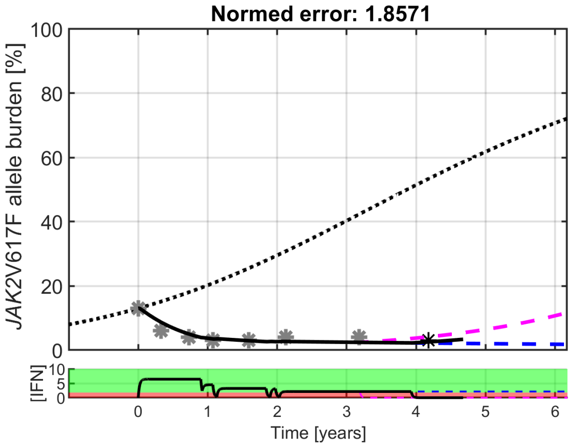

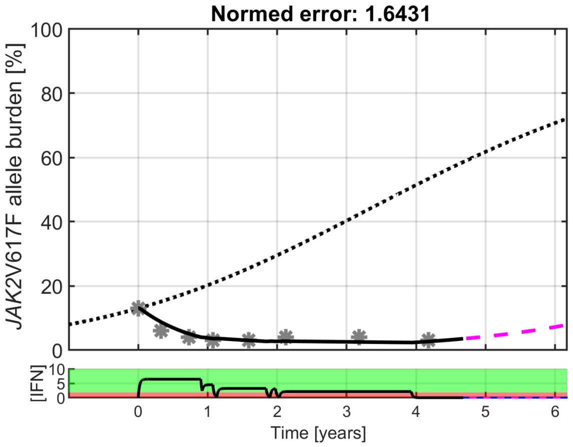

Patient P011

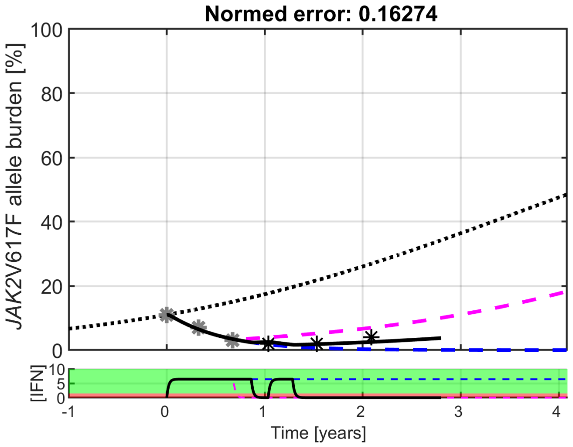

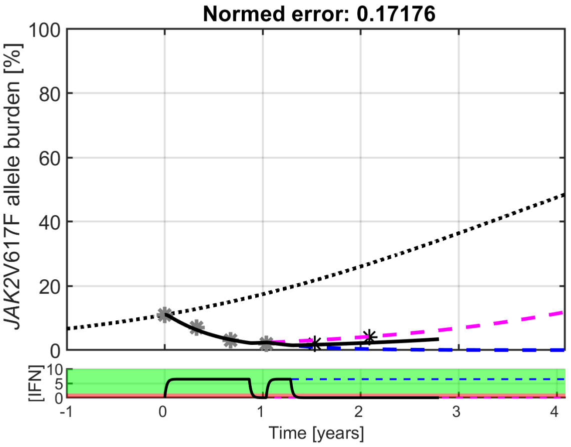

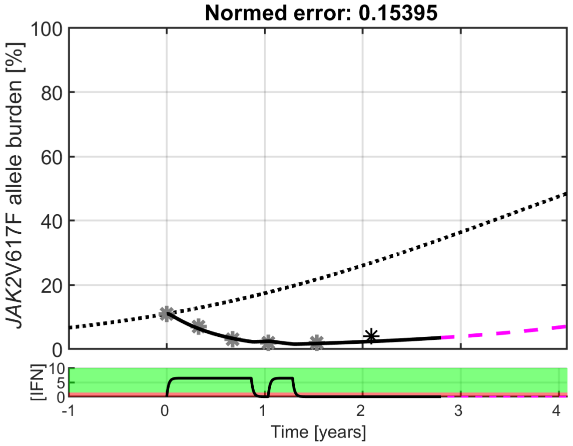

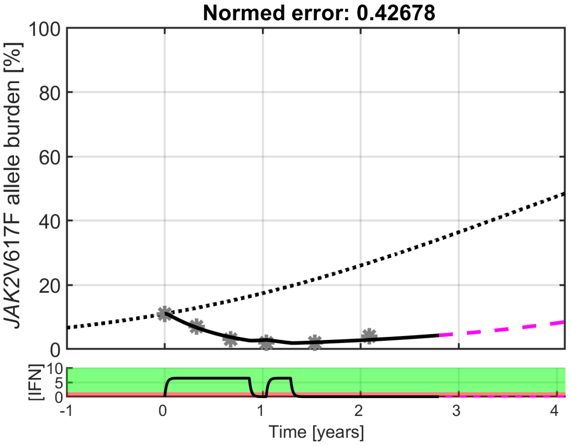

Patient P014

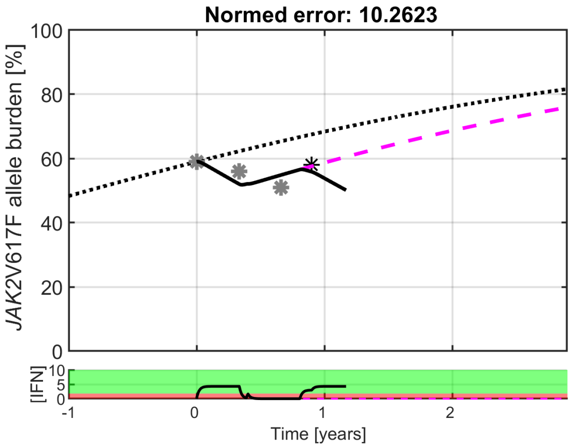

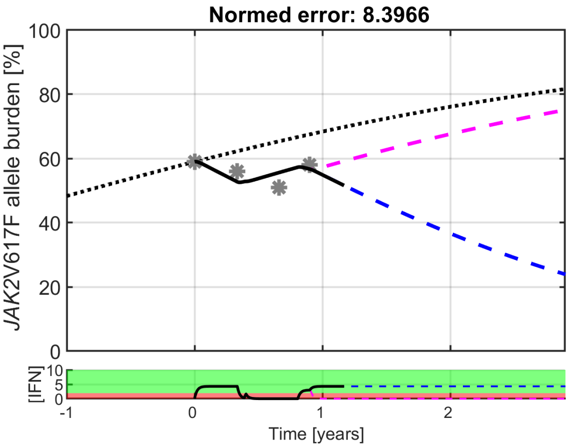

Patient P016

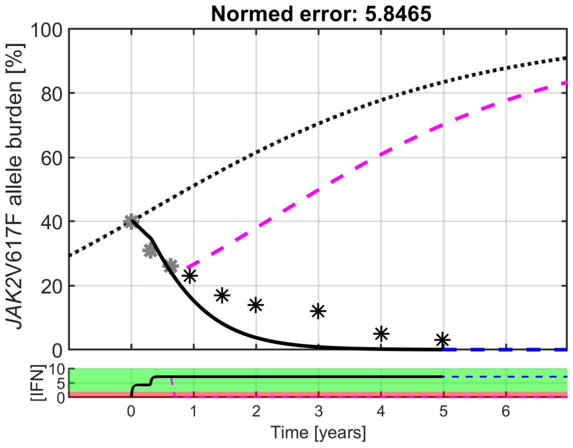

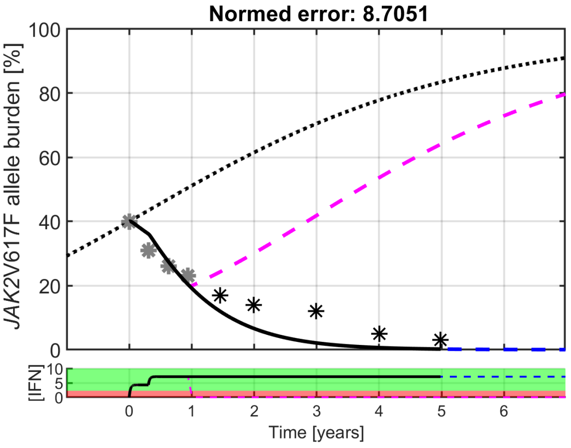

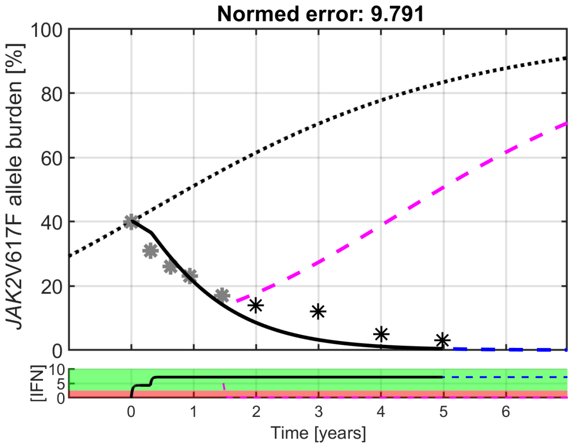

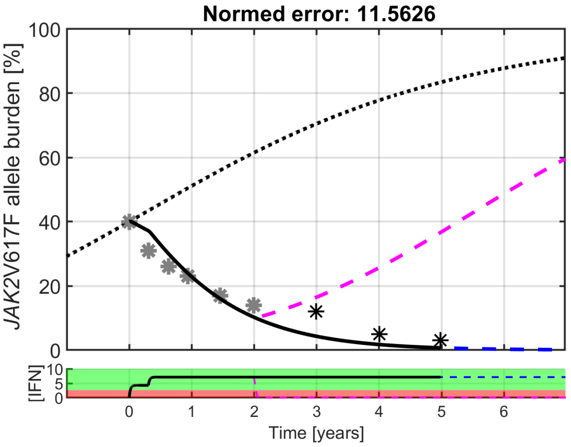

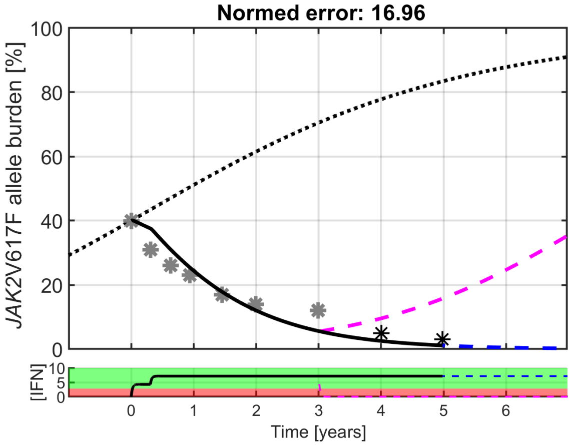

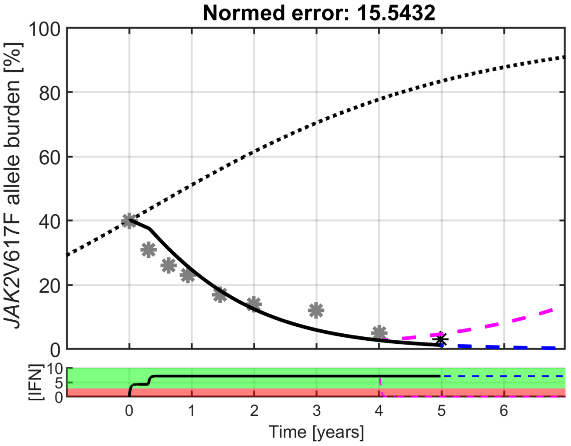

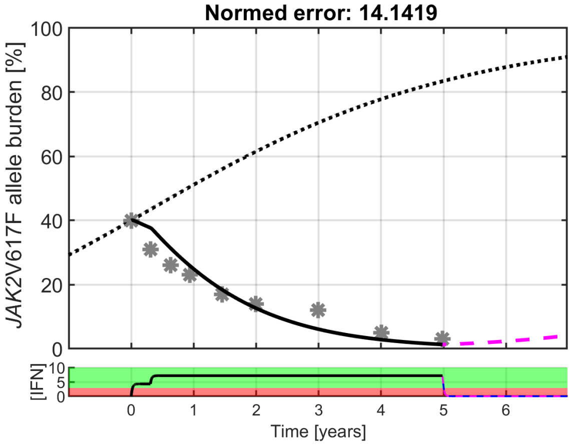

Patient P021

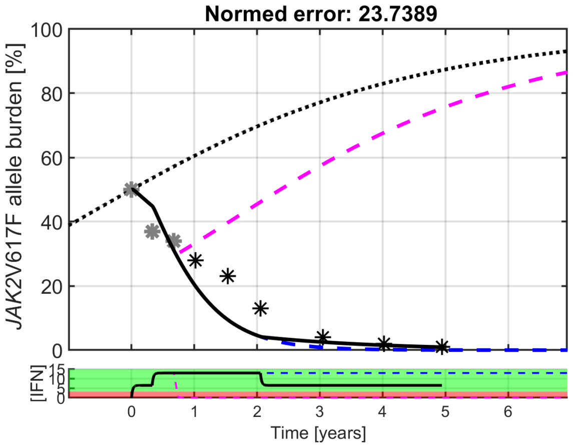

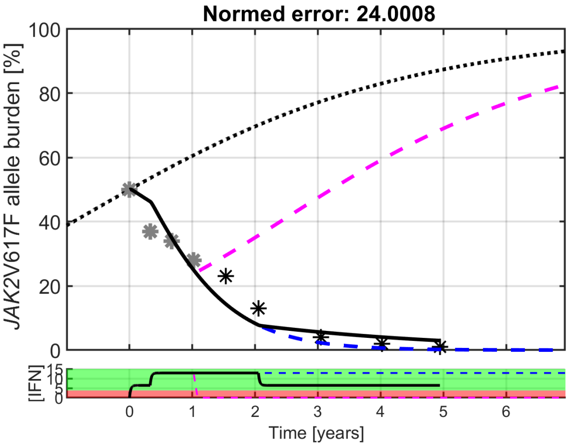

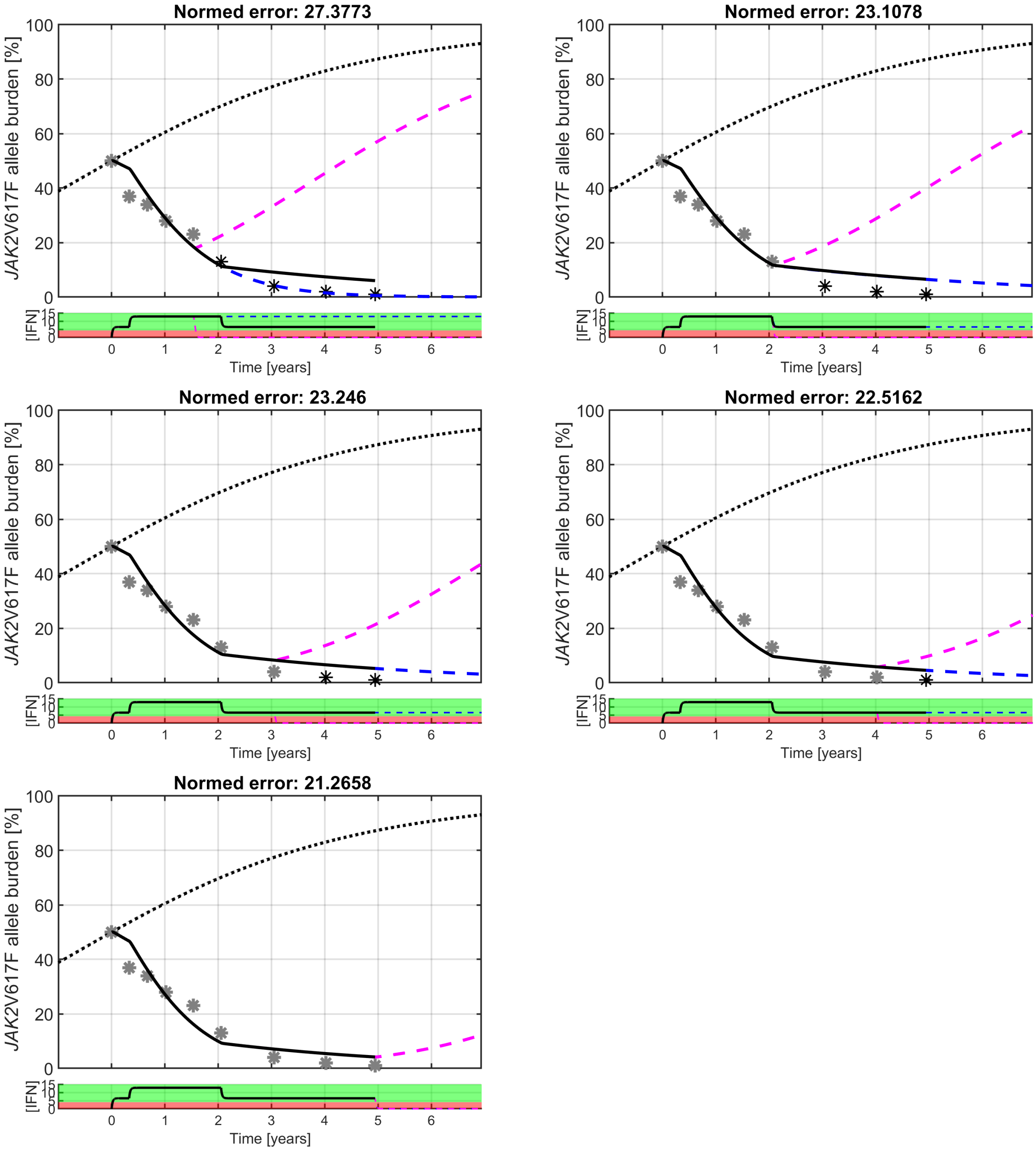

Patient P026

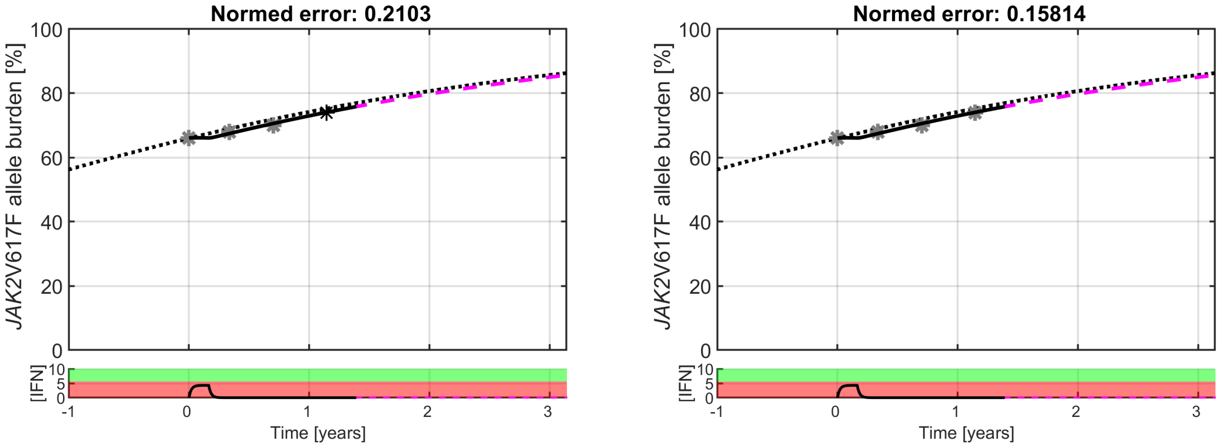

Patient P028

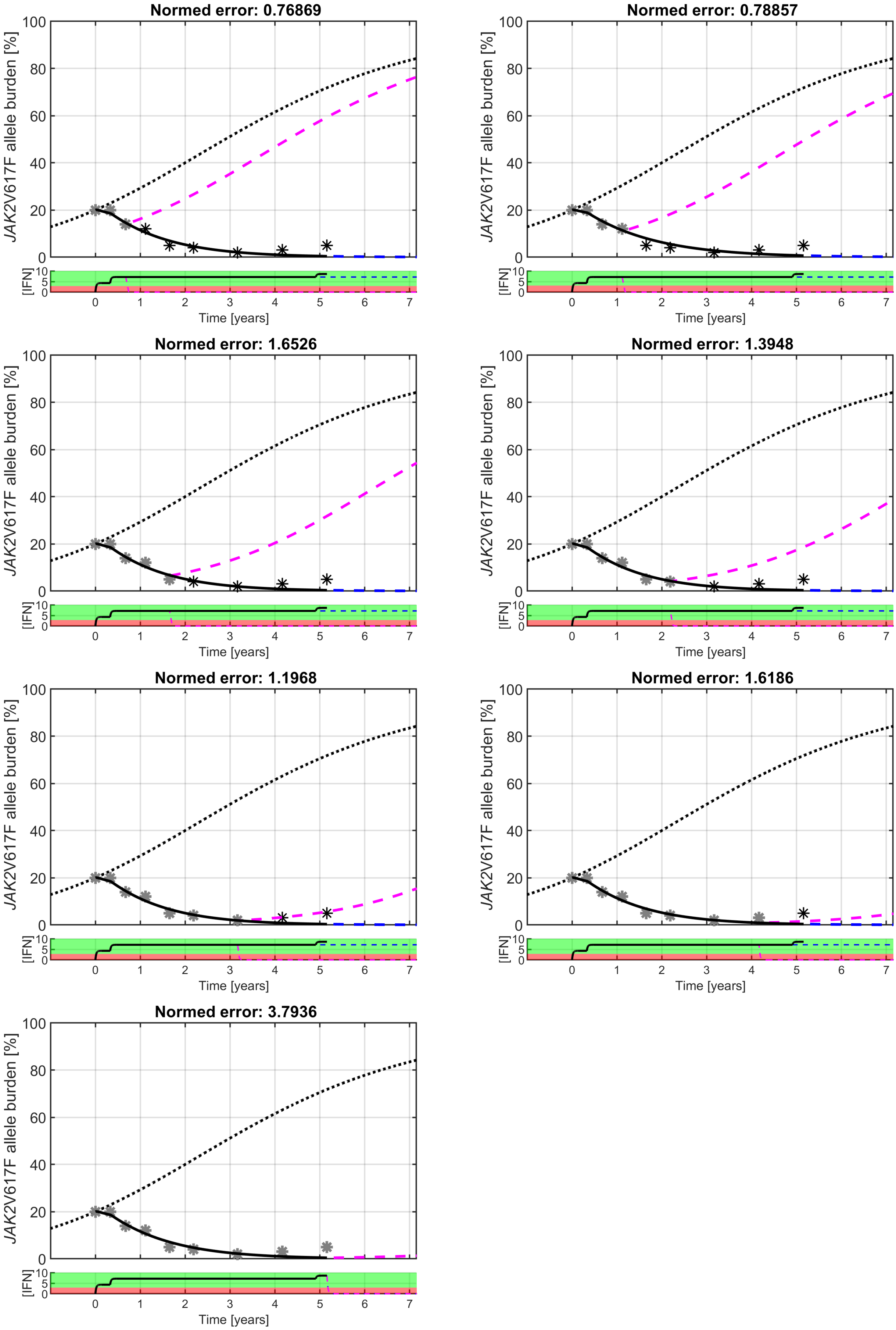

Patient P035

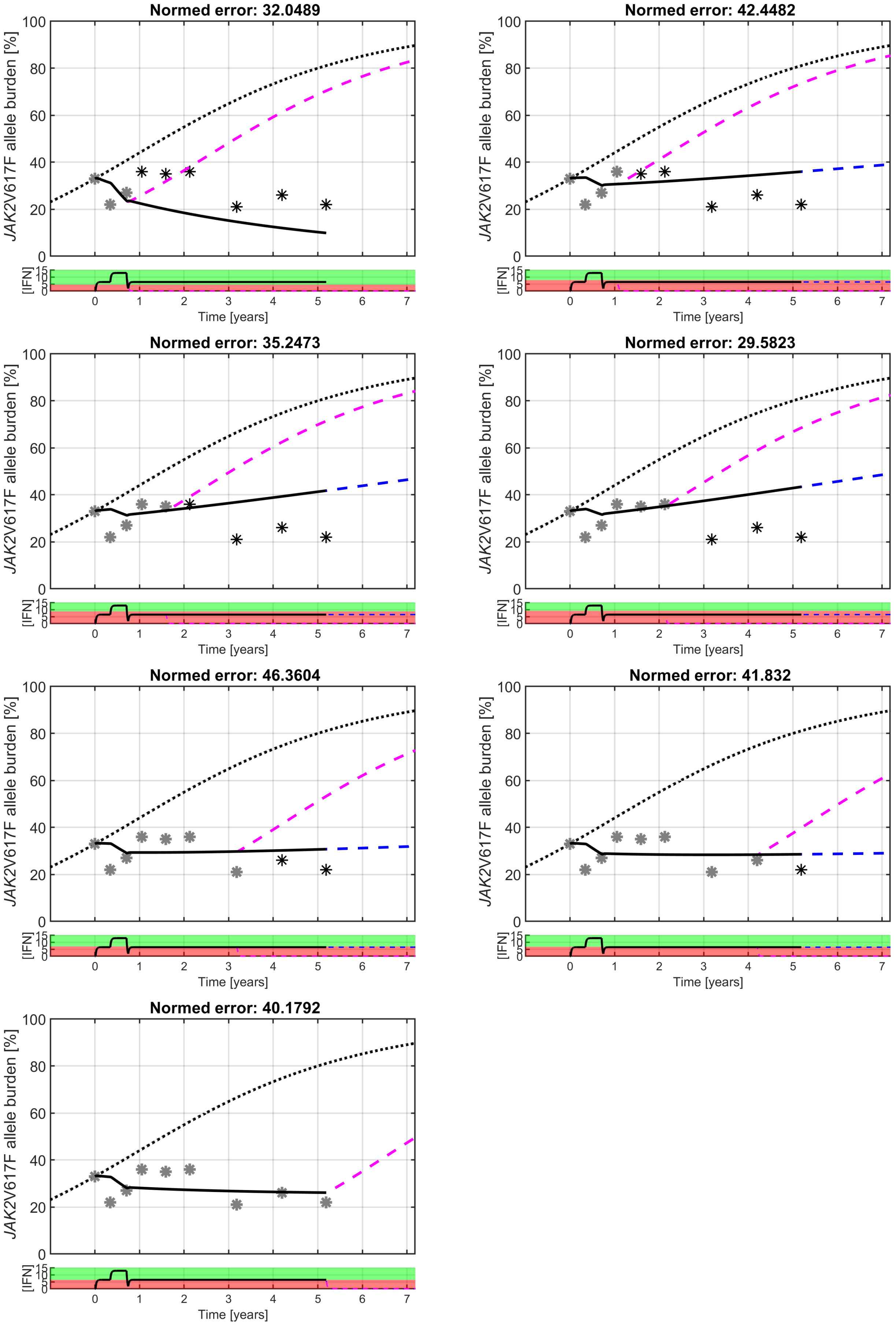

Patient P039

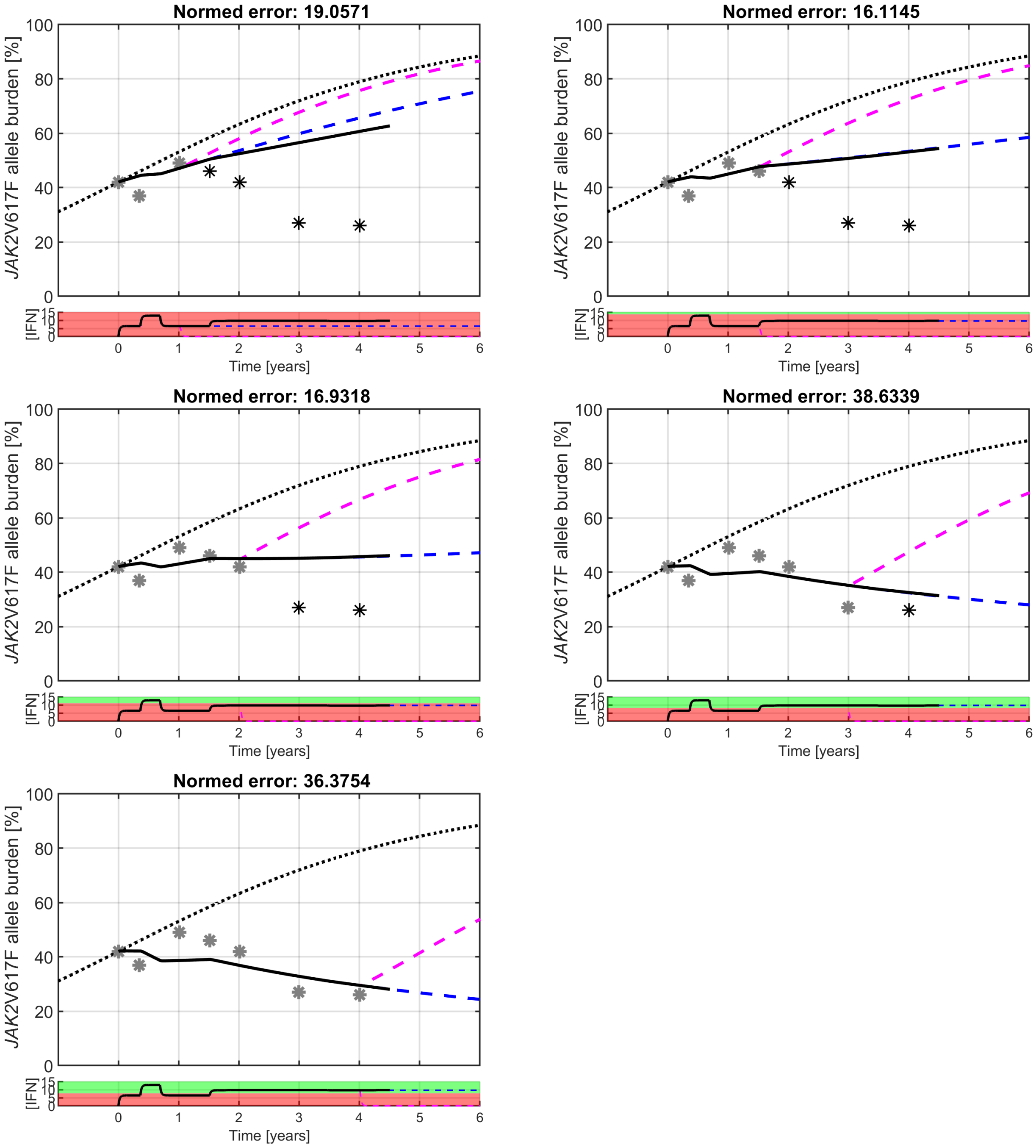

Patient P045

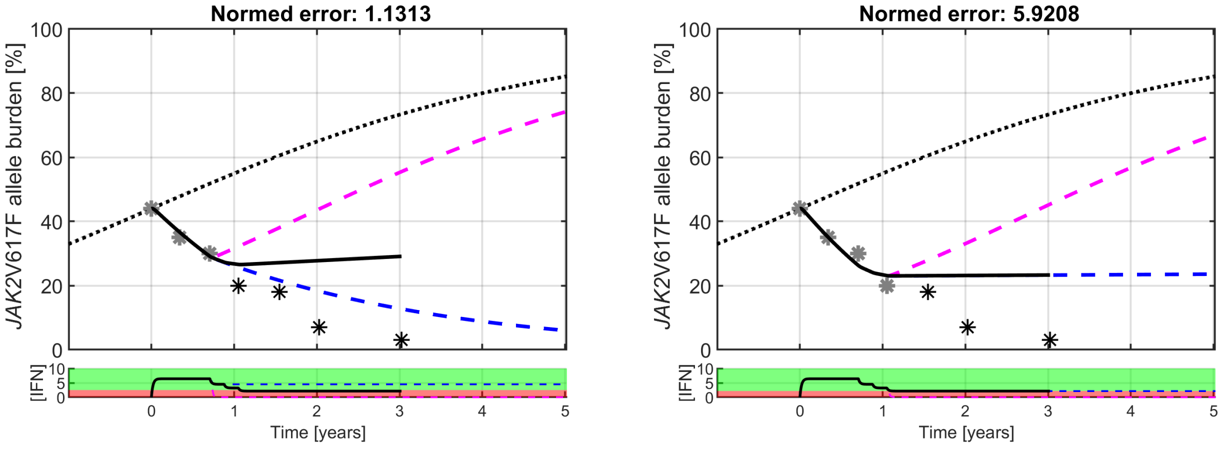

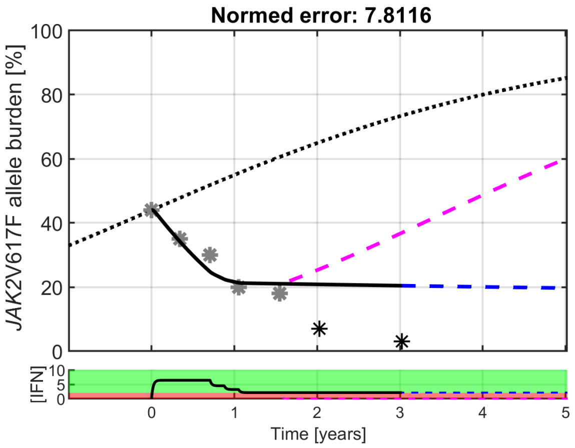

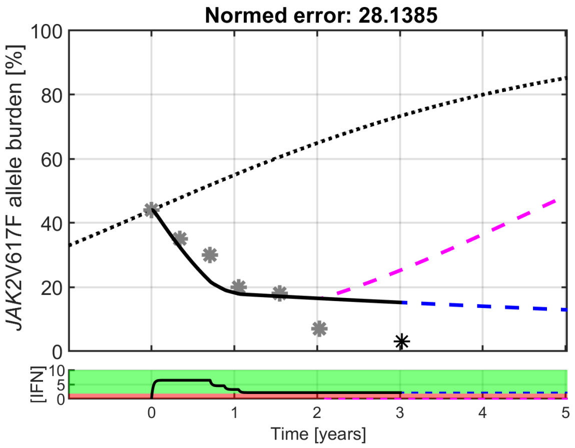

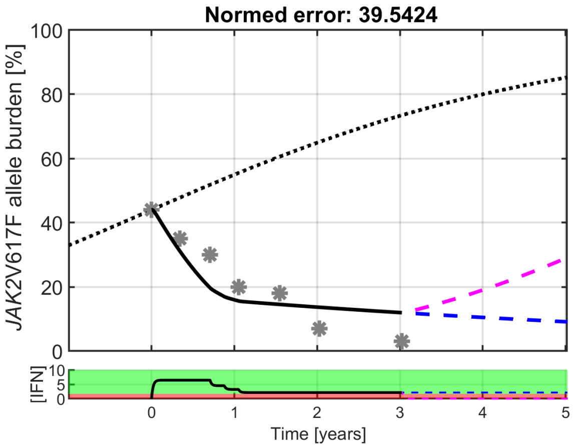

Patient P046

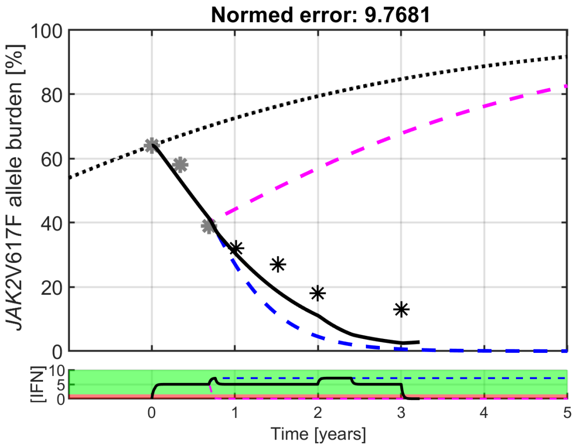

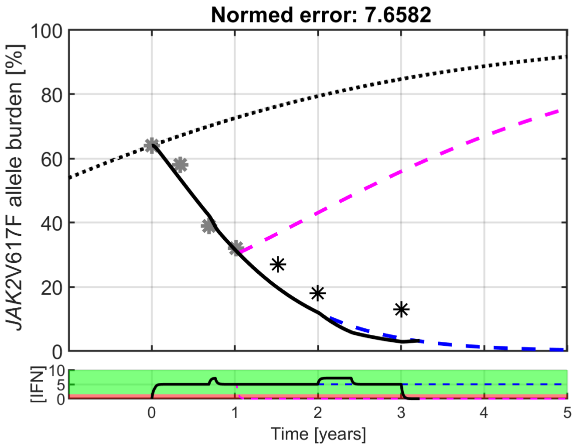

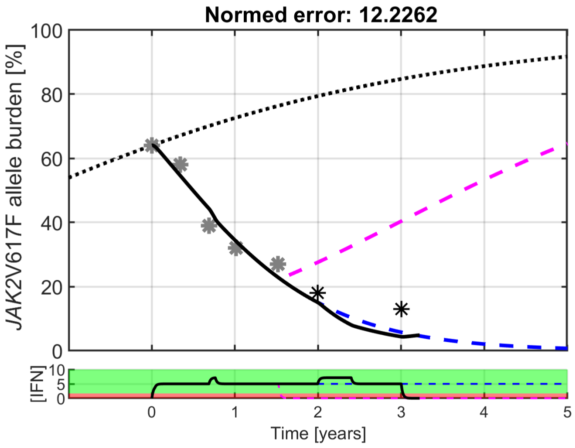

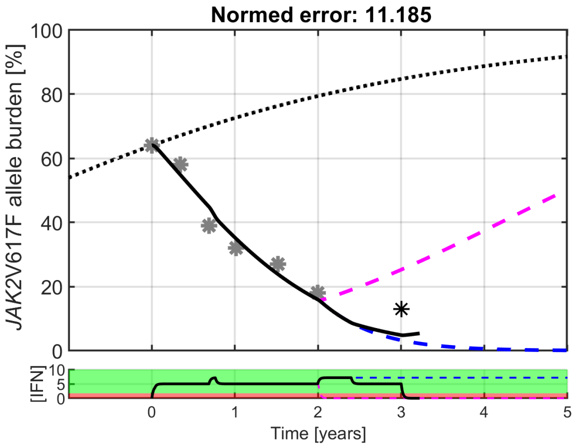

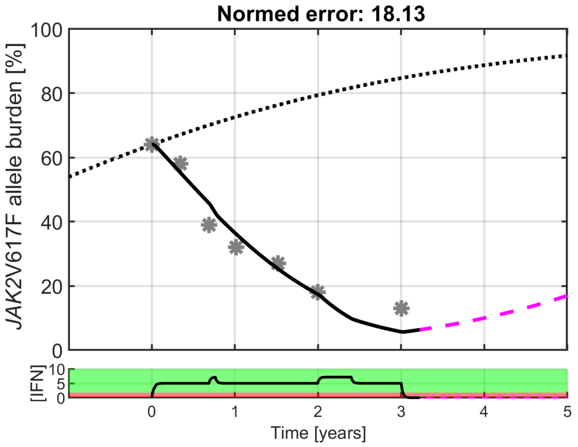

Patient P046

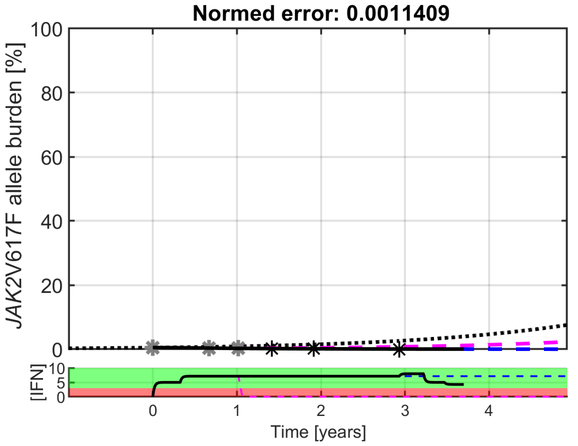

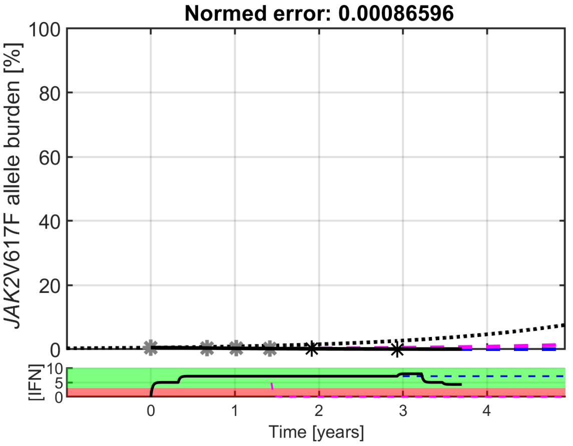

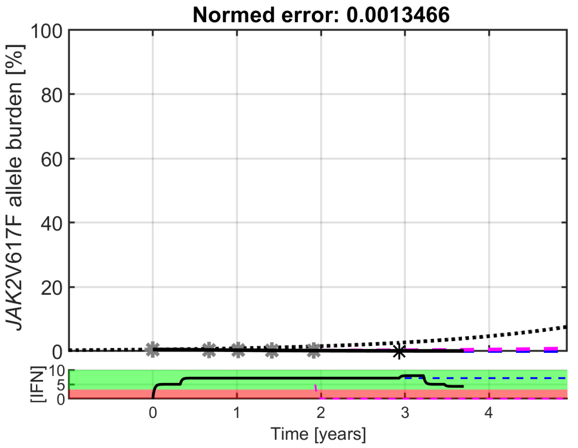

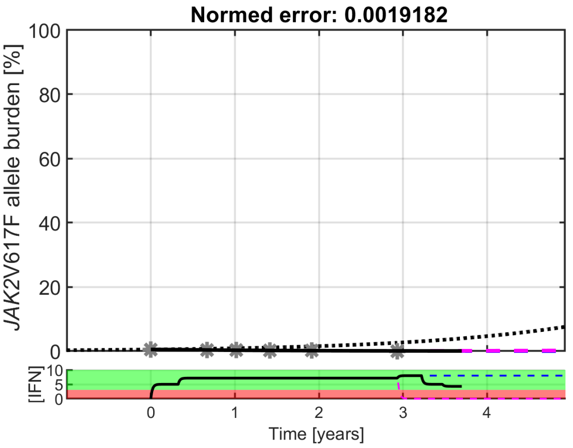

Patient P062

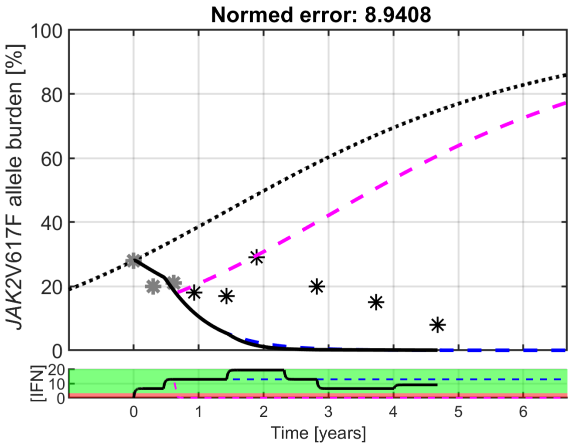

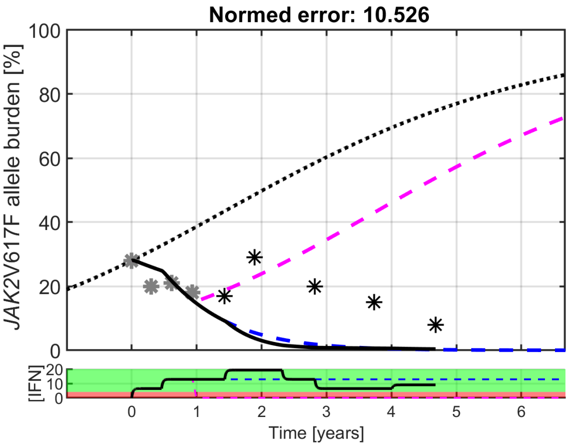

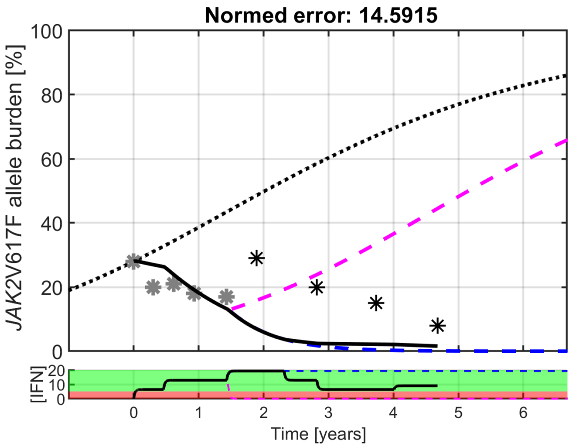

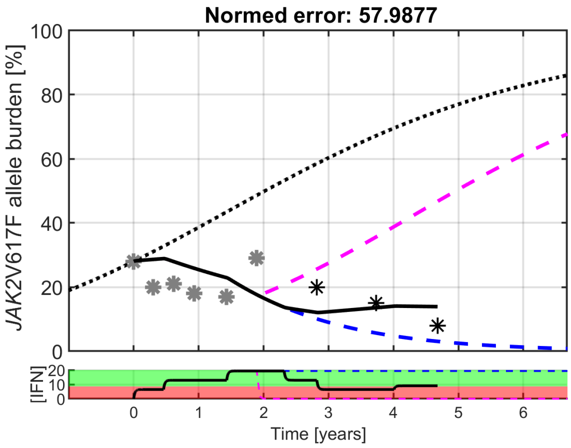

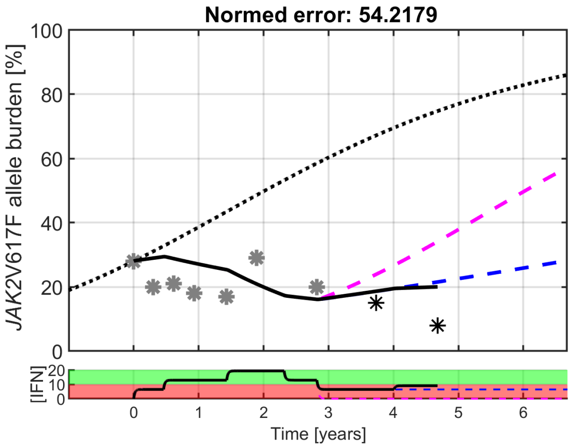

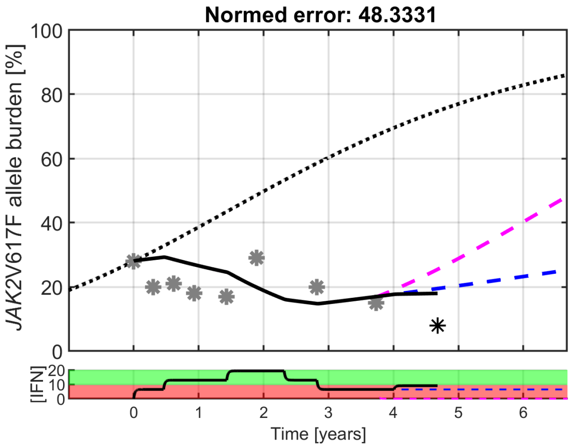

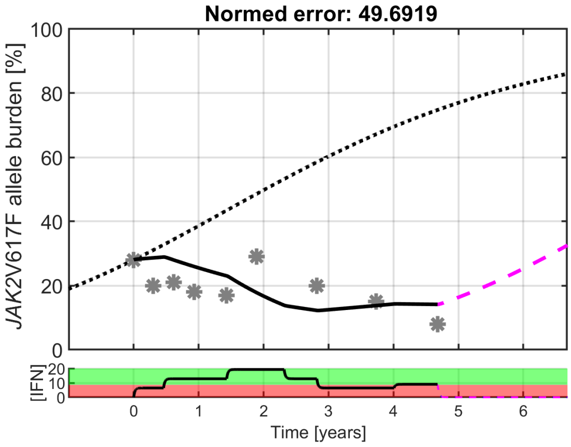

Patient P067

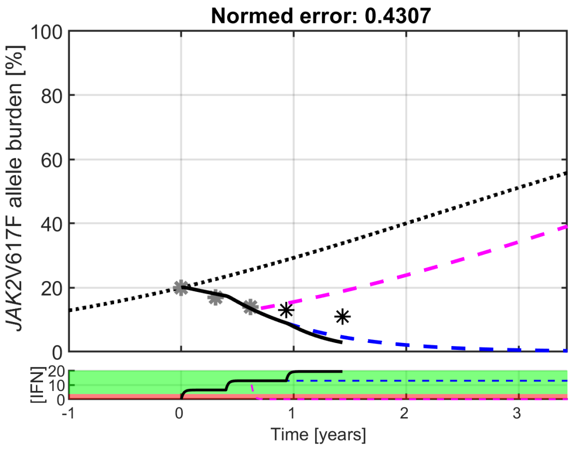

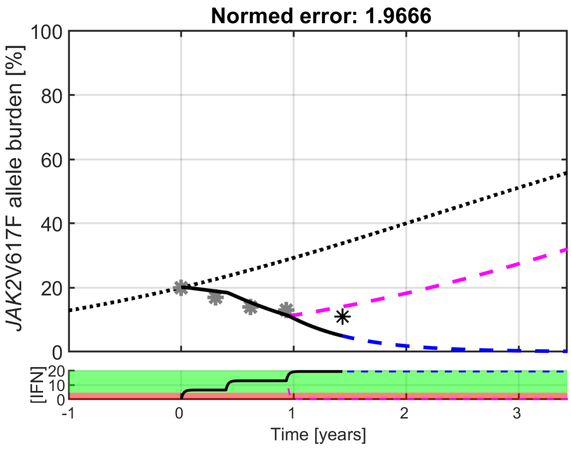

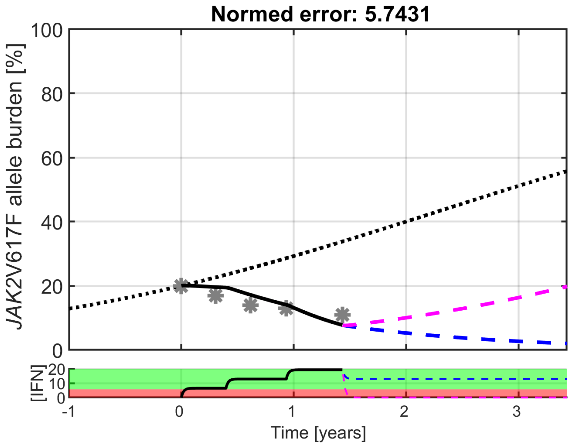

Patient P070

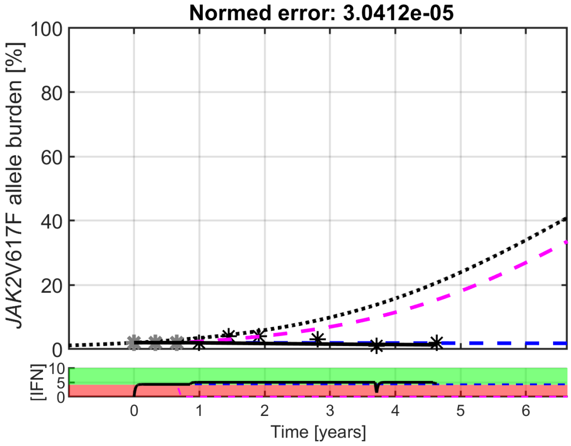

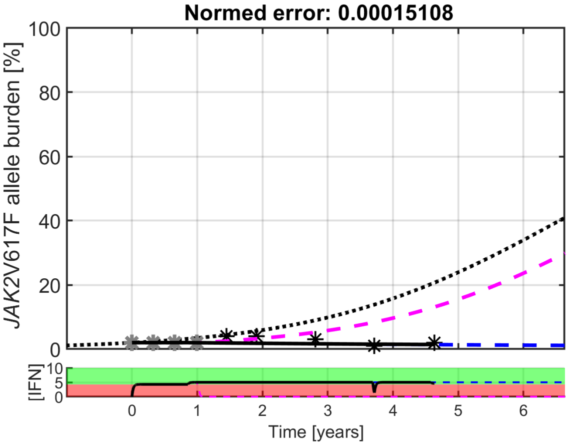

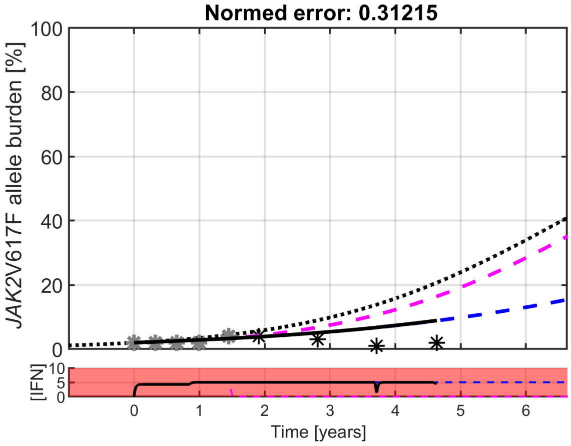

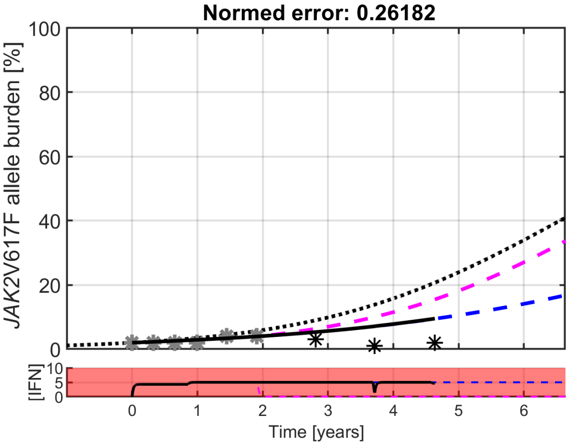

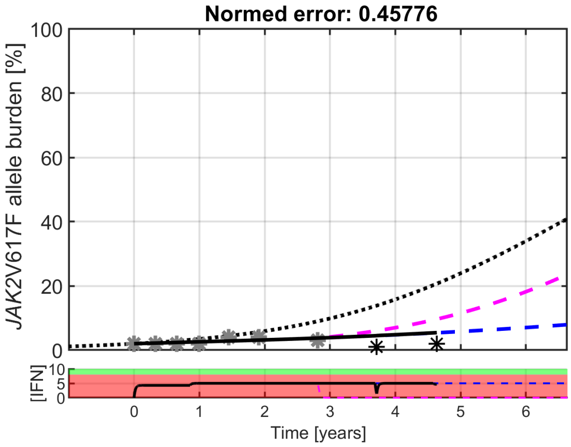

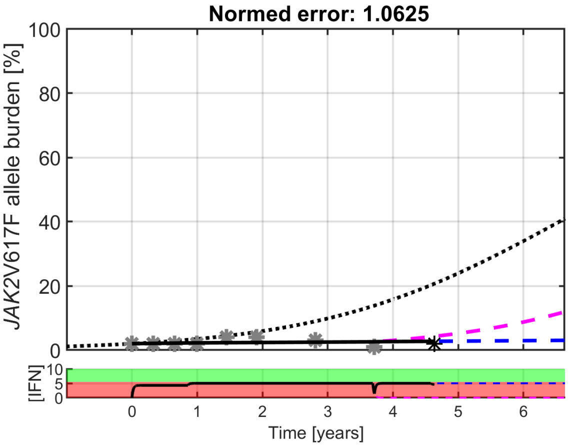

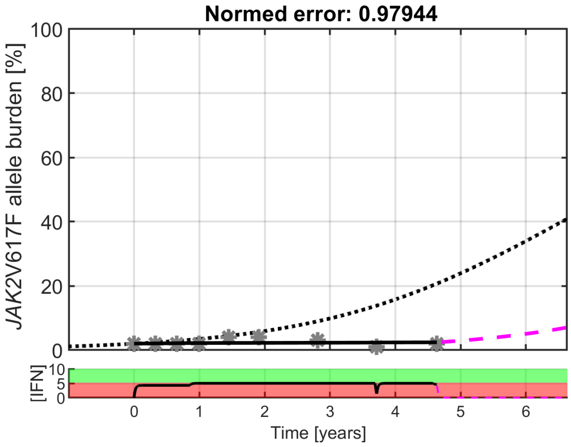

Patient P075

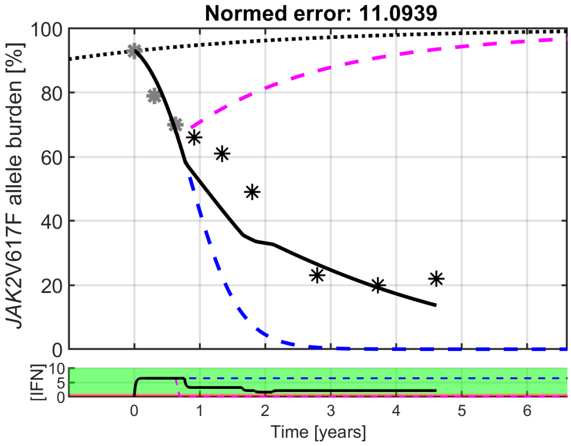

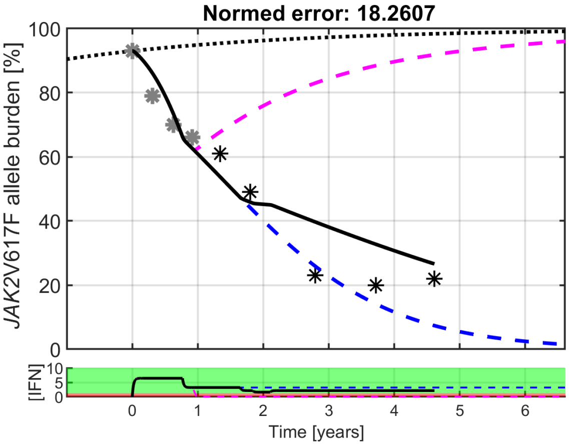

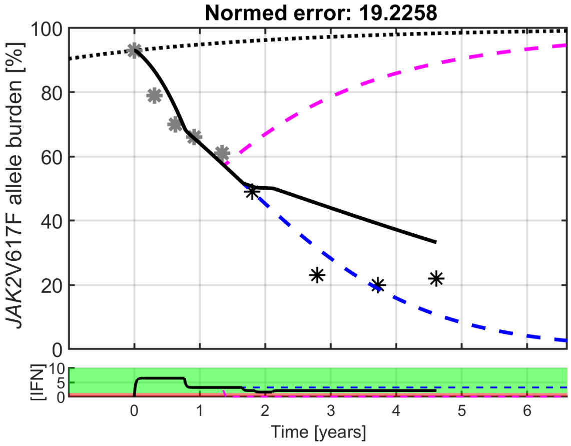

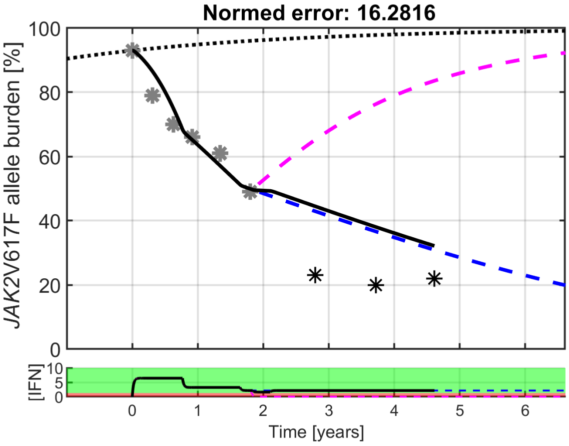

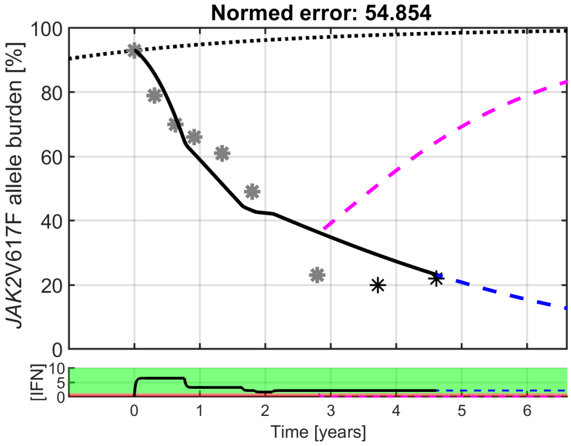

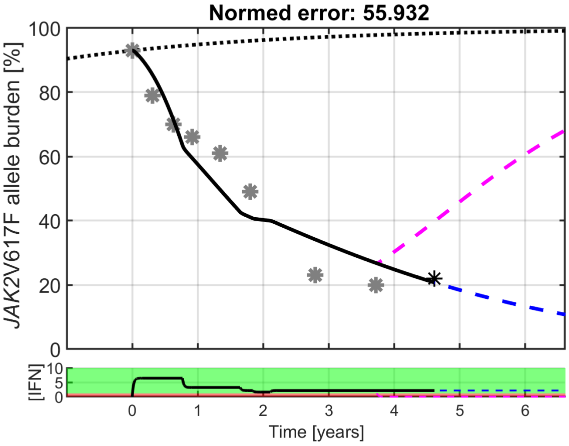

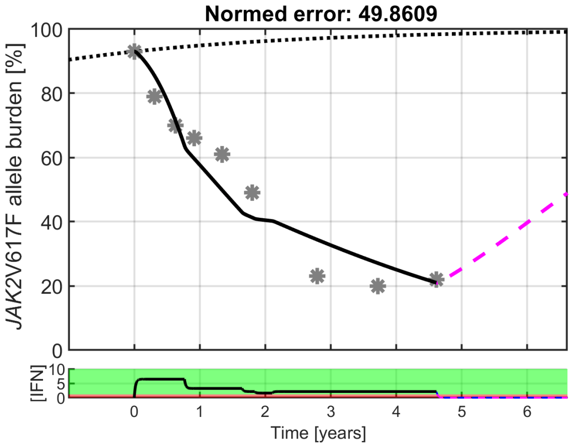

Patient P078

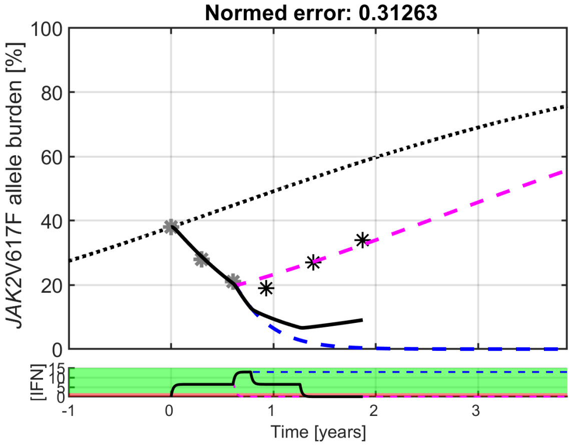

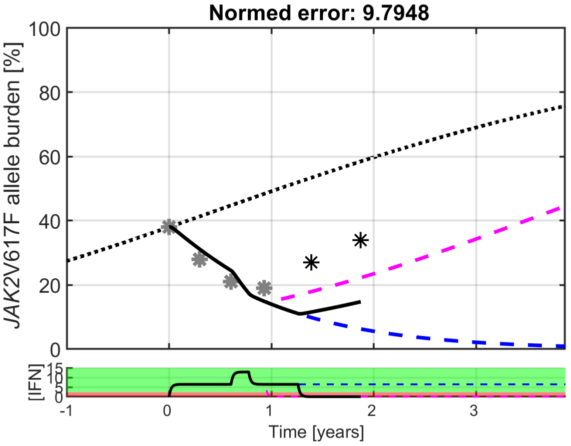

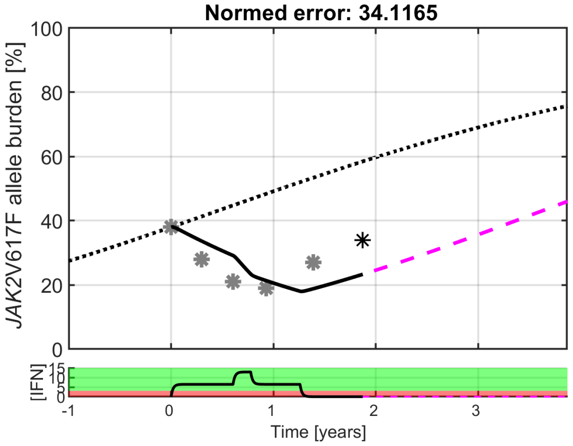

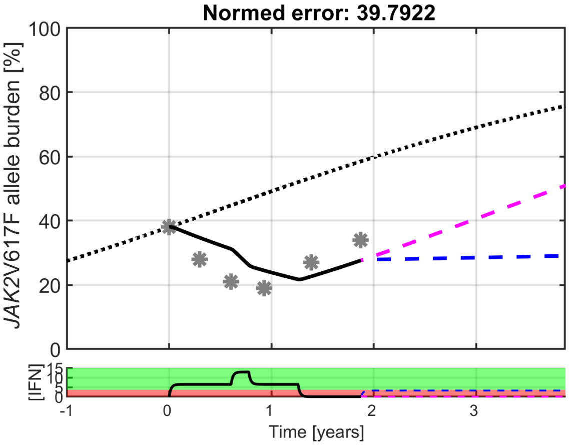

Patient P079

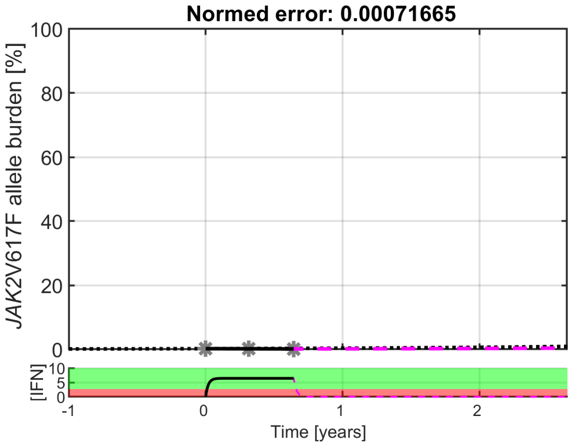

Patient P082

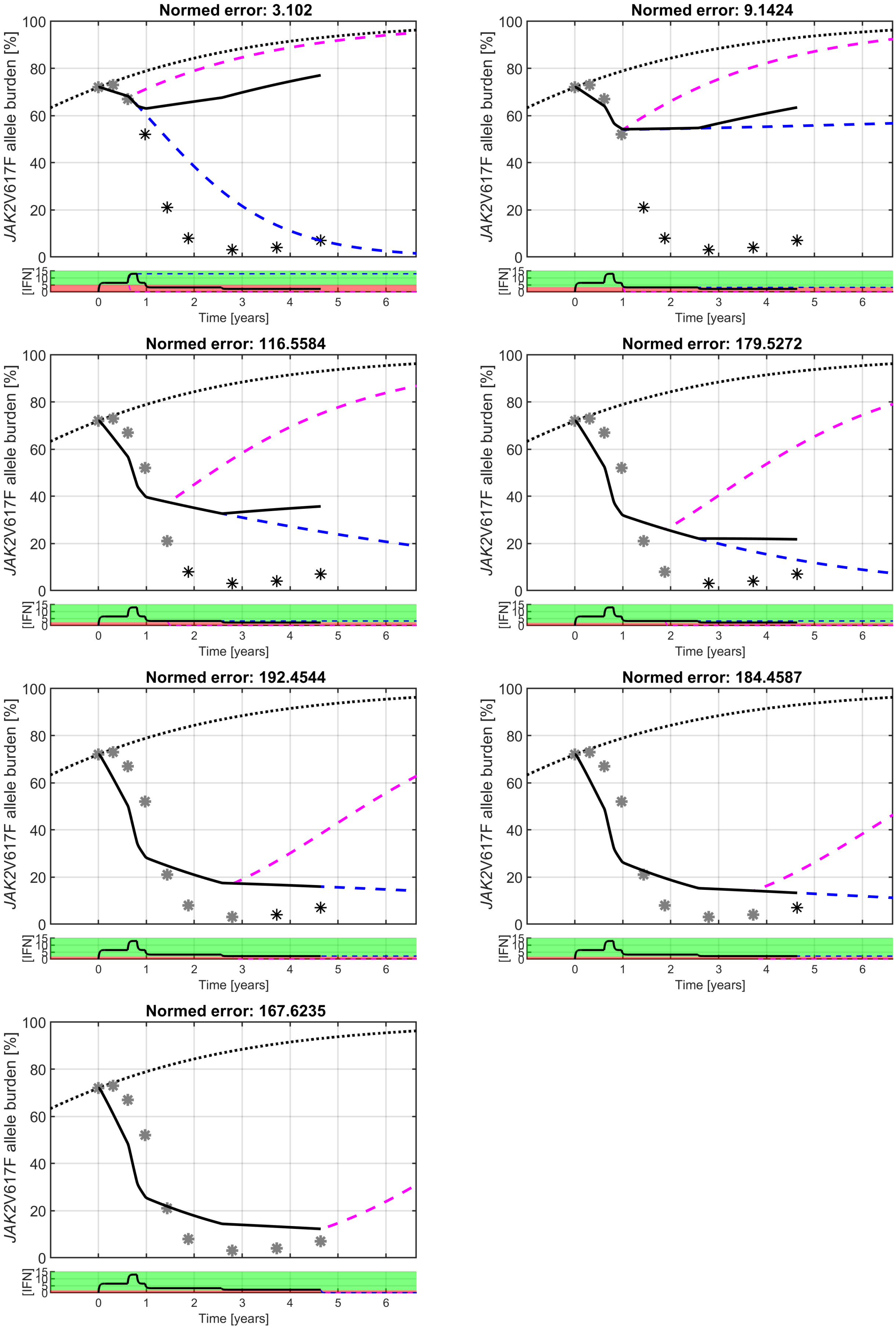

Patient P084

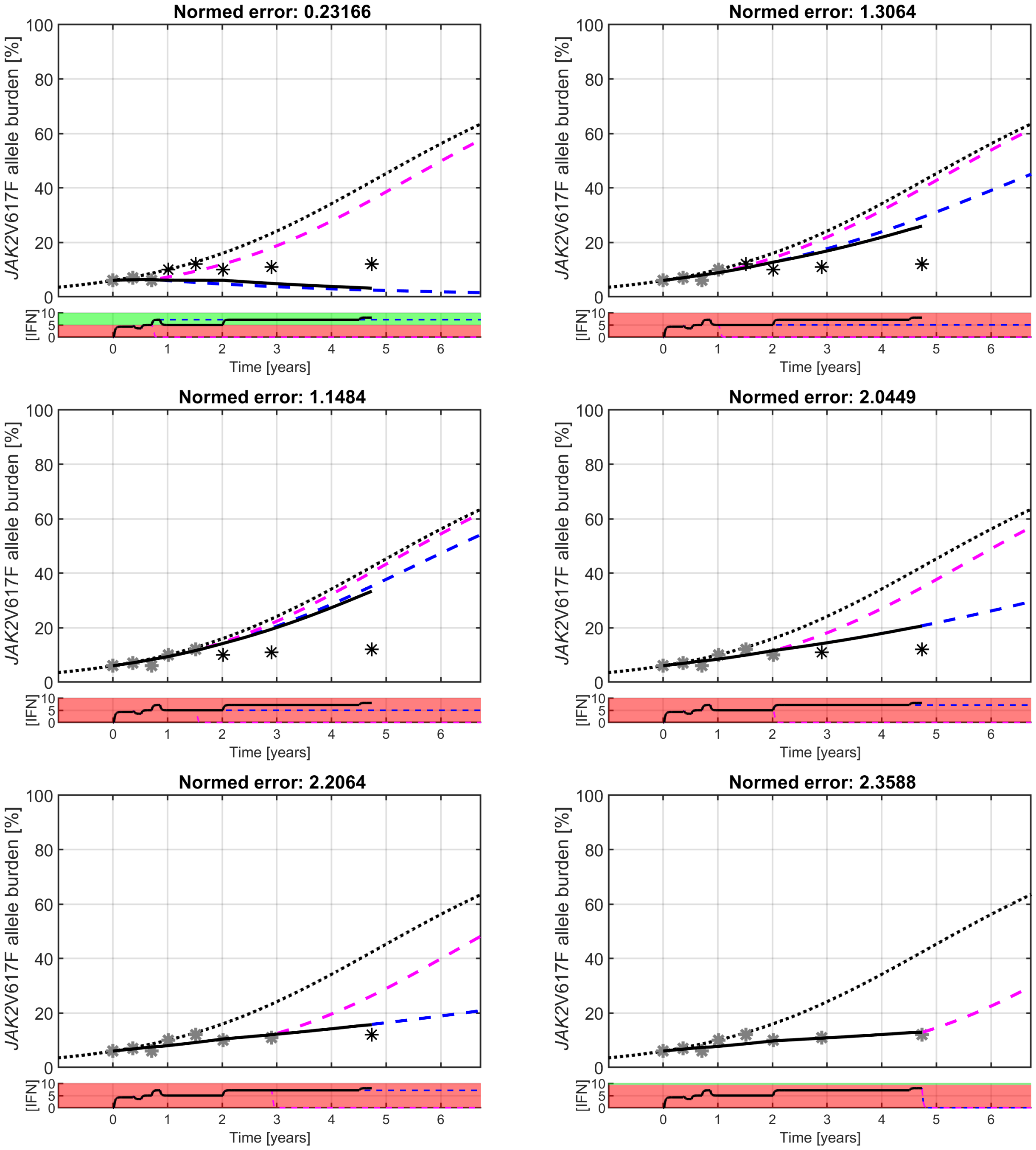

Patient P086

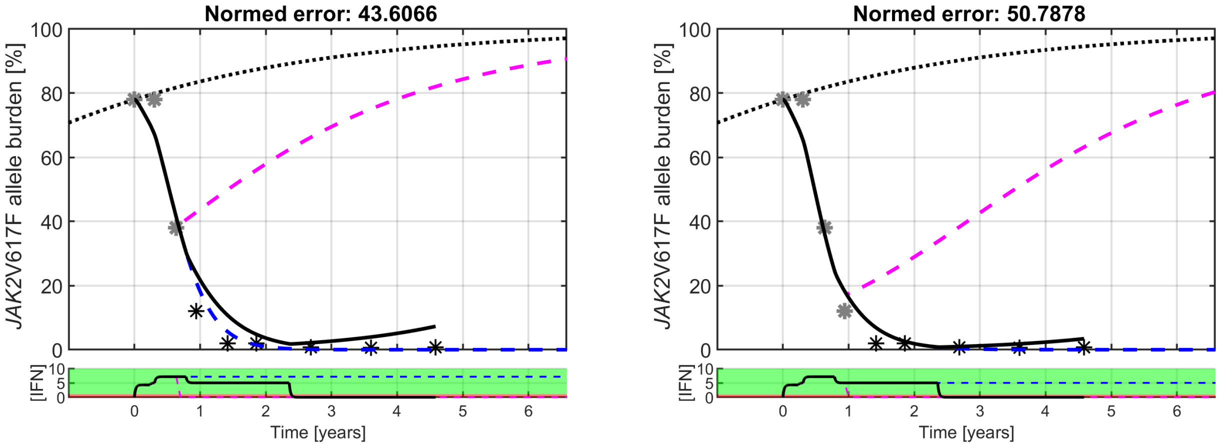

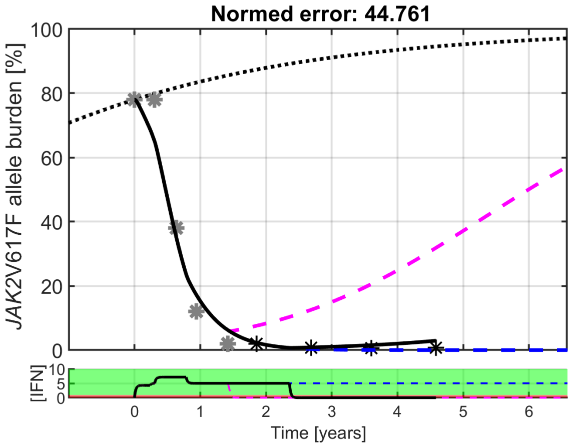

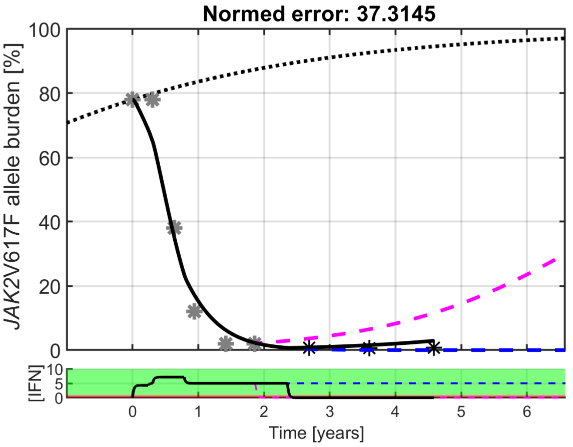

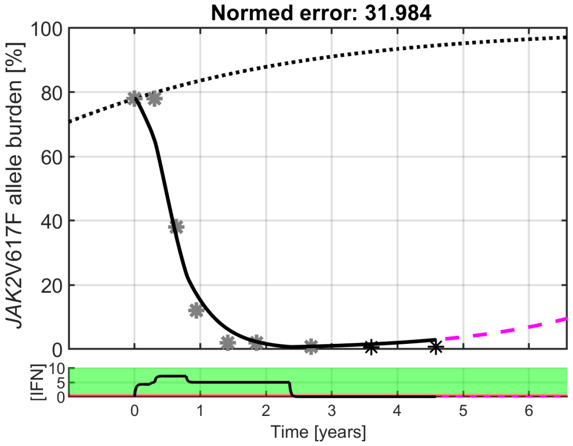

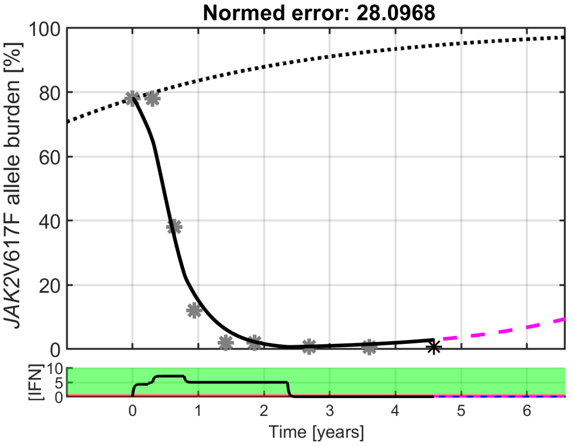

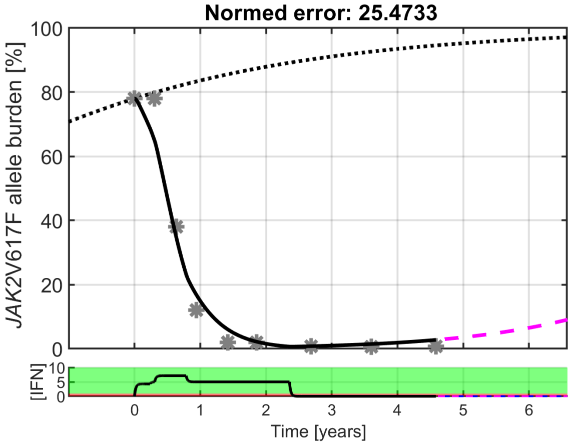

Patient P087

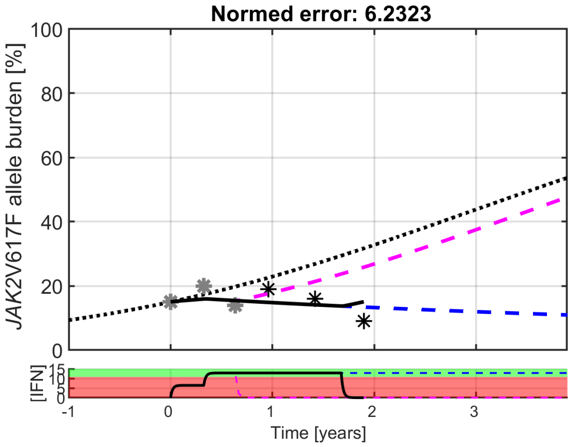

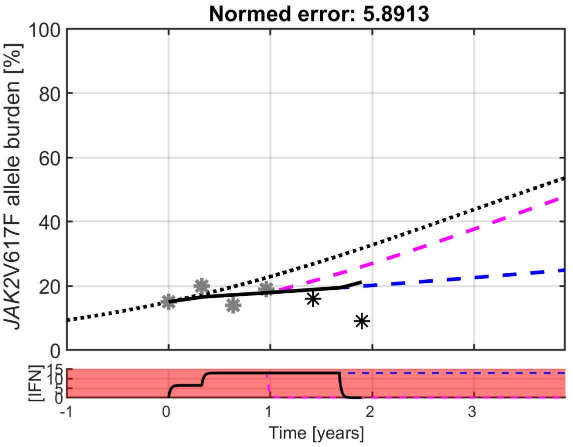

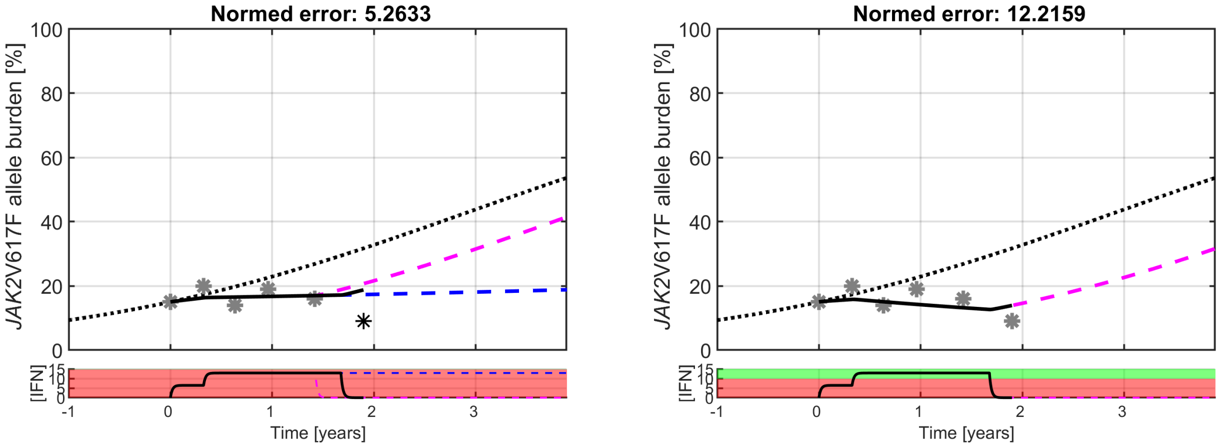

Patient P089

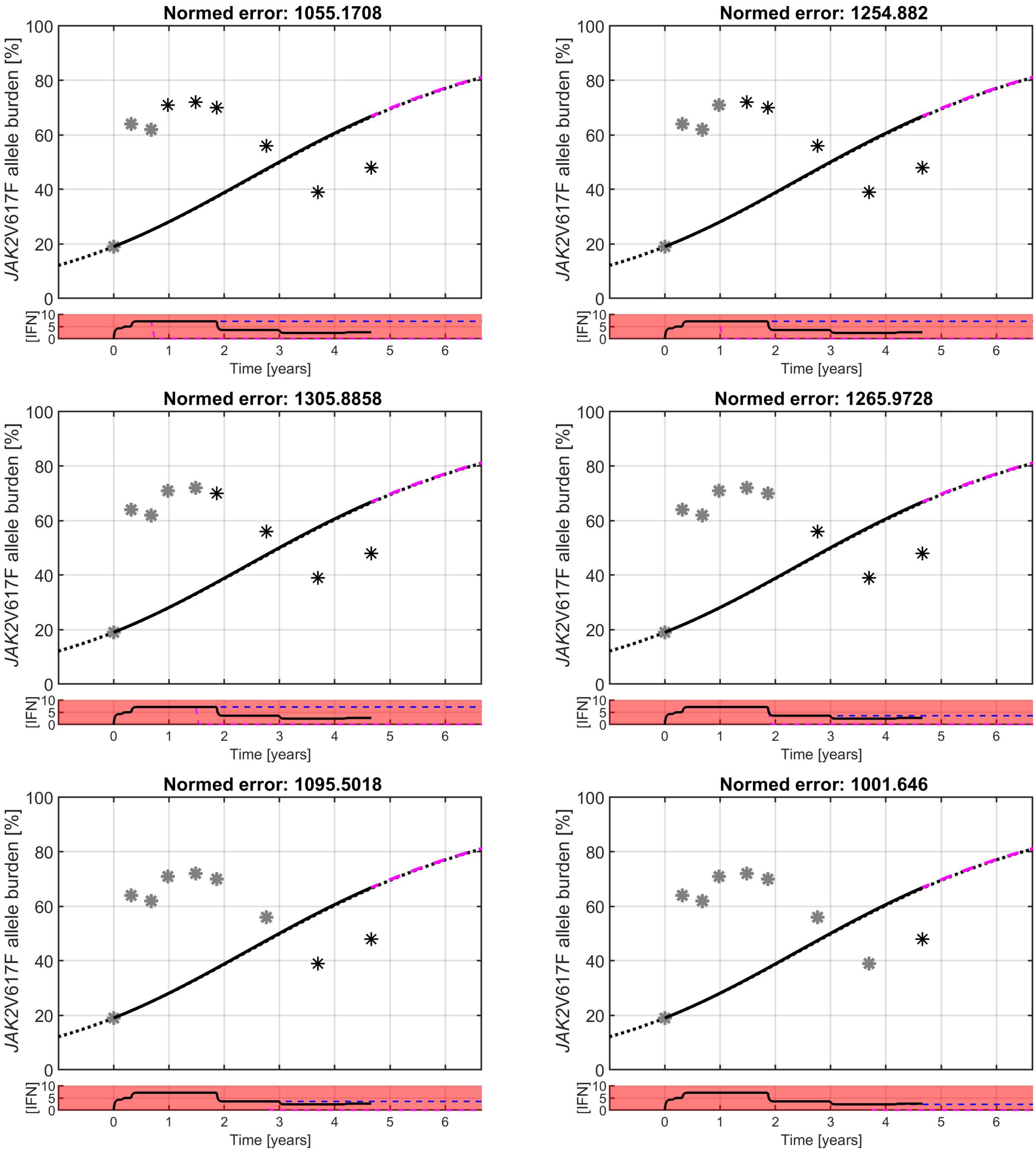

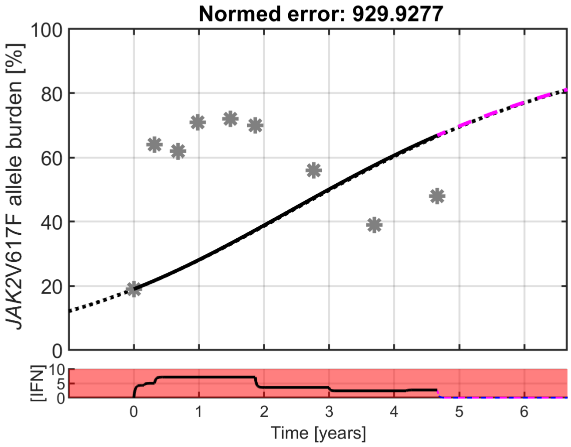

Patient P105

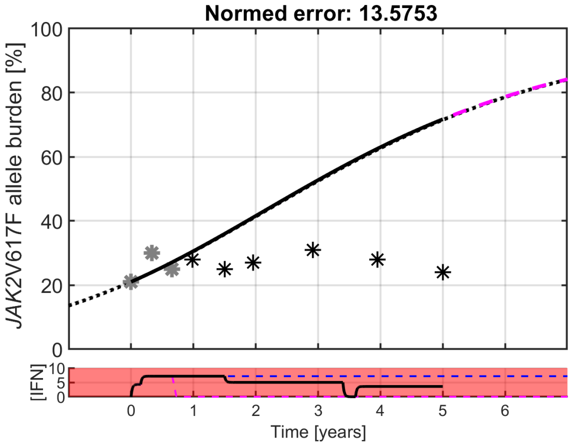

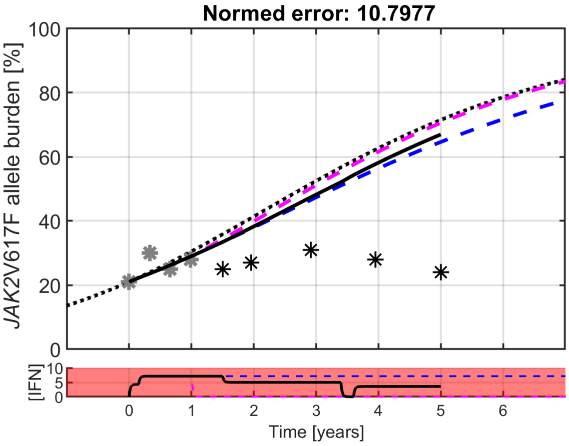

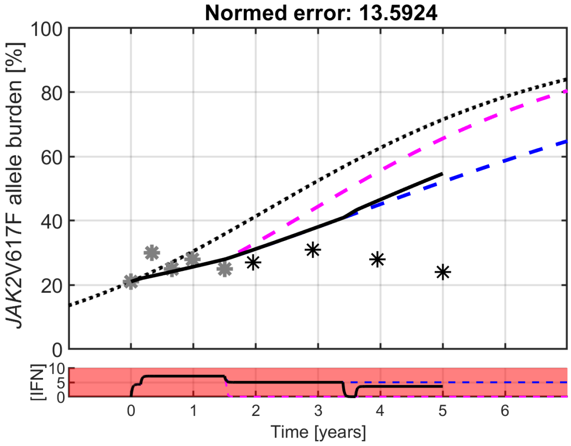

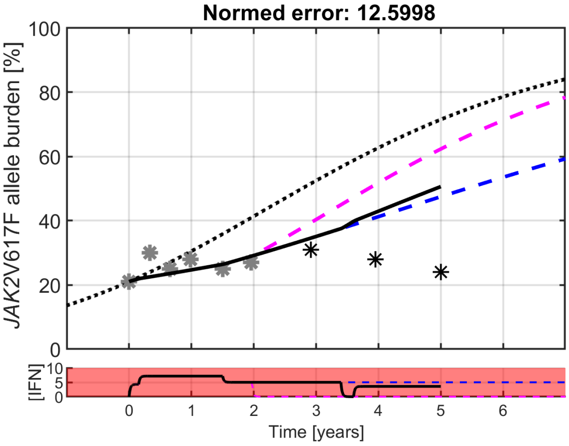

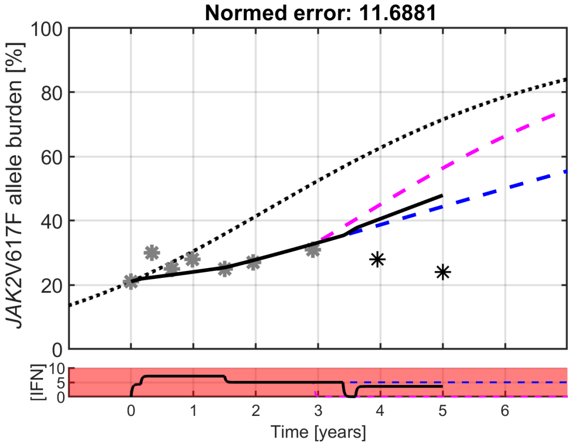

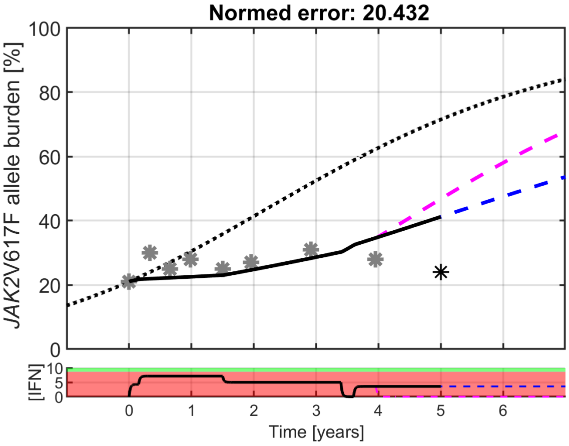

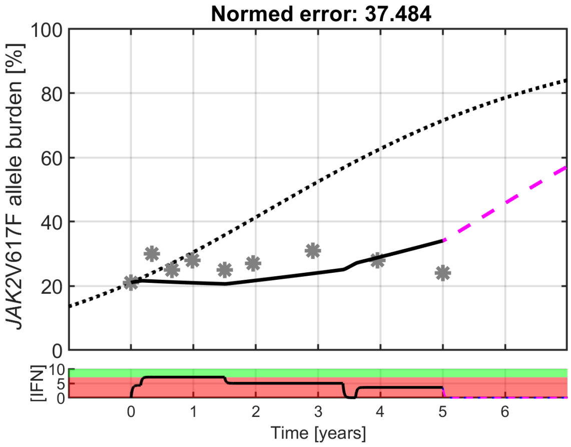

Patient P106

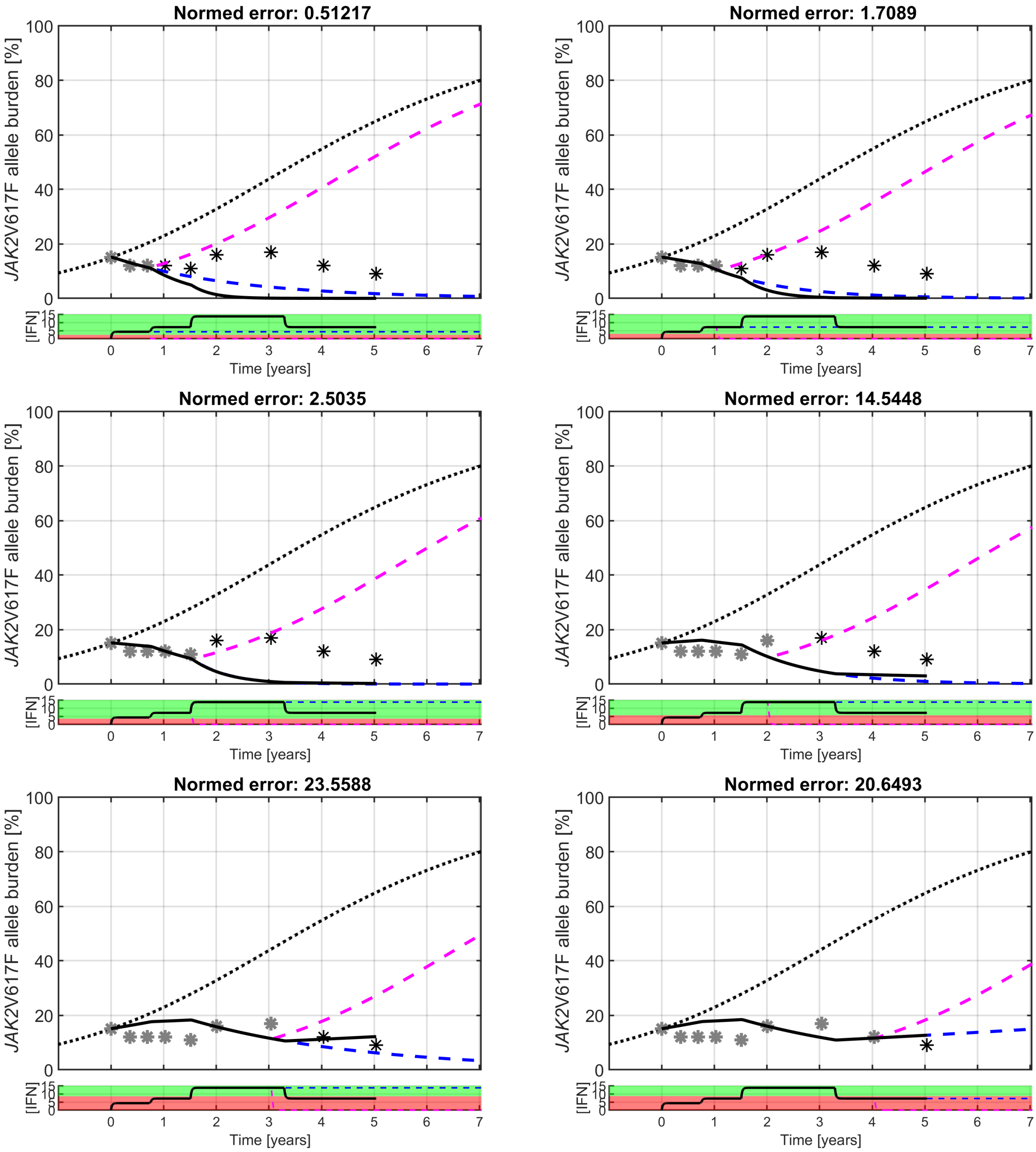

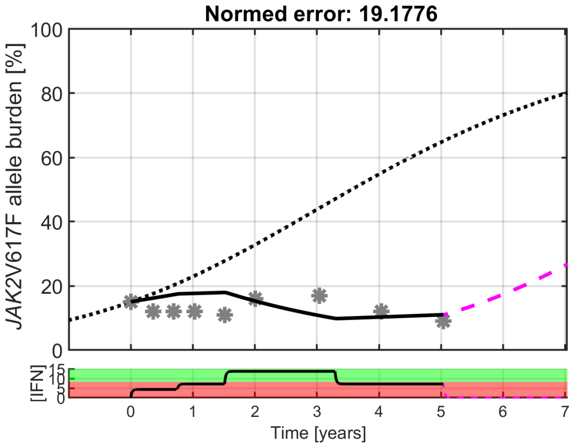

Patient P111

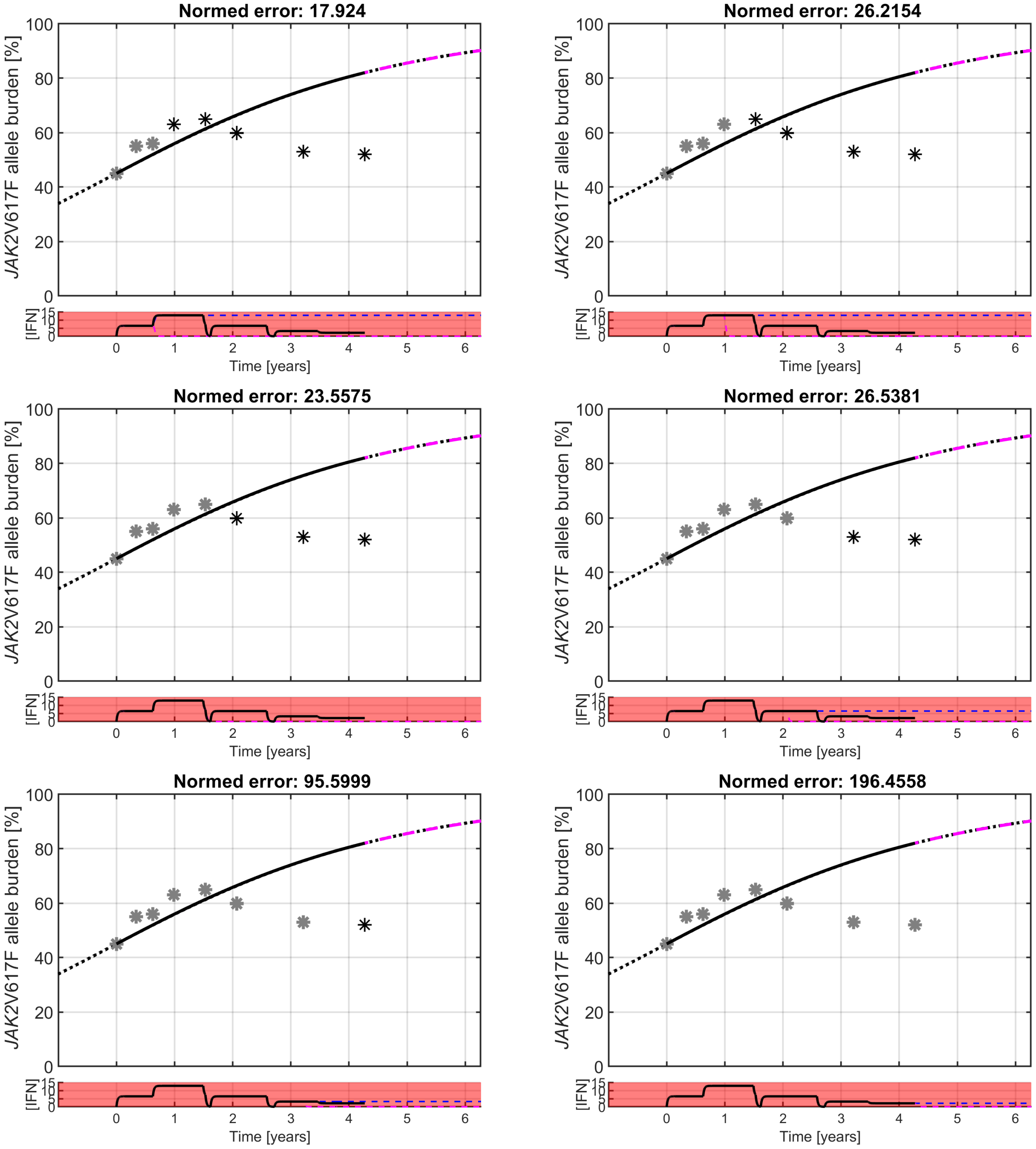

Patient P113

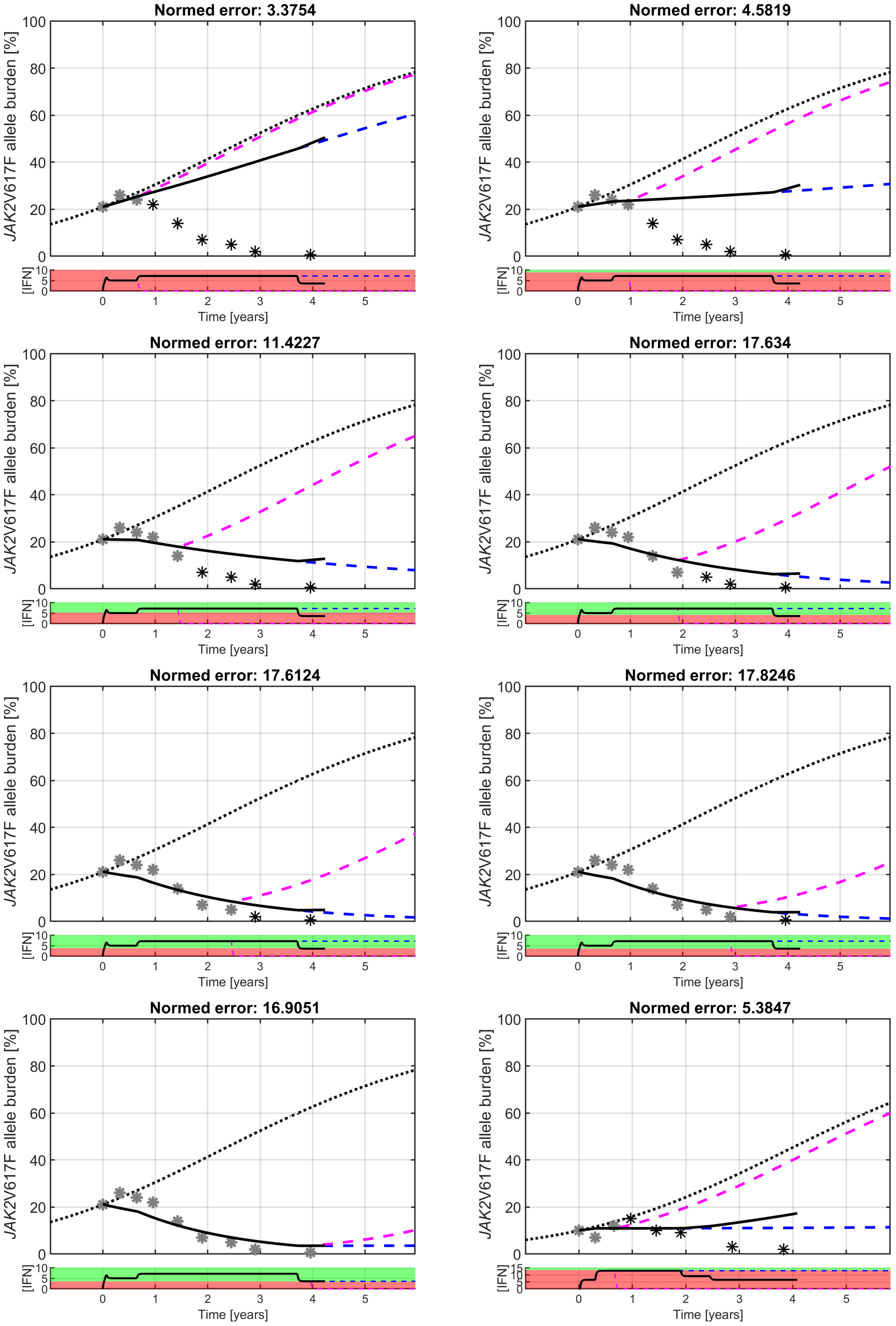

Patient P114

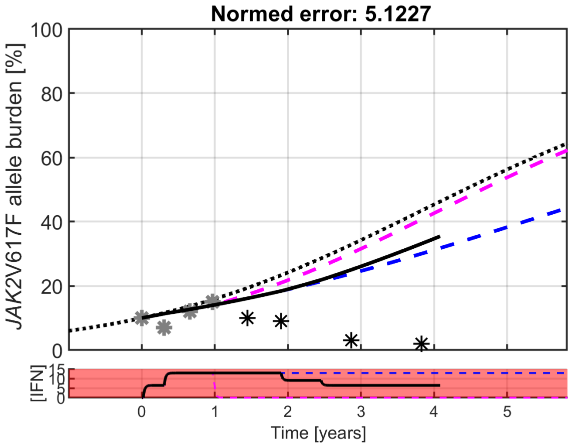

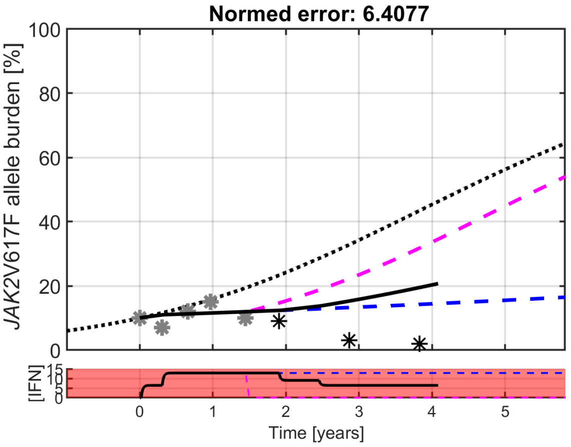

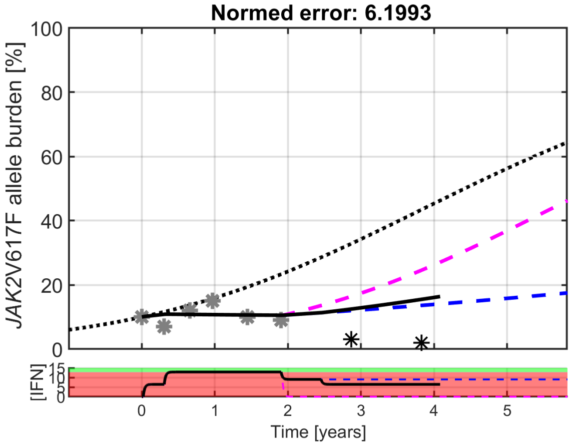

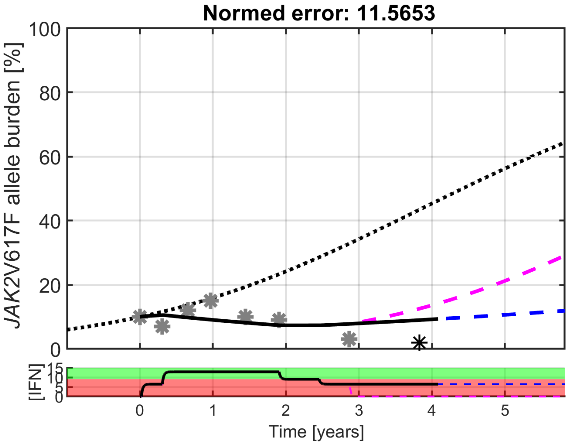

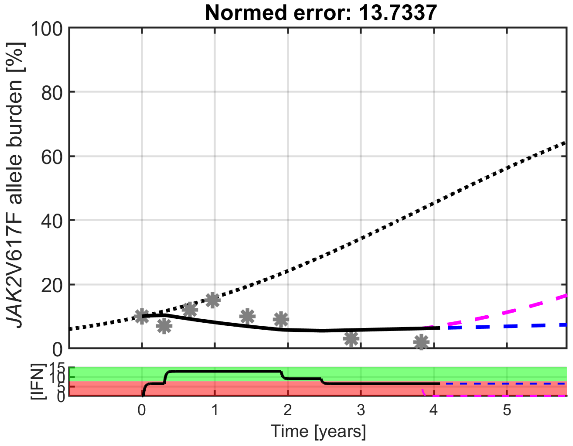

Patient P117

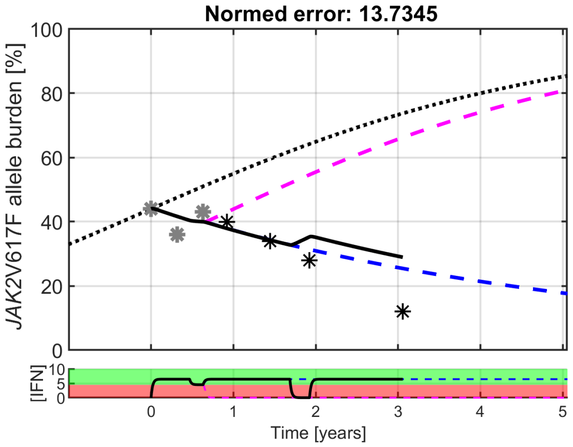

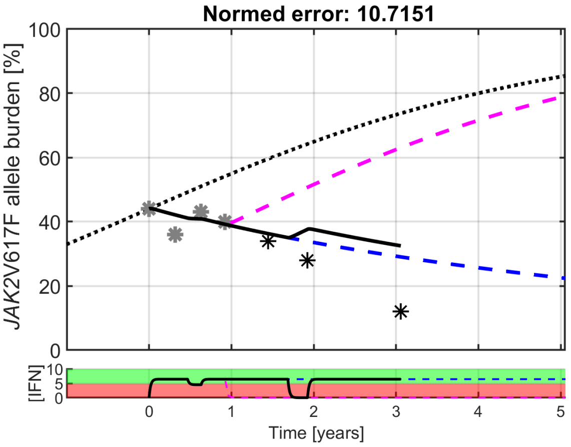

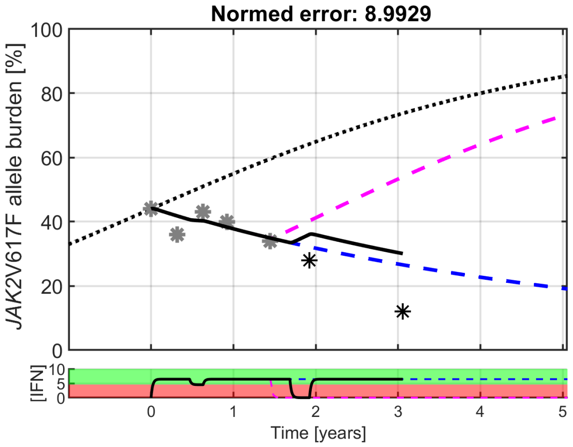

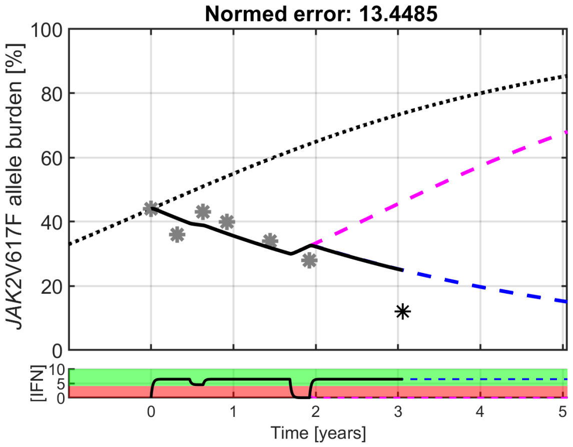

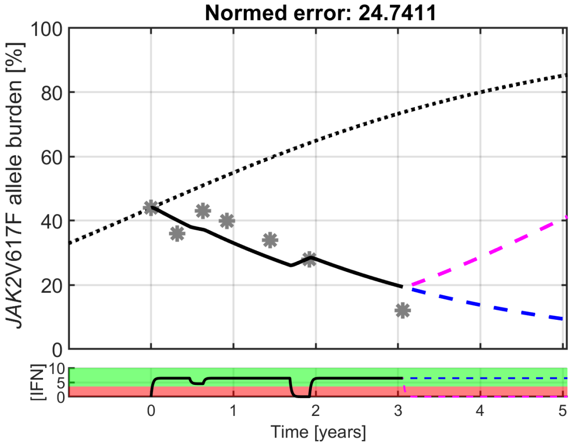

Patient P122

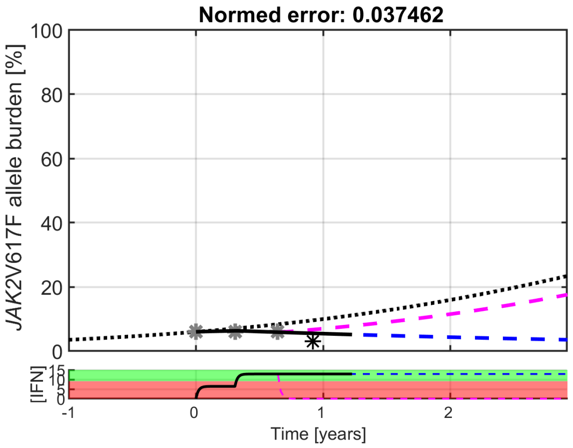

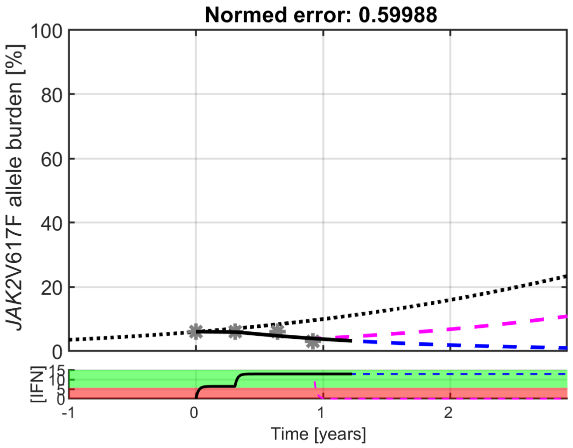

Patient P124

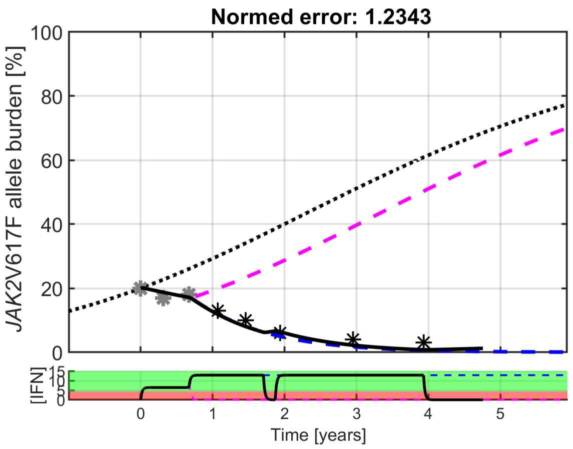

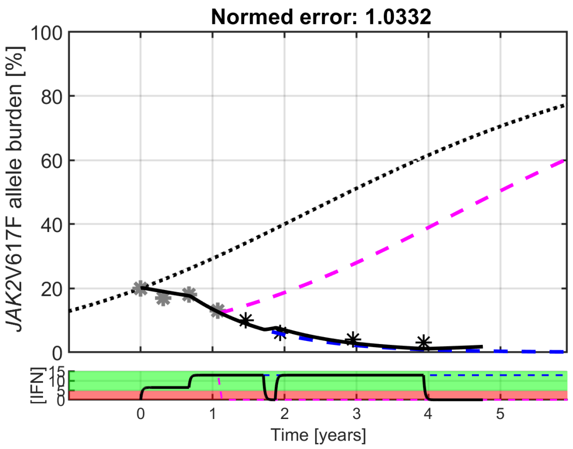

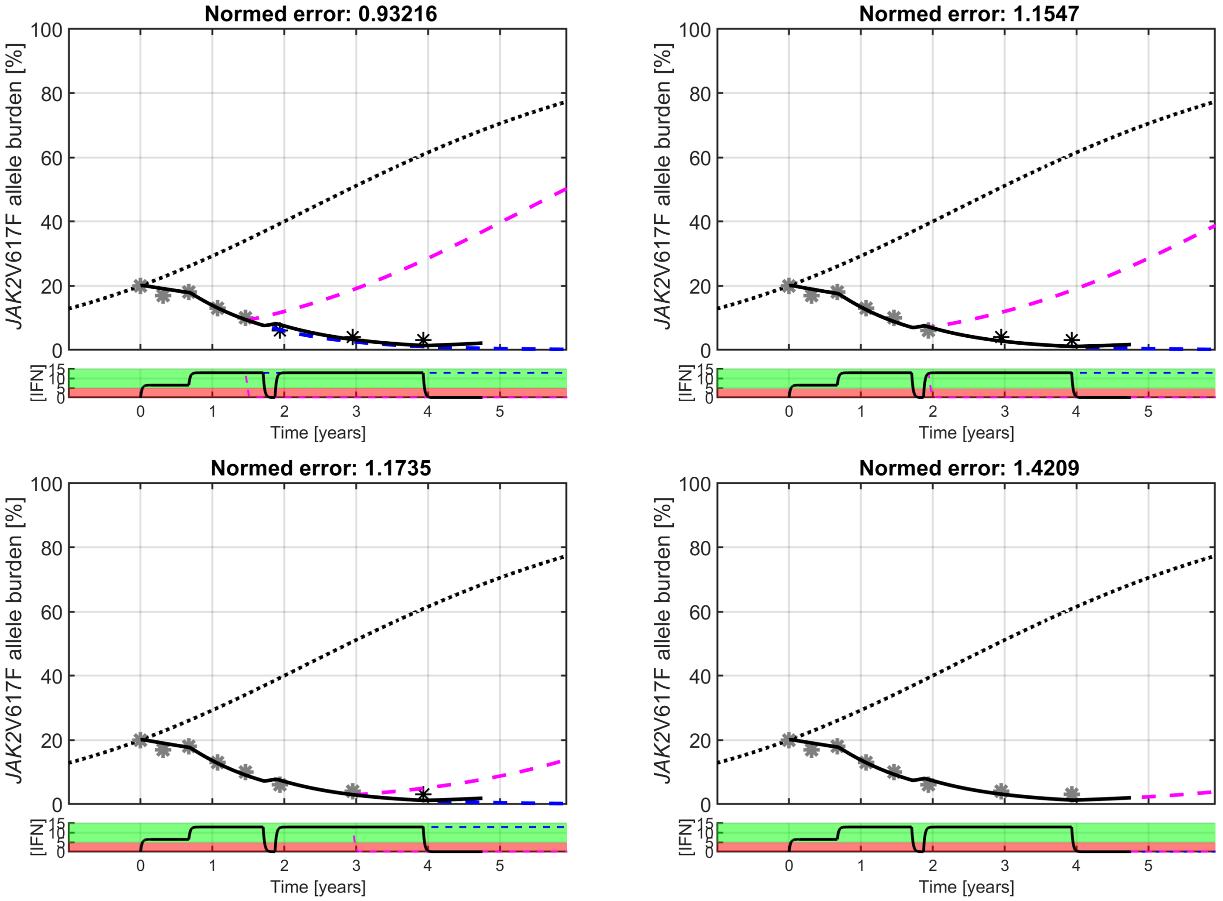

Patient P125

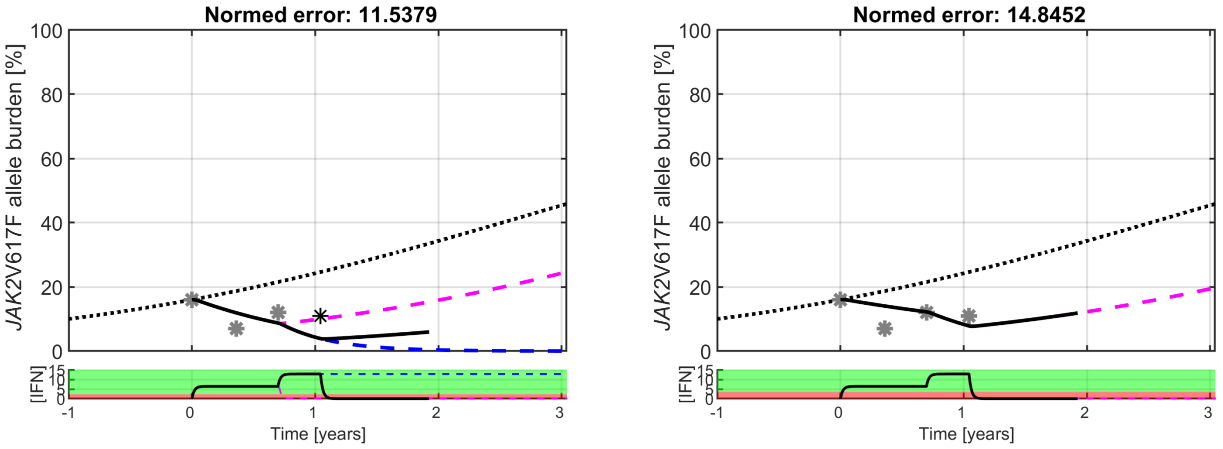

Patient P129

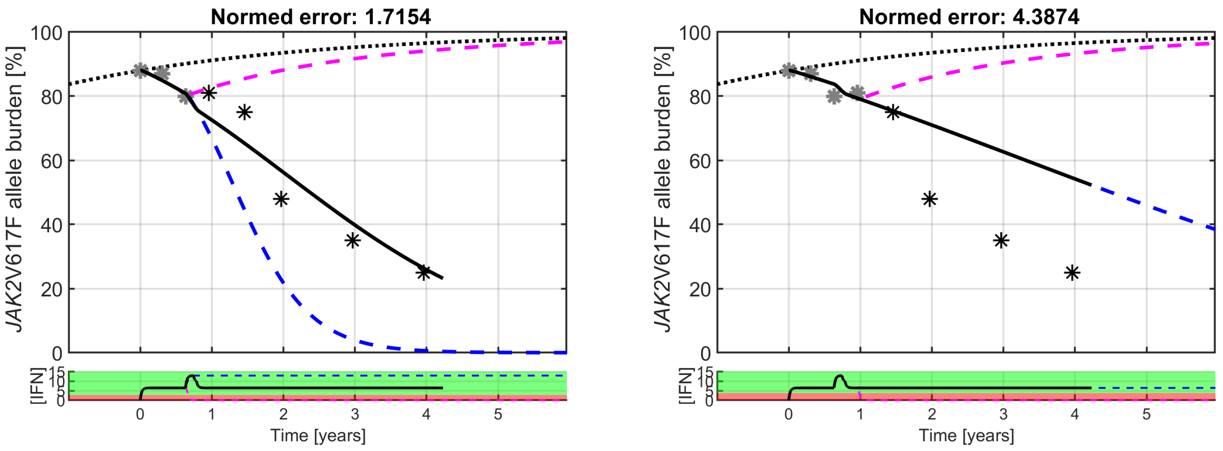

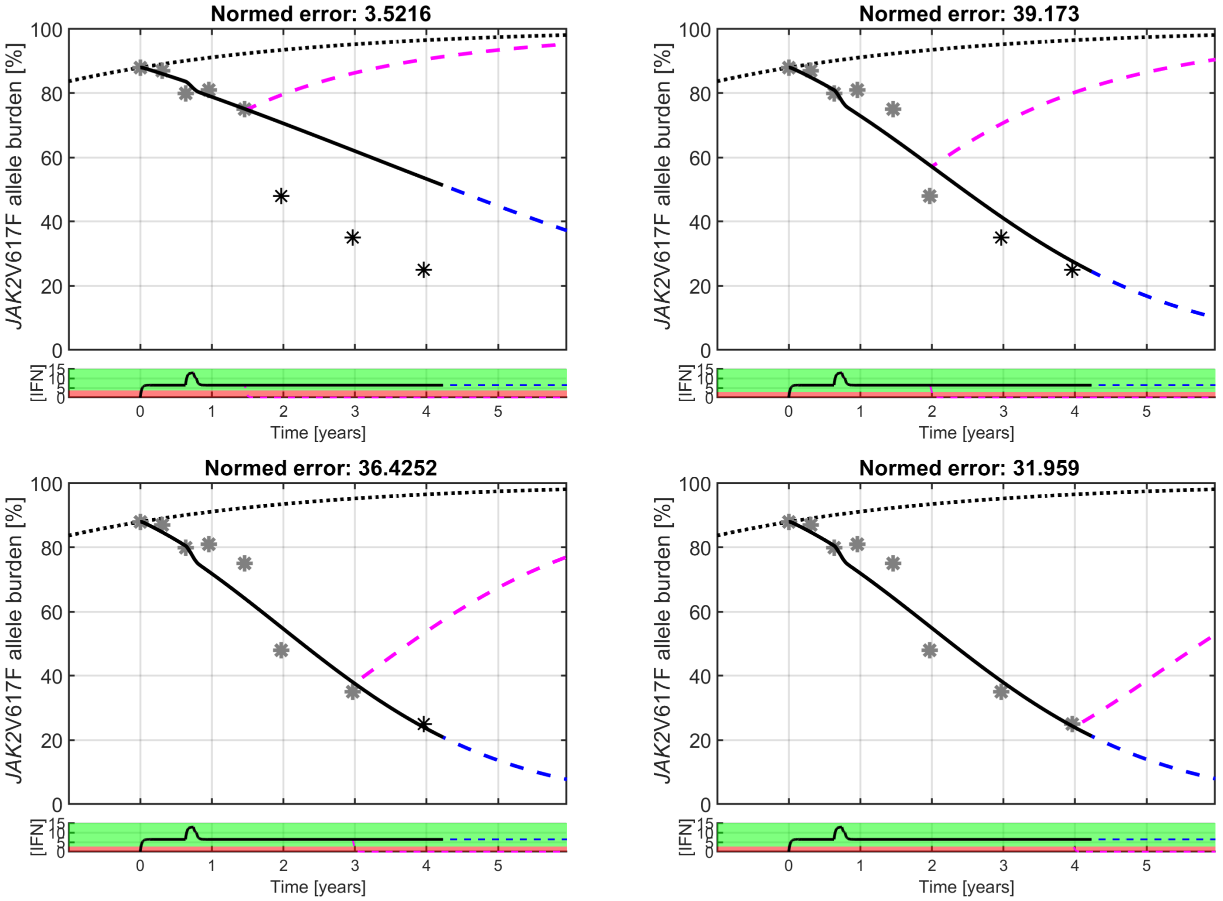

Patient P131

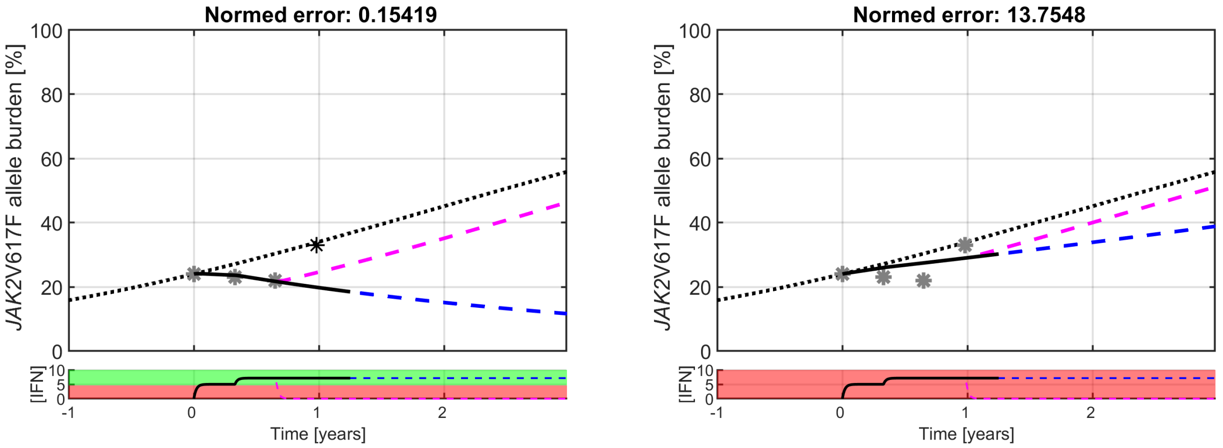

Patient P135

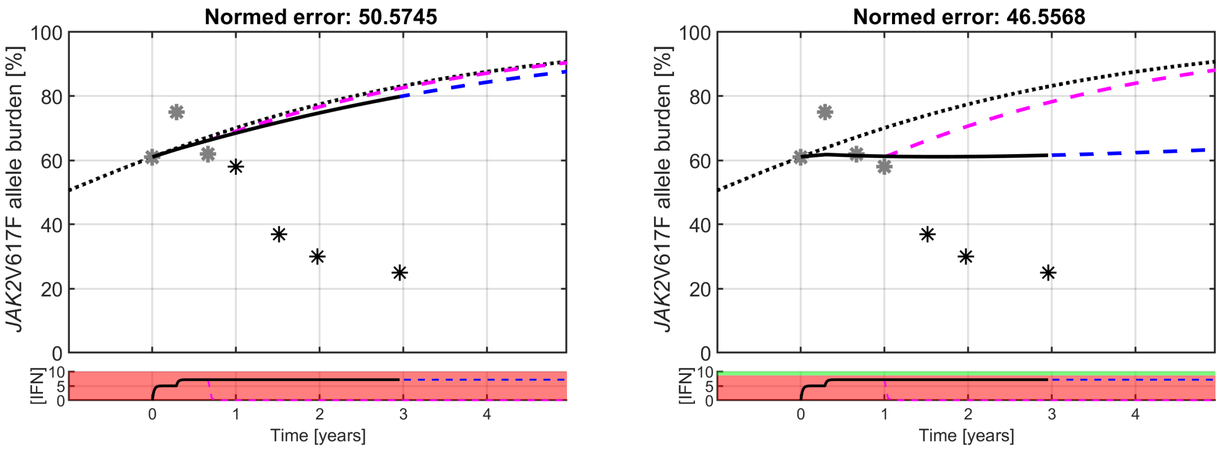

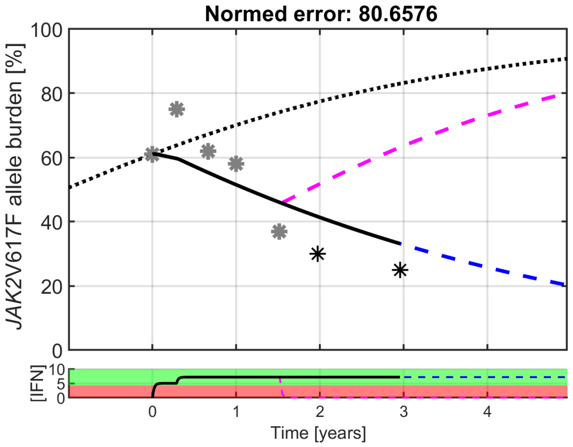

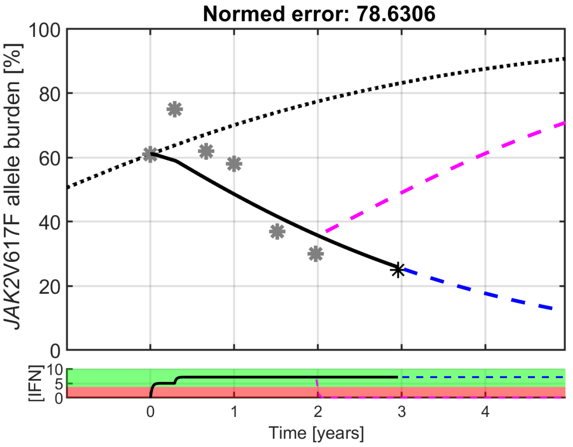

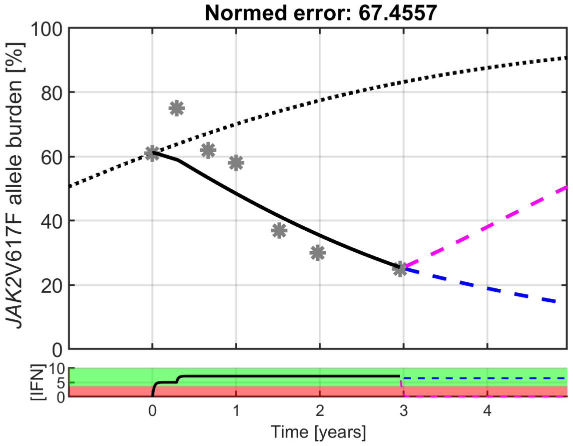

Patient P139

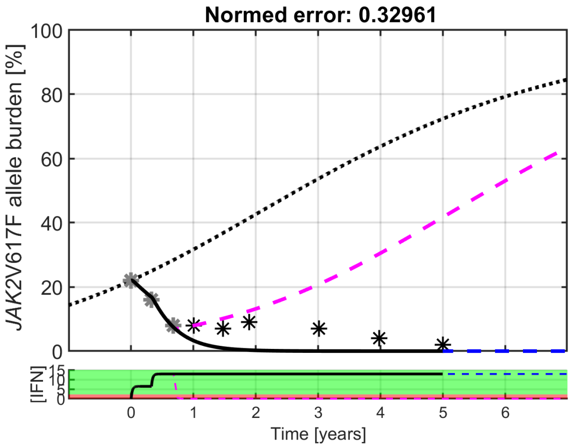

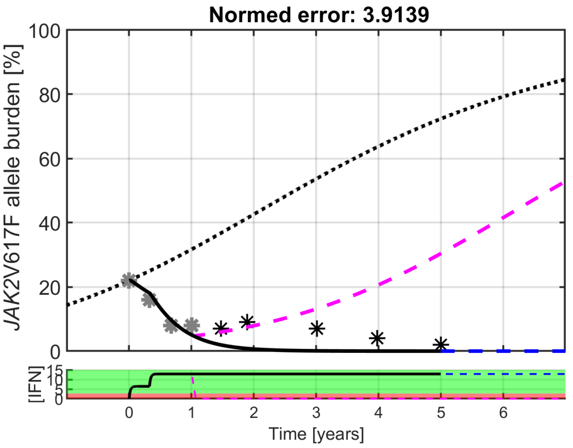

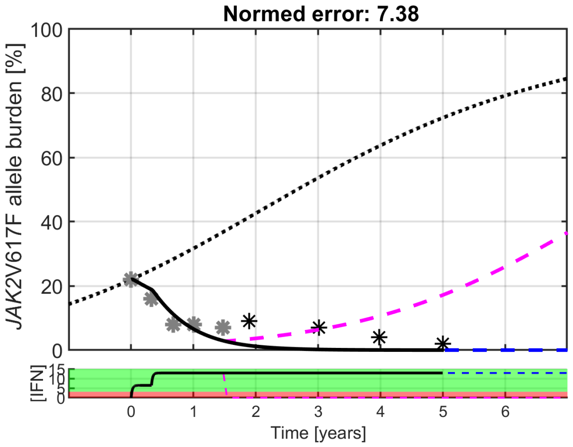

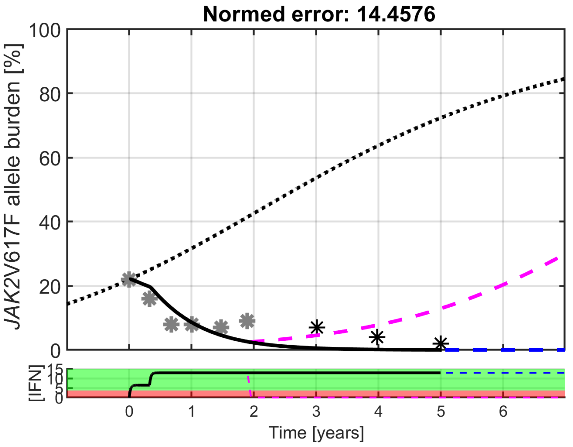

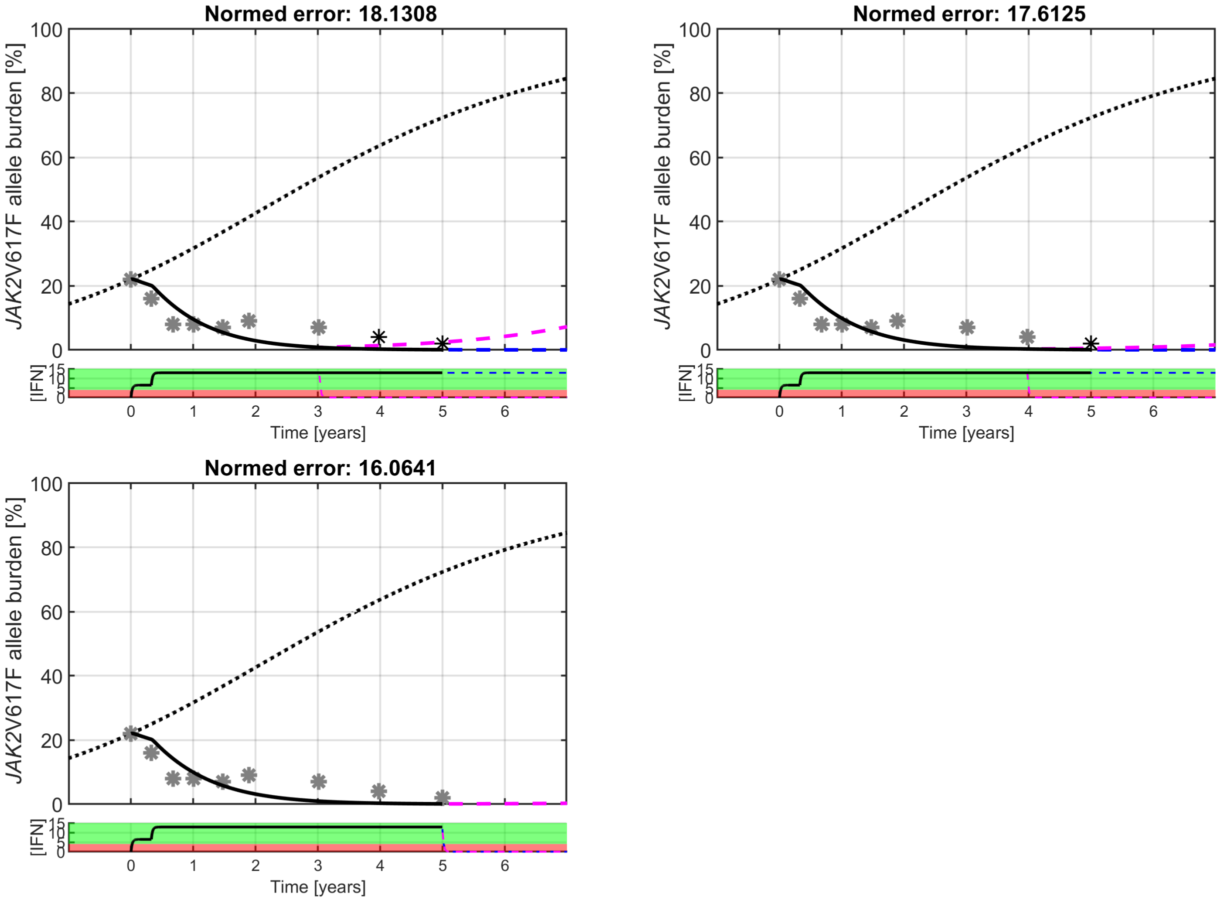

Patient P140

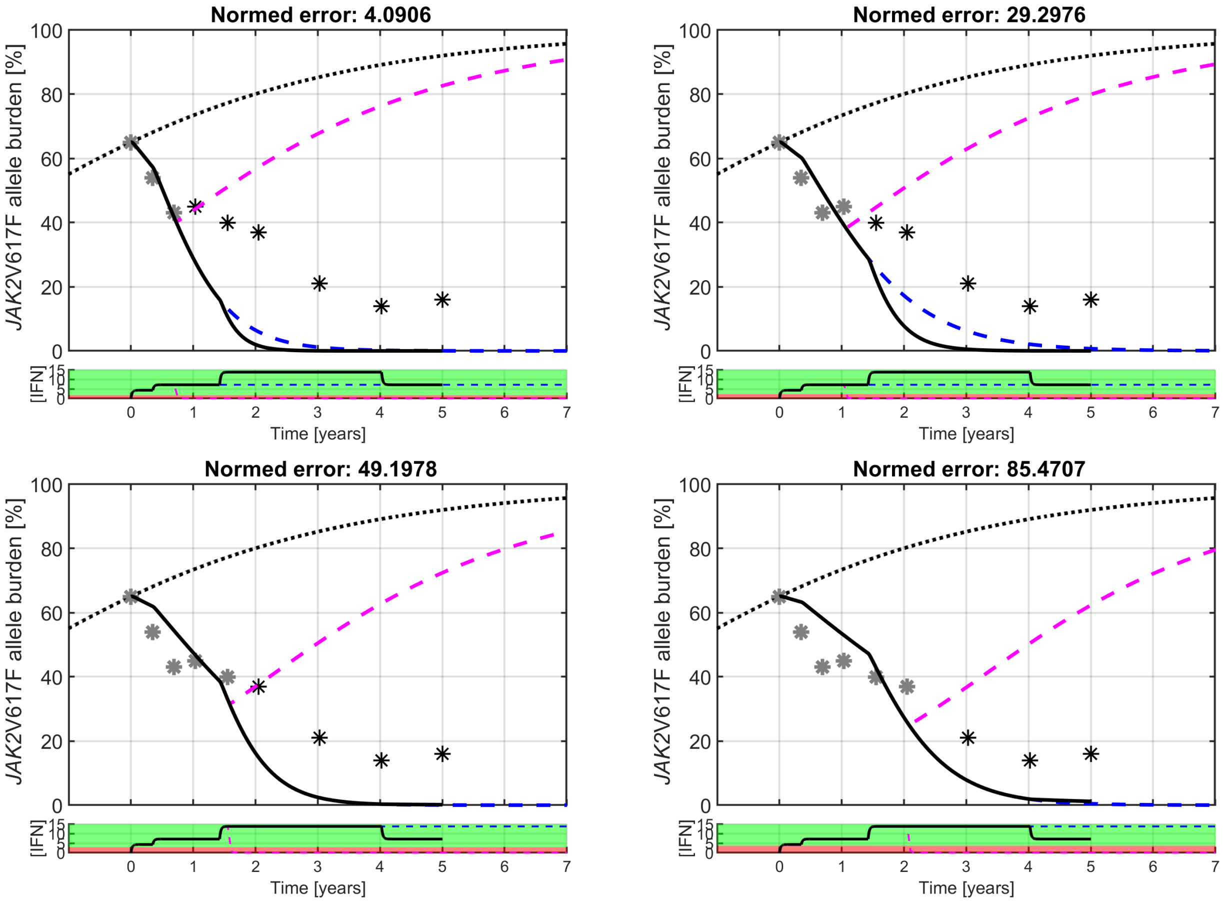

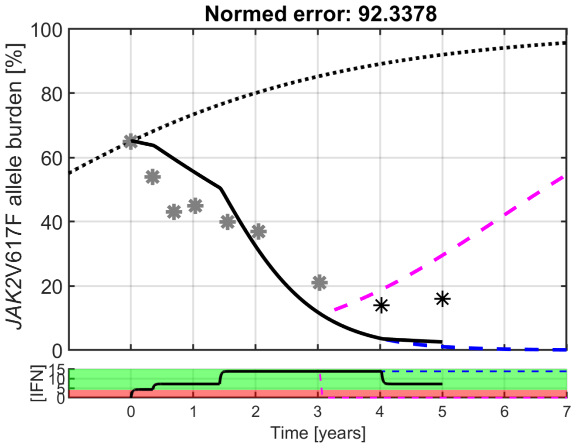

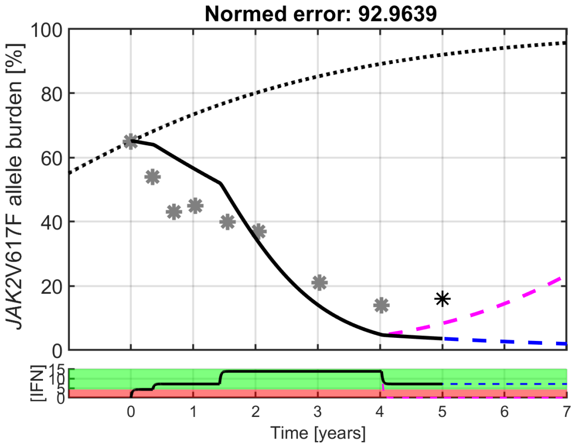

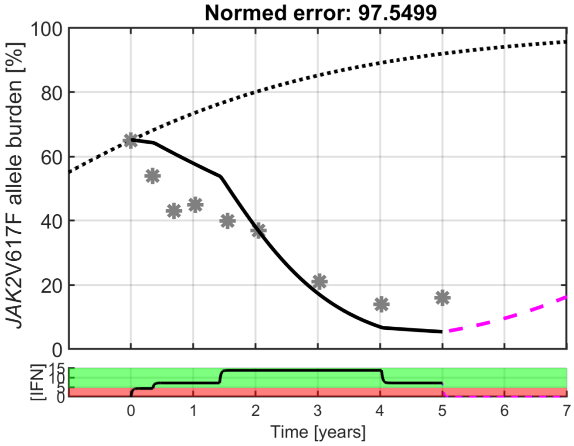

Patient P142

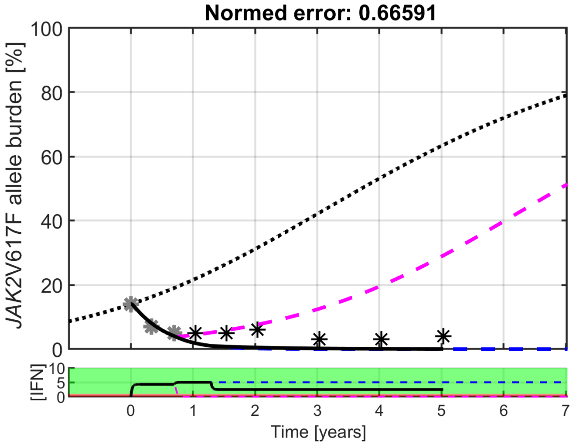

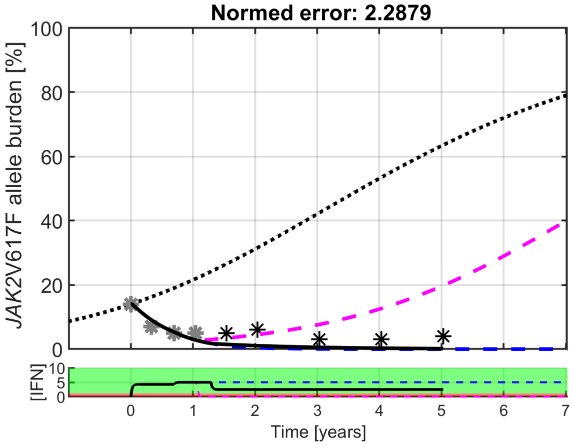

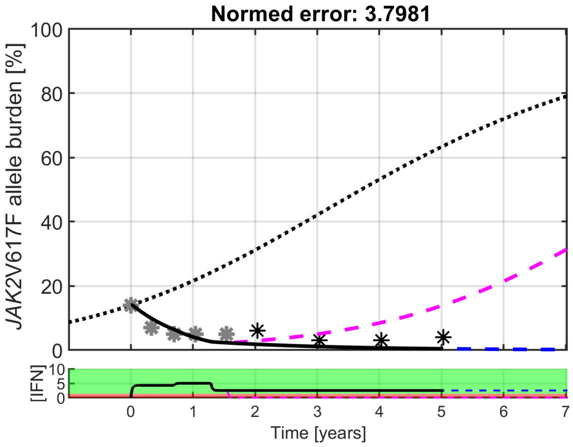

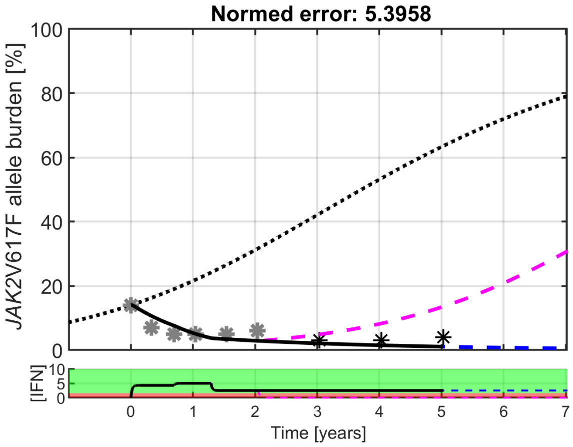

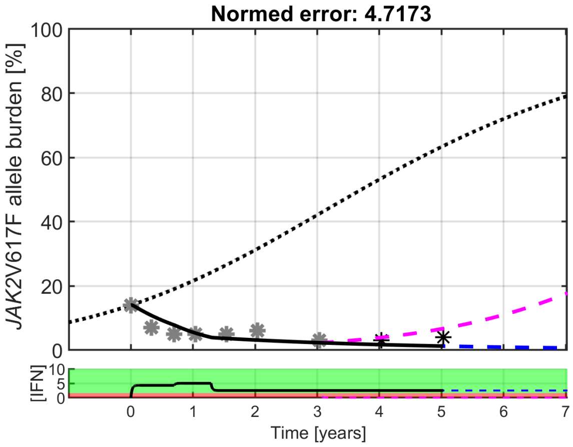

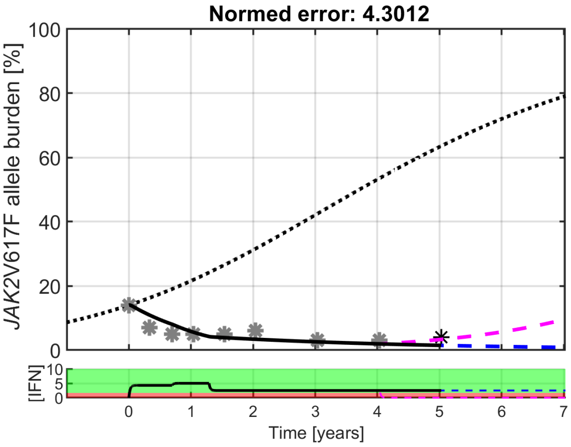

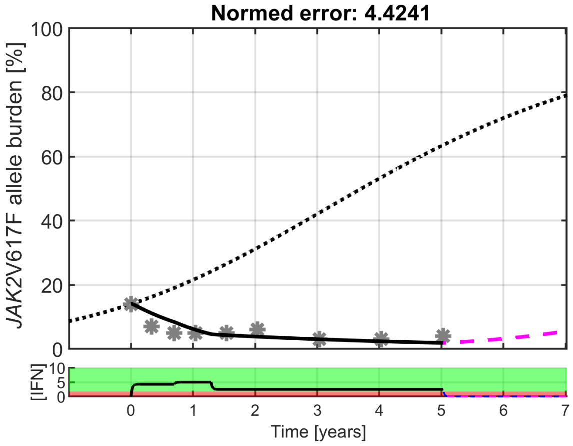

Patient P144

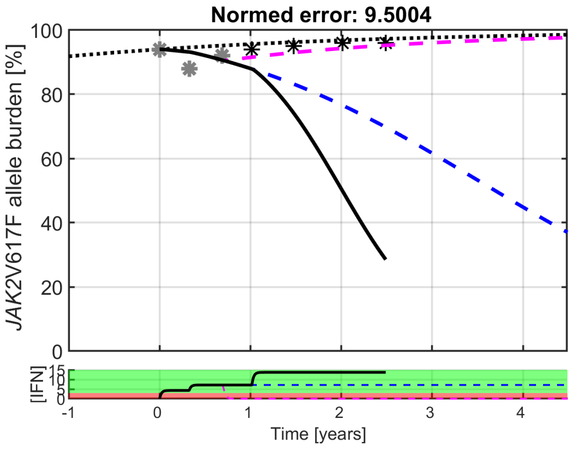

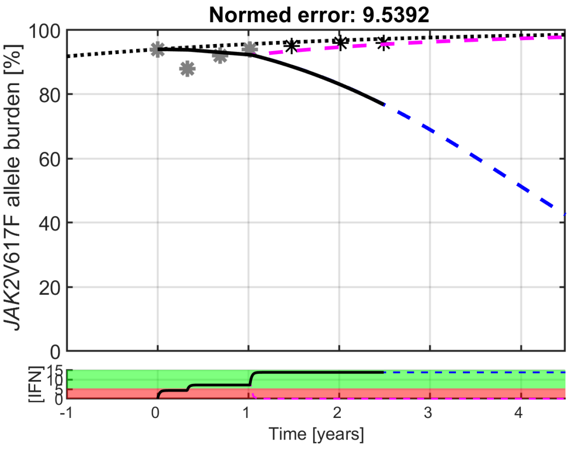

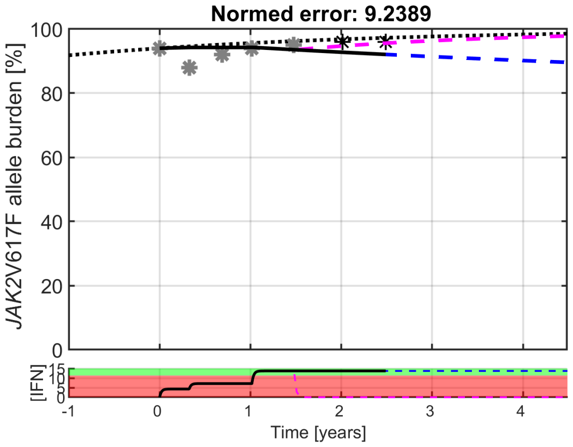

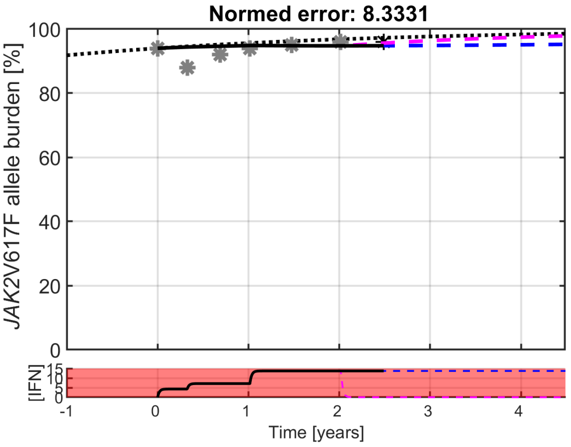

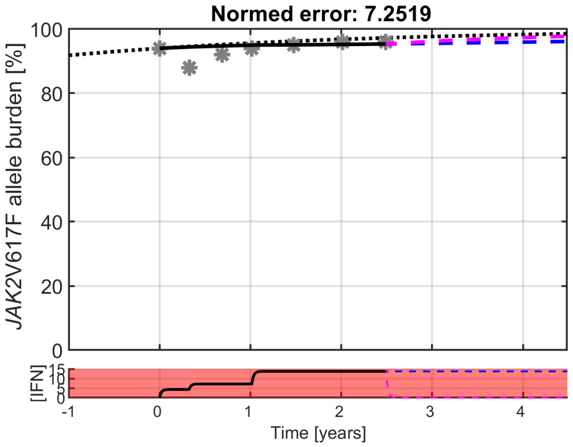

Patient P145

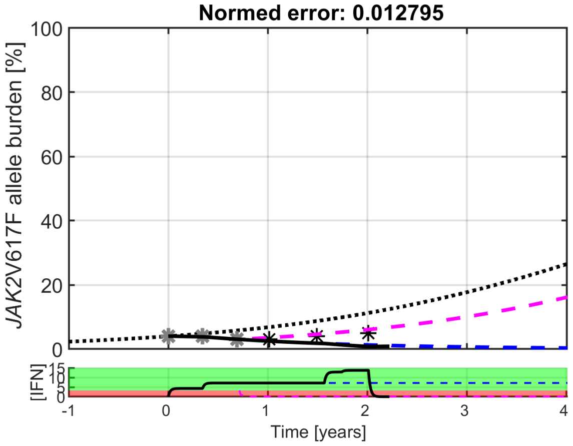

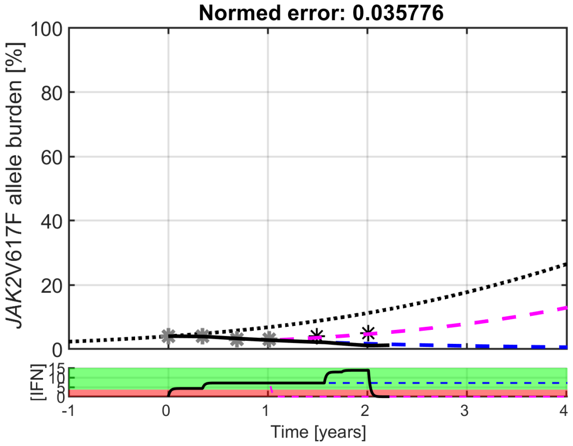

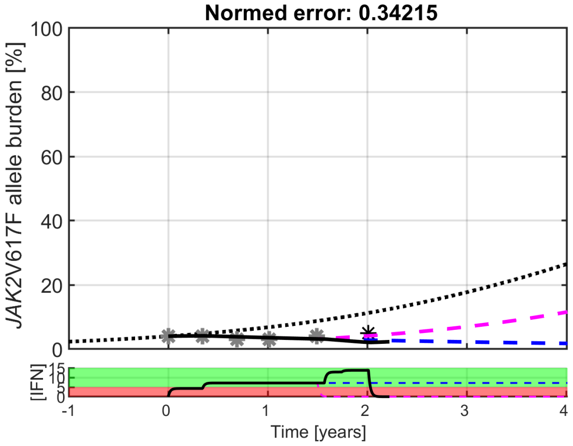

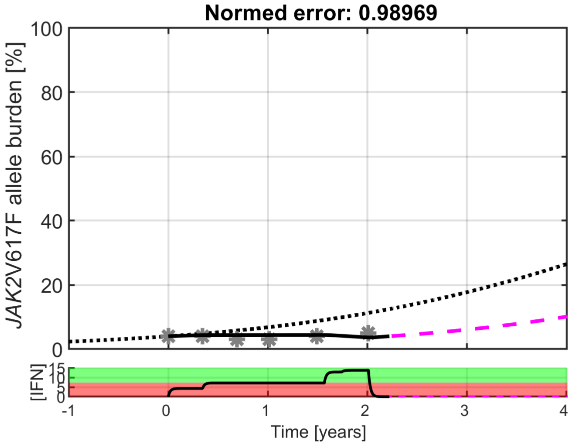

Patient P148

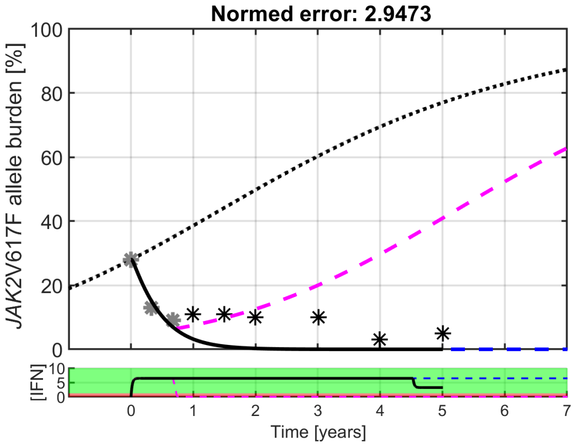

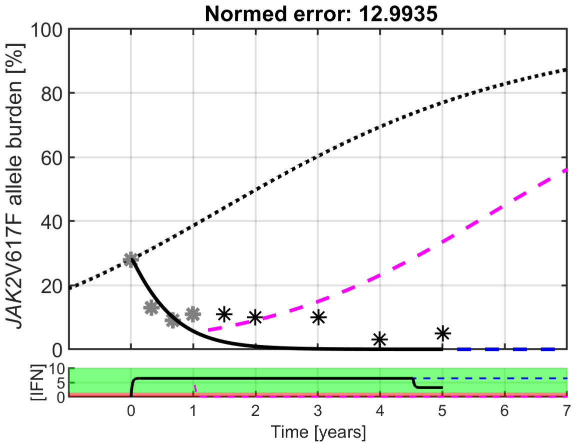

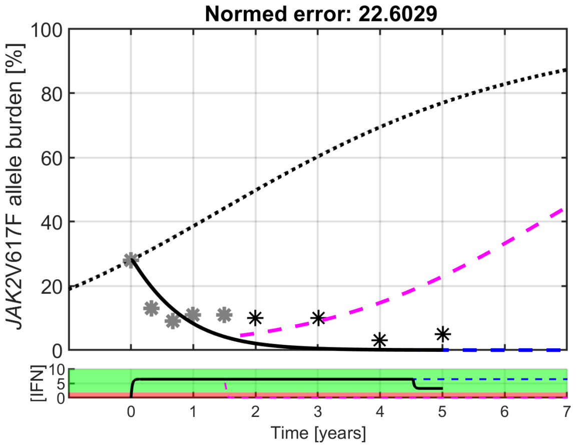

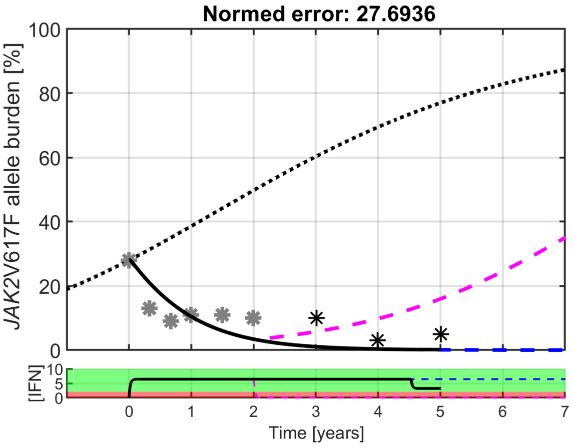

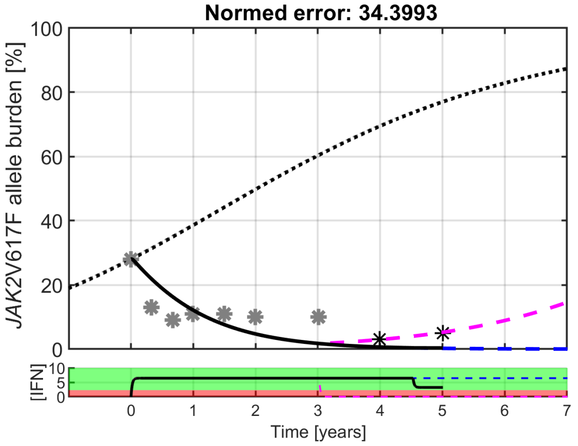

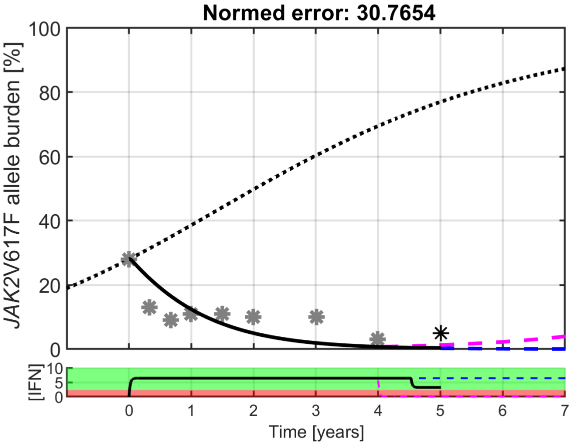

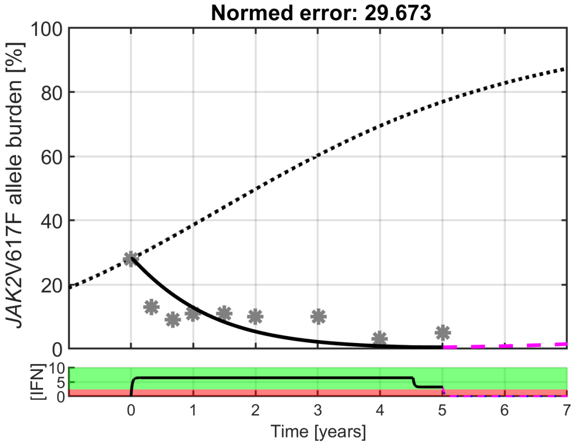

Patient P150

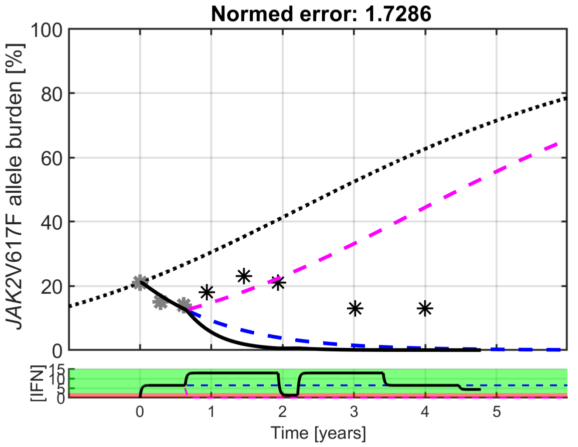

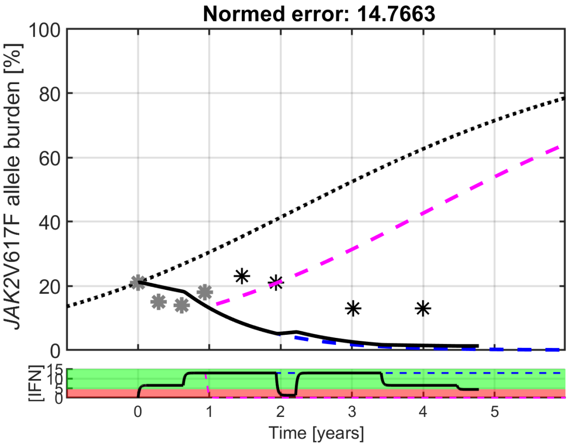

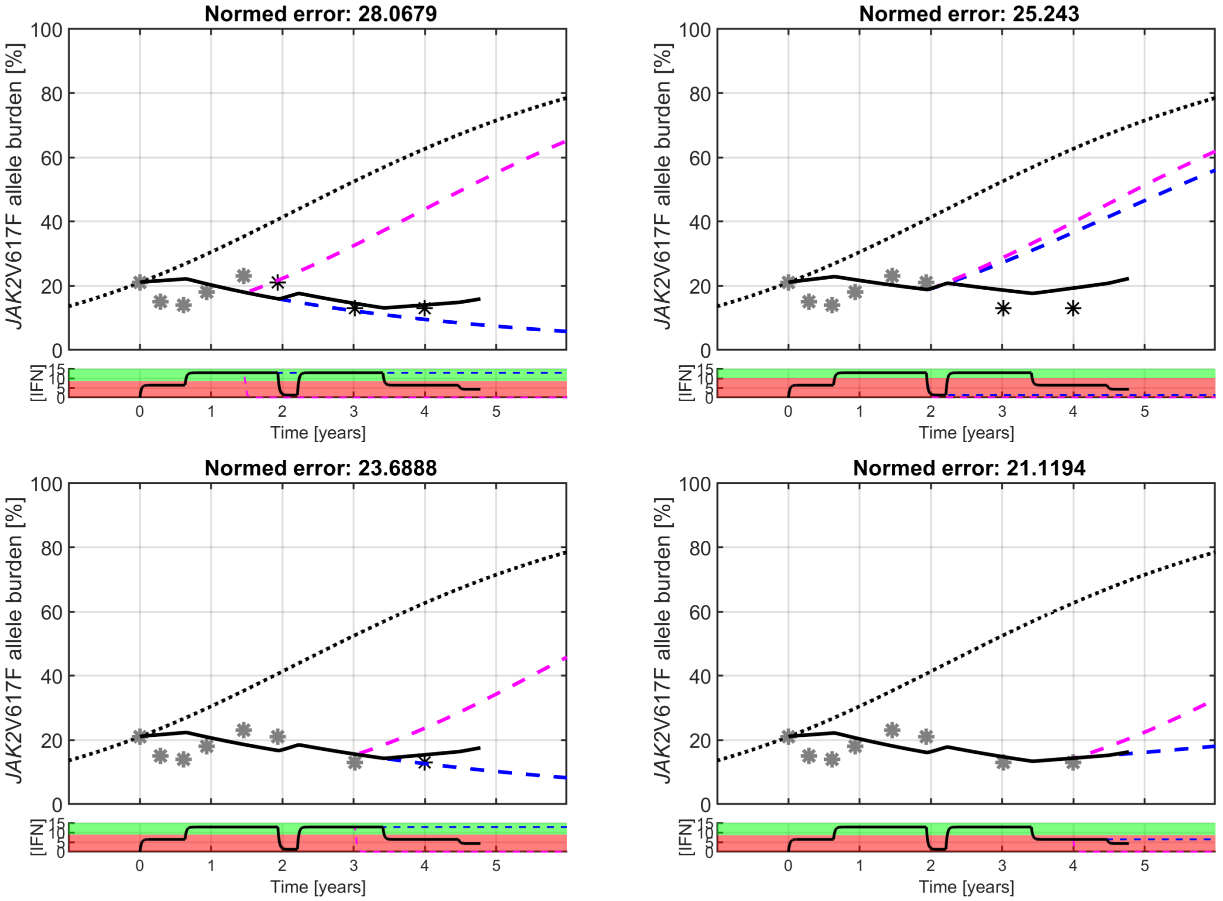

Patient P152

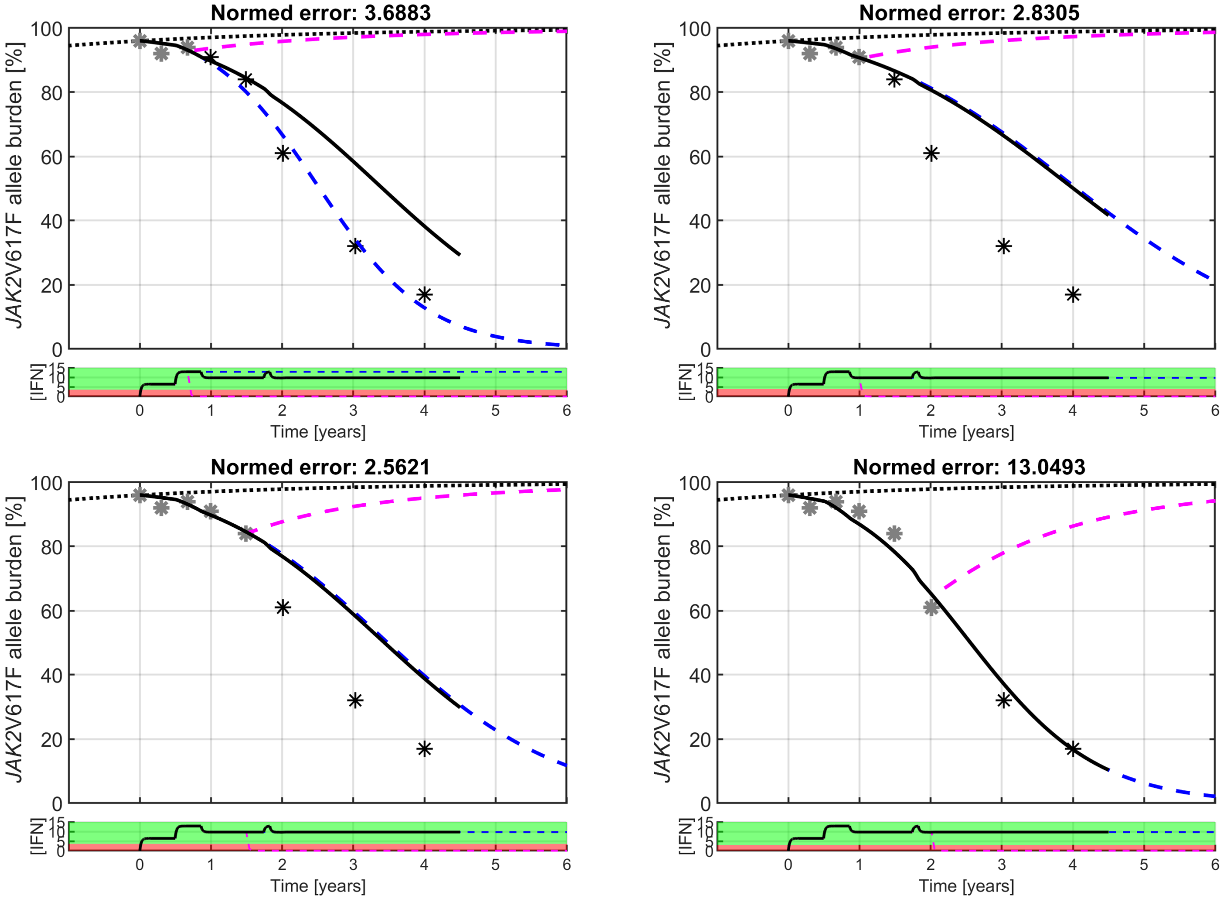

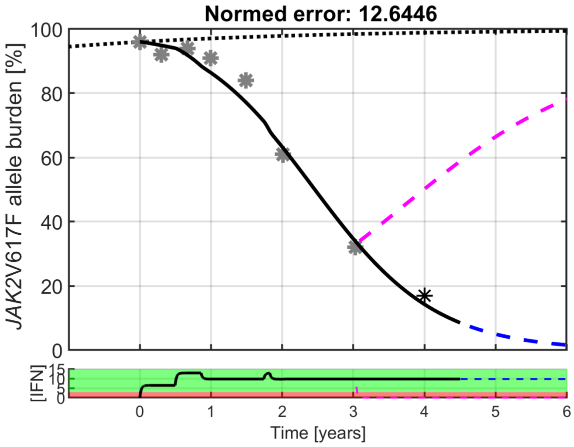

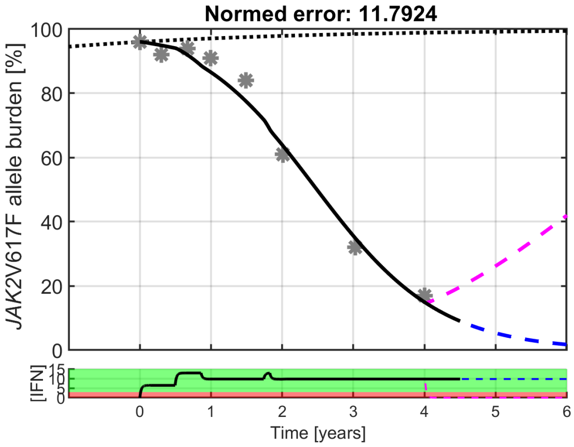

Patient P156

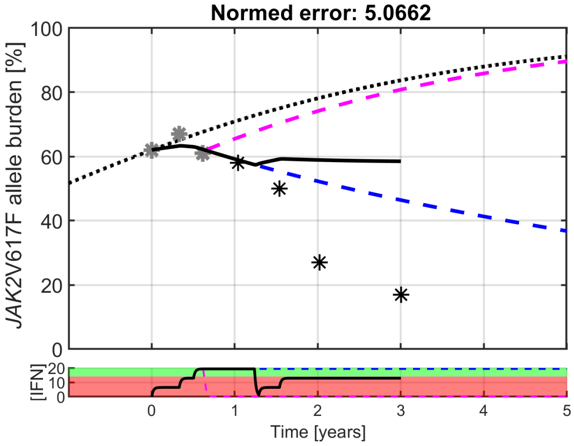

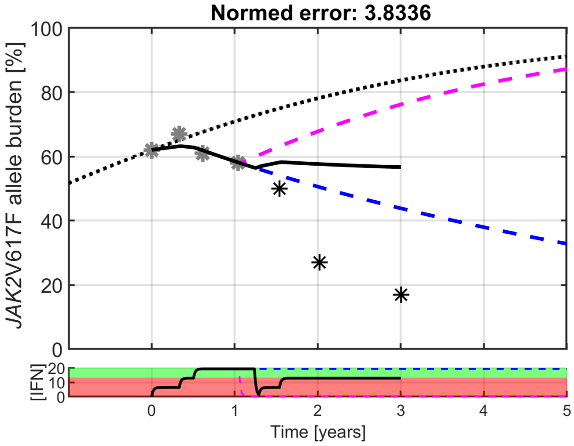

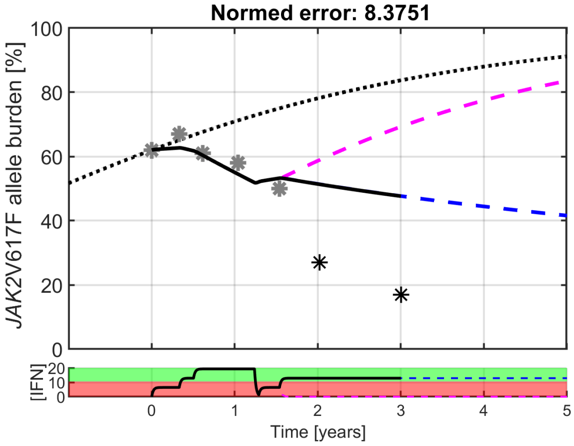

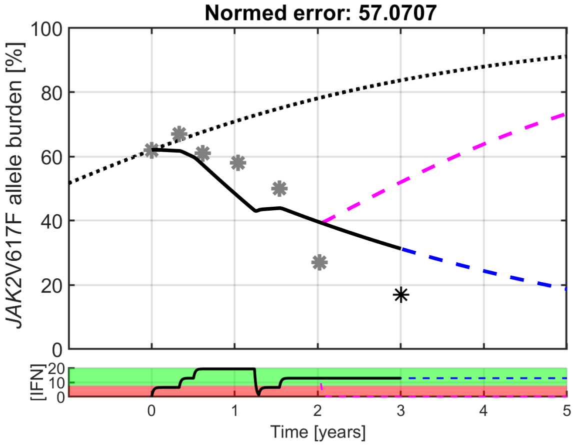

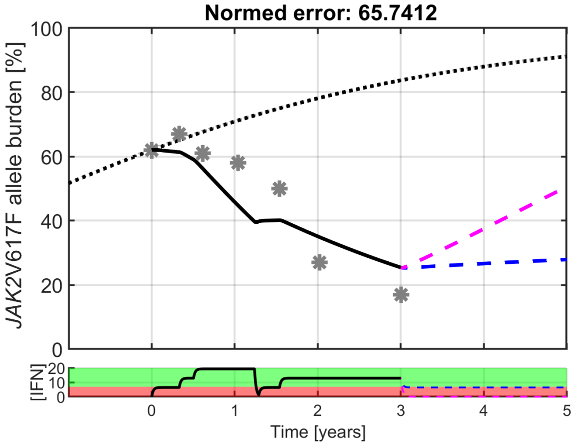

Patient P158

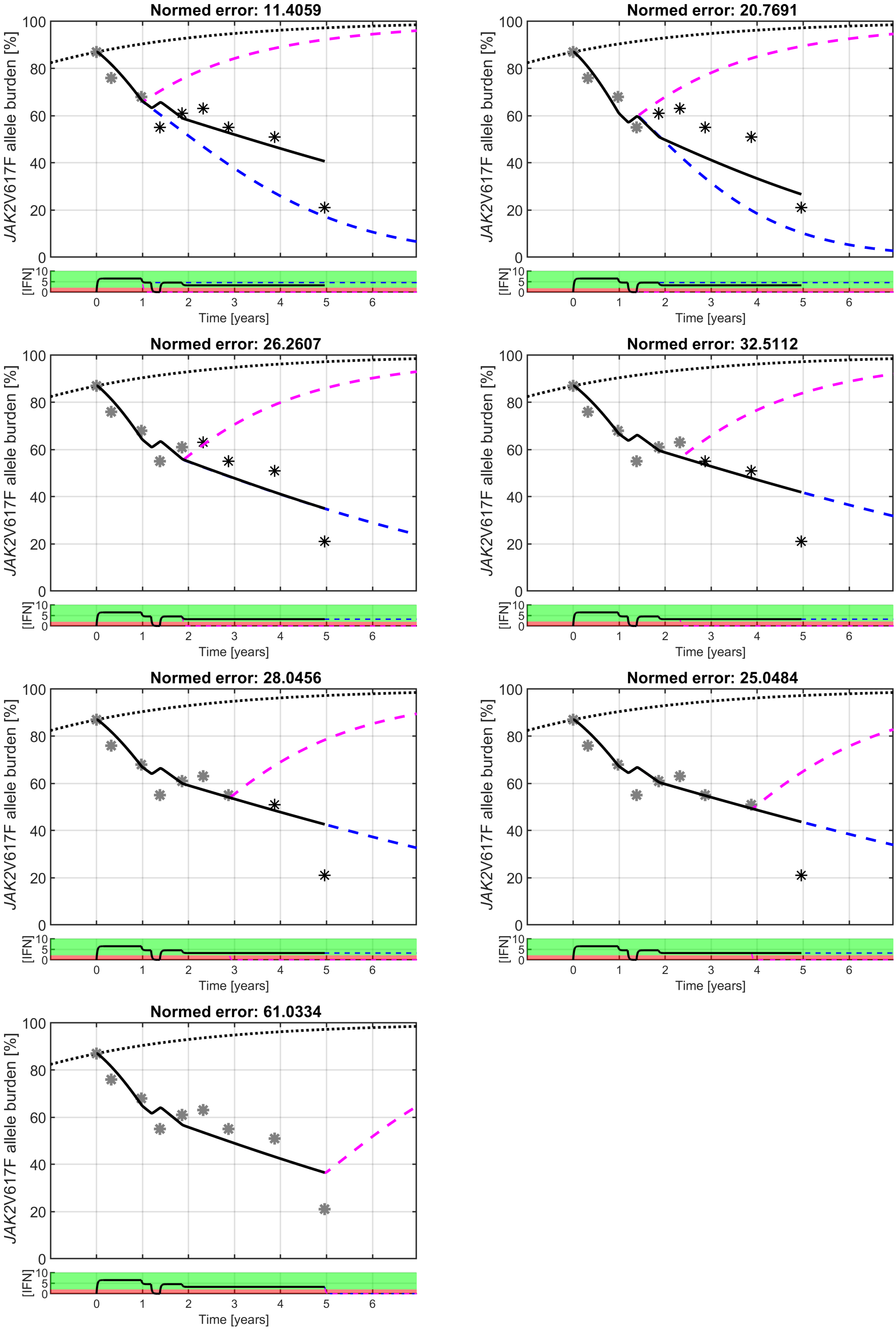

Patient P162

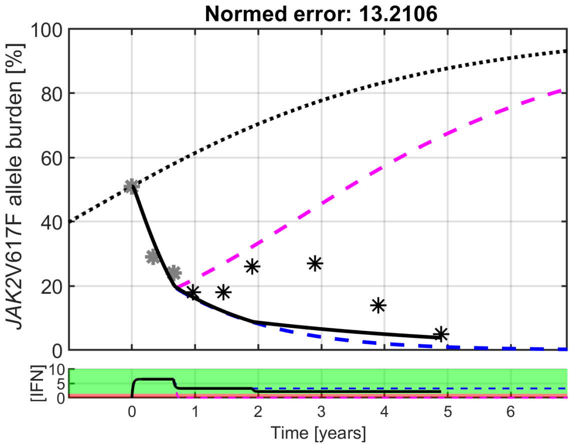

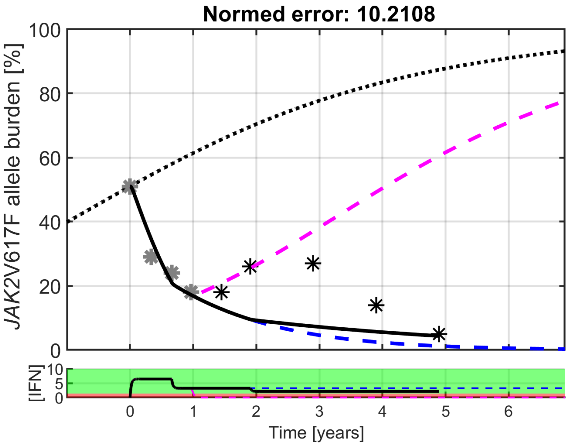

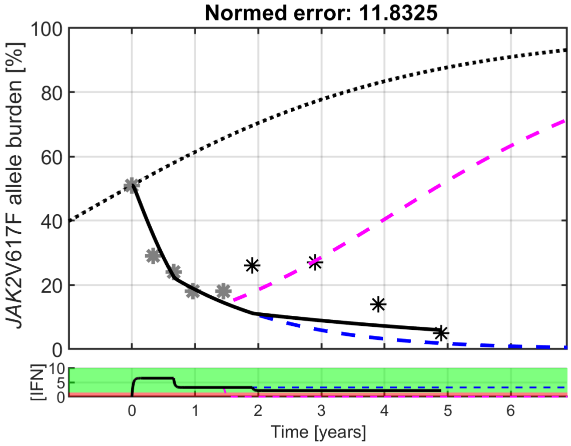

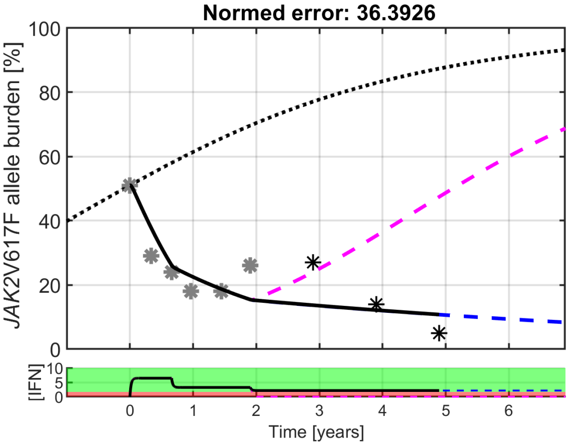

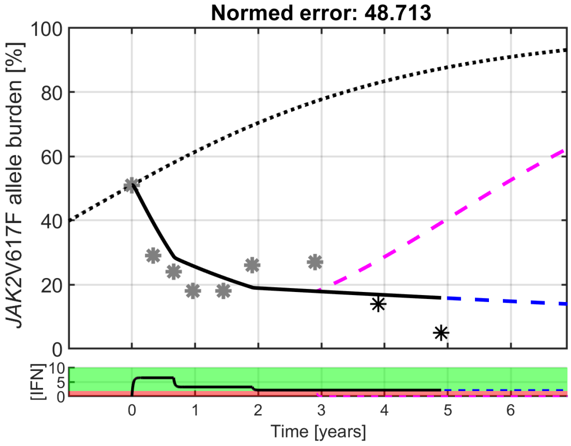

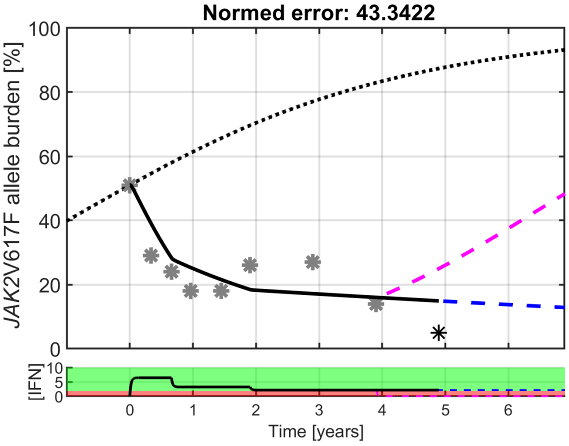

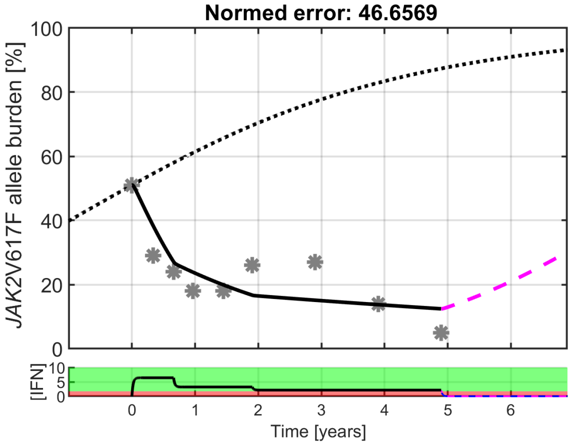

Patient P163

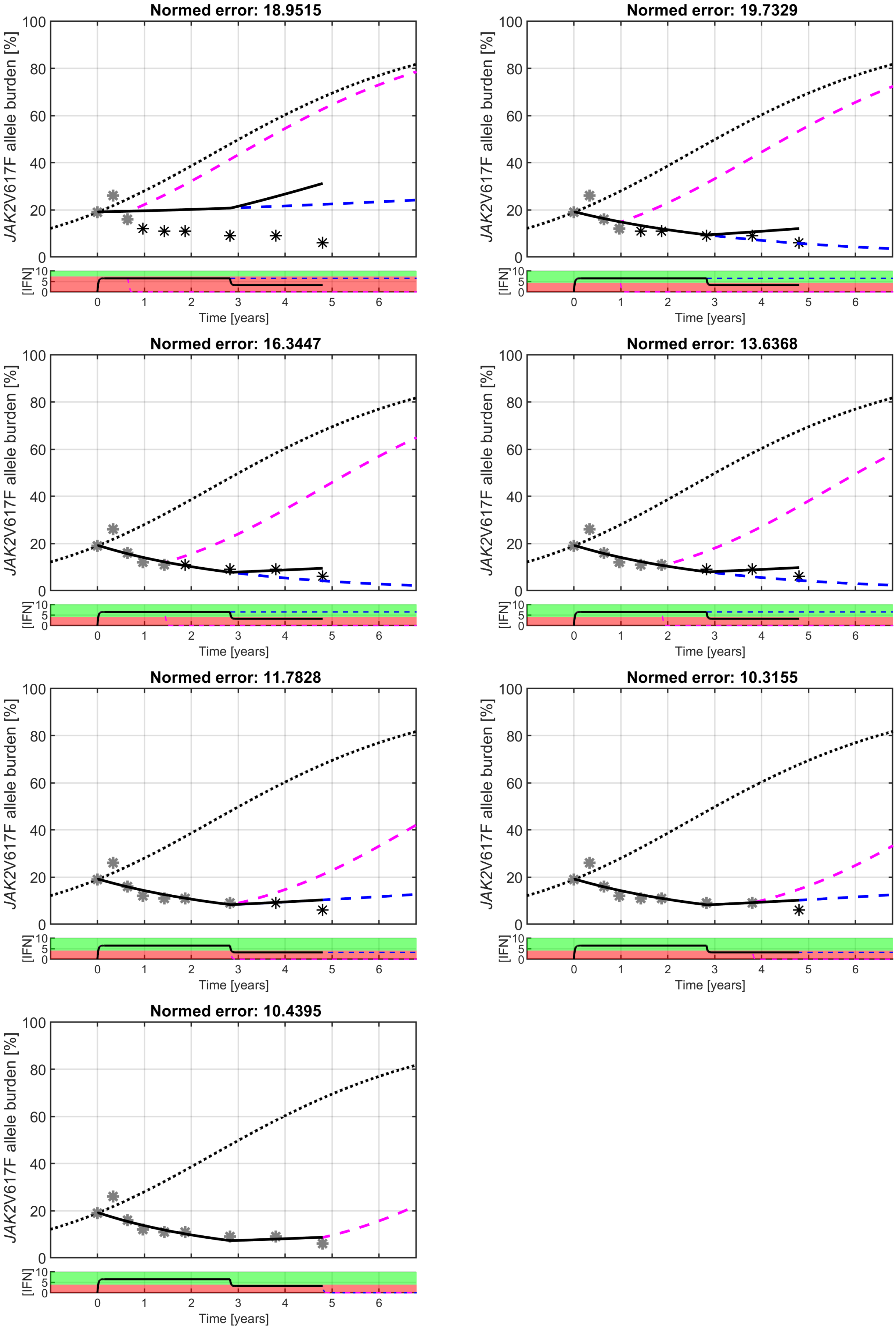

Patient P164

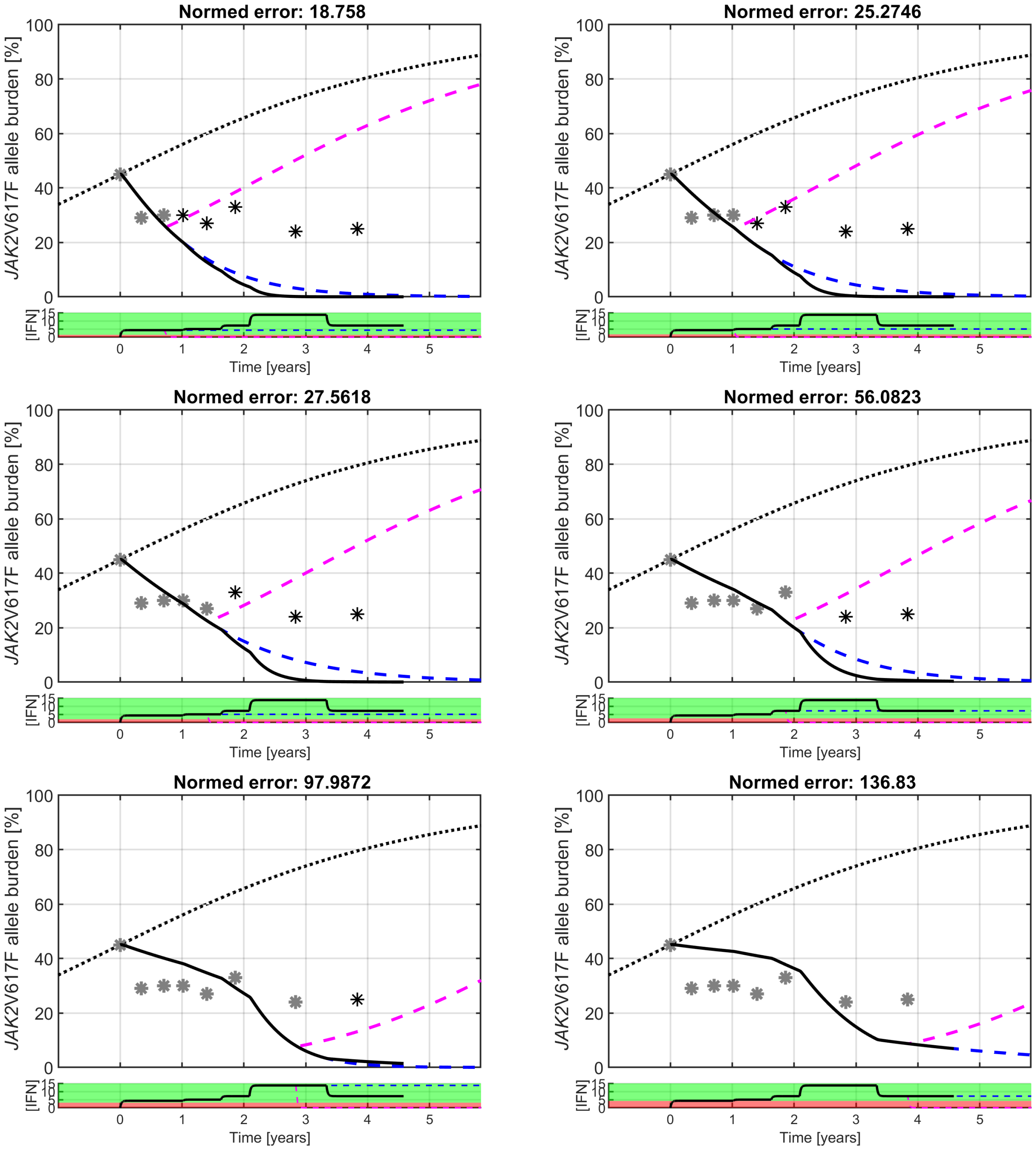

Patient P167

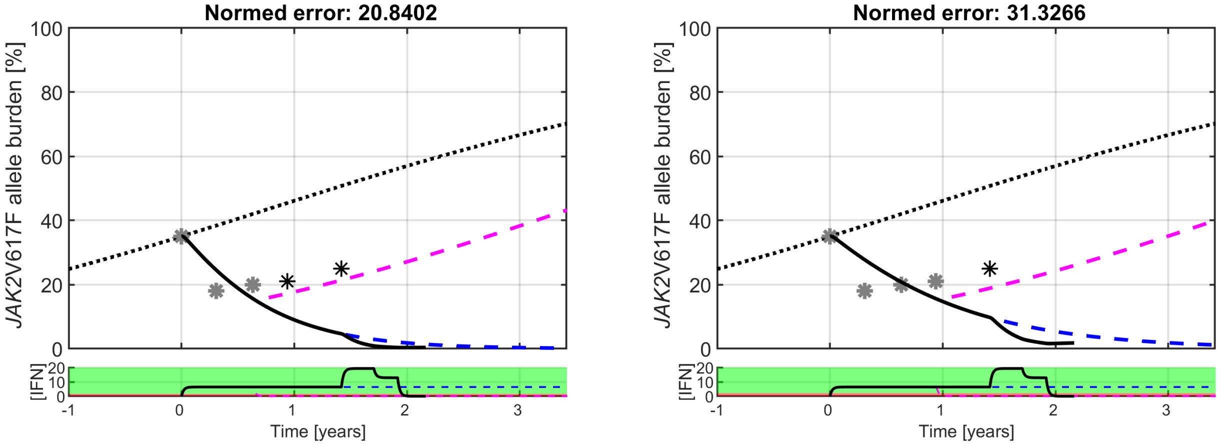

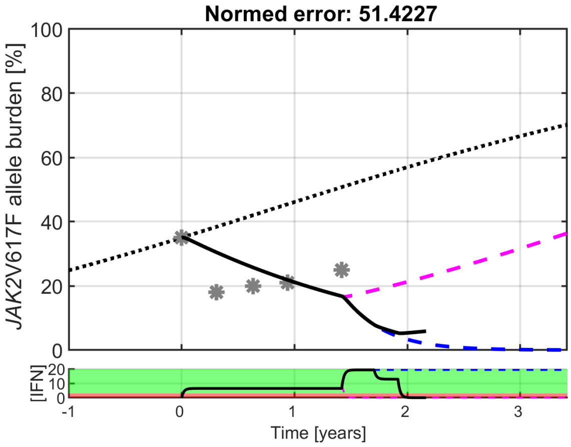

Patient P184

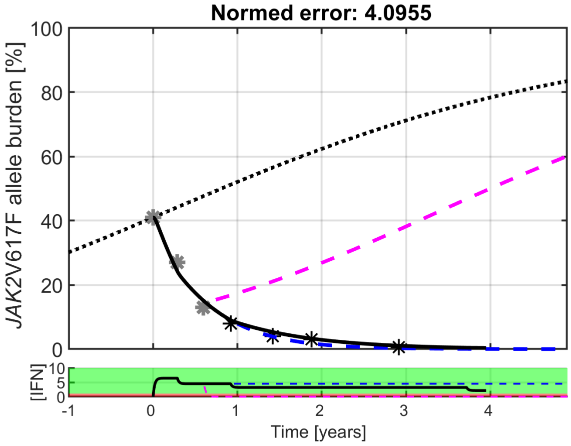

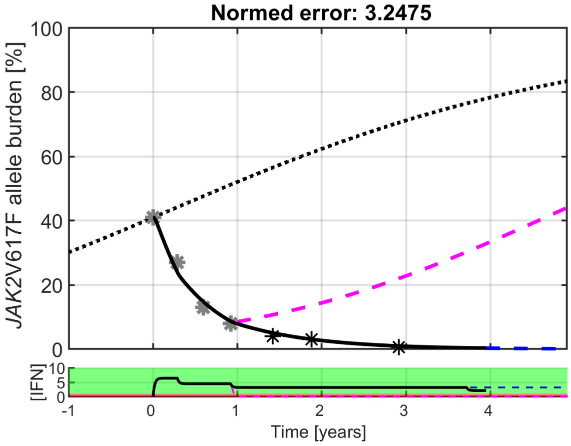

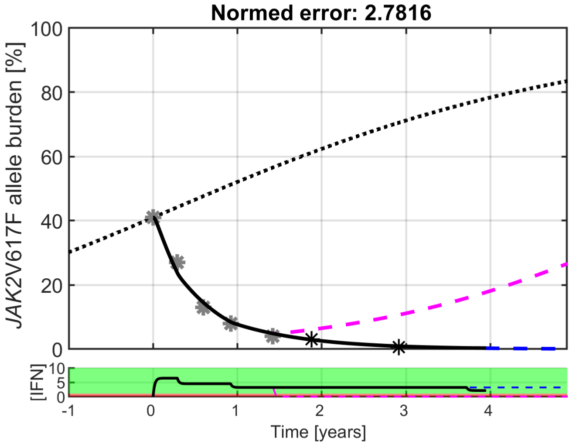

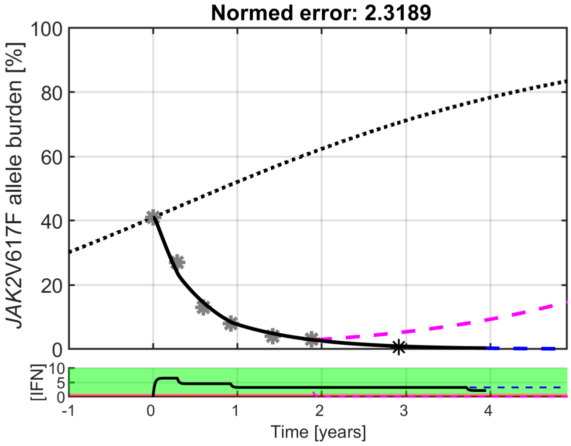

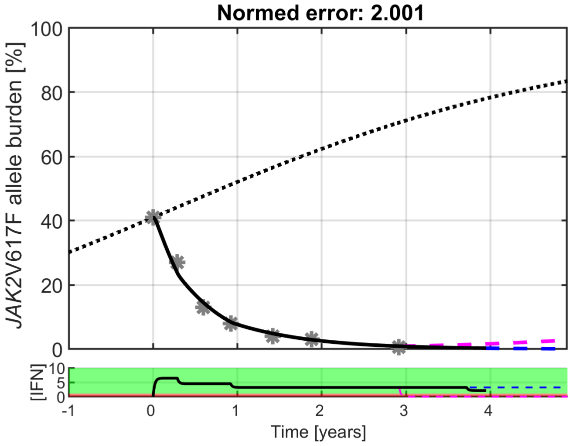

Patient P187

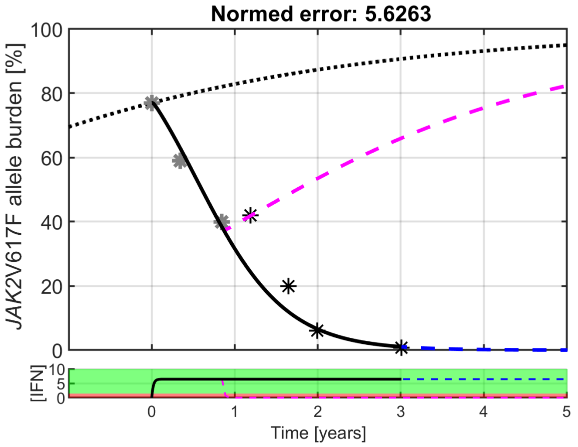

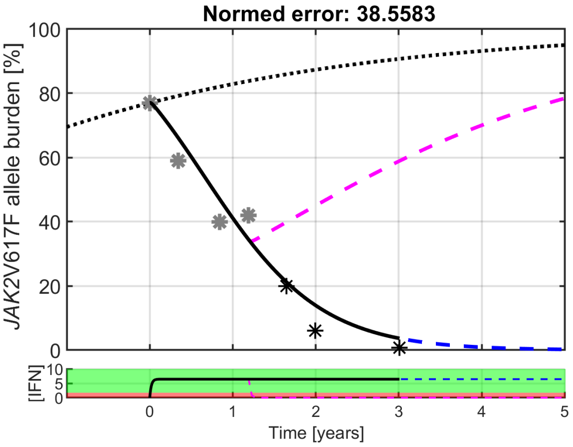

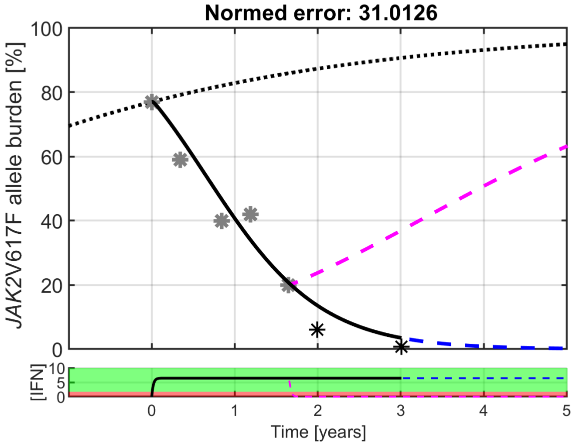

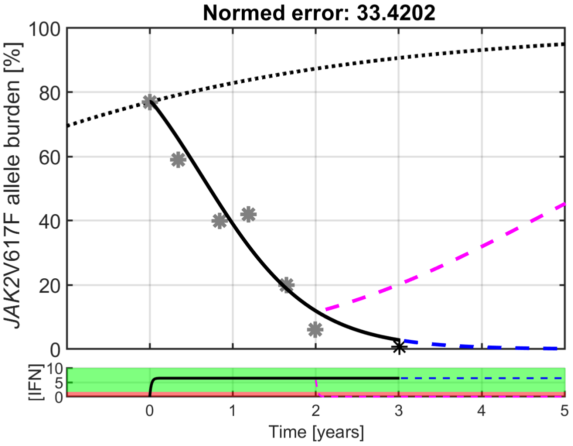

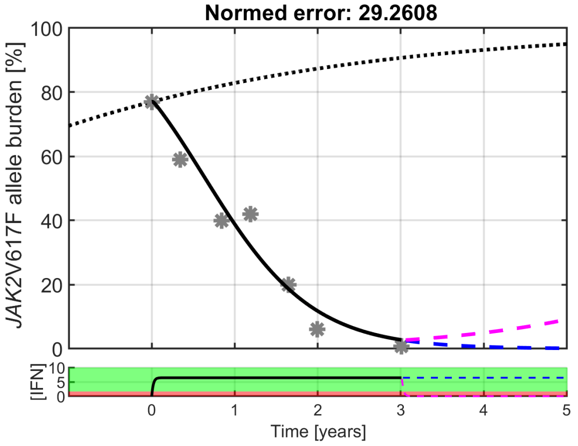

Patient P190

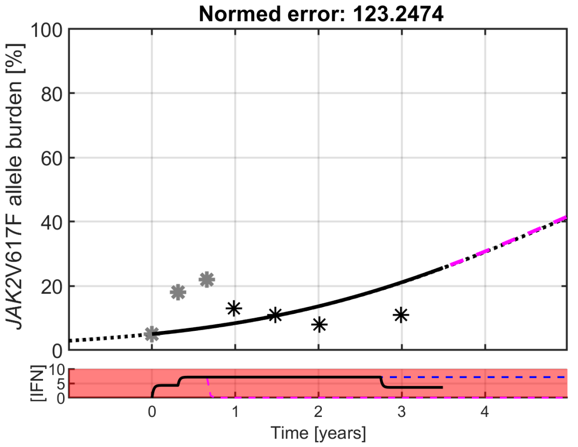

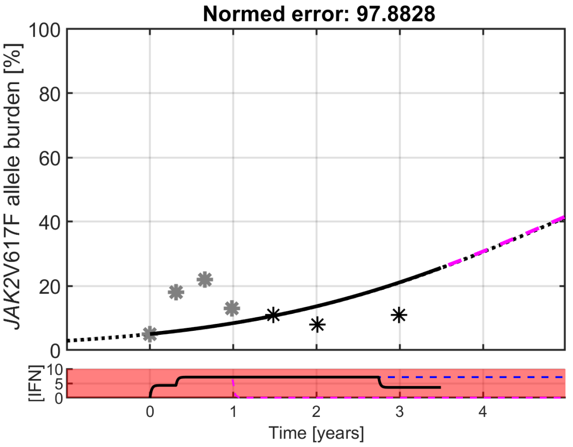

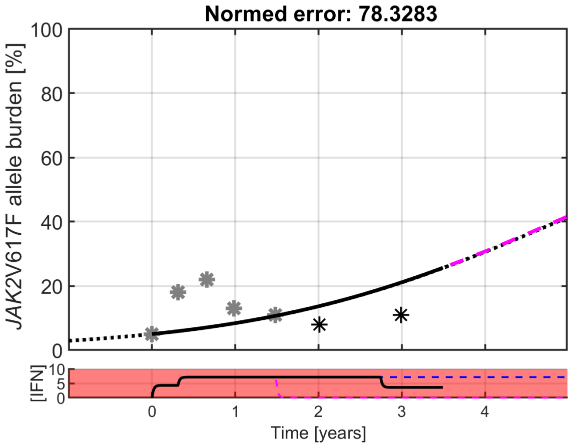

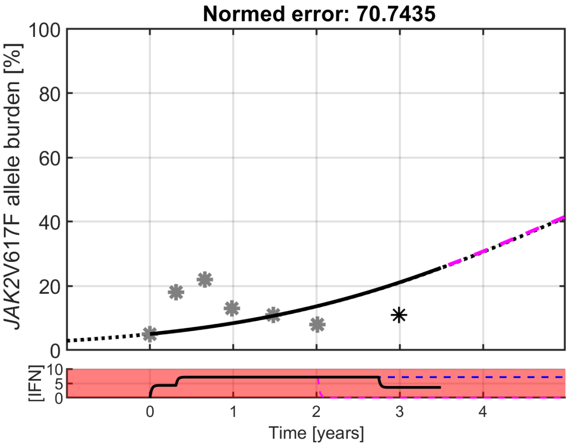

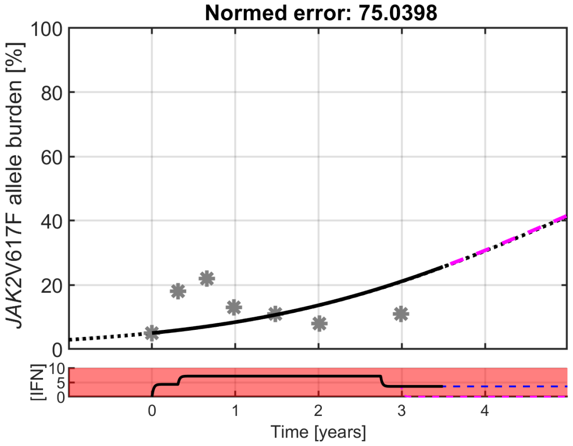

Patient P196

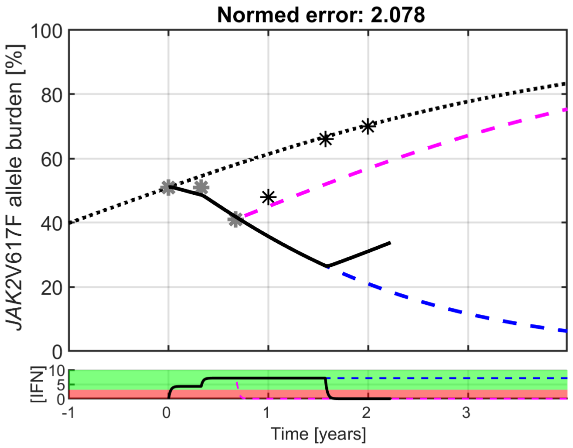

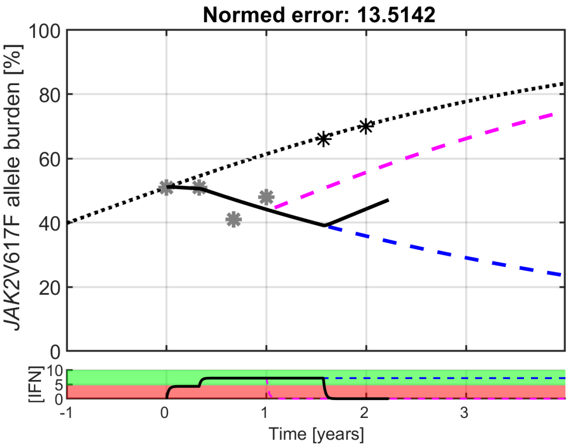

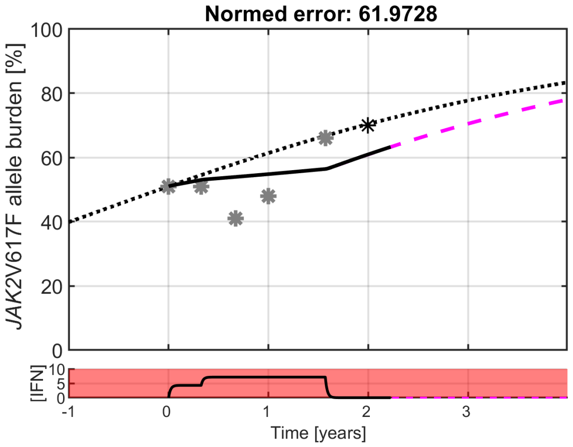

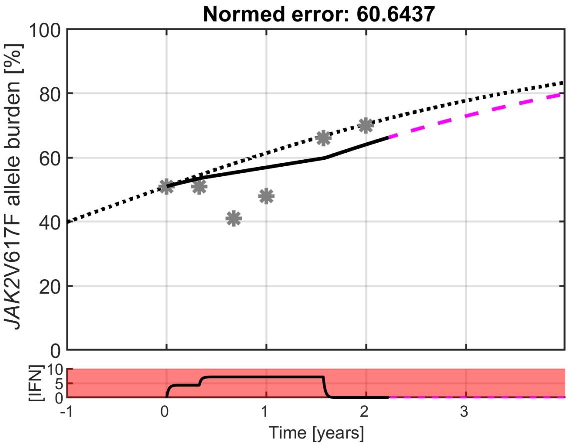

Patient P197

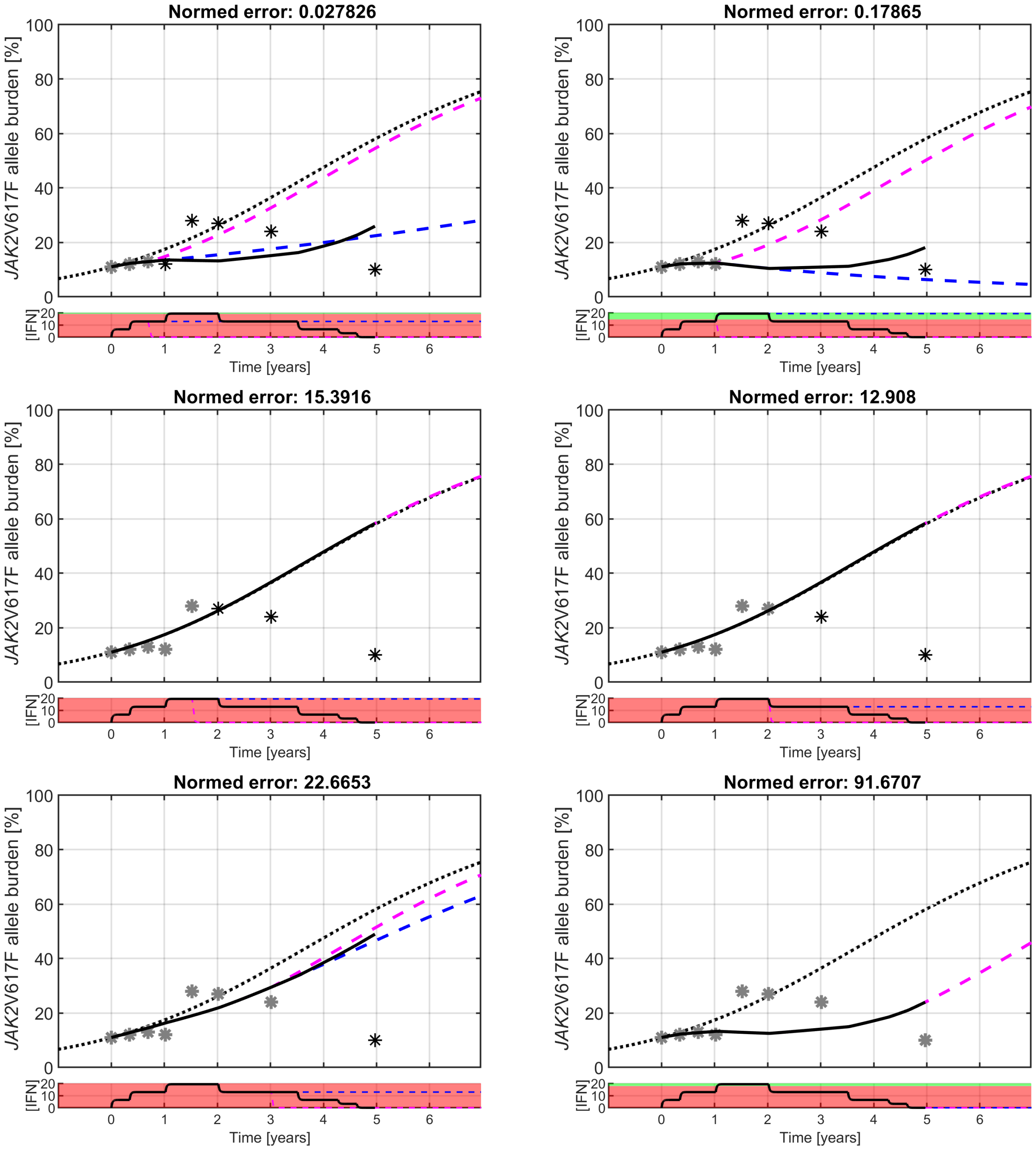

Patient P198

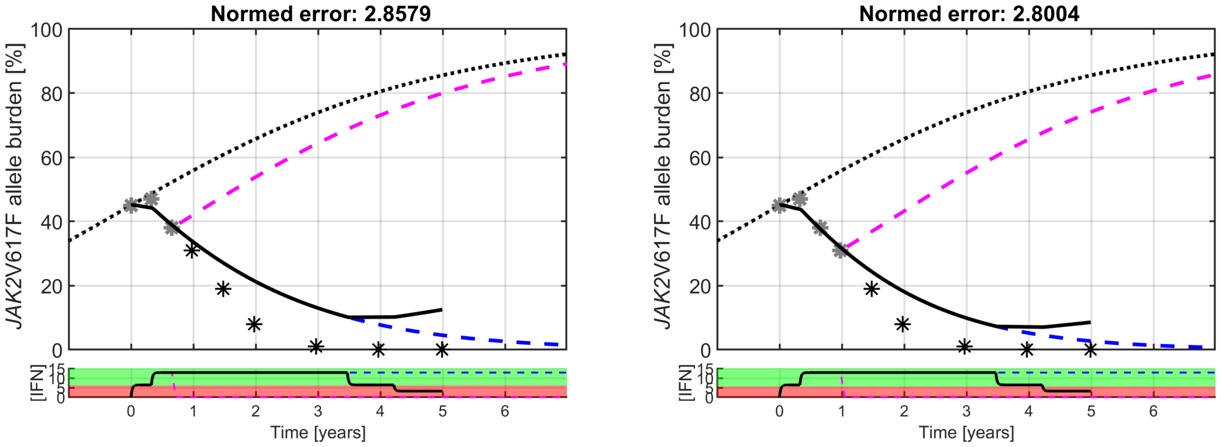

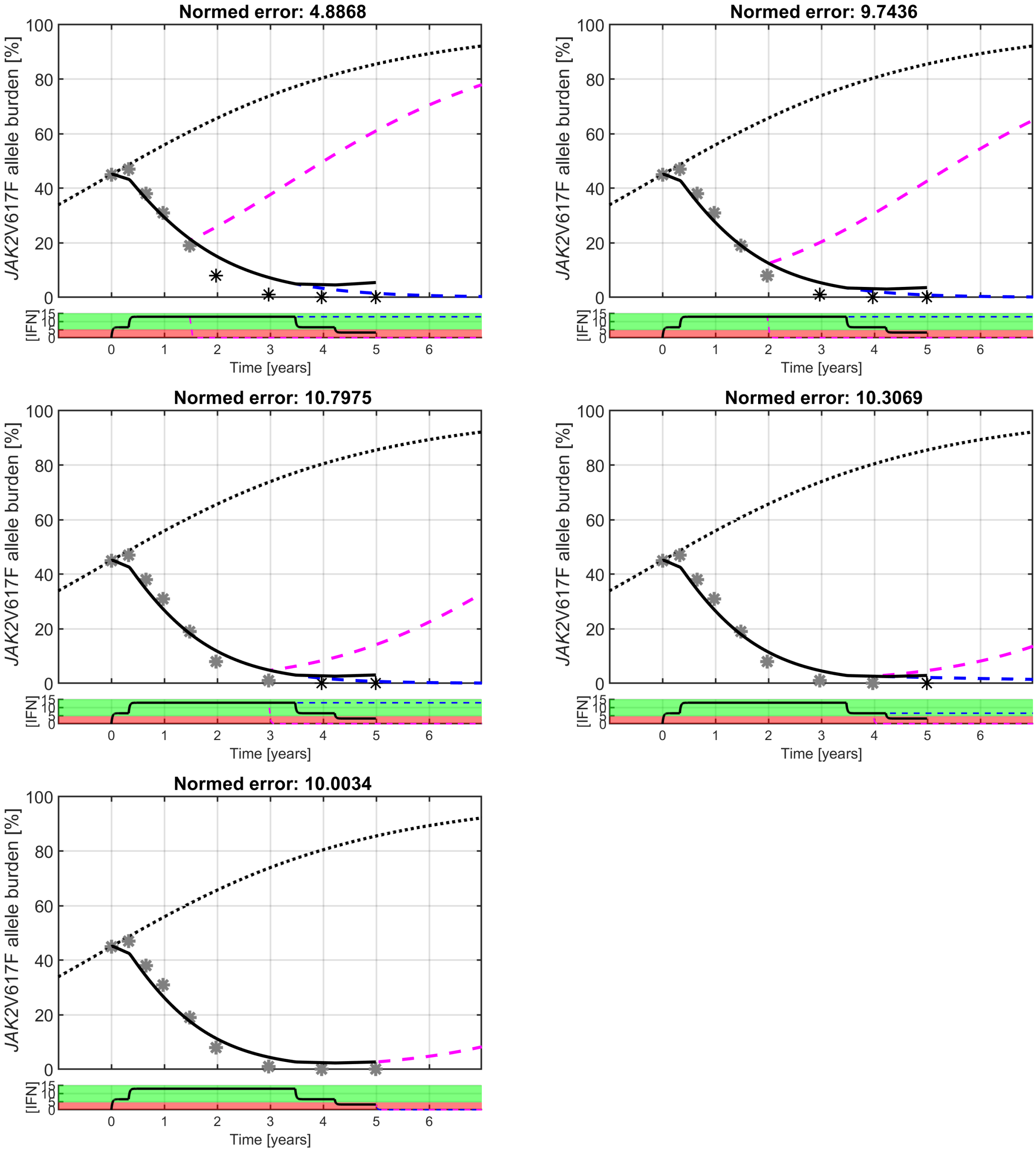

Patient P199

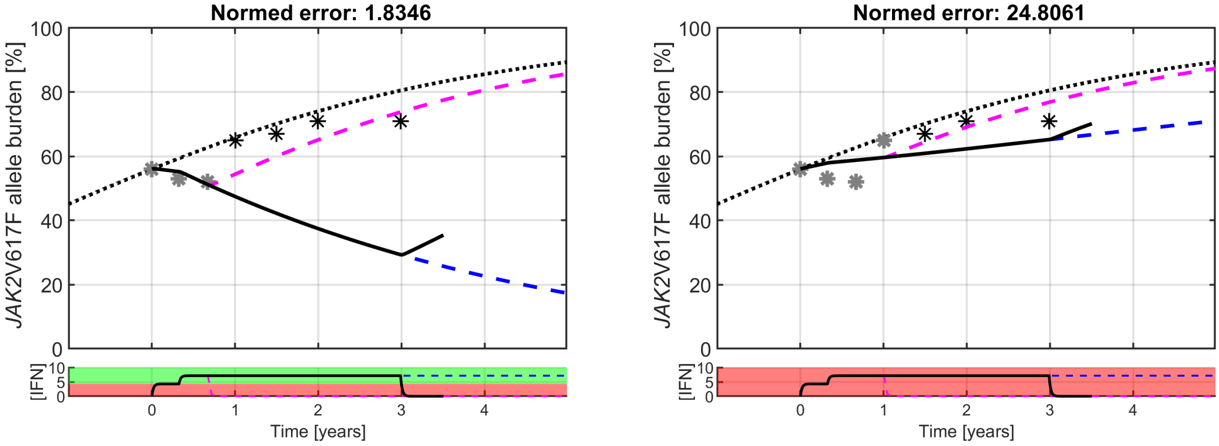

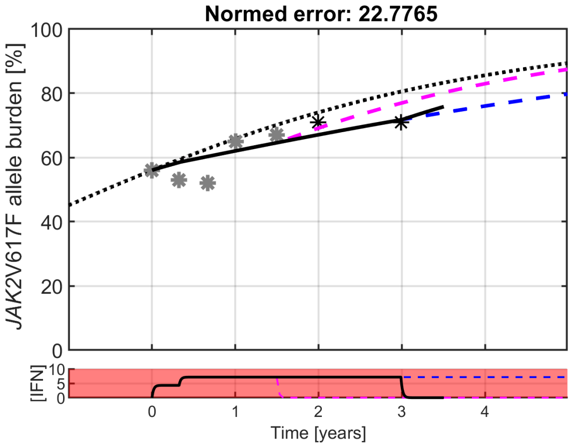

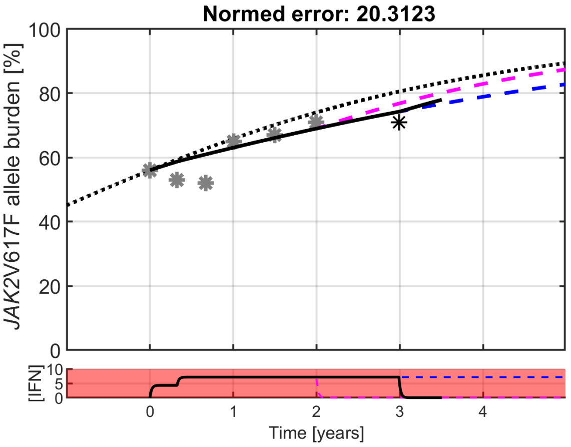

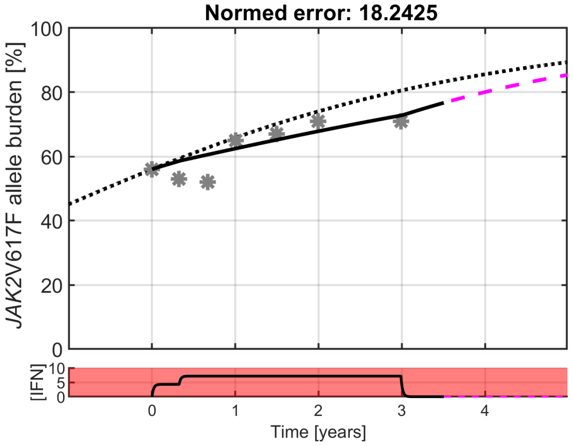

Patient P201

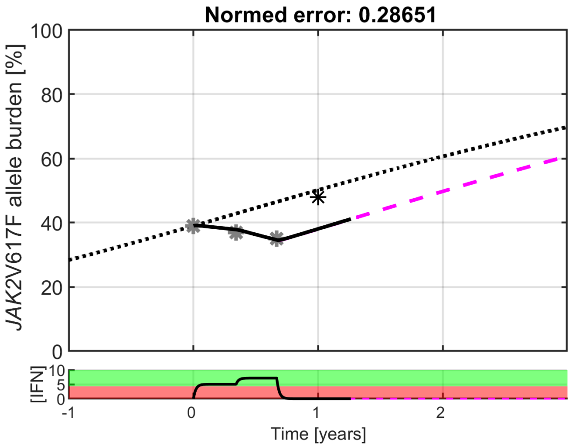

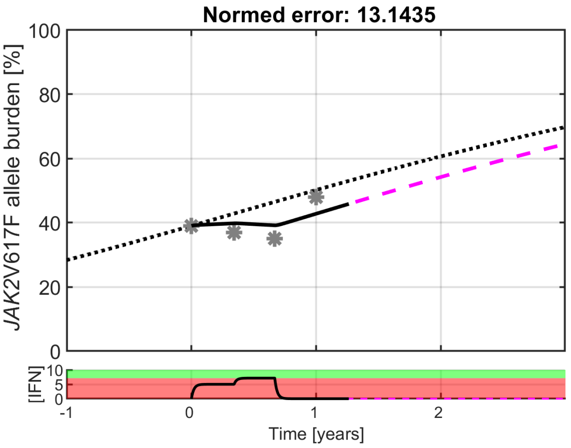

Patient P202

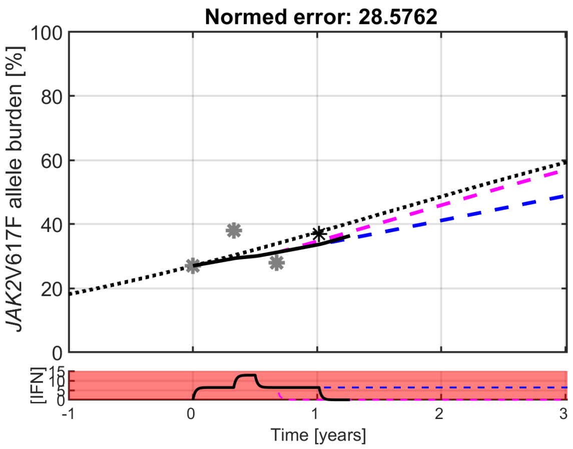

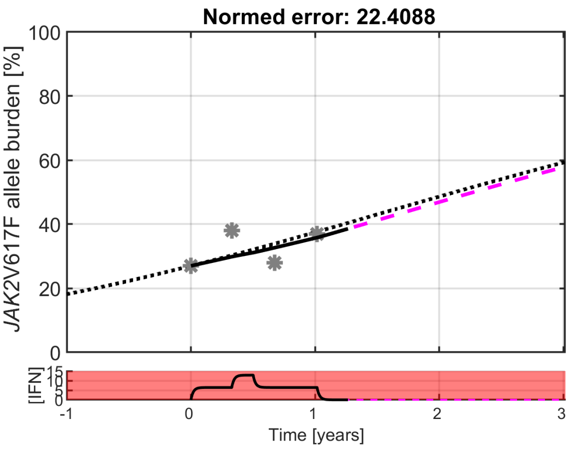

Patient P203

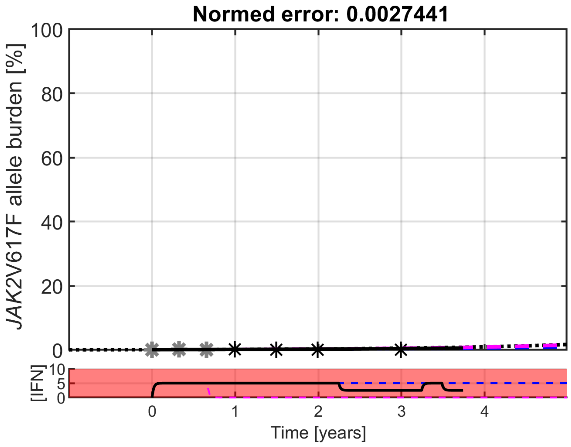

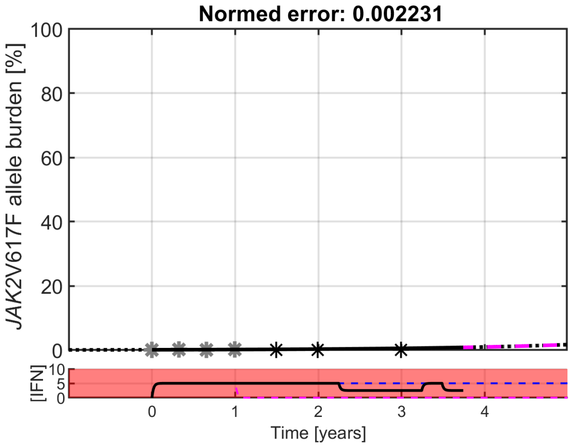

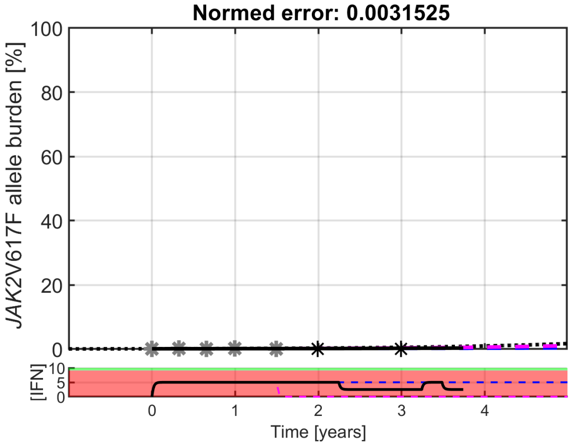

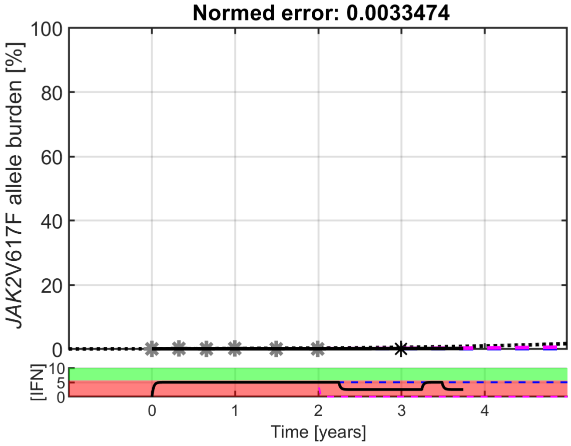

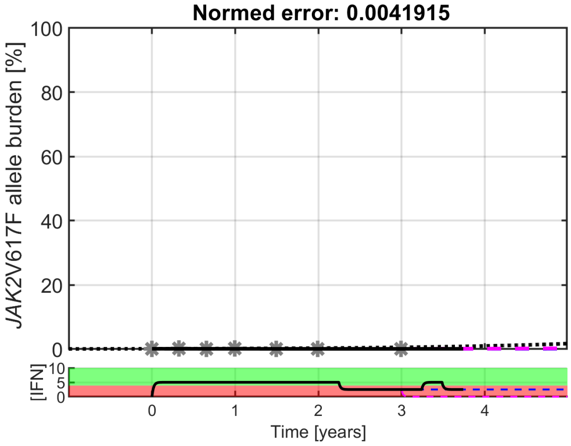

Patient P205

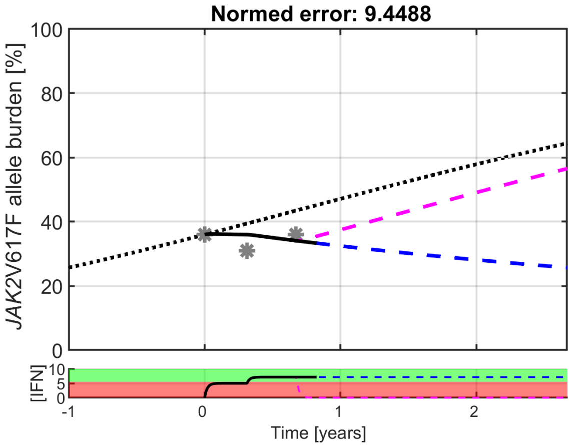

Supplement: Supplementary file 1 [file cancers-12-02119-s001.pdf]
